# ^13^C NMR-Based Chemical Fingerprint for the Varietal and Geographical Discrimination of Wines

**DOI:** 10.3390/foods9081040

**Published:** 2020-08-02

**Authors:** Alberto Mannu, Ioannis K. Karabagias, Maria Enrica Di Pietro, Salvatore Baldino, Vassilios K. Karabagias, Anastasia V. Badeka

**Affiliations:** 1 Department of Chemistry, University of Turin, Via Pietro Giuria, 7, I-10125 Turin, Italy; salvatore.baldino@unito.it; 2 Laboratory of Food Chemistry, Department of Chemistry, University of Ioannina, 45110 Ioannina, Greece; vkarambagias@gmail.com (V.K.K.); abadeka@uoi.gr (A.V.B.); 3 Department of Chemistry, Materials and Chemical Engineering “G. Natta”, Politecnico di Milano, Piazza L. da Vinci 32, 20133 Milan, Italy; mariaenrica.dipietro@polimi.it

**Keywords:** wine, ^13^C NMR fingerprint, MANOVA, factor analysis, *k*-nearest neighbors, partial least squares-discriminant analysis, variable importance in projection, varietal discrimination, geographical discrimination

## Abstract

A fast, economic, and eco-friendly methodology for the wine variety and geographical origin differentiation using ^13^C nuclear magnetic resonance (NMR) data in combination with machine learning was developed. Wine samples of different grape varieties cultivated in different regions in Greece were subjected to ^13^C NMR analysis. The relative integrals of the ^13^C spectral window were processed and extracted to build a chemical fingerprint for the characterization of each specific wine variety, and then subjected to factor analysis, multivariate analysis of variance, and *k*-nearest neighbors analysis. The statistical analysis results showed that the ^13^C NMR fingerprint could be used as a rapid and accurate indicator of the wine variety differentiation. An almost perfect classification rate based on training (99.8%) and holdout methods (99.9%) was obtained. Results were further tested on the basis of Cronbach’s alpha reliability analysis, where a very low random error (0.30) was estimated, indicating the accuracy and strength of the aforementioned methodology for the discrimination of the wine variety. The obtained data were grouped according to the geographical origin of wine samples and further subjected to principal component analysis (PCA) and partial least squares-discriminant analysis (PLS-DA). The PLS-DA and variable importance in projection (VIP) allowed the determination of a chemical fingerprint characteristic of each geographical group. The statistical analysis revealed the possibility of acquiring useful information on wines, by simply processing the ^13^C NMR raw data, without the need to determine any specific metabolomic profile. In total, the obtained fingerprint can be used for the development of rapid quality-control methodologies concerning wine.

## 1. Introduction

The global impact of wine production in terms of economy and people involved is not negligible. According to the report of the International Organization of Vine and Wine (OIV), the world wine production in 2016 reached 259,500.000 hectoliters. Among the largest producers, Italy holds the first position, followed by France and Spain. The wine production of Romania is of medium size and constant, while that of the United States increased from 2015 to 2016. Referring to 2016, in South America, and particularly in Argentina, Chile, and Brazil, the wine production decreased, while the opposite trend was observed in Australia and New Zealand. Greece holds the 16th position in the world ranking, with a wine production that reached 2.6 million hectoliters in 2016. Currently, five countries consume approximately 50% of the world’s wine production: United States (13%), France (12%), Italy (9%), Germany (8%), and China (7%) [1]. Considering the size of the wine market, there is a great interest in the clarification and maintenance of wine quality and varietal authenticity. It is then clear, that more effective analytical methods are needed to control and monitor the quality of wine, improve the production stages, increase the knowledge about its composition, and more generally, reach the consumer needs at ahigh level.

The development, however, of new tools for quality assessment in food and related areas represents a continuous challenge for researchers. Within the many available analytical techniques for food analysis and control, fast and sustainable nuclear magnetic resonance (NMR) spectroscopy for food and biological samples has largely affirmed its utility and advantages especially in analyses based on metabolomics [2,3,4]. Many protocols based on different analytical techniques have been reported with the aim of distinguishing between wine samples on the basis of cultivar, geographical, biological, and processes information. Some techniques, such as high-performance liquid chromatography coupled to diode array detector (HPLC-DAD) [5], mass spectrometry (MS) coupled with inductively coupled plasma (ICP/MS) [6], or with gas chromatography (GC) [7,8] require specific sample treatments as the solid-phase micro-extraction (SPME). On the other hand, other techniques, such as IR [9] or Raman [10] spectroscopy have been employed in combination with chemometrics to determine a fingerprint specific to each wine type, resulting in effectiveness and time saving. More specifically, the ^1^H and ^13^C NMR spectra of wine and a deuterium natural abundance NMR method (SNIF-NMR: site-specific natural isotope fractionation) can be exploited to determine a molecular fingerprint, which can be used directly for the characterization and quantitative identification of specific metabolites (ethanol, glycerol, 2,3-butanediol, ethyl acetate, malic acid, tartaric acid, succinic acid, lactic acid, alanine, valine, proline, choline, gallic acid, etc.)in samples of different varieties and geographical origins [11,12]. The wine metabolome is based on the identification of either the compositional or spectrometric characteristics, and it depends on several factors such as agronomic practices and pedoclimatic conditions [2], grape variety [5,13], wine-making/fermentation strategies [14], and geographical origin [5,15]. The wine metabolome can be used for the quality control and authentication of wine and for the support of the already known labeling of protected designation of origin (PDO) and protected geographical indication (PGI). However, all the aforementioned NMR-based methodologies require an excessive amount of analysis time and the purchase of standards for the quantification of the metabolites. Regarding the Greek wine varieties, in addition to the most important wines Assyrtiko, Moschofilero, Agiorgitiko, and Xinomavro, numerous other grape varieties are present in different territories (Augoustiatis, Kakotrygis, Kalavritiko Black, Krassato, Lagorthi, Savatiano, etc.) which are all vinifiable, whether they go toward vinification of monovarietals or are used as part of wine blends. In all cases, these contribute to the character and diversity of the wines, showcasing them as one-of-a-kind [16]. In this context, the aim of the present study was to develop a fast, economic, eco-friendly, and effective analytical methodology for the classification of commercial wine samples of different varieties, cultivated in the Hellenic zone, in combination with statistical tools. Thus, in the present study, PDO and PGI wine samples were subjected to ^13^C NMR analysis, and a chemical fingerprint characteristic of each sample was determined and processed through statistical analysis. As an outcome, the study contributes to the rapid quality-control analysis of different wine varieties from different regions and the support of the identical character of certified wines distributed by the domestic wine industry in the local or international markets.

## 2. Materials and Methods

### 2.1. Wine Samples and Handling

Wine samples of different varieties, bottled in 2015–2018, were considered in the study. Most of the wine samples were of protected designation of origin (PDO) and protected geographical indication (PGI). Table 1 lists the harvesting year, type, geographical origin, variety, and alcohol volume for each wine sample. For the present study, the samples were categorized in the following eight groups: Group A: Syrah + Syrah-based wines (sample nos. 3, 4, 5, 27, 31, 32); Group B: Muscat (sample nos. 9, 10, 12); Group C: Xinomavro+Xinomavro-based wines (sample nos. 7, 33, 34, 35); Group D: Assyrtiko+Assyrtiko-based wines (sample nos. 19, 23, 29); Group E: Malagouzia (sample nos. 8, 20, 22); Group F: Other wine varieties (sample nos. 2, 6, 11, 13, 14, 15, 16, 18, 21, 24, 28, 30, 36); Group G: Agiorgitiko (sample nos.25, 26); and Group H: Debina (sample nos.1, 17). For the geographical origin differentiation, wine samples were grouped according to Table 2. Three different bottles of each wine variety (N = 36 × 3ib, where ib: independent bottles of wine) were subjected to the study, and the mean value of the data arising from the three independent bottles was used further in the processing of results.

### 2.2. Chemicals 

Deuterated water (D_2_O) and tetramethylsilane (TMS) were purchased from Sigma–Aldrich, Italy.

### 2.3. ^13^C NMR Analysis

Wine samples were collected from the original bottle and analyzed in pure form (0.4 mL). Proton-decoupled ^13^C NMR analysis, in the presence of 30 mL of D_2_O containing 10% of tetramethylsilane (TMS, internal reference) (Sigma-Aldrich Italy), was then conducted. As the TMS is almost insoluble in D_2_O, when reference was not detected, the typical signal at 60.1 ppm of glucose, common to all the samples, was taken as an internal reference for scale adjusting [17]. Each sample was analyzed in triplicate and the mean value of the three replicates was used for the statistical analysis. The NMR measurements were performed on a Bruker NEO 500 spectrometer equipped with a 5 mm pulsed-field z-gradient broad band BBFO probe and a variable-temperature unit and operating at 125 MHz for the carbon nucleus. The following acquisition parameters were applied for each measurement: time domain 32K, 2500 scans, and a spectral width of 240 ppm. The spectral data from the ^13^C NMR analysis were processed as follows: the entire spectrum in the range 0–220 ppm was divided in parts of 0.1 ppm (bin = 0.1 ppm), and all the corresponding signals were extracted and saved in a text file. For each signal, the value of the corresponding integral was considered for the statistical analysis. About 2500 integrals from each spectrum were extracted and subjected to MetaboAnalyst processing as described in the following section.

### 2.4. Statistical Analysis

Given the large amount of data contained in the processed ^13^C NMR spectra, as a first step, a dimension-reduction technique (factor analysis) was used to investigate whether a satisfactory total variance could be explained in the multidimensional space, among wine samples of different varieties. The basic principle of factor analysis is to provide a reduction in the correlated variables (in our case, the ^13^C NMR integrals) to highlight those that have the higher communalities (common variance shared by factors with specific variables) of the independent latent variables. A higher communality (≥0.4) indicates that larger amount of the variance in the variable has been extracted during the factor analysis. The extraction method was principal component analysis (PCA). The efficiency (accuracy and strength) of factor analysis was checked by the Kaiser–Meyer–Olkin (KMO) test, which comprises a measure of how well-suited the data set is for factor analysis. The value that was considered acceptable during the analysis was that of KMO ≥0.50. An additional criterion for factor analysis was the Bartlett’s test of sphericity, which is a test that highlights the hypothesis that the correlation matrix used in the analysis is an identical matrix. Small probability values (*p* < 0.05) indicate that factor analysis may be useful with data treatment [18]. For the classification of wine samples according to variety, multivariate analysis of variance (MANOVA) in combination with *k*-nearest neighbors (*k*-NN) analysis was implemented. At first, MANOVA indicated the significant (*p* < 0.05) ^13^C NMR integrals in relation to the wine variety. Thereafter, *k*-NN, as a supervised classification technique, was used to classify wine samples according to variety. The group of wine samples was considered as the factor variable, whereas the ^13^C NMR integrals were the independent variables. Finally, the reliability of the performed analyses was tested using the Cronbach’s alpha index. The calculation of Cronbach’s alpha has become a common practice for scientists to test the reliability level of research [19] when multiple-item measures or multi-element parameter analysis are employed. Cronbach’s alpha test provides a measure of the internal consistency of a group of objects or scale. It is expressed as a number between 0 and 1. On the other hand, the internal consistency describes the extent to which all the items in a test measure the same concept or construct, and hence, it is connected to the inter-relatedness of the items within the test, to ensure validity. In addition, reliability analysis estimates the amount of measurement error in a test. Therefore, for high-quality tests it is mandatory to evaluate the reliability of data supplied in an examination or a research study. For the geographical origin differentiation of wine samples, given the challenging grouping of samples (Table 2), multivariate analyses such as principal component analysis (PCA), partial least squares-discriminant analysis (PLS–DA), and variable importance in the projection (VIP) were performed using the online tool MetaboAnalyst 4.0 [20]. The PLS regression was performed by employing the *plsr* function provided by *R pls* package [21]. Classification and cross-validation were performed using the corresponding wrapper function contained in *caret* package [22]. Statistical analysis was performed by the SPPSS statistics software version 26.0 and the MetaboAnalyst online tool [23,24]. The raw data were filtered, normalized by the sum, and then processed by the specific statistical tool (vide infra). Filtering of the raw data was conducted to remove variables of very small values (close to baseline or detection limit) and variables that are near-constant values throughout the experiment conditions (housekeeping or homeostasis) [25]. All the variables exhibiting a relative standard deviation (RSD) >10% were not considered in the statistical analysis.

## 3. Results and Discussion

### 3.1. Commercial Characteristics of the Wine Samples—Consumer Issues

The product status and characteristics of the wine samples herein considered according to the brand labeling are presented and discussed exhaustively in the Supplementary Materials. The information presented should be considered as a typical screenshot for the reader/consumer (consumer’s choice) aimed to a rapid (and available) knowledge on these wines and not an advertisement of these specific brands. The consumer’s need should fill the gap between researchers and food community, as the cooperation between the two societies would guarantee their welfare and the authenticity of the wine products introduced in the international market.

### 3.2. ^13^C NMR Fingerprints of Wine Samples—MANOVA Analysis

**Figure 1:**
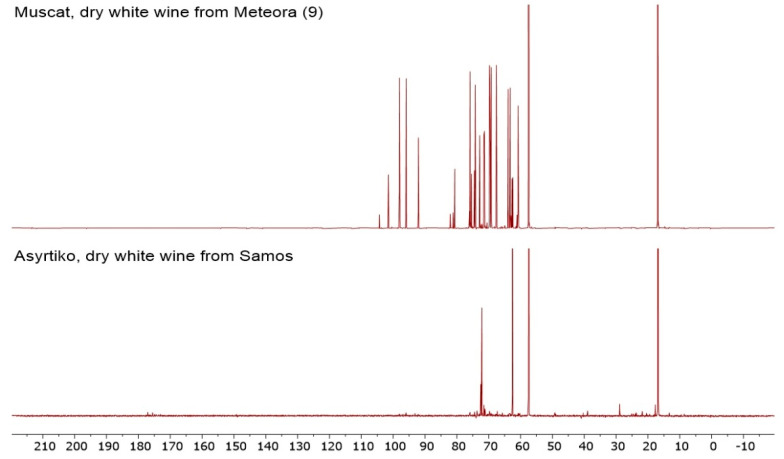
Qualitative ^13^C nuclear magnetic resonance (NMR) spectra comparison between Assyrtiko (Meteora) and Muscat (Samos Island) wines.

**Figure 2:**
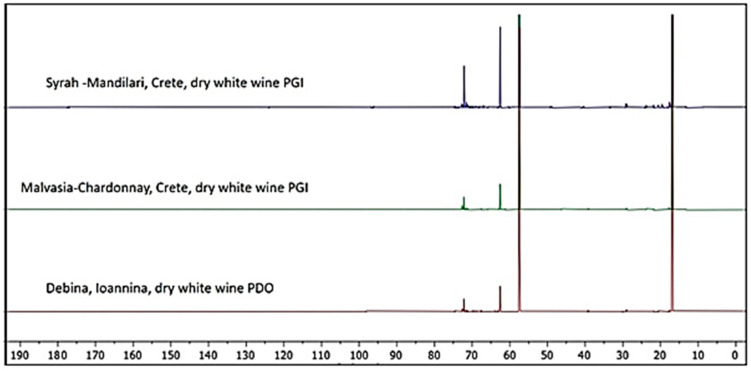
^13^C NMR spectra comparison between Syrah–Mandilari (Crete), Malvasia–Chardonnay (Crete), and Debina (Zitsa, Ioannina) wines.

As an alternative methodology to the classical employment of standards or comparison handling of the chemical shift information of the wine metabolites based on the related references and databases [12,26,27,28] for the characterization of wine, we propose the determination of a molecular fingerprint on the basis of ^13^CNMR measurements. This approach allows us, with the proper combination of specific NMR technique (^1^H, ^13^C, ^31^P) and statistical tools, to obtain a chemical fingerprint of samples that can be employed in discrimination and quality assessment. Such a fingerprint is representative of the chemical composition of the samples and can be used for classification purposes without determining specific compositional characteristics [29]. In addition, any chemical additive due to the agricultural process or wine production will be treated as valuable information. Products present in low amounts (traces) do not affect the statistical outcome as they are filtered as described above. For each sample, the spectral region from 0 to 220 ppm was divided in intervals of 0.1 ppm (bin = 0.1 ppm). The corresponding integrals were considered and subjected to statistical analysis (Supplementary Materials Figure S1). The time of analysis was about 2 h for ^13^C NMR, while the extraction of the raw data from the FID (Free Induction Decay) required a few minutes. The proposed technique is different from the already reported employment of NMR for the determination of ethanol content in wine [30] or for the recognition of specific chemical groups [31]. Indeed, the implementation of a chemical fingerprint based on the entire spectrum allows indicating even small differences between samples, which can be highlighted only by specific combination of patterns of signals through an opportune statistical multivariate analysis. Herein, ^13^C NMR has been preferred to faster the ^1^H NMR analysis because carbon frequencies are spread over a larger spectral window, enhancing resolution and reducing overlaps. In fact, ^1^H NMR signals range from 0 to about 12 ppm, while in the case of ^13^C NMR the signals window ranges from 0 to about 220 ppm. Thus, numerous frequencies were recorded for each wine sample (Supplementary Materials Table S1), allowing enough data for an exhaustive statistical analysis. The determination of this specific chemical fingerprint does not require the employment of any standard or database access, thus proving quite economic when compared with other methodologies of food classification. Moreover, no specific manipulation or treatment of the samples is needed, reducing the employment of chemicals and toxic solvents and, thus, enhancing the sustainability of the methodology. Regarding the studied wine samples, significant (*p* < 0.05) differences were recorded in the ^13^C NMR integrals according to the wine variety using MANOVA (Table 3). The power of the combination between ^13^C NMR and statistical analysis is evident even from a qualitative comparison between the spectra. If, e.g., the spectra relative to Muscat PDO and Assyrtiko (Meteora) wines are compared, important differences can be noted (Figure 1). It is evident that dry white wines from Meteora (Assyrtiko grapes) and from Samos (Muscat grapes) have different composition (Figure 1). In particular, the relative amount of carbonyl compounds (between 170 and 180 ppm) is higher in the wine from Meteora, while the Samos sample is richer in chemicals with carbons close to heteroatoms or involved in multiple bonds (between 60 and 110 ppm). On the contrary, the aliphatic part of the spectrum (between 0 to 50 ppm) is more populated with signals in the Meteora wine sample. If in some cases, such as the one reported in Figure 1, it is possible to qualitatively distinguish between wine samples, other comparisons can be more difficult. In Figure 2, samples of Syrah–Mandilari (Crete), Malvasia–Chardonnay (Crete), and Debina (Zitsa, Ioannina) are compared. Despite the known differences between these three wines, it is evident that, in this specific case, a visual discrimination is not possible (Figure 2). Nevertheless, the ^13^C NMR spectra contain much information, not directly visually accessible, which, if extracted and properly processed, can be used for sample discrimination. This matter will be discussed in the next sections.

### 3.3. Classification of Wine Samples According to Variety Using the ^13^C NMR Integrals and Chemometrics

#### 3.3.1. Factor Analysis

Factor analysis showed that 9 components (^13^C NMR integrals of the respective wine varieties) could explain 90.549% (ca. 90.55%) of the total variance. The first component (eigenvalue of 7.169) explained 21.770% of the total variance, whereas the second component (eigenvalue of 5.031) explained 14.373% of the total variance. Similarly, the third component (eigenvalue of 4.143) explained 11.838% of the total variance. The fourth component (eigenvalue of 3.410) explained 9.743% of the total variance. The fifth component (eigenvalue of 2.903) explained 8.295% of the total variance. The sixth component (eigenvalue of 2.598) explained 7.422% of the total variance. The seventh principal component (eigenvalue of 2.533) explained 7.238% of the total variance. The eighth principal component (eigenvalue of 1.852) explained 5.291% of the total variance. Finally, the ninth principal component (eigenvalue of 1.603) explained 4.579% of the total variance. The first component was more highly correlated with the ^13^C NMR integrals of Syrah/Merlot/Cabernet wines (Syrah-based wines) from Korinthos (correlation value of 0.975). The second component was more highly correlated with the ^13^C NMR integrals of Xinomavro wines from Epanomi (correlation value of 0.994). The third component was more highly correlated with the ^13^C NMR integrals of semi dry rosé wines from Samos Island (correlation value of 0.979). The fourth component was more highly correlated with the ^13^C NMR integrals of Debina wines from Zitsa (Ioannina) (correlation value of 0.995). The fifth component was more highly correlated with the ^13^C NMR integrals of Malagouzia wines from Meteora (correlation value of 0.974). The sixth component was more highly correlated with the ^13^C NMR integrals of Muscat wines from Samos Island (correlation value of 0.975). The seventh component was more highly correlated with the ^13^C NMR integrals of Syrah–Xinomavro wines (Syrah-based wines) from Naoussa (correlation value of 0.742). The eighth component was more highly correlated with the ^13^C NMR integrals of Xinomavro-based wines and pure Xinomavro wines from Naoussa (correlation value of 0.962). Finally, the ninth component was more highly correlated with the ^13^C NMR integrals of Xinomavro+Xinomavro-based wines and wine samples of other varieties from Macedonia and Trifylia (correlation value of 0.895). The KMO measure of sampling adequacy was 0.776, whereas Bartlett’s test of sphericity (X^2^ = 1,962,733.748, df = 595, *p = 0.000* < 0.001) approved the effectiveness of analysis.

#### 3.3.2. k-NN Analysis

**Figure 3:**
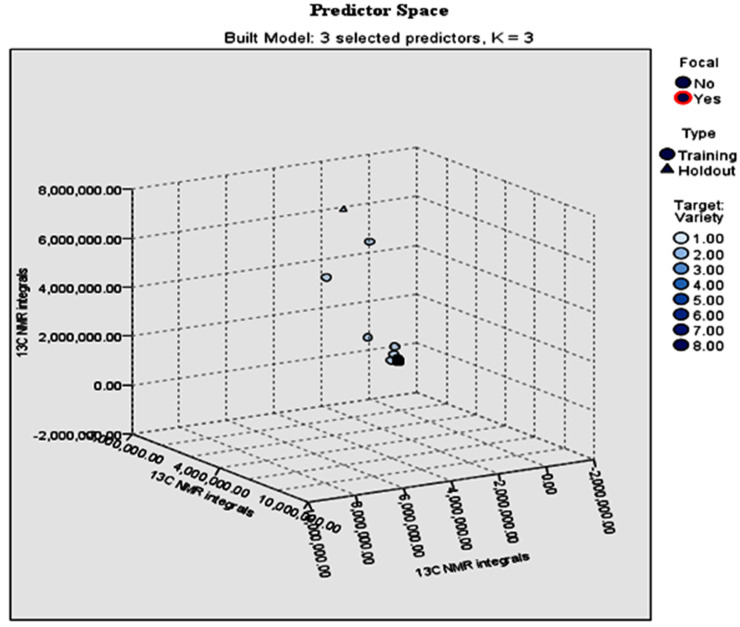
Classification of wine according to variety using the ^13^C NMR integrals in combination with *k*-NN analysis. 1: Syrah+Syrah-based wines; 2: Muscat; 3: Xinomavro+Xinomavro-based wines; 4: Assyrtiko+Assyrtiko-based wines; 5: Malagouzia; 6: other wine varieties; 7: Agiorgitiko. 8: Debina. This chart is a lower-dimensional projection of the predictor space, which contains a total of 26 predictors.

During the *k*-nearest neighbors analysis (*k*-NN), the wine variety (8 varieties) was considered as the target parameter, whereas the significant ^13^C NMR integrals indicated by MANOVA were the features. The classification ability of the model was evaluated by application of training and holdout partitions. In total, the recorded number (N) of the ^13^C NMR integrals was N = 17,584. In the training sample, the 70% of the cases (N = 12,336 ^13^C NMR integrals) representing the 70.2% of the population of samples were randomly assigned to partitions, while the rest of the cases (N = 5248 ^13^C NMR integrals), representing the 29.8% of the population of samples, were assigned to the holdout sample.The overall classification rates were 99.8% and 99.9% for training and holdout samples, respectively (Table 4). As can be observed, this was an almost perfect classification for the different wine varieties based on the number of the ^13^C NMR integrals. The respective error summary of the *k*-NN model (percent of records incorrectly classified) was 0.2% for the training and 0.1% for the holdout sample. Figure 3 shows the classification of wine according to variety using the ^13^C NMR integrals in combination with *k*-NN analysis. This chart is a lower-dimensional projection of the predictor space, which contains a total of 26 predictors. In a similar study, Godelmann et al. [32] analyzed different German wine varieties using ^1^H NMR spectroscopy with multivariate data analysis, including principal component analysis, (PCA), linear discriminant analysis (LDA), and multivariate analysis of variance (MANOVA). The grape varieties Pinot Noir, Lemberger, Pinot blanc/Pinot gris, Müller–Thurgau, Riesling, and Gewürztraminer, were successfully classified (ca. 97%), and it was reported that the metabolites mainly responsible for the observed differentiation of the wine varieties were shikimic acid, caftaric acid, and 2,3-butanediol. Wines from the varieties Agiorgitiko, Mandilaria, Moschofilero, and Assyrtiko produced in Greece [27] and Cabernet Sauvignon, Merlot, Feteasca Neagra, Pinot Noir, and Mamaia wines, produced in Romania [33] were classified (85.7% correct classification rate) according to variety using NMR-based metabolomics and LDA. Cabernet Sauvignon and Shiraz wines, produced in Australia, showed a clear separation based on their respective metabolite profile. In particular, Cabernet Sauvignon had higher levels of proline, while Shiraz wines had higher levels of sugars (fructose and glucose), succinate, methanol, acetate, and some aliphatic amino acids [34].

#### 3.3.3. Reliability Analysis

Finally, during the reliability analysis all cases (N = 17,584 ^13^C NMR integrals) were valid and subjected to the test. Results showed that Cronbach’s alpha had the value of 0.836, indicating a good reliability of the analysis carried out. The variance error of the analysis (random error) was: 0.836 × 0.836 = 0.698896; 1 − 0.698896 = 0.301104 = ~0.30. Therefore, the presented data with a reliability level of 0.836 have a random error of 0.30.

## 4. Classification of Wine Samples According to Geographical Origin Using the ^13^C NMR Integrals and Chemometrics

**Figure 4:**
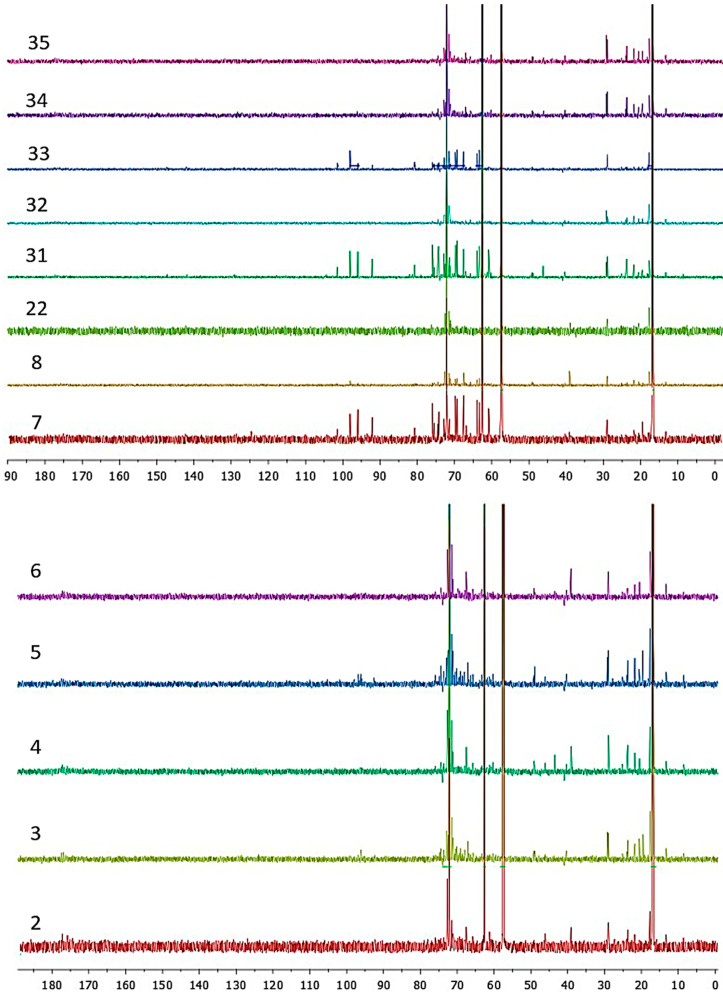

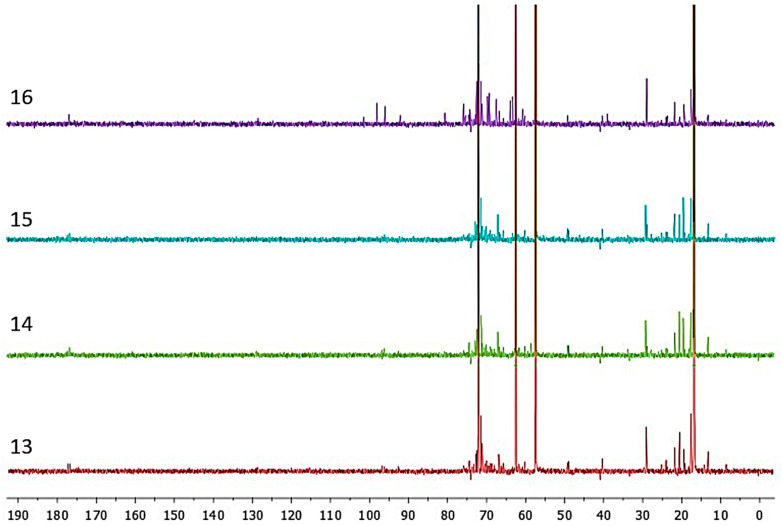
NMR spectra of wine samples from Macedonia (**top**), Crete (**center**), and Ileia (**bottom**) regions.

Herein, a combination between the ^13^C NMR integrals and partial least squares-discriminant analysis (PLS-DA) was successfully used for this purpose. PLS-DA was selected after unsuccessful attempts to clusterize NMR data through PCA, which is also reported for comparison. Nevertheless, the relatively low number of wine samples originating from not all the major wine regions in Greece may comprise limitations of the study. A high number of the proton-decoupled ^13^C NMR spectra related to the wine samples were acquired and processed as reported in Section 2.3, and about 2500 integrals for each spectrum were extracted and considered for the statistical analysis. In that sense, a chemical fingerprint characteristic of each group was obtained from the PLS-DA data through VIP representation. The list reported in Table 1 is very heterogeneous and challenging for a possible discrimination, as the considered wine samples differ from each other in many characteristics. In general, different grapes were considered in terms of color (red and white) and variety. Furthermore, wines arising from different geographical areas in Greece were considered, which were prepared according to different processes. In this context, an effort to classify wine samples on the basis of their geographical origin was carried out (Table 2). The classification reported in Table 2 considers samples for each group of wines that are very different in grape type and year of production. It is a general classification based on the geographical origin of samples. Thus, the possibility of finding specific markers that allow distinguishing between these groups is of interest. The difficulties related to a visual classification based on the analysis of the ^13^C NMR data can be highlighted by comparing the spectra of wine samples from Macedonia, Crete, and Ileia regions (Figure 4).

From the visual analysis of the spectra reported in Figure 4, it is possible to notice that same differences are appreciable within samples. For instance, samples 7, 8, 32, and 34 showed a group of signals between 90 and 105 ppm, which correspond to the window usually associated to C–C double bonds or to a carbon on a heteroatom. On the other hand, the wine samples from Crete, as well as from Ileia regions, showed signals in the carbonyl area, between 170 and 180 ppm (Supplementary Materials Figure S1). Even though it is possible to qualitatively analyze the NMR data, a direct relationship between the easily visible ^13^C signals and the geographical origin of wines cannot be found. One classical route for grouping the considered samples would be to identify specific metabolites that are characteristics for each wine and to find a relationship between such metabolites and the geographical origin of wine. Nevertheless, such an approach would include an exhaustive characterization of the wine samples with the employment of standards and dedicated libraries. Our analytical proposal lies into the fact that an analytical signal, such as the ^13^C NMR FID, contains much hidden information that can be extrapolated with multivariate statistical analysis. As matter of fact, to reach the target of grouping the analyzed wine samples on the basis of the different geographical origin, it would not be necessary to characterize any metabolite, but just to find the proper statistical method able to identify the differences in the whole FID between wines belonging to the different geographical groups.

Godelmann et al. [32] reported an analogue study where German wines were classified by determining a specific chemical fingerprint for each wine by a combination of ^1^H NMR and statistical analysis, including PCA and PLS-DA. As an evolution of such approach, with the aim of increasing the amount of information constituting the chemical fingerprint, ^13^C NMR raw data were considered. In fact, the acquisition window of ^13^C NMR (from 0 to 220 ppm) is much wider than the ^1^H NMR one (from 0 to about 10 ppm), resulting in a more elevated number of points and information.

**Figure 5:**
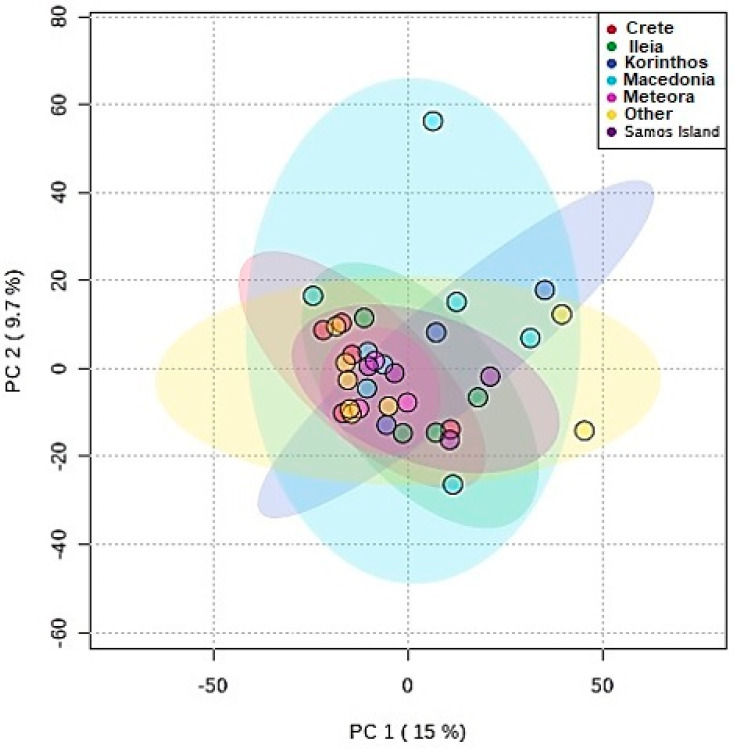
2D score plot for wines of different geographical origin using principal components analysis (PCA).

At first, PCA was applied to seek a suitable statistical methodology that would allow the discrimination of wine samples according to geographical origin based on the ^13^C NMR data (Figure 5).

Looking at the 2D score plot reported in Figure 5, it is evident that PCA-based discrimination of samples of different geographical origin was not possible. In fact, other relevant factors, such as the grape variety or production conditions, including the production year (vintage), impede the building of an unsupervised classification based on the principal components.

### Partial Least Squares-Discriminant Analysis (PLS-DA)

As an alternative statistical method to the unsupervised method of PCA, the supervised PLS-DA was considered. PLS regression is a supervised statistical model particularly effective when categorical variables are considered. It overcomes the difficulties of PCA in cases where more variables than observations exist [35,36]. It is known that the PLS algorithm often is able to generate groups but with a low reliability, which can usually be highlighted by a cross-validation procedure [37]. In our case, the cross-validation procedure gave the following results, indicating a model not affordable (Table 5).

Nevertheless, even in this case, useful information about the structure of the groups can be extrapolated [37].

**Figure 6:**
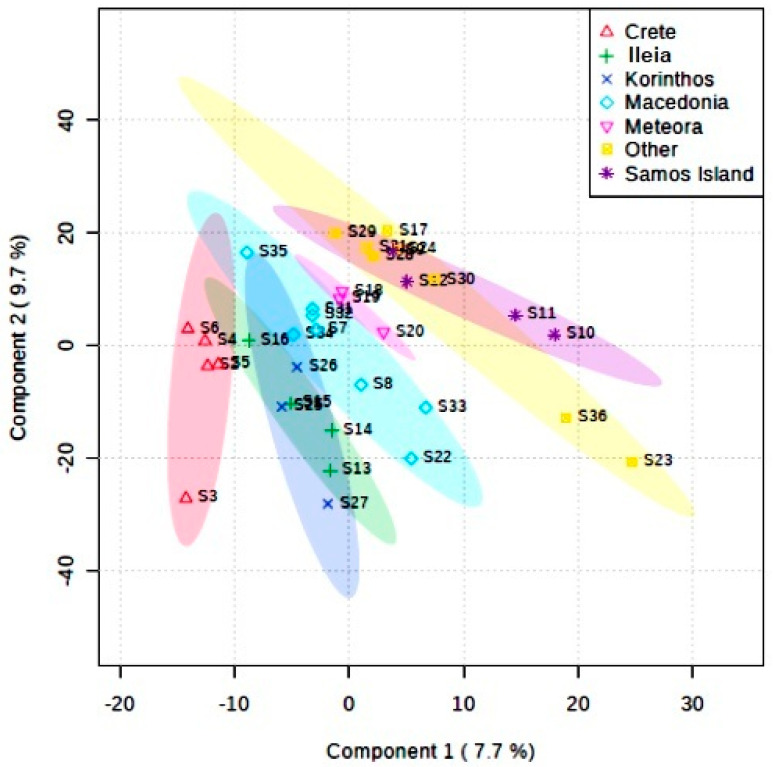
2Dscore plot for wines of different geographical origin using PLS-DA.

Herein, PLS-DA allows the selection of the most relevant markers (in this case, represented by specific combination of ^13^C signals) that can be used to determine a chemical fingerprint representative of each group. In Figure 6, 2D score plots between the selected components are reported.

Looking at the 2D plot shown in Figure 2, it is possible to appreciate the better discrimination among wines of different geographical origin when PLS-DA regression analysis is used. Looking at the structure of the groups it is possible to gain information about the variability of wines within the same geographical origin. As expected, the group “other” shows a wider distribution as it contains different wines. Among them, Chardonnay from Trifylia (Messinia) and Assyrtiko from Drama appear more similar than expected. Within the wine samples from Crete, the Syrah–Mandilari dry red PGI shows a different fingerprint with respect to that of the other wines of the same group.

**Figure 7:**
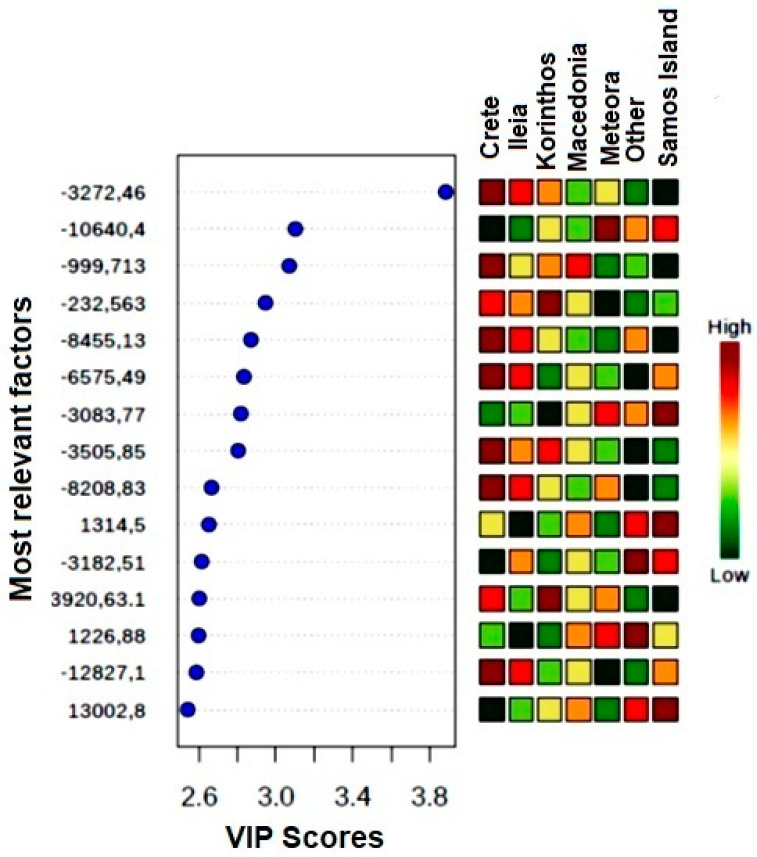
Variable importance in projection (VIP) score plot for the sevengeographical groups identified by PLS-DA.

On the basis of the data obtained by PLS-DA, variable importance in projection (VIP) was used to extract the most relevant combination of ^13^C NMR responses for each geographical group [38]. VIP is the result of a weighted sum of squares of the PLS-DA loadings, and it is related to the amount of explained Y-variable in each dimension. It can provide a fingerprint, characteristic for each group, defined on the basis of the geographical origin (Figure 7).

The 15 most representative factors that determine the variance are reported in the VIP plot. Each factor is expressed in each group (Crete, Ileia, Korinthos, Macedonia, Meteora, Samos Island, and other regions) with a different intensity. The overall combination for each group of the 15 factors and their intensities constitutes our chemical fingerprint. It is representative of the wines arising from a specific geographical area. As the statistical analysis has been carried out on the basis of portions of the ^13^C NMR FID, no physical meaning is associated to each of the 15 factors extracted. Nevertheless, these generated variables constitute a unique numeric code that is characteristic of the considered geographical groups.

Proceeding further to a comparison between the varietal and geographical origin discrimination results and the *k*-nearest neighbor and discriminant analyses, it should be stressed that *k*-NN is a completely non-parametric approach, given that no assumptions are made about the shape of the decision boundary. Therefore, we can expect this approach to dominate the discriminant analysis when the decision boundary is highly non-linear (the opposite holds for discriminant analysis). On the other hand, *k*-NN does not provide which predictors are significant (absence of a table of coefficients with *p*-values). The *k*-NN classification algorithm predicts the test sample’s category according to the *k*-training samples, which are the nearest neighbors to the test sample, and classifies it to the category that has the largest category probability [39].

## 5. Conclusions

The demanding need for certified products and, especially, for the parallel discrimination of already certified products in the market, as an evolution to consumer’s rights and for the needs of food choice support, sets the questioning of developing fast, accurate, economic, eco-friendly, and non-destructive methodologies, in combination with machine learning, for the beneficial characterization of such products. In the present case, wine samples of different varieties were unambiguously differentiated according to variety using ^13^C NMR spectral data in combination with chemometrics. More specifically, the combination of factor analysis (non-supervised statistical technique) with *k*-NN (supervised statistical technique) resulted in the exhaustive classification of wine samples according to variety. The analyst, using only the ^13^C NMR spectra, has the advantage in a short time to screen the varietal authenticity of wine samples of different varieties or blends of wine varieties. On the other hand, the classification of wines arising from different grapes and production years on the basis of the geographical origin can be challenging. Similarly, the statistical analysis of the ^13^C NMR raw data (hidden markers contained in the ^13^C NMR FID) can provide a way to reach such a target without the need of any specific molecular characterization and eliminating any sample collection problems by indicating specific chemical fingerprints using PLS-DA. Results, however, showed that the geographical origin discrimination of the wine samples used in this study is a more difficult task compared to the respective varietal discrimination. To the best of our knowledge, this is the first report in the literature presenting this approach for the characterization of the purity of different Greek wine varieties from different regions, constituting, therefore, the novelty of the present study and providing supportive knowledge for the wine industry and researchers in this field, in terms of the development of rapid methodologies for the characterization and differentiation of wine. To overcome any limitations regarding the effectiveness of the present study, a larger number of wine samples from all the major wine regions of Greece is required.

## Figures and Tables

**Table 1:** The wine samples used in the study.

Wine Samples	Year	Type	Geographical Origin	Variety	Alcohol Volume (%)
1	2017	Dry white wine—PDO	Zitsa, Ioannina	Debina	12.0
2	2017	Dry white wine—PGI	Crete	Malvasia di Candia Aromatica–Chardonnay	12.5
3	2016	Dry red wine—PGI	Crete	Syrah–Mandilari	13.0
4	2017	Dry Rosé wine—PGI	Crete	Syrah–Mandilari	13.0
5	2017	Dry red wine—PGI	Crete	Syrah	12.5
6	2017	Dry white wine—PGI	Crete	Vidiano	13.0
7	2018	Dry red wine—PGI	Epanomi, Macedonia	Xinomavro	13.5
8	2018	Dry white wine—PGI	Epanomi, Macedonia	Malagouzia	13.5
9	2017	Dry white wine—PDO	Samos Island	Muscat	15.0
10	2017	Dry white wine	Samos Island	Muscat	12.5
11	2018	Semi dry rosé wine	Samos Island	Samos red grapes	12.5
12	2011	Nectar, white wine naturally sweet—PDO	Samos Island	Muscat	14.0
13	2016	Dry red wine—PGI	Letrinoi, Ileia	Refosco	14.0
14	2015	Dry red wine—PGI	Letrinoi, Ileia	Daphne Nera–Mavrodafni	13.0
15	2016	Dry red wine—PGI	Ileia	Augoustiatis	13.0
16	2017	Dry white wine	Ileia	Albariño	13.5
17	2017	Demi Sec white wine	Zitsa, Ioannina	Debina	12.0
18	2016	Dry red wine—PGI	Meteora	Limniona	13.0
19	2017	Dry white wine—PGI	Meteora	Assyrtiko	14.0
20	2018	Dry white wine—PGI	Meteora	Malagouzia	13.0
21	2017	Dry white wine—PGI	Markopoulo, Athens	Savatiano	12.5
22	2018	Dry white wine—PGI	Macedonia	Malagouzia	12.5
23	2017	Dry white wine—PGI	Drama	Assyrtiko	13.5
24	2016	Dry red wine—PGI	Zitsa, Ioannina	Vlahiko	12.0
25	2017	Dry rosé wine—PGI	Korinthos	Agiorgitiko	13.0
26	2017	Semi sweet red wine—PGI	Korinthos	Agiorgitiko	12.0
27	2015	Dry red wine	Korinthos	Syrah/Merlot/Cabernet	14.0
28	2017	Dry red wine—PGI	Kavala	Merlot–Cabernet Sauvignon–Agiorgitiko	14.0
29	2018	Dry white wine—PGI	Kavala	Assyrtiko–Sauvignon Blanc	13.0
30	2017	Dry white wine—PDO	Mantinia, Messinia	Moschofilero	12.0
31	2015	Dry red wine—PGI	Naoussa, Macedonia	Syrah–Xinomavro	12.0
32	2015	Dry red wine—PGI	Naoussa, Macedonia	Syrah	13.0
33	2015	Dry red wine—Table wine	Naoussa, Macedonia	Xinomavro–Mavroudi–Sefka	11.0
34	2016	Dry red wine—PDO	Naoussa, Macedonia	Xinomavro	12.5
35	2015	Dry red wine—PGI	Macedonia	Merlot–Xinomavro	13.0
36	2016	Dry white varietal wine	Trifylia, Messinia	Chardonnay	13.5

PDO: protected designation of origin. PGI: protected geographical indication.

**Table 2:** Grouping of wine samples according to geographical origin.

Group	Samples
Crete	2, 3, 4, 5, 6
Ilia	13, 14, 15, 16
Korinthos	25, 26, 27
Macedonia	7, 8, 22, 31, 32, 33, 34, 35
Meteora	18, 19, 20
Samos Island	9, 10, 11, 12
Others	1, 17, 21, 23, 24, 28, 29, 30, 36

**Table 3:** Significant contribution (*p* < 0.05) of the relative integrals of the ^13^C NMR spectra in relation to the wine variety.

Wine Samples	Wilks’ Lambda	F	df1	df2	*p*
Malvasia di Candia Aromatica–Chardonnay (Other wine varieties)(Crete)	0.999	2.224	7	17576	*0.029*
Syrah–Mandilari (Syrah +Syrah-based wines)	0.999	3.755	7	17576	*0.000*
Syrah–Mandilari (Syrah +Syrah-based wines)(Crete)	0.998	4.562	7	17576	*0.000*
Syrah–Mandilari (Syrah +Syrah-based wines)(Crete)	1.000	0.865	7	17576	*0.533*
Vidiano (Other wine varieties)(Crete)	0.999	2.717	7	17576	*0.008*
Xinomavro (Xinomavro+Xinomavro-based wines)(Epanomi)	0.998	3.930	7	17576	*0.000*
Malagouzia (Epanomi)	0.997	6.429	7	17576	*0.000*
Muscat (Samos Island)	1.000	1.208	7	17576	*0.294*
Muscat (Samos Island)	0.997	7.611	7	17576	*0.000*
Samos red grapes wine (Other wine varieties)	0.998	4.177	7	17576	*0.000*
Muscat (Samos Island)	0.999	2.883	7	17576	*0.005*
Refosko (Other wine varieties)(Letrinoi)	0.999	1.453	7	17576	*0.179*
Daphne Nera–Mavrodafni (Other wine varieties)(Letrinoi)	0.999	3.485	7	17576	*0.001*
Augoustiatis (Other wine varieties)(Ileia)	0.999	3.257	7	17576	*0.002*
Albarinó (Other wine varieties)(Ileia)	0.999	1.311	7	17576	*0.240*
Debina (Zitsa, Ioannina)	0.998	4.105	7	17576	*0.000*
Limniona (Other wine varieties)(Meteora)	0.999	1.282	7	17576	*0.255*
Assyrtiko (Assyrtiko+Assyrtiko-based wines)(Meteora)	0.999	3.335	7	17576	*0.001*
Malagouzia (Meteora)	1.000	0.362	7	17576	*0.925*
Savatiano (Other wine varieties)(Markopoulo)	0.999	3.227	7	17576	*0.002*
Malagouzia (Macedonia)	0.998	3.875	7	17576	*0.000*
Assyrtiko (Assyrtiko+Assyrtiko-based wines)(Drama)	0.999	2.996	7	17576	*0.004*
Vlahiko (Zitsa, Ioannina)	0.999	1.657	7	17576	*0.115*
Agiorgitiko (Korinthos)	0.999	2.218	7	17576	*0.030*
Agiorgitiko (Korinthos)	0.999	2.865	7	17576	*0.005*
Syrah/Merlot/Cabernet Sauvignon (Syrah+Syrah-based wines)(Korinthos)	0.999	3.073	7	17576	*0.003*
Merlot–Cabernet Sauvignon–Agiorgitiko (Other wine varieties)(Kavala)	0.999	3.394	7	17576	*0.001*
Assyrtiko/Sauvignon Blanc (Assyrtiko+Assyrtiko-based wines)(Kavala)	0.999	2.663	7	17576	*0.009*
Moschofilero (Other wine varieties) (Mantinia)	0.998	3.927	7	17576	*0.000*
Syrah–Xinomavro (Syrah+Syrah-based wines)(Naoussa)	0.999	3.536	7	17576	*0.001*
Syrah (Syrah+Syrah-based wines)(Naoussa)	1.000	0.324	7	17576	*0.944*
Xinomavro–Mavroudi–Sefka (Xinomavro+Xinomavro-based wines)(Naoussa)	0.999	1.688	7	17576	*0.107*
Xinomavro(Naoussa)	0.997	7.937	7	17576	*0.000*
Merlot–Xinmavro (Xinomavro+Xinomavro-based wines)(Macedonia)	0.999	2.305	7	17576	*0.024*
Chardonnay (Other wine varieties)(Trifylia)	0.999	3.686	7	17576	*0.001*

Tests of equality of the average values (means) of the grouped wine varieties. F: values of the F-distribution; df: degrees of freedom; *p*: probability.

**Table 4:** Classification of wine samples according to variety based on the relative integrals of the ^13^C NMR spectra and *k*-NN analysis.

Partition	Observed	Predicted								
		Syrah + Syrah-Based Wines	Muscat	Xinomavro + Xinomavro-Based Wines	Assyrtiko + Assyrtiko-Based Wines	Malagouzia	Other wine Varieties	Agiorgitiko	Debina	Percent Correct
**Training**	Syrah+Syrah-based wines	1560	0	0	0	2	0	1	0	99.8%
	Muscat	0	1533	0	0	0	0	1	0	99.9%
	Xinomavro + Xinmavro-based wines	0	0	1515	0	1	0	1	0	99.9%
	Assyrtiko + Assyrtiko-based wines	0	0	0	1539	1	0	1	0	99.9%
	Malagouzia	0	0	0	0	1524	0	4	0	99.7%
	Other wine varieties	0	0	0	0	1	1543	5	0	99.6%
	Agiorgitiko	0	0	0	0	2	0	1577	0	99.9%
	Debina	0	0	0	0	2	0	0	1523	99.9%
	**Overall Percent**	12.6%	12.4%	12.3%	12.5%	12.4%	12.5%	12.9%	12.3%	99.8%
**Holdout**	Syrah+Syrah-based wines	634	0	0	0	0	0	1	0	99.8%
	Muscat	0	664	0	0	0	0	0	0	100.0%
	Xinomavro + Xinmavro-based wines	0	0	680	0	1	0	0	0	99.9%
	Assyrtiko + Assyrtiko-based wines	0	0	0	656	1	0	0	0	99.8%
	Malagouzia	0	0	0	0	670	0	0	0	100.0%
	Other wine varieties	0	0	0	0	0	648	1	0	99.8%
	Agiorgitiko	0	0	0	0	0	0	619	0	100.0%
	Debina	0	0	0	0	0	0	0	673	100.0%
	**Missing**	0	0	0	0	0	0	0	0	
	**Overall Percent**	12.1%	12.7%	13.0%	12.5%	12.8%	12.3%	11.8%	12.8%	99.9%

**Table 5:** Cross–validation parameters for the partial least squares-discriminant analysis (PLS-DA).

Measure	Component 1	Component 2	Component 3
Accuracy	0.15833	0.15	0.225
R^2^	0.62002	0.89925	0.97146
Q2	−0.37807	−0.1962	−0.16277

## Supplementary Materials

The following are available online at https://www.mdpi.com/2304-8158/9/8/1040/s1, STEX: Information on wine samples, Figure S1: ^13^C–^1^H NMR spectra recorded during the analysis of wine samples of different varieties, Table S1: ^13^C NMR integrals (mean values) according to the variety of wine samples.
