# Supplementary material for: 13C NMR-Based Chemical Fingerprint for the Varietal and Geographical Discrimination of Wines
Source: Foods. 2020 Aug 2;9(8):1040. doi: 10.3390/foods9081040 (PMC7466255; doi:10.3390/foods9081040)
Supplement: Supplementary file 1 [file foods-09-01040-s001.pdf]

SUPPLEMENTARY MATERIAL FOR ONLINE REVIEW

Supplementary text (STEX):

Information on wine samples

Syrah and Syrah based wines

The Syrah wines may be rather oaked or unoaked. This was the case for the samples obtained from Crete. For example, the red dry wine PRINOS was unoaked. On the other hand, the Syrah-Mandilari (70%-30%) wine included 12 months of aging in oak barrels, giving an exuberant wine with a strong bouquet of raspberries and pepper to dominate. It is a wine with a pronounced sharpness, pleasant duration and great aging potential. Rosé wines are also prepared (i.e., PRINOS rosé). The colour of this wine variety is reminiscent of pomegranate seeds. The aroma refers to ripe red fruits, mainly cherry. The consumer may also distinguish a cool and balanced taste, with delicate tannins, pleasant acidity and a long aftertaste.

Muscat

The small-berried Muscats, perched on the highest mountains of Samos Island give a very powerful wine, which conveys perfectly and in a balanced manner the characteristics of the variety. A similar case comprises the white Muscat, VIN DOUX (VIN DE LIQUEUR) which shows the classic expression of small-berried Muscat of Samos Island with distinct aromas of flowers and fruit. The white Samos Muscat is vinified after it has been exposed to the Aegean sun and gives us a unique sweet wine, which after 6 years of ageing, obtains exceptional complexity and balance. The ancient technique of drying the grapes in the sun before fermentation is still used in Samos Island.

Xinomavro and Xinomavro based wines

Xinomavro is a black grape variety with sour taste. Xinomavro, a charismatic and demanding grape variety of Macedonia, which was ideally acclimated to the microclimate of Epanomi, gives a rosé wine with a strong aroma and acidity. It may be combined, harmoniously, with the summer Mediterranean flavours and exotic cuisines. It may be characterized as a delicious Greek variety that transcends the gastronomic borders (Ktima Gerovassiliou, Epanomi, Macedonia).

Another example includes the red dry wine that retains the traditional aspects of the noble Xinomavro variety, which grows in the charismatic wine growing area of Naoussa. The rich aroma, the distinct bouquet and the deep red color are the trademarks of this wine that shares the name that produced it. The first sense of aroma is that of wood of the barrel. Intense tannins are seen in the palate, but these blend harmoniously. It may be characterized as a warm and mouth-full wine with balanced acidity and persistent taste of red fruit and spices. It remains 12 months in barrel and 6 months in bottle.

A wine that revolutionizes the palate is the excellent blending of the Xinomavro and Syrah varieties, which result in the production of soft tannins and fruity aromas that perfectly complement the European cuisine. The mixing of the Xinomavro and Syrah varieties gives a pleasant aroma of black fruit, which is delicious with soft tannins and fruity aromatic flavour This blend of wine varieties remains 6 months in barrel and 3 months in bottle. Vivid and velvet purple colour are the chromatic notes that accompany these wines (VAENI Naoussa, Macedonian PGI wines). Finally, the blend of Xinomavro, Mavroudi, and Sefka varieties gives a pleasant red wine which brings a sense of vivid flavour. It keeps the freshness and characteristic taste till its last drop. Its aroma resembles the bright red fruit. It has a taste of mature fruit with a light balanced body, however, well structured. It has been characterized as a pleasant wine that expresses the specificity of the grapes of the Naoussa region

Assyrtiko and Assyrtiko based wines

For example, the wine ''Idisma Drios'' from Drama expresses the sweetness that the oak barrels bestow on the character of the wine. Fermentation and maturation takes place in oak barrels for 6 months. On the other hand, the Assyrtiko wine from Meteora is produced from grapes grown in selected vineyards of the Estate around Theopetra Cave, near Meteora Rocks. Finally, the Assyrtiko variety may be well combined with Sauvignon Blanc variety to give a fruity wine indicating the unique characteristics of Paggaio region (Kavala, Macedonia).

Malagouzia

Malagouzia variety from Meteora originates from grapes grown in selected vineyards of the Estate around Theopetra Cave near Meteora Rocks. The soil is rich of calcareous clays, the subsoil is slate. The mesoclimate is continental with the influence of Pindos Mountains and Pineios River. On the other hand, the Malagouzia variety cultivated in Epanomi region (KTIMA Gerovassiliou), was rescued from extinction thanks to the efforts of Evangelos Gerovassiliou, who recognized its potential and vinified it for the first time in the 1970s. Grapes from the Estate's first vineyards give a rich wine with fruity aromas that balance the crispy acidity.

Other varieties

Limniona

Limniona variety comes from grapes grown on the Estate's sloping vineyards around the cave of Theopetra, near Meteora Rocks. The fermentation is carried out in French oak tank, the aging lasts for 12 months in French and American oak barrels and 12 months in bottles. Some tasting notes are the deep ruby colour with purple highlights, aromas of cherry, raspberry and dried fruits and notes of nutmeg and black pepper . On the palate, silky tannins, red fruit flavours and complex aftertaste may be observed.

Savatiano

A fine dry white wine carefully produced with Savatiano grapes at the Papagiannakos domaine. It is clear in colour and has the delicate bouquet of this fine grape from Attica, Athens. It comprised a wine to be indulged in.

Vlahiko

A red wine which is maturated for 12 months in French oak barrels. It is a wine that possesses soft tannins and high acidity, a strong bouquet, with sharp rubin-like colour and notes of red fruits, dried plums and pepper. The velvety round flavour with vanilla notes are some additional sensory characteristics. It is a product of low-yield crop cultivation in Zitsa (Ioannina).

Refosco, Augoustiatis, Daphne Nera, and Albariño

Refosco variety comprises an evidence of vinification of the clone of the Refosco variety, brought from Northern Italy by Theodoros Mercouris in 1870 and is still preserved today in the winery estate in Korakochori Ileias (Peloponnese).

Augoustiatis variety is an evidence of local red wine variety of the Ionian Islands by grapes from the vineyards of the Merkouri estate in Korakochori, Ileias. Similarly, the Daphne Nera variety is an evidence of vinification of the Mavrodafni variety, Tsigelo clone, from grapes cultivated in the vineyards of the Merkouri Estate in Korakochori, Ileias. Finally, Albariño grape variety is a proof of vinification of the Spanish Albariño variety (Rías Baixas, Galicia, Spain) imported into the Greek vineyards of Merkouri Estate in 2009.

Merlot/Cabernet Sauvignon/Agiorgitiko

After 12 months of aging in oak barrels, it gives an aromatic wine with a rich body that highlights the unique characteristics of the area (Ktima Biblia Chora, Paggaio, Kavala).

Semi dry rosé table wine: Selana

Selana (or Selini, the moon in Greek) was the Greek goddess of the lunar soul. Selana is balancing between the soft spices and the summer fruit, in the same way that the moon brings balance from day to night. A rosé wine, from red varieties of the Samian vineyards, which enchants the palate, as enchanting as the moon is.

Vidiano and blends

The grapes of the Vidiano variety create a wine with a distinctive golden color and a complex bouquet of citrus aromas such as bergamot, as well as scents of pear, banana, and melon. It is a tasty, and somewhat, oily wine with unique acidity, which makes it an excellent accompaniment for a wide range of dishes and foods. The Vidiano and Assyrtiko varieties were blended harmoniously and gave this particular wine. Separate vinification of each variety, completion of alcoholic fermentation in oak barrels, and aging for 3 months. The colour may be gold-yellow. Aromas of fresh and dried fruits, citrus fruits, vanilla and nuts characterize this wine. It is a very rich wine in the mouth-feel, with crispy acidity and a long aftertaste. Finally, this wine blend responds well to further aging.

Malvasia di Candia Aromatica-Chardonnay

Malvasia di Candia Aromatica (50%)-Chardonnay (50%) (PRINOS blanc) is a wine that has a shiny yellow colour with greenish reflections. A rich aromatic bouquet with dominant aromas of peach, pineapple, banana and lemon, characterize it. In the mouth, a balanced acidity and a long lasting fruity aftertaste is observed.

Dry white varietal wine-Chardonnay

From small, selected vineyards in the semi-mountainous region of Trifyllia, Chardonnay wine has a bright yellow-green color. This type of wine is intense in the nose and in the mouth, possessing aromas of white flesh fruit reminiscent of fresh peach, mango and pineapple. Rich and balanced mouth-feel, with great acidity and vibrant sense of oiliness, due to aging on fine lees in oak barrels, is the main potential of this wine.

Moschofilero

The dry white wine Moschofilero from Mantinia is produced from Moschofilero variety grown in vineyards at Zevgolatio in the Mantinia region, at an altitude of 650 m. The colour of this wine is bright to yellow-green. Numerous elegant floral aromas, such as lemon, citrus and rose petals, typical of the Moschofilero variety, are developed. This wine is rich and fruity on the palate, with a long aromatic aftertaste.

Agiorgitiko

The dry rosé wine, Agiorgitiko from Nemea, is a bright to cherry-red colored wine. There is a good intensity in the nose with aromas of ripe, sweet fruit, strawberry, cherry and milk chocolate. On the palate, it displays just a hint of sweetness and a noted acidity, which gives freshness and concentrated cherry flavor. Similarly, the Piccolo Mondo red wine, is a semi-sweet red wine of the Agiorgitiko variety from Nemea (Korinthos). This type of wine has a bright, transparent ruby red color. The intense aromas of red fruit in the nose and silky tannins on the palate are followed by a sweet and fruity aftertaste.

Debina

The dry white wine from the Debina variety is cultivated in the wine-growing zone of Zitsa, 700 m above sea level, in Ioannina. The colour is green to yellow. This type of wine has citrus notes, balanced with a long aftertaste (ZITSA CLASSICO). The white wine demi sec-Debina, is a product of secondary fermentation in base wine, which is carried out in small autoclaves (cuve close) under controlled conditions. It has a bright green colour with yellow notes, and distinctive citrus aromas.

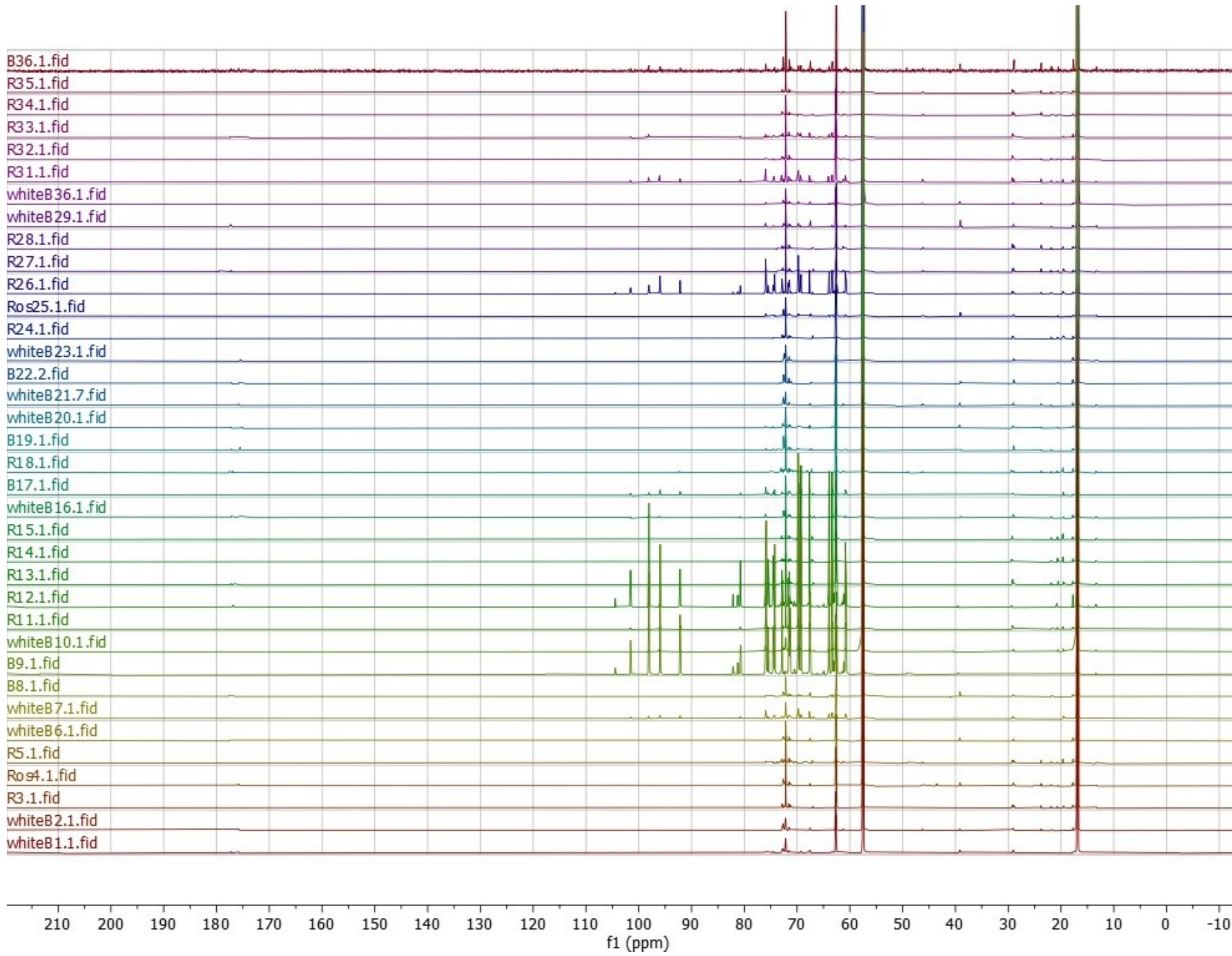
B36.1.fid
R35.1.fid
R34.1.fid
R33.1.fid
R32.1.fid
R31.1.fid
whiteB36.1.fid
whiteB29.1.fid
R28.1.fid
R27.1.fid
R26.1.fid
Ros25.1.fid
R24.1.fid
whiteB23.1.fid
B22.2.fid
whiteB21.7.fid
whiteB20.1.fid
B19.1.fid
R18.1.fid
B17.1.fid
whiteB16.1.fid
R15.1.fid
R14.1.fid
R13.1.fid
R12.1.fid
R11.1.fid
whiteB10.1.fid
B9.1.fid
B8.1.fid
whiteB7.1.fid
whiteB6.1.fid
R5.1.fid
Ros4.1.fid
R3.1.fid
whiteB2.1.fid
whiteB1.1.fid
210 200 190 180 170 160 150 140 130 120 110 100 90 80 70 60 50 40 30 20 10 0 -10
f1 (ppm)

138

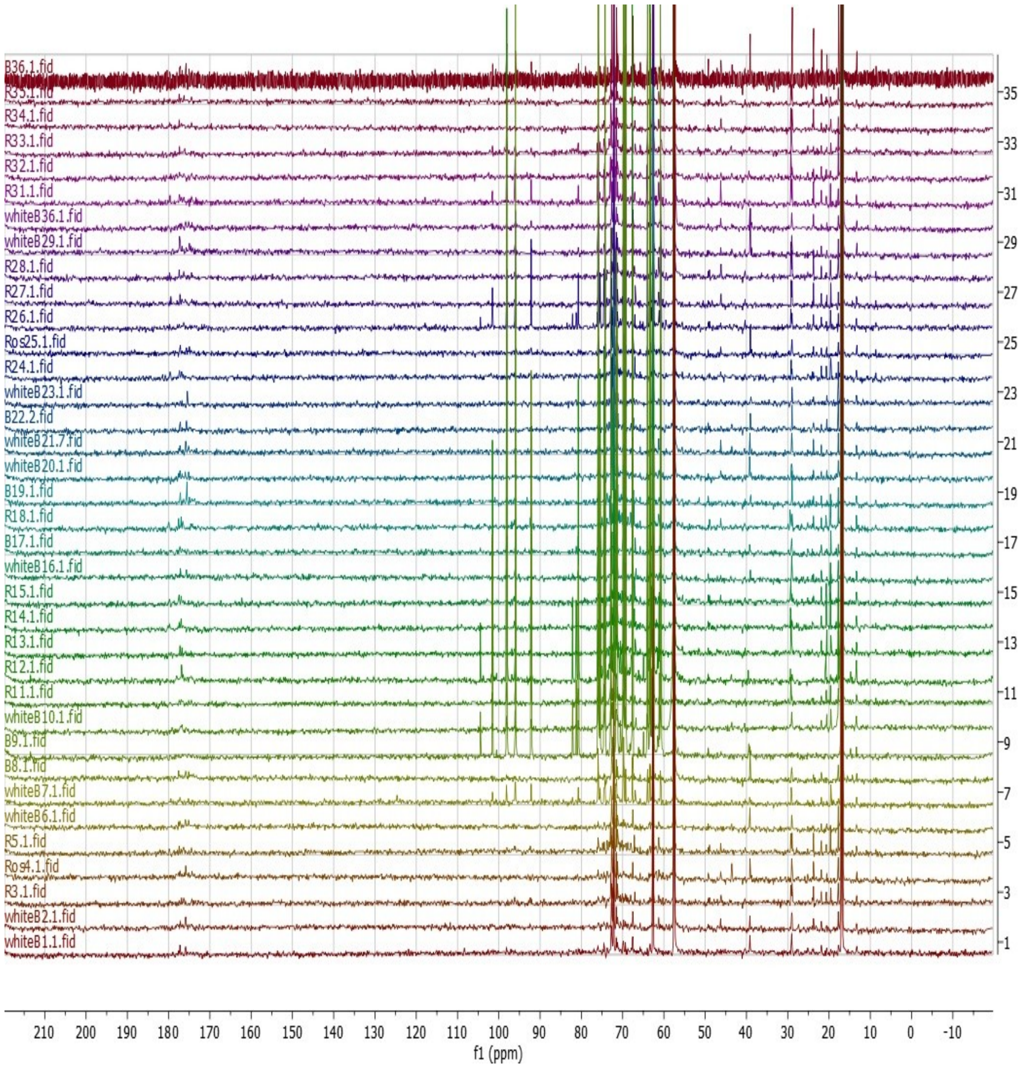

Supplementary Figure 1. ^{13}C-^{1}H NMR spectra recorded during the analysis of wine samples of different varieties.

Supplementary Table 1. ^{13}C NMR integrals (values) according to wine sample variety.

	S5	S6	S7	S8	S9	S10	S11	S12	S13	
	2017	2017	2018	2018	2017	2017	2018	2011	2016	
	Crete	Crete	Epanomi, Macedonia	Epanomi, Macedonia	Samos Island	Samos Island	Samos Island	Samos Island	Letrinoi, Ileia	L
	Syrah	Vidiano	Xinomavro	Malagouzia	Muscat	Muscat	Samos red grapes	Muscat	Refosco	Ne
	12.5	13.0	13.5	13.5	15.0	12.5	12.5	14.0	14.0	
ne	Dry red wine -PGI	Dry white wine-PGI	Dry red wine-PGI	Dry white wine-PGI	Dry white wine-PDO	Dry white wine	Semi dry rosé wine	Nectar, white wine naturally sweet -PDO-6 years aged	Dry red wine-PGI	Dry
	-6535.92	-10534	-11304.5	-9789.85	-18602.3	5280.15	-4083.41	-17067.1	-2453.77	
	-6180.73	-5052.4	-6027.12	-12326.9	-19504.4	368.854	-1197.79	-11481.9	1655.87	
	-783.869	-8238.28	-12552	-11461.6	-12357.9	-4646.28	-2536.88	-15233.7	-3123.25	
	-5257.11	-13386.5	-15532.5	-19045.1	-16027.1	-6403.17	-7084.34	-15031.6	-11799.5	
	-2200.24	-12443.9	2223.44	-6505.77	-10297.4	-1163.7	1340.19	-19031.9	6178.87	
	-4968.95	-1642.46	-4074.96	-5858.11	-8365.73	1639.97	1126.59	-17204.5	-143.102	
	-5145.74	-7198.48	-6535.17	-12032.6	-14673.4	-3218.26	-7293.06	-13354.3	-7706.22	
	-3074.8	-4757.1	-9517.74	-12091.8	-8771.53	3023.61	1122.18	-16319.8	644.494	
	-1753.97	-1026.08	-1920.26	-15348.2	-13984.5	1193.6	-290.619	-10989.6	105.826	
	-4455.34	-7990.18	-2710.69	-6923.96	-14171.1	476.52	-2896.87	-10748.5	1104.44	
	-8939.52	-6676.28	-7136	-6296.21	-13296	-631.756	-988.398	-23025.4	4575.35	
	-3726.93	-767.949	-7811.83	-7133.93	-16541.4	964.258	3421.01	-15213.3	750.748	
	2965.9	-5412.03	-3946.84	-14711.6	-13943.4	391.002	6112.12	-14581.1	-5559.54	
	-8917.44	-14120	-11494.7	-7052.38	-13926.6	-2849.88	1980.88	-16809.8	-3887.26	
	-6844.44	-9348.8	-9262.16	-9357.04	-9919.67	-1740.09	2122.95	-8116.77	-2931.15	
	130.818	-12021.4	-1484.2	-15424.2	-13369.6	-3674.08	-2345.77	-14874.4	-670.703	
	423	-8948.38	-9433.71	-18306.7	-9100.9	6907.97	-2172.89	-21195.3	-1727.53	
	-4869.28	-12228	-12630.1	-11526.1	-18058.4	6654.22	-7779.62	-14919.4	-3889.64	
	-8514.04	-11456.3	-11267.5	-9099.1	-22138.1	-5139.78	-9.74219	-14897.6	719.758	
	-3007.38	-10934.9	-5958.62	-14372.2	-12145.9	2050.44	939.535	-15094.4	-819.775	
	-7495.33	-3967.44	-6032.06	-6697.98	-11750.3	-1264.37	4400.9	-13629	-3068.39	
	4051.67	-4973.68	-11216.7	-14121.6	-11845.5	1613.52	-1314.47	-16526.1	-3474.11	

-3189.05	-7280.67	-8161.79	-13327	-11188.8	2720.19	6195.22	-11200.6	1299.27
4025.54	-5042.76	-4736.08	-7546.9	-12069.5	-3104.89	1857.67	-6954.97	7886.99
2601.33	-7885.11	-4208.89	-9951.19	-8543.06	-4842.71	6859.14	-11358.6	-3075.14
-8505.73	-9016.59	-5527.77	-7914.03	-13059.2	2518.35	6814.81	-11690.9	-3669.42
-5056.63	-6646.47	-12297.8	-14573.1	-12086.6	416.748	-3672.65	-15149.2	-86.5859
-6779.3	-8621.92	-8166.37	-2625.77	-14641.5	-2162.5	3736.77	-11172.7	-2526.82
670.336	-3341.94	-10905	-11514.9	-12924.5	2494.59	-2218.89	-17468.5	-3841.15
-2784.6	-7891.49	-12969.5	-9840.78	-11211.2	-1597.64	557.328	-17085.4	3177.53
-10119.7	-7058.59	-11934.3	-11292.7	-13754.1	-2688.83	2524.37	-13950.4	7762.61
1131.59	-7825.66	-14068.9	-13327.2	-6286.51	213.646	3403.59	-12704.5	2989.96
-8435.28	-10799.6	-5870.61	-12354.1	-7417.41	-165.316	-2565.82	-8611.94	-2662.61
915.57	-10593.7	-14575	-13827.1	-19639.5	-225.262	-456.762	-15705.8	-4448.27
-5983.24	-9763.69	-9641.6	-11742.6	-13263.9	-1016.66	1048.03	-14125.2	-1698.89
-333.242	-15453.1	-4088.79	-8003.3	-12443.7	-6930.58	1971.95	-9243.59	538.725
-3811.71	-14081.1	-8084.62	-11199.1	-11995.9	-4399.29	-29.3594	-14344.2	-2729.02
-2871.05	-8452.97	-12453.6	-5764.37	-10352.6	-6725.79	2650.54	-10777.2	579.988
-2599.96	-15526.2	-10071.8	-9033.61	-3960.77	7907.58	383.154	-10347	-3638.26
-4657.68	-12447.8	-4604.37	-9208.59	-9557.75	297.379	7435.7	-5565.65	-9759.04
-2497.36	-11721.9	-4596.73	-13895.5	-6261.15	-6247.26	-1973.88	-13218.9	-7300.44
1200.25	-16700.1	-7945.86	-6731.85	-7658.73	-346.561	1286.65	-16983.3	6628.34
-4741.41	-14722.6	-2848.51	-9737.59	-9836.4	-2527.74	367.832	-17637.2	-9133.03
-6201.14	-6028.83	-9788.89	-11593	-11728.8	-3804.47	5663.93	-12763.4	-4589.77
-4159.83	-6725.62	-2817.16	-4685.59	-10826.7	-4628.49	335.146	-13481.8	2073.11
-4606.32	-11114.8	-4994.34	-7892.56	-12584.9	193.781	-4207.32	-12616.4	-176.859
-6912.01	-16160.3	-4753.46	-7642.94	-10550.2	-26.9609	1507.94	-16223.1	109.699
-1352.11	-8950.33	-10754	-13307.8	-12657.6	2547.78	-3848.68	-10823.2	-3538.34
-1531.87	-2628.96	-11947.1	-4261.23	-10564.1	7845.29	3029.63	-10804.4	-6406.9
-12059.4	-16688.7	-1032.45	-13407.6	-8193.26	909.285	-505.357	-15472.9	3417.91
-1340.62	-5945.15	-18097.2	-6791.9	-9887.92	-7820.46	-184.564	-14835.6	-1176.65
-259.115	-10942	-4426.57	-14660.1	-18449.5	171.518	-4263.91	-7781.29	-8351.56
-4890.78	-6863.19	-4289.29	-13529.3	-13034.2	476.432	-3750.35	-8980.6	-4290.24
-5540.43	-6317.14	-4328.7	-10412.2	-12006.8	57.5625	-2249.85	-14555	-6400.47
-5925.58	-15224.2	-3663.6	-8512.94	-13671.2	-5723.89	-568.656	-17401.4	-1079.29

-4519.87	-6641.19	-4990.66	-3768.72	-10666.2	4086.71	-563.807	-16308.3	-4484.65
-6420.9	-8290.8	-8361.7	-6475.35	-6808.92	-913.82	-4487.52	-12175	-1616.19
-4395.95	-8299.89	-6112.39	-11288	-263.24	-1697.55	-1386.11	-12135.5	2982.62
-2682.18	-4215.34	-12165.3	-4700.15	-11665.4	7240.32	7101.25	-18436.1	557.299
-2110.54	-11128.1	-4872.78	-7097.53	-10116.4	6812.3	7092.32	-14188.5	-2410.53
-4106.18	-7260.93	-8547.25	-10239.9	-16468.5	3333.23	-6145.66	-18127.6	-2147.46
-4330.5	-5911.99	-3453.49	-10722.2	-4708.07	1006.21	-50.1191	-13264.1	-5853.12
-1990.74	-8791.87	-5290.71	-1774.55	-13345.3	3906.7	3984.31	-14893.9	509.084
-7094.4	-9500.52	-1176.82	-4273.78	-8786.57	5777.17	-3749.24	-15709.5	-3380.93
286.393	-7095.98	-5091.31	-7836.88	-15045.4	-3322.44	834.563	-19853.8	-2726.11
-6302.82	-10158.4	-7311.14	-12331.7	-9872.69	4381.33	1541.42	-11138.2	329.193
-9958.14	-8891.66	-13960.2	-15166.4	-9451.84	-3076.39	-1531.71	-9358.92	580.611
-6921.56	-9399.53	-5617.24	-3808.08	-14846.1	-3274.28	-3614.42	-16295.5	-5138.19
-4146.73	-7893.25	-7089.4	-9256	-10454.8	-1536.49	32.9219	-14205.1	-7765.54
-4840.49	-1812.92	-14083.1	-12060.5	-12542.8	-3486.8	6969.93	-14801.5	-2066.21
-6976.88	-10453.8	-9416.03	-14368.5	-10384.4	-6208.24	-5275.41	-13876	-2290.86
-13708	-9116.09	-6844.75	-10317.5	-17806.8	-4984.37	-183.205	-15451.4	-1862.42
-2658.62	-13198.1	-9559.36	-7897.5	-12851.2	-1555.1	-993.928	-18689.2	-5108.93
-2176.86	-15330	-9958.29	-10669.4	-13758.3	-6075.79	-5973.8	-19689	-2533.25
-10005	-10526.4	-13000	-14317.2	-15043.9	-913.791	-2213.83	-10181.4	-4707.85
-6258.99	-18875.2	-16499.2	-5889.62	-15922.7	769.496	1746.98	-13650.6	-5099.66
1360.27	-10792.5	-12844.9	-11004.7	-17008.5	497.408	-2286.86	-16206.2	-5194.39
2337.5	-13648.6	-11069.6	-8490.35	-13653.7	-2047.99	3166.3	-19095.1	-3658.39
-126.398	-7973.79	-7690.25	-6548.09	-8508.11	2106.91	893.937	-15013.2	-6359.62
-3794.42	-7133.26	-10159.3	-9088.77	-13049.2	-2645.88	-8838.85	-18298.3	-5012.81
-9734.1	-7841.43	-4855.29	-14368.1	-20772.8	4718.72	-4665.64	-22274.2	-6313.76
-3895.63	-2321.46	-6545.7	-10344	-17779.6	5333.22	-445.363	-16651.2	-2278.34
2681.44	-10166	-4984.75	-13627.2	-14382.3	-376.93	4445.79	-11904.6	66.7422
-10496.2	-10388.2	-7073.23	-2911.76	-18850.4	-3317	-5051.74	-11664.3	-5812.44
-1905.83	-9309.37	-10542.4	-7527.31	-17121.8	-584.418	-5818.48	-13772.9	-4373.04
-3539.11	-16741.7	-11369.4	-15840	-14973.2	-363.15	-5888.36	-13019.6	77.7754
8027.77	-5534.66	732.395	-406.502	-13469.6	3789.4	12621.7	-6251.6	8753.79
-4136.11	-6687.86	-6929.5	-7783.49	-3053.37	4181.36	-1800.79	-3891.48	6178.35

-2408.44	-7595.27	-10569.9	-11630.7	-11029.2	399.223	-5876.4	-17471.2	-612.561
-2807.27	-12443.8	-13670.6	-7069.33	-14256.3	2725.92	-260.777	-10814.7	-4258.94
-1862.28	-9901.39	-2144.7	-3895.52	-13803.1	2905.53	-6536.67	-9600.85	-3177.79
-4589.92	-8327.01	-3204.78	-6307.17	-15600.3	-2381.81	-260.654	-11434.8	-1386.22
-3073.11	-10858.2	-6049.26	-3016.15	-13786.6	-4213.72	-3629.99	-14234.2	-1989.98
-4510.48	-6835.16	-4400.81	-14012.3	-19890	-6133.75	-101.215	-5670.16	-2016.34
-3817.21	-8575.86	-10832.3	-9151.99	-13715	-532.518	-2234.77	-14672.5	1776.03
-8834.73	-4682.35	-11845.3	-13265.3	-10013	431.141	4610.14	-17848	-1755.48
-3756.7	-6073.66	-8183.03	-10815.1	-14236.3	-737.986	4564.08	-10784.9	-839.699
-4549.58	-7967.14	-12097.4	-15356.6	-14786.3	-1321.1	2506.18	-11229.8	354.996
2970.85	-2525.4	-2622.54	-4466.91	-13112.2	-3327	2176.98	-13858.6	-4236.66
343.746	-12632.7	-6975.9	-7644.77	-10002.9	-1724.35	5859.26	-12868.7	-1051.41
-3091.79	-8090.86	-8010.8	-6551.51	-5210.52	797.26	5193.86	-16135.1	516.035
-4301.32	-9553.71	-4291.18	-10103.3	-11864.3	-2097.43	-4081.24	-17242.9	-5974.14
980.584	-16250.4	-4734.87	-11687.6	-9872.74	-2728.59	5381.47	-14551.4	-3000.91
-5008.98	-13173.5	-7828.92	-8288.52	-9393.73	-2146.93	-3765.08	-13447.4	-2447.23
-10404.6	-14630.1	-2284.58	-12909.7	-10691	-2777.74	-8295.5	-20146.6	-11355.3
1540.67	-9306.82	-2637.84	-6176.49	-12510.6	-273.625	1524.46	-12005.5	-1521.42
122.387	-10384.2	-14790.7	-8526.33	-15360.8	5910.05	-990.684	-9579.52	-5835.72
-761.168	-8223.56	-6456.97	-11311	-8689.5	-1608.52	3652.71	-4405.38	1598.63
-5658.11	-8096.46	-4944.82	-8559	-7161.58	691.41	2949.74	-6114.98	1456.09
6365.58	-6014.68	-3416.39	-11434	-12985.3	-5920.73	-6026.57	-12323.9	-884.766
-5756.76	-5574.23	-7799.91	-5385.76	-13059.3	4781.33	5839.77	-8613.14	354.061
-6277.4	-6612.66	-9386.55	-7566.78	-12266.2	-2810.88	3173.13	-14923.1	-2920.95
-7356.46	-7977.93	-10590.2	-4601.01	-16026.1	932.924	7418.28	-13539.6	1395.57
-3387.65	-9361.36	-1155.6	-10679.7	-12806.5	-656.303	5354.35	-9877.57	-7670.94
-2228.1	-9461.59	-8975.33	-8035.65	-14066.1	-3613.16	-2713.27	-20694.6	-719.021
-4030.51	-9497.39	-4900.11	-18700.9	-17832	-537.285	5277.74	-7268.01	-5091.36
-169.824	-6863.77	-11785.6	-6632.22	-18262.4	2968.55	3561.02	-14209.5	-142.342
-2266.55	-9843.33	-2365.32	-7671.88	-8656.94	6342.58	119.051	-12115.9	271.742
-575.605	-11226.8	-6579.25	-11926.9	-13725.2	2501.26	1096.22	-10621.6	-2003.89
-3464.31	-6247.78	-4321.63	-3849.35	-5523.26	4595.49	-3029.21	-8035.51	664.002
-8004.95	-15086.1	-12164.8	-12156.5	-8258.25	-1835	-3518.9	-18131.7	-7869.37

-5950.16	-14110.1	-11090.5	-10210.9	-15107.7	-4757.6	-3667.93	-18900.3	-4364.43
-4487.56	-4625.44	-1814.71	-9734.17	-14561.7	3536.01	-501.865	-22143.5	-538.785
-978.789	-2773.52	-4581.22	-9309.46	-12180.1	-617.332	1850.26	-10973	-3406.38
-880.295	-9347.4	-13691.1	-5348.58	-11939.1	-4043.41	473.455	-17397.7	1734.77
1704.86	-9130.3	-8251.32	-7176.86	-14204.5	-7160.86	-3713.86	-15215.3	453.225
-8264.76	-13088.6	-9074.05	-6964.1	-15543.8	-4634.11	-3307.65	-17080.1	-4048.68
-4640.17	-6600.86	-10042.4	-8767.5	-11104.3	-5303.47	68.5723	-19133.3	-6953.97
-2280.94	-4423.77	-10195	-6144.32	-14586.8	-408.557	-3490.91	-13593.2	71.6875
-6308.5	-11769.4	-7375.1	-3834.69	-9035.96	481.199	547.535	-17053.4	-3149.4
-2620.35	-6730.93	-3778.47	-12242	-3624.15	-2464.63	5203.25	-10412.1	-2569.98
3672.73	54.7168	-4465.16	-8969.72	-13749.9	-31.7578	4623.89	-12849	2113.26
8933.1	4453.5	12921.7	11025.6	-12925.9	4643.26	15421	-1782.99	21904.9
17396.3	7973.38	14165.4	7911.5	15013.9	30884.4	20998.2	46336.8	27522.7
-6284.69	-1251.94	-477.971	-9077.88	-11422.5	15316	-105.447	-2406.14	-2489.12
2761.1	-3803.16	-6857.84	-5988.97	-2736.66	5954.14	2016.87	-3926.31	9914.76
-3047.13	-10528	-7464.68	-10629	-10192.7	431.961	855.508	-4542.27	-363.254
-4955.91	-11089.3	-13212.2	-6925.54	-13934.2	2237.45	714.975	-10858.4	-7761.36
-12058.4	-11559.9	-7062.76	-10327.7	-9267.15	-8641.28	514.232	-14397.5	4848.8
-6741.77	-13013.3	-11449.7	-15541.6	-11825	-362.389	-284.102	-9344.65	177.076
-8844.46	-10051.3	-5571.77	-8364.11	-16141.4	2296.76	1311.98	-16100.7	1002.23
1366.84	-7509.52	-7478.38	-5983.08	-12480.7	3353.01	-2804.59	-13827.5	4499.29
-1480.35	-4221.29	-1065.19	-6737.52	-14696.9	-1430.11	6722.78	-10130.5	893.422
5310.98	-10374.1	-2299.04	7417.33	-4987.87	2694.73	4151.6	1328.22	11023.2
3993.6	-12806.2	-5285.03	-6714.86	-16082	7416.77	2932.22	-15753	-3872.9
-5760.09	-8067.71	-13819.3	-4535.32	-14297	1257.7	-887.07	-10172.3	-1133.52
330.93	-6464.13	-14115.2	-6730.26	-10291.8	-3436.75	-6038.34	-12101.3	638.684
-10897.1	-12307.1	-9043.4	-11389.9	10463.7	1954.51	1685.85	16258.8	-1313.62
-4611.13	-9458.57	-9270.49	-4074.52	2171.7	-417.334	7030.11	2397.8	-724.764
814.264	-651.943	-667.939	-4740.89	-8282.69	1366.42	4315.88	-9781.11	2533.71
-2810.2	-2938.1	-4523.86	-6732.59	-10547.4	4273.37	-4055.35	-14205.8	836.697
-235.643	-11992.8	-5484.39	-11227.7	-9835.54	4043.97	2336.7	-8418.57	257.596
-5634.97	-5138.9	-9076.95	-7347.11	-12495.4	-4661.58	6450.59	-3653.44	2324.83
892.684	-5407.02	-9782.22	-10427.2	-10513.7	-1243.6	637.898	-6959.67	-533.943

-5350.45	-5637.86	-2792.19	-7853.55	-6784.42	2112.82	887.48	-13916.3	-341.982
-2960.74	-10622.7	-5554.12	4718.45	-14226.7	2473.55	6767.58	-15176	5515.65
-7548.17	-8237.63	-3914.12	-8469.42	-16961.3	3121.27	2388.23	-5252.76	-3662.3
-3038.79	-8206.42	-4842.62	-12759	-10898	4619.01	939.258	-7190.11	-2741.09
-10513.2	-8852.95	-13584	-5286.79	-10716.5	3080.02	2065.03	-11426.3	562.521
-4397.31	-10696	-7660.94	-5255.78	-9287.78	-5348.5	1220.51	-10396.3	3576.29
-776.766	-9234.11	-11995.3	-3098.76	-8429.76	1592.2	4247.52	-6822.21	3529.07
-4945.38	-3025.02	-5355.77	-2825.7	-5394.11	8232.98	10364.7	-4647.09	5108.94
-6246.85	-9182.76	-10402.5	-8121.01	-4577.91	4743.85	10866.2	-1812.64	10461.2
-3790.12	-4474.03	-5724.41	-2535.37	-5816.58	15882.1	9954.43	-4753.12	11290
5044.07	-7774	7289.23	17271.3	3065.68	15069.5	16374.8	-5515.71	19957.1
9448.44	-1446.95	5354.63	13239	-1517.67	31854.5	32527.8	1440.33	30946.4
16306	4199.96	37708.3	33125.2	14024.2	46729.8	39095.9	11959.2	50515.4
134605	126613	343914	173124	106493	152734	168209	96982	194288
8.27E+06	7.49E+06	6.01E+06	9.44E+06	335661	1.82E+06	8.27E+06	333806	9.26E+06
1.63E+06	3.26E+06	4.20E+06	1.10E+06	1.28E+07	7.83E+06	2.00E+06	1.20E+07	2.56E+06
139311	154685	170121	106953	312728	968380	122857	328560	133904
32477.5	31555	41707.9	7778.24	53428.1	465448	18109.7	51234.9	15934.3
12012.7	19462.3	14859.9	3253.94	23594.7	302496	6651.08	5391.01	2403.11
12202.4	2200.43	7678.48	-1934.59	3738.39	230862	1322.67	-222.174	-7018.41
6163.44	5116.42	-4410.72	-3169.98	-3749.82	170666	2559.53	914.262	-1327.46
4131.23	2464.52	-2821.92	-10946.1	-4244.1	121550	424.211	-3145.48	-5073.61
12664.5	8174.01	9499.6	2426.51	-7609.31	92367.9	6082.68	-5265.1	13462.6
72878.9	57791	10059.2	34237.8	-3251.48	83953.7	38146.7	211438	58487
1500.55	-2316.91	-4188.75	-9053.56	-10022.1	32905.2	-2602.99	-7609.84	-9156.21
-113.891	-3298.09	-6589.7	-7895	-13945.9	26753.9	1695.02	-14548.5	-2297.09
970.855	-5159.04	3171.14	-5518.62	-11627.1	21145	7695.54	-14335.1	252.408
1424.42	-5910.48	2530.74	-8536.17	-13913.7	5790.07	-2431.16	6199.21	-5055.8
11456.7	-1513.71	-5644.84	-2499.21	-14562.5	6264.2	7912.01	-9704.28	8860.56
-1101.72	-3624.35	-8210.25	-13599.6	-6103.63	2354.74	4290.83	-11378.9	-5078.73
-9132.25	-13580	-9842.97	-6995.29	-9118.66	1993.48	2167.41	-984.717	-1320.95
-3988.32	-4480.51	-5440.15	-9699.91	-13652.3	763.234	-6814.15	-13661.5	-6625.88
463.986	-7505.24	-3614.13	-7575.06	-10573	-370.844	1255.72	-11085.3	-8625.15

1806.6	-3989.34	-3766.91	-3425.81	-4323.75	1978.65	1387.85	-15217.6	-5331.6
7227.36	-1474.83	5236.17	-5412.33	-11909.8	173.623	2514.77	-17676.3	-5193.56
-2040.71	-9559.25	-4055.08	-5442	-7904.34	-3504.88	-4986.68	-4053.72	-6200.65
665.363	-7282.21	1924.08	-4127.56	-11014.9	-4467.26	-3927.4	-13836.2	-7226.94
-8880.28	-8542.97	-7269.44	-14195.2	-18182	-4833.32	113.336	-11038.5	-10911.1
7596.98	-5850.82	7796.45	-12018.1	-11666.5	4737.59	1746.92	-627.549	3942.47
-4094.35	-12802	-7218.57	-10766.7	-15571.1	-3395.35	-3401.01	-9589.53	-2144.9
-1537.83	-8954.39	3519.99	-15132.8	-10135.5	15228	6.32227	-2964.86	3019.98
2987.8	-3221.46	47163.5	-8221.55	-15371.6	49692	2093.14	-5188.1	46073.5
72711.8	-6581.84	1736.51	-12758.4	-9662.6	5767.95	46489.8	6784.26	-2464.7
1119.7	-4617.92	-5902.9	-14499.6	-9611.08	-4944.61	1417.14	-6548.84	-3776.54
-2569.11	409.277	-9872.39	-10962.6	-12210.8	-3043.04	-5160.86	-13566.5	-7812.13
2128.59	-5950.86	-10975	-13066	-10684	-2808.96	5832.42	-16074.6	-6372.66
-3612.71	-7208.54	-11697.5	-17223.6	-17224.2	-3731.51	-2062.05	-10404	-8879.85
-4639.61	-8076.49	-10998.4	-5829.46	-13704.5	-12278.2	430.451	-10855.3	-7073.54
-2581.77	-6518.75	-7931.04	-7943.95	-12475.8	-557.326	6113.82	-11677.9	-1109.9
-1634.44	69.5664	470.047	-8206.44	-6775.21	-3373.61	5461.64	-6418.44	1736.84
504.539	-2585.55	-8332.26	-6419.32	-7318.43	-1670.34	-2278.7	-16421.9	-7488.53
1241.72	9635.91	4170.11	-933.414	-6381.05	35360	2537.72	782.879	60539.9
22024.9	-3712.66	1350.09	-15318.5	-4062.42	6880.67	33258.2	-6377.34	-856.682
6220.17	-1109.59	-5164.93	-7329.95	-7302.39	2649.5	4081.78	60566.3	-4920.46
-994.232	-11871.8	-3249.8	-486.609	-3098.11	4329.84	-3082.23	-4198.61	-1686.96
279.381	-3401.73	-8221.41	-5501.71	-13536.7	4832.76	1357.27	-6806.78	-1869.27
-402.52	-13388.6	-10705.7	-8953.79	-5717.02	-1930.02	-2464.18	-6000.37	-8598.65
899.695	-3557.7	-4956.77	-14184.4	-18821	-703.932	1449.76	-15967.4	-1791.14
995.473	-14252.2	-7142.26	-15252.3	-10918	-5129.04	-4811.71	-10271.1	-1509.25
-318.293	-8145.17	1962.05	-3091.81	-13767.6	522.832	4624.13	-14183	-5801.65
-5769.11	-8645.92	-10217.4	-9109.77	-11002.1	-2981.36	-1109.47	-8240.19	4074
-7923.1	-7477.5	-6730.08	-7162.02	-16450.3	-5224.27	625.262	-9963.25	-394.936
879.928	-10105.8	-1890.52	-5478.48	-12537.3	-3153.37	1155.23	-7309.77	-7115.01
-4394.56	-9913.2	-1718.41	-7858.59	-12857.3	2062.88	-1656.64	-14775	-7103.91
31194.5	10012.1	13696.9	13236.9	-13479.6	19590.6	17580.5	-3434.63	32180.7
4939.64	-5217.6	-3964.83	-10484.3	-5423.61	2345.21	554.426	-6964.46	2052.66

1059.9	-4244.64	-969.205	-4996.89	-6264.26	-268.65	-7063.77	-5282.59	3593.1
62.5918	-13160.1	-1332.77	-7711.17	-10279	-5881.79	-633.934	-9984.13	503.758
-5954.06	-11233.8	-5056.48	-10073.4	-10579	319.557	3031.95	-13394.2	-239.811
-7369.84	-8350.53	-7424.44	-2883.53	-6484.33	-2995.55	51.8281	-10162.2	-1765.82
1020.21	-13144	-7426.21	-15173.8	-10587	-3513.81	-2471.94	-8047.27	-443.738
2511.17	-853.414	-10037.4	-9791.12	-8820.71	-383.365	1105.68	-6160.28	-12164.2
-2363.87	-2541.39	-2780.58	-11083.3	-15243.3	-2109.69	-2251.08	-11395.7	-8899.91
-4805.78	-1922.63	-4137.39	-9717.99	-5685.89	-7918.2	1643.43	-12397.6	-6042.48
-2463.91	-4775.51	-3902.21	-6846.81	-7153.41	-4118.02	3597.01	-4163.1	-4666.1
-4392.86	-10399.3	-5410.03	-8278.01	-11568.1	1196.15	4516.32	-6972.86	2638.41
-946.113	-8277.18	-5553.17	-11282.9	-672.008	3190.93	3325.46	-3701.29	-658.287
160.314	-7787.84	-8780.13	-15704.6	-10524.2	2149.95	-2509.84	-11693.9	-2259.21
-4098.24	-5396.95	-8760.7	-6667.5	-13142.7	-2598.66	-5005.68	-7733.93	-249.01
-2873.46	-4107.78	-7881.83	-11619.9	-15163.1	-3763.28	-516.107	-11180.6	-6827.45
842.988	-4538.01	-8893.88	-22.3926	-13834.1	-3788.29	2364.94	-8837.9	-8123.21
-11025.9	-10311.7	-10605.9	-13966.7	-13207.3	-1413.32	-520.371	-12674.8	-5936.33
-680.463	-10590.6	-11449.5	-9761.48	-12356.2	1222.21	-6075.32	-12135.4	-3251.98
52761.6	4331.04	4330.23	8062.69	-5540.88	3585.98	12425.4	-595.709	-56.5645
1338.62	-2572.88	-8343.83	-4048.44	-3579.93	-2127.62	1869.75	-8028	-2032.84
1173.31	-3656.48	410.221	-5065.4	-8311.96	-1199.34	-1812.07	-2742.52	-1724.9
4790.43	3892.21	5119.34	-10031.4	-7599.81	5884.77	12132.3	-4473.31	9212.32
4396.57	-8509.63	-5482.63	-11745	-13770.3	2932.04	-130.225	-13473.7	-1066.42
745.264	-7335.68	-5103.17	-12009.1	-9141.44	-5506.13	1597.68	-14086.2	-3016.03
-2893.84	-1792.95	-10724.1	-5499.66	-12770.6	-9953.89	4009.15	-12079.2	-4179.66
1600.22	-9381.33	-3385.63	-7766.11	-13280.3	-10204.7	2593.45	-14185.1	-1055.63
46.5938	-2538.82	-805.805	-10121.7	-6328.91	808.904	-5041.3	-8943.93	-761.461
2704.61	-998.449	-7255.63	-7125.05	-8186.34	534.973	-282.094	-8647.62	-7149.01
1090.48	-10748.9	-11246.2	-6771.36	-8848.84	-5598.94	4479.74	-11365.5	4085.24
-4695.7	-12360	681.08	-12389.9	-7292.74	-3225.39	-5908.34	-12785.1	-3982.34
-4098.15	-6381.99	-7799.28	-7550.5	-8623.18	1040.97	-4914.39	-7525.54	-7892.72
-1160.12	-2306.08	-1571.96	-6951.45	-8425.94	3409.3	331.068	-9218.55	-1989.39
10177.3	3235.55	5727.92	-2587.56	-1514.85	6091.47	8702.31	-14160.9	7499.74
4256.47	-10455.7	-3848.25	-9514.01	-13056.7	-3527.56	7315.87	-11829.3	-4708.51

-6052.03	-6754.34	-6772.12	-9600.32	-19061.9	4238.17	-2226.4	-22607.6	-4453.49
-2452.14	-10166.4	-7071.45	-6217.13	-8373.32	1812.23	3125.83	-9406.32	-3202.32
-3815.26	-6912.13	-766.168	-15448.7	-9752.84	1862.59	-3931.69	-13599.9	990.545
-1614.61	-9038.71	-2146.29	-6241.66	-11472.9	-1417.91	-2151.67	-10731.8	-600.629
1793.32	-7697.09	-6841.92	-10817.9	-9502.98	-2990.79	-964.58	-12226.1	-7984.9
-3672.05	-6944.99	-4384.83	-15572.5	-15958.5	-5180.77	1251	-13528.3	-6709.47
-552.873	-4765.13	-8395.79	-12861.4	-9639.39	-2662.82	-1094.89	-15263.7	-6045.84
1935.95	-2649.32	-2886.13	-10119.9	-18245.5	-4501.57	3013.69	-13764.7	-1523.46
-3690.86	-9856.73	-9056.06	-14774.9	-10973.9	-5821.46	-4669.29	-12288.9	939.666
1992.35	-13375	-5881.19	-9780.9	-12784.7	-8717.79	2570.84	-10609.5	-1584.99
-5781.62	-7465.31	-6631.69	-11070	-9870.25	-7555.89	-2807.06	-13879.7	1658.16
-1925.35	-2381.79	-4151.87	-7458.13	-19715.7	-3782.71	2953.32	-12542	2338.6
202.383	-8670.19	-4635.7	-11260.7	-8852.04	23.4199	-1691.52	-15591.1	2326.32
703.537	-7335.4	-5212.67	-11424.3	-11955.2	-6134.54	-2437.43	-13106	-1485.63
-4741.23	-11944.8	-6055.32	-10155.7	-13262.1	456.508	1567	-17227	-3546.15
-3824.84	-7494.87	-7021.31	-7666.98	-5738.65	-4330.71	3820.98	-20881.9	-3623.59
-4562.21	-9811.56	-7186.3	-5550.45	-18798.7	-5657.97	-2528.9	-18996.9	-13387.8
-5463.74	-13755.6	-771.422	-12418.4	-18923.2	-8471.07	4757.9	-10592.6	-6200.72
1261.96	-7247.47	-5008.09	-14800.4	-18053.3	5250.96	545.311	-13558.6	-751.674
-3379.46	-7410.77	-5810.24	-5833.02	-12885.4	-3065.27	160.324	-21024.1	-1166.41
-45.2617	-6467.07	-2511.36	-2820.87	-12780.7	-2042.31	-958	-12502.4	5499.09
4222.81	-4472.04	1342.18	-8805	-7918.19	2272.36	4117.24	-10526.8	-1275.5
4200.88	-4033.69	-7526.58	-14799.7	299.561	2946.34	-488.645	-6988.83	1200.63
1911.92	-10696.3	-8515.36	-9661.29	-14691.3	2454.45	2624.93	-10688.2	954.781
-1675.67	-2733.05	5866.77	-6688.71	-6793.87	4830.48	2265.55	-10335.1	3883.11
11883.5	-6060.21	5161.68	-2509.61	-5031.27	-536.584	-673.445	-6955.47	1214.5
-681.111	-7833.29	1046.73	-1405.74	-5118.6	1049.19	-141.932	-11622.8	5616.48
840.363	-6564.67	-2231.72	-2636.16	-8522.62	3736.65	-1619.63	-14980.5	4013.47
-3745.03	-4294.01	2177.62	-6967.87	-6540.1	-2282.22	-6255.27	-12902.2	354.27
-1974.15	-2459.76	-5219.52	-1732.82	-10701.7	-956.916	1393.45	-17365.9	-3046.15
-4144.76	-5343.37	-6413.7	-5318.3	-10069.6	-6842.02	-3130.48	-17463.1	-1435.4
-6423.09	-5992.05	-3645.32	-10237.4	-5103.42	-2733.33	4914.84	-10224.8	-4909.25
2008.78	-4964.31	-5873.44	-6709.26	-11279.8	-4411.32	-2629.28	-6952.61	-5670.08

-4207.02	-8642.64	-4266.56	-6169.27	-10577.7	-3654.85	1117.45	-6431.19	5816.5
10058.5	-1664.33	-2129.47	-11626.5	-12982.7	437.959	-8344.65	-7186.13	-3190.69
2518.06	-137.553	-7565.25	279.707	-5910.83	1408.52	467.43	-8897.46	2617.83
52873.2	21964	5807.22	13585.4	243.41	20778	16539.9	8120.28	17792.8
11301.9	33468.5	24356.4	27015.8	-8311.96	42085.4	7008.67	-8720.47	22572.7
9802.95	-1426.37	40541.9	-4049.25	-12339.9	10567.3	9145.62	-222.398	88282.2
50758.8	-5019.57	-1785.44	-2938.69	-13412.2	7575.17	68868.1	-11519.7	-1249.53
8866.36	-5938.39	-1046.37	-14139.2	-2007.7	3120.6	-2620.81	23537.2	-3035.67
-889.652	-6626.81	-7874.46	-9467.73	-9047.28	6373.3	-5374.42	-10098.3	-1707.82
-2825.06	-8622.62	-1803.34	-13712.7	-10389.6	189.393	-2456.43	-965.443	-1925.68
858.832	955.045	-3923.26	-9926.8	-3530.28	-640.475	-2892.48	-18242.8	-3111.49
-62.5586	-5919	583.277	-11.8203	-9677.02	-2159.48	2628.58	-10580.4	5120.9
7368.11	-1202.91	-4927.78	-3203.36	-6713.47	269.645	10105.3	-13427.1	637.23
7948.98	-691.236	-1651.17	-7679.31	-9225.18	-3380.13	2104.32	-12152.7	-2058.6
653.842	-3535.89	-6979.94	-10573.3	-13890.4	206.244	1331.38	-12445.8	-691.594
6519.71	-1511.16	-9607.15	-4414.26	-9392.54	-2731.95	-2967.03	-11941	-3193.39
3076.57	-71.8594	-4155.83	-11112.6	-4195.35	4284.98	-4702.93	-6501.71	-4748.9
-4753.36	-6464.13	-5476.93	-10133	-3996.2	-1963.49	3047.24	-14625.8	641.865
-1682.65	-2063.14	741.715	-9983.58	-13049.8	-7497.32	-2761.84	-9994.59	5872.84
-4736.96	-3976.98	-4388.75	406.828	-15723.4	-2489.27	7554.85	-8868.09	-2552.9
4611.38	-3777.1	-606.27	-7710.5	-9039.11	-4404.68	8012.72	-8274.89	3799.86
-915.227	-794.629	-3689.43	-2776.6	-10562	-1609.57	-172.115	-9823.32	-3778.2
-1722.96	-11220.9	-3679.57	-11081.6	-10150.5	2104.6	-2318.71	-11128.3	-3385.49
-7917.79	-6641.3	-4234.02	-7036.06	-9408.96	-2397.11	470.613	-12584.6	-932.324
928.295	-1612.99	-3162.15	-4301.3	-10042.7	-1285.03	-1605.98	-13096	3494.21
-7130.69	-868.74	-3743.84	-9233.02	-5212.57	-6470	4306	-12042.6	830.715
-1169.45	-1252.5	-5120.33	-9252.37	-9521.12	727.629	-3302.43	-8666.09	-3593.4
2615.38	-4789.1	-5242.09	-6466.86	-5043.4	-4756.63	2953.66	-12168	-4833.89
573.57	-9036.22	-5048.04	-7640.93	-6300.16	301.855	4649.93	-5057.47	-2784.36
-370.059	-144.545	-3202.49	-4570.52	-6954.21	722.627	-5381.96	-20640	1435
-3972.47	-9489	-2415.87	-466.732	-5085.8	-3379.23	1349.92	-13849.5	-3959.23
-1538.78	3673.68	-8786.15	-7695.63	-12978	-594.834	-113.541	-6528.45	-1769.73
-3213.64	1107.27	-6207.27	-5868.93	-9077.21	-6791.23	-2252.85	-9045.04	-1013.05

3832.44	-9199.12	1074.2	-7216.26	-6691.5	-2562.44	1564.01	-19284.7	-1619.69
-2525.16	-5725.42	-4543.96	-9885.53	-8558.25	287.053	-7126.91	-10761.9	-9957.36
-2407.51	-2249.34	-3718.6	-7013.65	-9049	39.0723	2016.19	-15422.7	-3735.69
2040.25	-4018.37	-2177.91	-12782.3	-9912.99	-5734.39	1351.12	-13373.5	116.582
847.281	-11558.1	-5390.07	-12341.6	-9034.81	-4584.81	-3902.65	-13741.9	-3294.18
3124.63	-7547.67	-3124.13	-4630.4	-9857.36	-4240.54	276.791	-12868.7	5000.85
-2281.34	-5861.59	-11375.1	-9599.19	-10847.7	-2080.96	839.66	-13377	-595.025
34.1152	-4096.84	-2906.79	-14978.8	-11472.5	-3922.79	-9737.77	-15775.3	-5144.17
6893.39	-4588.61	-150.699	-8227.44	-6267.74	2347.14	3305.63	-1842.02	2945.13
330.096	-3390.13	-2767.33	-11502.6	-8539.15	-5662.89	7072.65	-6157.91	5805.78
3325.85	-3959.58	-1141.14	-3139.81	-5348.51	516.951	1542.75	-8712.15	4954.43
6071.27	-4758.03	-2402.17	-4168.19	-10855.4	-1424.09	-6543.56	-10175.3	-1618.54
8628.88	755.867	-2271.42	-7773.62	-9574.46	-6462.18	-5367.07	-7890.4	-11016.7
751.627	1262.88	-6919.53	-7714.27	-12378.1	-938.775	597.365	-7338.21	-284.428
-124.082	1511.36	-4840.37	-6637.57	-5857.78	4090.63	1963.45	-5651.43	-3093.26
-4004.6	-11252.5	-15910.8	-17301.2	-9815.01	-9674.57	-9198.32	-13144	-5673.05
-1635.18	-6757.89	-3442.4	-16864.8	-18597.3	5527.37	-4558.54	-19030.3	-4843.47
-3557.03	-9419.44	-6426.27	-13234.4	-18525.4	-4598.21	2888.17	-14886.4	124.641
-3687.24	-10910.7	-3406.31	-8923.06	-10024.8	-8932.13	1945.62	-14686.6	-5390.09
-2124.69	-11759.5	-5562.81	-7190.53	-13096.3	-4098.15	-1580.71	-15428.5	-3815.11
6458.37	-6886.09	-2494.96	-14120.5	-8118.49	-537.168	-804.123	-19125.6	-5308.53
-514.42	-10503.8	-5776.47	-11850.7	-13370.9	-1263.12	-2110.8	-5732.23	-6175.16
3557.65	-8626.82	-4691.89	-7594.83	-19944.3	-2567.56	1612.63	-11374.3	-8410.5
-328.98	-8070.59	-7446.65	-15088.1	-8038.89	-4116.29	-200.258	-10090	-7485.69
-1017.8	-5610.81	-2864.59	-5210.39	-12401.6	-3199.38	2922.15	-13430	-4787.33
2975.11	-11010.5	-6467.8	-10718.6	-4640.7	-3917.05	-2360.51	-11405.2	-3328.35
3317.42	-8351.88	-2638.65	-5541.85	-12193.7	-7138.54	-1716.33	-11683.5	-3236.77
4597.89	-3439.88	-1365.02	-7205.79	-11017.2	-1508.2	-9227.69	-14570	1802.32
1802.71	-1145.16	-222.586	-11150.7	-6121.25	-1739.71	-549.318	-4123.52	-3808.91
3228.16	-1160.82	2504.94	-9693.74	-13509.3	610.018	6000.57	-12163.4	-5958.19
-3111.03	534.205	-4588.94	-3514.16	-13463.2	-1313.61	614.697	-9076.88	-2349.28
-996.828	-4128.61	-5845.43	-4140.04	-16383.8	470.959	1531.91	-15997.7	-1988.63
3073.85	-13370.2	-8054.55	-9540.3	-15097.7	2719.31	1228.54	-8382.04	2202.77

-111.018	-4149.53	-11124.5	-10994.3	-4529.59	-3230.54	-1091.64	-17659.3	785.508
2508.64	-3754.45	-1445.37	-8594.74	-10718.4	-917.564	4310.36	-6201.51	-3779.38
-39.1465	-6695.05	-4532.33	-10614.7	-11861.6	-4218.02	1670.99	-11076.6	2558.75
2584.99	-9786.9	-6487.9	-6332.69	-8803.38	-3001.81	219.824	-9861.91	-2804.42
-69.2285	-3475.67	-9095.54	-8001.04	-16428.6	-5064.06	6733.94	-3663.1	-3843.29
-2825.86	200.535	-7364.64	-6549.96	-15755.2	-1046.38	-3501.69	-9859.23	5111.22
-653.9	-1456.57	-5490.83	-14515.2	-17398.6	-3448.74	-5484.99	-6069.46	-5240.85
3798.22	-2114.63	-9265.52	-7487.1	-16221	-4734.9	3098.64	-9348.19	1047.13
-3025.79	-8158.37	-3262.4	-4520.2	-14982.8	3417.58	-3744.82	-9622.4	215.711
-4155.01	-6436.69	-2404.57	-14189	-6821.43	-2920.28	-682.775	-9822.38	244.73
-1638.24	-7356.91	-1579.4	-6355.07	-4903.55	2275.4	2074.79	-6864.64	3731.52
7458.11	-2711.71	-4948.91	-4893.66	-8709.27	-3096.99	2122.7	-13660	3625.3
1855.4	-4810.52	-958.492	-12917	-8774.83	-4662.84	-1475.24	-16607.5	4103.09
2609.23	-8617.45	-4121.74	-9281.58	-12605.9	-3387.68	4042.33	-13127	-2038.96
1570.82	-11380.6	-17.5039	-8258.91	-13281.8	-5656.51	2733.46	-8041.64	-709.994
2806.13	-5856.22	-2578.08	-5964.37	-9826.47	3662.9	-3412.26	-14989.5	-2022.88
1250.4	1759.51	-7827.84	-855.387	-8837.71	-761.217	2461.51	-4100.49	-2058.09
960.479	890.75	-4749.92	-5688.38	-6968.06	-4531.68	-7297.81	-9692.56	-2280.17
2386.67	-49.8223	-549.363	-13313.8	-8222.25	372.336	-4071.79	-13760.8	-938.723
2021.29	-749.686	-3617.12	-7067.61	-13377.3	-2229.23	-2153.8	-15244.8	-436.545
-3101.09	-6430.6	-6728.71	-5517.63	-11066.9	-4355.03	2611.77	-9541.01	452.67
-4682.76	-2752.49	-684.826	-6428.45	-13544	-2826.51	2134.57	-8276.14	-6072.61
-4901.92	-3050.69	-10754.8	-8834.35	-10797.8	-1862.79	2430.06	-7412.76	-7906.8
3034.1	1011.12	-9343.68	-3825.14	-4668.39	903.316	-591.332	-5064.72	-1603.57
2235.17	-1756.71	-6090.33	-9333.42	-19673.3	-5632.52	815.346	-5959.49	-5434.43
-3723.63	3761.21	-10136.2	-13970.6	-15598.1	256.479	-1713.58	-8089.44	-3414.51
2496.78	-8647.62	-7944.67	-4003.89	-7053.05	-1135.79	5570.08	-9651.24	1062.59
713.422	-7432.08	-3636.61	-7496.96	-14094.6	298.17	-1864.06	-18209.8	-9070.64
3504.71	-12472.1	-4414.83	-10127.4	-13541.7	-7169.17	-486.852	-4282.45	-5352.32
-2994.01	-6895.38	-170.799	-12536	-12430.6	-623.418	-729.145	-3156.79	-6680.97
6377.63	-3940.14	-1938.18	-7629.27	-8648.96	-3492.74	-883.084	-9307.94	-3603.53
-2698.46	-6179.77	-7176.35	-14565.3	-11745.7	-5504.79	2098.52	-16055.4	-9267.44
-2731.57	-1183.98	871.092	-6293.32	-8266.08	2290.49	-1643.39	-18228.2	-1159.29

-6567.82	-4393.51	-2164.97	-11572	-11933.4	-497.029	1389.52	-11302.2	931.504
2429.14	-919.164	-6239.5	-4378.38	-5948.22	216.631	3286.5	-10098.7	-2632.38
5138.55	-2894.3	-5221.32	-3595.22	-6050.87	-6334.65	-3150.87	-7533.99	-3650.75
2267.08	-6997.73	6554.71	-9697.59	-6859.15	-1872.07	4010.45	-9855.21	-647.184
5146.92	-824.637	-3639.06	1328.04	5082.73	-2725.25	-1981.88	-10080.5	-1154.41
2690	306.611	182.211	3212.14	-4105.25	-1941.73	-7106.74	-2651.43	226.848
-2168.92	52948	5149.44	83691.2	-11092.2	-5774.18	-2929.59	-11588.4	-2683.74
399.744	4707.52	14249.3	-5640.91	1017.45	-3589.99	-5039.82	-11386.4	277.035
-1746.31	1075.27	-975.381	829.104	166.25	-5779.3	703.328	-17338.1	-2224
-2879.14	369.285	-1524.85	-8751.39	23628.8	-3057.64	2437.6	-10922.9	3543.39
-3077.62	-4746.95	-6554.87	-8903.11	-2449.03	-1403.26	-2166.65	19309.5	561.207
468.742	-4041.13	-2964.39	-5565.14	-7657.53	-3008.34	-3124.52	-3299.31	1372.74
2644.35	-3118.09	-5367.67	-7016.75	-16089.6	-12750.8	-1385.99	-9676.24	5383.08
577.986	-7369.3	-2919.59	-7771.22	-11516.6	-464.699	538.391	-19928	-5195.22
-1276.87	-8237.83	2008.27	-10090.3	-9937.29	-500.041	-4710.69	-4184.43	-3260.98
543.764	-10386.4	-1861.62	-10993.6	-16885.1	-4820.68	2226.66	-9711.88	-9911.29
-3101.03	-8032.27	-833.877	-9300.93	-14142.9	-3229.34	-10021.4	-12734.8	-7187.72
3683.79	-15523.4	2177.76	-11368.9	-9418.13	-11078.9	-737.301	-18695.4	1685.45
12368.9	3899.9	8260.6	-532.848	-11691.3	4231.24	9855.54	-7749.72	12157.3
3430.3	-285.859	-4620.76	-2193.73	-5063.69	11563.1	1260.3	-5676.07	-3509.63
-4199.27	-7131.67	1363.29	-3531.29	-6584.5	1649.3	-1416.33	-5551.75	-8980.12
-1680.52	-2302.08	-2168.37	-11044.5	-10475.5	1372.89	-3816.58	-11206.1	2861.05
5420.57	-3646.08	594.484	-7101.02	-11973	-3063.58	2724	-12426.3	-2289.34
3402.41	-2850.54	3212.64	-11662.7	-10627.6	-4033.64	970.553	-6016.64	-7556.87
-8965.53	-12799.9	-12980.2	-24721.2	-19952.3	-9134.93	-8341.59	-25805.4	-14960.9
-7075.99	-6402.52	-4904.36	-1151.92	-10304.1	-2600.24	-8398.44	-8376.49	-7666.9
-6138.69	-6421.09	-4789.49	-7833.43	-16426.1	-13195.3	-2806.26	-10849	-4077.11
-1450.39	-3674.71	-4929.01	-11092.8	-8166.24	-5943.26	1958.91	-11813.9	-1360.04
3669.02	-1960.16	-8553.74	-5676.26	-8559.2	-5141.65	-1503.23	-9983.91	-7896.97
7374.07	-3164.63	-5452.43	-9989.32	-8704.69	-3092.92	-3253.07	-5204.55	-3403.44
3116.32	-2628.4	-4273.57	-15932.6	-12021.7	-836.172	3641.71	-6519.86	-857.027
1697.14	-6612.16	3546.54	-9577.09	-12254	-3508.42	1810.65	-11060	2125.92
3708.96	-4686.63	-2943.37	-12178	-10899.1	2056.91	5779.9	-8354.94	2300.35

1453.02	-13527.8	-8048.96	-9131.38	-8447.57	-858.291	-6425.43	-5087.7	-6781.62
283.766	-6542.44	-6353.53	-12473.4	-5753.36	1733.27	-4764.93	-11677.4	600.187
1476.12	-5523.89	-5354.02	-4451.69	-5105.42	-4795.11	3847.21	-14526.1	4934.3
-1794.67	-5315.23	-126.805	-15699.8	-5150.59	1585.09	-2737.89	-7093.38	-4890.71
-1006.09	-4779.76	-606.449	-991.635	-12844.3	-3123.35	-1502.24	-11297.7	-4437.1
-1381.73	-3025.19	-2626.32	-4856.27	-14981.5	-3513.16	1546.67	-7477.47	-5804.18
112.195	-6117.4	654.76	-7290.3	-10616.5	-2250.88	-12319	-13657.8	3172.03
1018.82	-5434.15	-6815.82	-7223.84	-11475.3	58.4102	-5753.57	-13761.8	-2742.61
5890.74	-4512.51	1362.39	-7776.83	-7768.36	1875.98	3407.32	-3903.19	-4611.73
4620.62	-2665.73	-4.81445	-8741.74	-11373.1	3392.48	-205.387	-5744.53	-845.164
-2589.52	-2872.8	-4069.1	-11652.9	-6432.33	-2485.23	3113.75	-10970.4	-1227.6
507.104	-1104.25	-1666.98	-3281.21	-14058.8	890.414	2786.35	-8545.13	5875.68
-6668.02	2535.72	-1962.54	-2043.91	-19150.4	2592.37	-140.295	-8207.99	803.908
3776.13	288.629	-6896.65	-6125.85	-14764.4	-6330.56	-3082.04	-11777.9	-4513.95
6435.85	-8148.98	-5045.99	-11895.5	-6984.91	-824.645	4867.82	-6729.28	-3562.51
-2755.57	-237.898	-3376.91	-6263.12	-11253	-6984.71	-3116.55	-6045.67	-4587.45
5533.86	-9128.14	2360.72	-2189.92	-15963.2	1712.53	94.3672	-6179.69	-6556.53
4369.3	9171.88	6975.36	1553.97	-9783.16	13707.7	3871.18	-6358.87	6685.51
6935.96	2646.63	5172.86	-6135.19	-4855.42	69.5566	7369.31	8337.7	-575.967
4188.68	2613.72	-1535.78	-5569.34	-7630.13	650.041	2777.05	2821.25	-1875.95
916.404	-1003.48	-1145.88	-5782.1	-7089	2031.11	2516.5	-2610.91	-6971.44
2910	-5925.07	-7110.19	-5389.39	-16152.9	-1348.84	-4408.18	-9162.87	-4885.28
-3103.1	-4489.16	-3181.74	-9075.17	-8161.37	-139.629	47.1934	-7747.08	-4908.7
-1086.49	804.277	-6302.47	-7593.95	-12659.4	-9498.27	-146.504	-7195.47	-6261.56
2595.46	-2903.59	-2006.02	-4264.02	-17496.7	-3677.06	-5715.42	-9561.09	-7588.31
-1856.14	-4310.59	-8415.46	-2248.04	-12954.8	-8042.31	684.113	-10736.1	-2032.35
4090.8	-1319.25	-2699.15	-3061.29	-14741.8	1454.8	-2132.46	-6145.84	5448.87
-1290.06	-2024.4	-1162.97	-3959.52	-9000.01	5615.46	-4986.47	-10835.6	-184.953
6020.53	-2797.88	-5980.04	-4991.11	-10420.4	-10784.8	-1442.55	-13679.6	923.271
479.521	1663.08	-7283.54	-7822.08	-14687.1	-3574.4	2957.09	-8635.77	-3164.27
-4891.21	-2845.98	850.051	-9456.56	-12215.1	-4980.8	423.5	-5373.14	-4089.32
623.018	-358.434	778.164	-6857.45	-9227.52	-2981.28	4276.68	-11771.8	-9289.51
4558.26	-1114.12	-2332.82	-13018.8	-12167.2	4054.76	-4158.04	-21075.4	-880.824

-1515.9	-4002.37	1512.97	-2081.44	-6692.65	-5559.35	2489.65	-6057.11	2200.53
-1145.17	-3775	907.945	-4616.62	-3970.46	-6865.9	5148.57	-9295.1	2822
2402.67	-7478.62	-9211.02	-5682.99	-12362.7	-3853.07	-606.717	-14790.8	3715.19
9109.12	-7610.65	-7301.04	-787.934	-13783.2	-5469.42	2811.21	-6195.96	-4100.66
2734.85	1617.8	-3730.75	-4171.51	-15158.9	-556.596	5710.54	-15725.3	-2649.12
-486.467	-9934.84	-7448.9	-13053.8	-8133.58	-2274.46	1368.55	-9185.81	-1012.81
2523.28	-891.729	-7636.82	-7394.38	-8087.98	-4648.85	7561.77	-13436.6	-4716.6
4132.44	1990.62	-5144.25	-6273.73	-5795.81	-3204.97	-595.996	-11570.7	429.912
-1841.62	-9052.08	-4632.35	-10696.9	-8140.6	-6608.99	-1209.13	-11387.9	576.875
-1373.88	-1716.94	2631.06	-13226.7	-5806.98	-7746.12	-3324.18	-7534.53	1297.89
24036.8	2903.14	3073.16	-6050.75	2075.25	-584.096	5857.26	-8935.52	4215.4
25150.4	3206.86	4052.46	-719.914	-2880.57	198.375	8506.73	438.232	-380.305
-53.1074	-6063.9	-8435.14	-7691.29	-10652.8	-2008.29	-246.846	-8586.04	-127.191
-5054.84	-5135.44	-3681.3	-5289.23	-8979.67	1237.43	-3408.67	-6759.64	-3539.17
-3891.95	-3834.4	-4913.29	-11908.4	-10311.1	-4510.76	-626.025	-9992.67	4246.84
-2409.74	-2217.18	-2943.46	-9268.05	-11077	-5905.49	-645.271	-12688.9	-2564.97
4333.72	-640.896	130.266	-6167.61	-13542.1	-3182.53	2943	-7687.24	-3953.49
265.729	-8425.32	-944.232	-7847.07	-14634.4	585.412	2823.63	-3749.81	5885.08
-4676.83	-8666.19	-5241.35	-4331.3	-8238.29	-2562.7	-1962.33	-6035.6	785.023
-233.574	2125.7	2017.37	-2777.06	-331.949	-12408.4	4367.75	-3774.85	3314.29
8472.53	-2445.92	1216.87	175.529	-4328.19	-99.3418	343.395	-3518.39	9300.83
3805.38	-1714.39	-9846.23	-9624.49	-14544.5	-5578.07	-3371.54	-9101.52	-2175.2
1158.86	-11322.1	-8936.99	-13789.6	-18250.9	-2404.89	-4615.63	-14785.9	-1828.73
-1157.99	-9775.74	-2186.92	-8129.71	-16257.8	-3271.71	-165.133	-12792.3	-6145.83
-4912.15	-8089.41	-5556.23	-9498.85	-9265.87	-3944.79	-3039.35	-9711.97	-8585.72
1883.32	-6423.13	-8692.43	-9372.47	-5875.12	-2619.49	1408.46	-7791.66	-2207.61
-3417.73	-1900.99	-1800.63	-7256.26	-8483.71	-2422.53	-1165.51	-12291.5	-7662.88
1824.82	-1250.57	-2450.25	-5996.72	-7655.29	-2825.07	-2788.5	-11171.7	-2394.94
6271.32	-394.035	-8160.42	-10524.5	-14171	-5763.65	-6073.68	-8343.8	-5101.56
-1368.39	-2894.86	-6097.74	-5162.21	-17301.5	-6279.45	-8274.15	-15552.1	-7768.93
-2404.73	-941.957	-3546.42	-8242.7	-8665.77	1495.27	-7599.98	-8422.82	-7203.77
-2850.29	-315.42	-10506.7	-14210.3	-13453.8	-8134.15	-6246.93	-14606.4	-7284.94
-3210.86	-3039.59	-9257.69	-2477.78	-8780.61	-3058.57	-3983	-8100	-8556.91

2266.71	-4778.3	-2998.64	-3924	-6547.54	-5397.97	-3058.04	-8879.94	1929.01
2364.92	-2851.94	-1226.95	-8080.72	-4794.25	-8976.83	2010.04	-7985.38	-687.617
2639.69	-3272.8	-6524.57	-4677.03	-11269.5	-2691.22	1593.72	-8315	652.414
1607.88	3922.61	-3384.23	-10188.7	-11389.1	-6627.08	8106.88	-9150.62	-6706.19
4275.22	-552.717	-7793.91	-8020.14	-6494.5	-2607.32	-477.924	-10202.8	3866.82
20073.9	3468.26	1822.73	962.674	-14145.6	5182.41	7886.71	-6641.59	1790.07
6669.56	-239.492	1325.25	-9586.32	-7847.19	-2985.4	-2009.78	1176.63	-6353.43
-3823.88	-5285.17	2523.89	-2541.39	-8895.49	637.256	259.553	-9190.82	-5898.18
13018.4	9687.51	1079.16	5235.38	12366.9	3542.3	11823.3	9635.3	12866.1
-699.18	-4362.13	-363.393	-4740.43	-9235.74	701.357	-853.211	-8699.76	385.848
3325.32	-7364.46	-2444.43	-6903.62	-2165.12	-2967.23	1253.69	-3875.29	-7226.18
1376.51	-5373.74	-2710.78	-9280.56	-7677.87	-1146.32	-6446.22	-9738.98	-1788.72
2823.12	3140.78	-8782.91	-9970.89	-11399.2	-4305.79	-1179.5	-7335.22	-7208.37
3164.5	-8095.37	-5517.53	-4944.1	-11683.6	-3739.64	239.811	-10798	-3715.6
6374.32	-8341.5	-166.756	-9330.6	-12537.5	-6490.83	-349.564	-17084.8	-6251.81
-2790.53	-4909.41	-9224.9	-11673.5	-11227.1	-7650.01	-5666.05	-18093.7	2502.27
2228.59	-1458.05	-6611.41	-7393.78	-12811.9	-9111.88	-651.48	-14745.8	-2600.77
521.65	-5329.27	-5619.87	-3777.46	-9405.86	2070.04	1031.69	-8390.49	-3122.45
2023.11	-5498.64	-3506.16	-8451.94	-11894.7	4282	-6911.93	-12284.2	-2691.11
-1401.65	-2301.58	-2582.73	-5612.68	-11414.3	-82.5195	-5494.16	-8557.6	-3762.36
-4741.6	-2116.05	-1025.89	-8653.21	-14618.4	-2032.66	-3253.44	-8523.3	-8810.5
-327.992	1062.86	-7724.92	-14336.1	-7562.99	-4651.25	-6135.13	-16315.4	-2609.07
-2370.02	-8697.96	-6386.19	-7366.96	-16042.3	-9562.14	-3911.73	-15103.3	-3456.36
1179.49	-1528.86	-8500.94	-5384.91	-9441	-8728.41	1299.35	-11362.1	-1996.76
-3240.69	-1974.68	-3046.38	-7444.07	-11944.6	-3933.88	2445.5	-11398.7	-589.492
-4488.08	-5307.12	2036.46	-7239.7	-13908.6	-7613.82	-3847.94	-8464.05	-1874.84
-2680.48	-7554.83	-1015.56	-14566.3	-14488.4	-11489.7	-3529.43	-15339.8	-7309.02
-4349.81	-5295.77	-3953.49	-17052.4	-10721	-10748.2	-9230.51	-17352.1	-7113.49
1660.85	-5152.86	-7009.13	-13119.5	-10984.5	-4280.04	-5760.49	-12604.6	-6938.2
5521.56	-1387.97	-3829.63	-5626.85	-6559.43	-6586.82	-285.553	-595.441	5892.11
-281.91	-2597.27	3430.23	-4298.7	-3086.13	-5745.83	596.686	-6591.28	1685.34
4293.66	159.424	4334.19	-7667.26	-2201.27	-4515.15	-1778.98	-5319.43	11090.9
4229.11	2780.26	3432.21	-5006.09	-5634.99	-6333.34	4827.22	-522.236	2176.52

3596.81	-4327.23	-2585.56	-7575.8	-11887.7	-191.734	869.965	-1942.37	-6806.94
-2648.36	-1466.61	-3837.9	-4287.03	-10898.8	-2190.44	7232.02	-147.27	-3781.56
5154.92	345.793	-5742.71	-5396.75	85.2695	808.086	9692.3	-3328.11	3967.61
4245.26	-79.5781	2453.49	-9807.12	-12588.8	-4137.85	307.313	-5145.39	4454.95
8465.86	-3647.08	-5099.58	-4897.75	-6540.48	1852.2	3432.69	-9460.03	4159.33
-2102.17	-2817.26	-5837.57	-6536.71	-5984.36	-8100.36	-168.377	-11999.9	3084.35
556.15	-5549.99	722.334	-9129.02	-9779.49	-3695.08	-1607.12	-18579.2	1367
2760.36	556.186	-5200.64	-12580.7	-379.314	-2829.32	2805.62	-13933.9	-6489.91
970.949	-2266.71	-1192.77	-7509.2	-8562.88	-1295.79	-1297.7	-5770.42	5843.75
-3989.92	-2830.04	-3925.97	-2987.85	-14972.1	-3143.18	-5578.75	-8247.26	-3566.18
-2505.98	4890.79	-554.326	-7630.68	-9424.29	-2156.85	-6152.86	-8856.86	5470.48
-2479.11	5412.78	2452.5	3829.52	-6231.84	-1705.8	4843.07	-3730.76	2488.05
1036.55	-5508.68	532.568	-1116.18	-4940.31	-11266.4	-4414.33	-11349	4116.1
-3550.63	-8995.72	-2431.34	-5512.63	-2897.76	-625.289	-832.482	-10201.7	1840.64
697.885	2534.52	-4530.93	-4004.74	-6972.07	2360.59	-1119.38	-11102.4	-1604.57
5809.17	1050.47	-5832.45	-6571.18	-13950.9	3159.19	-3636.72	-9488.24	-3407.28
1088.65	1614.36	1467.02	-5684.19	-13667.3	-4684.6	-26.0527	-6054.3	3184.65
-5223.91	-5462.11	-375.205	200.27	-4403.87	-2564.16	1684.86	-15685.3	-9197.4
-263.439	-2008.66	1690.77	-5641.2	-3630.5	-2486.46	3777.79	-7239.56	1527.48
-678.207	-2539.19	-2612.21	-6633.7	-9888.49	-5558.44	8347.32	-13857.8	-2615.88
4127.62	-668.863	-7910.35	-12307.8	-10618.3	-206.188	5333.62	-21629.5	-4721.06
-734.359	-247.266	-185.553	-4847.57	-10393	1933.88	23.3438	-4600.09	4461.9
31.8906	-4604.74	-2419.64	1579.74	-7615.66	-2959.51	-3382.88	-6493.66	-3766.68
-1978.72	-2357.38	339.031	-5818.01	-8002.13	-3760.65	6495.81	-10049.9	-1246.76
-2948.42	-3666.52	-7842.67	-3382.18	-4769.47	-9765.24	-3763.39	-14353.3	-5646.77
-1625.26	-1759.31	-8356.31	-6140.05	-14164	-10261.6	-6150.73	-9973.75	-4074.65
-3185.22	-4019.79	-5179.05	-3720.51	-6732.66	-5003.74	-5709.98	-10905.6	-2428.35
-1628.54	-429.301	-2717.36	-419.611	-4711.46	-12196.4	3234.91	-10346.8	-4013.54
723.676	-3287.93	342.467	-2883.67	-8420.59	-6501.71	-6030.34	-11867.1	-1912.51
1463.21	1102.77	-4680.79	-6676.2	-9242.56	1372.92	974.568	-8330.03	-2485.35
-491.439	3627.84	-773.309	-1956.47	-8652.5	-2598.51	-3609.94	-11716.3	-6353.36
-4427.12	-7027.54	890.877	-4112.9	-12830.7	-6878.51	-10456.2	-12287.8	-1687.72
-4915.98	-2291.43	-2168.28	-6300.02	-7243.23	1096.79	-4138.95	-3019.06	211.145

-1837.23	-3872.88	2723.67	-8366.26	-2909.56	-4815.7	-8877	-5659.52	-4408.53
307.611	-4857.78	-7736.82	-2685.04	-15855.2	-3400.29	-3444.54	-5999.74	2030.67
-5300.48	-8149.48	-5587.74	-2964.34	-16359.5	-4731.74	-5069.82	-13757	-6424.3
1393.07	-4512.98	2241.11	-3644.31	-6533.08	-2786.16	-4072.47	-6226.42	569.646
9668.04	179.477	7740.02	5793.03	-1873.58	-3230.98	558.656	4422.18	7260.46
11331.2	5518.86	7099.35	2623.1	-2159.25	6941.55	6872.09	4862.79	17348.1
-243.457	3309.89	5621	507.375	1904.46	2790.76	-2277.47	-4903.19	5275.51
9704.42	409.055	-2756.59	-4127.14	-3002.52	-3201.73	6093.28	5323.75	4940.67
9093.15	-1126.79	4827.78	-4809.36	-5364.93	-2967.26	4411.71	-2804.37	3906.39
2249.07	-918.828	-5182.31	-3769.25	-9871.78	-3557.81	-4941.21	-12485.9	4457.2
-3318.87	-1015.84	1678.55	-2266.54	-5672.14	-2034.29	4785.92	231.9	6057.85
9157	-274.729	1934.65	-5220.22	-2649.09	2901.84	5104.61	8365.02	5492.93
2541.78	-3393.09	697.924	638.441	-3273.78	-3667.11	647.32	-5427.34	6143.52
6890.98	1074.44	-74.9941	-1716.44	-10477.4	2487.22	7082.25	48.5078	11775.2
4914.9	-670.373	5636.07	8085.84	-6966.63	2096.49	-3754.18	-6149.28	12727.6
7461.2	-170.693	-1551.84	3894.12	12934.3	2122.01	7051.54	15575.4	7304.08
7621.65	2853.31	384.934	6625.67	5684.87	-6001.24	1123.81	14062.1	15139.9
8951.23	3074.1	11745.9	10057.7	5204.1	1776.83	-2395.04	5738.65	17902.3
10576	36.8457	9024.6	6713.6	-1958.81	1671.04	9183.9	3821.56	14074.8
8682.8	5157.87	7351.44	16601.8	4429.78	2846.26	8475.21	14022.9	19655.8
10869.6	10585.7	18048.7	17142.8	7097.54	4055.12	5349.96	12594.7	19077.8
22766.3	14432	19880	26135	33299.2	9068.99	17874.1	33086.7	34110.1
19364.1	29955.3	49325.3	38499.4	44692	22387.3	22101.3	45803.2	52402.4
116358	115840	228404	164012	215955	117828	123043	177382	190296
4.43E+06	2.41E+06	3.90E+06	4.90E+06	1.11E+07	1.61E+06	3.97E+06	9.01E+06	7.38E+06
4.02E+06	6.78E+06	4.93E+06	4.11E+06	895928	6.71E+06	4.76E+06	2.16E+06	2.74E+06
115017	129651	171817	80690.3	132688	861101	118381	126572	100897
26404.4	29387.4	29203.1	3172.25	27095	406099	25465.3	14612.2	6792.94
14065.5	14253.9	15017.9	-8037.52	6927.31	278846	15153.9	4315.97	2945.82
6419.29	4370.83	9142.15	-13418.3	6042.12	209271	-1079.35	-1737.87	-7186.51
10998.4	5204.85	4472.5	-10528.1	-3852.19	147243	6043.54	205.068	-10226.6
8597.03	-1227.44	3424.88	-8613.38	997.727	110346	6395.81	-5719.35	-2123.87
10813	2895.79	4263.45	1172.44	3847.73	78358.1	-1702.98	-5967.23	2012.02

-1036.33	-355.385	-2036.52	-8950.94	-336.391	51555.1	500.021	-8516.96	-4091.5
1493.11	-522.793	2730.46	-6946.97	-3553.58	32554.2	4022.43	-2862.1	-8345.78
7944.07	-2345.35	4.12305	-7170.37	-6570.13	22782.2	1467.87	-7342.11	-4802.72
-569.674	3211.62	-2743.59	-8285.59	-976.686	15961.1	5612.15	-8191.36	-2284.22
9797.72	-2366.27	-3226.33	-5969.3	-247.197	6083.25	7016.57	-2162.25	-4427.76
-2212.1	-591.34	1308.63	-3241.49	-4022.27	785.645	-38.5957	-2612.92	178.494
2610.37	-1008.32	-249.377	-11934.5	-8081.44	-84.7715	-4812.54	-12095.6	-13025.3
7199.75	4454.12	-2718.66	-9058.78	-700.273	-2452.64	-203.996	-3591.1	-336.275
4519.13	-1899.17	1203.1	-11629	-7426.01	1772.5	-1163.62	-6761.34	-4220.75
3457.95	-1572.55	-2264.37	-10017	-4776.25	-7332.51	4422.62	-5694.22	-4982.06
9602.68	299.732	-7583.87	-5959.68	-3780.14	-3807.52	-928.621	-3487.71	836.041
13272.5	-1385.98	7201.44	-3110.35	-1491.98	-4589.31	5187.47	6210.65	4088.7
8109.38	34.5527	-1733.39	-4453.8	-463.541	-4087.06	-2354.7	7861.49	4177.07
2736.73	-2983.16	-4126.59	-14641.6	-9920.34	-2740.03	2306.86	-8558.99	-7532.35
2820.12	-1654.46	-4520.76	-10426	-13069.3	-6911.42	-6103.71	-11820.5	-4130.2
-3719.61	-5563.38	-8286.04	-13402.8	-9475.53	-7504.4	-2566.96	-521.477	-8886.84
7196.63	-7341	-8876.56	-6708.62	-7635	-5039.52	-1552.26	-1579.7	-3830.5
-6285.86	-2107.02	-7219.11	-4399.91	-11638.7	3387.1	-3557.76	-8411.02	-4040
622.029	-1681.53	-794.576	-7853.06	4185.36	-4763.01	-5467.75	406.75	-9927.17
11647.5	1736.77	6484.17	510.334	-3840.54	-1179.94	9487.02	5207.93	6611.91
6439.4	1119.43	339.256	-6888.15	9195.14	-1233.04	-3076.2	2435.64	-2291.76
7889.65	-2693.56	1908.92	-8360.73	12978.2	1844.54	0.205078	4474.45	-4600.77
4044.16	1617.46	-6827.66	1246.13	37839.3	422.336	-3812.66	16285.1	-5026.96
224.93	-502.043	42185	100.076	1.03E+06	9227.38	60178.3	185174	-2668.52
9300.8	-3514.66	38076.4	-6663.87	624739	27670.9	17375.8	648824	1106.62
12493.9	2250.85	85632.6	7667.02	2.13E+06	60532.4	113164	1.11E+06	8410.64
7775.71	6096.43	11725.1	-4686.26	69196.4	18114.9	6003.2	52535.9	-6321.78
11024.9	4001.06	2558.01	-3439.73	34720.4	3340.93	563.939	43729.6	7167.42
20264.3	3960.25	9047.53	2328.22	213365	12676.1	13327	228424	6203.02
40778.7	15541.6	6411.97	6944.05	14634.8	7460.07	11762	27865.2	7916.46
10872.2	5007.02	9479.53	-8164.14	25531.1	-1165.02	-4667.34	39621.1	-2143.65
2695.81	472.314	5355.89	21.6602	37175	3766.41	-2088.74	23666.4	-1354.85
-2112.95	-3096.11	-4442.07	-7270.46	44905.7	-3316.18	-1185.5	75074.6	-1222

3014.86	1147.54	-105.828	-1356.12	7163.9	2514.51	1256.16	26007.5	12228.3
8197.52	-4225.71	-1637.38	-5135.15	1877.57	1158.2	5306.1	9608.16	3560.55
16051	4378.3	4169.06	-1259.97	712.436	5477.84	11305.8	18616.2	15544.4
9851.59	244.693	1482.36	-5471.47	5869.37	-4399.2	974.971	10444	-501.223
14788.4	1913.29	1130.05	11291.4	1211.73	-2255.8	-446.506	11008.2	3363.95
8873.46	-1546.23	4683.18	-147.328	872.008	3832.82	3821.66	16844.7	4538.41
13505.3	-281.162	1496.8	-2657.7	5569.85	4524.57	17311.3	20983.7	13682.6
8571.74	1278.65	9950.14	4848.35	23526.3	-687.549	12174.6	37077.6	6386.61
30165	17234	61451.7	21308.5	825350	23787.3	60553.5	891150	35764.6
851512	339638	427155	625546	162699	120075	651287	1.07E+06	914999
259716	457670	395842	136377	66368	645281	251609	717171	301844
43334.6	23604.8	56213.6	13371	840886	108634	82413.1	968522	27189.3
13693.2	8552.96	6416.26	-6706.9	54299.8	47674.4	9067.22	76951.8	7975.67
13240.3	11217.1	8024.01	599.816	42262.6	35761.5	9507.5	75809	6092.44
8479.36	7420.09	15023.3	2836.63	225503	23361	14889.1	245247	573.693
-2028.63	3350.44	4488.27	-6849.8	43429.3	14364.8	3431.42	54244.6	-4947.33
5056.61	-4915.14	11646.2	-4738.1	443276	18142.1	8688.59	261296	-1023.08
17687.5	15353.3	103586	31435.6	1.98E+06	72681	122470	2.34E+06	6792.22
7097.92	7438.1	16359.7	-4843.97	62271.2	15374.9	7648.27	65315.3	-2001.95
12251.6	8259.92	6377.46	-471.418	23512.4	8152.64	12449.5	16962.7	11611.9
4432.89	9511.73	-1536.1	383.814	8335.68	8578.82	8465.88	22460.4	5642.04
2792.91	-1316.85	-3095.52	-14794.7	51552.6	-1485.44	-4885.57	49322.1	-6086.2
-3666.98	-6790.08	3938.51	-3902.39	62616.1	1569.67	-2605.27	52135.2	-13705.7
9502.67	-3135.85	88852.5	14712.1	2.35E+06	26125.6	110448	2.35E+06	-4129.09
-7733.37	-2533.42	38444.1	-8667.8	115292	36476.2	10377.4	251382	-7374.42
4319.17	3172.57	109.629	-9790.98	21978.3	3647.69	4229.17	24813	-4434.81
1718.3	-753.937	297.035	-11752.4	1494.02	-4433.73	405.336	-2767.2	-14.4707
-2352.91	-4225.04	-2566.67	-9108.22	-3499.38	-4679.34	-6985.46	-8439.68	-9686.51
-3429.31	-5545.71	-7244.35	-8629.66	-13664.5	562.973	1902.75	-6767.16	-3113.36
1912.72	-1260.05	108.91	-6645.15	-6251.94	-3993.76	311.885	-2833.7	-10893.9
-640.898	-8445.54	1366.28	-1648.03	-10322.2	-4984.95	-6991.82	-13163.5	-1833.4
2225.44	-1692.55	1879.93	-9194.96	-3682.45	-247.586	1640.96	-6703.66	-6219.68
-2708.34	-530.232	-5695.22	-3594.97	-8458.26	-3044.35	-4232.28	-3658.63	-13281.3

-1899.01	-4512.15	-5757.09	-4749.66	55589.4	2278.32	-4568.95	56251.3	-10514.9
5448.95	-767.893	-4391.27	-9582.69	2541.79	-4108.1	1959.1	5378.39	-4889.85
3332.27	4882.06	-1416.13	-5857.58	-6935.69	701.707	1041.27	-8158.04	-559.193
-3494.11	-1478.22	-7546.16	-6534.74	-12240.1	-7836.87	508.58	-3449.25	-6956.12
-4724.08	-2415.29	-5772.94	-9648.53	-9665.32	-5735.56	2694.66	-9146.24	-6625.46
623.266	2404.92	-6092	-11435.8	-5390.43	-7084.56	2044.64	-8922.82	-4498.25
-4133.3	-8908.13	996.539	-10409.6	-5889.96	-6519.09	1280.75	-3855.61	-1720.96
1617.79	-2825.12	2193.3	-4754.91	-10977	-6105.95	2445.91	-5570.71	-474.072
15140.3	8656.21	10966.3	8152.27	6566.96	764.609	11232.9	7178.59	9917.46
-4293.87	-1852.88	-3003.96	-4873.54	-10529.2	-2004.95	-2467.71	-15199	-4552.62
-960.783	-4095.28	3374.04	-11427.6	-9506.56	-4731.88	5932.36	-7731.07	-5180.08
-1550.77	-7574.04	3514.94	-9773.99	-4558.28	-5508.91	-507.303	-846.268	551.143
2470.37	-2907	-5119.95	-1940.51	14057.8	-4960.03	780.561	19818.2	86.5938
867.697	-3212.68	-1131.17	-7941.09	-5845.13	-7442.75	-282.031	-6249.72	-7293
12689.2	3526.27	-1134.18	-8447.07	-15283.6	-2204.1	1542.44	4571.62	3982.62
11469.3	-6349.13	-2838.92	-7874.52	-5378.73	-5220.18	-1306.13	-5956.27	-787.471
3521.39	2989.22	-936.373	-3902.78	-9456.9	-5510.4	-6723.42	-10153.1	-7171.53
9534.98	3002.76	8977.56	-1828.65	-9282.39	-697.963	-133.375	-8209.78	-5533.04
11782.5	-4081.24	7330.86	-3645.26	-4126.6	26395.3	5035.16	10403.9	4930.88
8422.25	7551.86	18913.9	445.029	-6108.65	19324.6	-2606.97	-6894.16	1657.53
14251.5	485.514	33333.1	-5499.7	-8110.66	1452.17	5611.1	-3054.83	36198.7
1575.85	-4391.13	4875.66	3659.37	-4599.44	1147.05	17705.2	676	-6794.46
46444.4	-6008.08	5410.6	-2183.96	-9293.8	-2243.95	13633.6	3784.67	-6371.24
529.371	-741.408	1966.93	8587.13	-5509.22	-6144.63	157.574	3987.11	-7862.73
1991.23	-1243.88	2024.71	-7585.67	-355.012	-7491.61	-3688.56	4444.27	1241.55
519.797	2592.04	-1658.02	-2517.96	34541.2	2691.58	2973.71	41114.6	-6964.73
-865.191	48224.4	28598.1	62965.6	1.42E+06	-8177.86	8540.03	246084	-4813.27
-444.186	1358.72	120988	21447	1.05E+06	66574.2	112162	2.33E+06	-228.154
4078.51	-2203.2	8648.51	-3073.1	52103.7	5791.88	3266.51	47118.1	308.785
14373.2	5442.76	4398.53	-1354.81	31930.9	1998.19	10432.4	23079	5738.68
17335.8	10262.8	2640.27	4469.95	7480.09	14530.4	8260.85	59312.5	8167.32
25723.7	2866.7	3752.85	-1450.28	5314.35	5658.47	17203.5	20805.9	12960.5
6763.49	-759.949	-145.219	181.133	5710.89	7307.26	3965.1	9826.29	10804.5

5999.43	4426.26	265.641	-2736.5	23971	4429.51	2284.86	27301.3	-1063.9
5497.16	3165.64	-698.391	-9308.37	-7424.57	205.514	-1420.81	3121.26	1891.04
6378.4	512.979	355.738	-5532.15	-573.457	-2788.01	2988.2	4717.54	-891.926
23951.3	-733.662	5954.04	1863.58	7944.58	654.633	3039.3	18790.2	12950.4
10361.8	2816.47	12418.4	-3948.77	6955.82	1342.58	2209.45	19647.6	10895.7
27031	11947.8	5614.4	1661.39	-560.422	8357.46	8023.44	19569.2	16714.8
3264.51	4316.35	5577.93	-3761.04	217.961	-5274.8	6254.57	6510.13	5614.02
6658.17	7491.76	3520.33	-1178.88	2726.86	4676.18	4965.69	12957.6	2215.64
4744.47	4069.18	5938.75	-689.148	40350.1	3662.75	5952.99	39530.9	13977.3
24372.1	-1966.57	-2240.18	233.398	70164.1	93.6074	7499	84248.3	-4313.13
14852	3427.8	74438	23099	2.41E+06	5009.73	96341.5	2.45E+06	1785.65
21947.8	12500	65107.1	9331.49	131331	55194.5	32991.8	290294	12661.7
13182	5242.19	2121.32	641.754	65050	9101.58	9155.2	64341.7	622.945
14858.1	3431.8	15216.2	-574.186	106609	3593.5	13095.5	69202.2	3976.95
17571.5	1337.57	60327.6	5979.99	2.30E+06	13969.7	44943	1.04E+06	8199.42
21248.2	21995.3	175432	30828.8	3.82E+06	99978.5	203828	2.14E+06	15813
12419.5	6624.74	92920	6609.11	303764	84567.1	81980.6	1.48E+06	-367.992
4385.24	832.439	4569.56	-326.289	102085	26313.7	4997.97	117118	-1966.63
9806.03	2769.57	6728.53	1265.51	19659	8358.14	6192.57	12058.2	21866.3
33203.6	14635.1	523.541	95.8457	3915.35	5870.38	18677.5	20584.1	12458.4
10923.4	1963.88	4066.4	-2404.34	2004.49	7033.57	1071.05	9402.32	1987.15
13146.3	3180.12	8108.68	341.488	1830.88	4913.8	4143.41	7732.16	-4414.34
12120.3	11802.8	7652.23	6702.11	18778.6	8030.4	5787.72	26443.7	17966.7
26455.6	8181.42	2326.42	6841.78	86255.4	4468.83	13999.5	98034.8	4572
3813.75	3622.47	905.609	-525.27	90.4258	9991.16	3222.46	10790.5	12214.5
22356.7	7794.67	12043.2	-2334.06	15706.3	12424.9	7266	33153.9	9027
27191.2	4460.16	12606.2	-990.719	-2153.19	1113.85	21968.5	46496.6	5988.64
22875.8	5411.78	10665.2	3701.94	4754.46	5366.23	11411.8	73604.5	16752.2
19712.6	4190.36	9018.74	-4939.97	11688.9	3400.09	4986.42	95112.4	3538.65
36061.2	32860.4	17097.9	16980.1	34736.6	21942.4	29939.4	52367.7	38889.4
62624.9	33558.5	20274.6	28404.4	80370.8	32109.9	42104.6	129792	48090.1
12912.4	2924.04	11349.4	-290.555	1.15E+06	18008.9	10190.5	157158	6551.97
14487.3	13844.1	63210.7	15017	315438	40372.5	77116.3	615217	22356.4

91820.2	72290.9	45082.9	48233	1.35E+06	48936.1	63397.7	468760	85340.4
21097	8452.16	50217.9	1472	123399	45243	74916.1	503840	4114.99
3864.72	1678.26	-656.822	-3857.53	18152.3	15487.8	1731.69	25785.9	4928.94
9149.15	-2621.29	6241.61	-2269.67	1275.43	10993.6	4702.6	2806.67	4963.67
23910.3	17810.2	8868.46	5061.78	7996.67	5745.63	9671.7	24499	25243.4
45049.3	24584.3	37494.9	28257.7	16325.6	25837.7	40586.2	78806.8	36695.6
488394	348324	282669	335278	51454.8	244574	403375	771179	545190
32306.8	26399.7	77633.6	14572.1	25803.7	119624	23029.3	49424.8	25886.4
27959.5	25217.6	22620	12461.1	-10417.7	48500.6	20470.1	13975.2	24618.5
10042.4	8981.46	7840.45	10885.4	41752.6	24442.8	4695.1	21314	7051
68266.7	33789.6	19505.8	33806.4	4327.22	67290	38299.4	86278.5	52177.2
10070.6	78867.7	9125.37	76446	18251	62089	5513.42	18240.3	17772.7
34880.6	31633.3	31525.1	9468.06	52616.7	21633.4	15787.2	47729.5	69109.6
21266.9	5954.63	44617.1	6484.1	1.41E+06	20833.5	81815.2	636105	8326.21
76238.6	13843.7	32774.9	-687.598	85984.7	43573	37215.3	113564	11289.5
23574.7	9979.81	9482.76	-5901.81	30060.7	12440.5	8057.36	59750.6	7858.45
14839.5	3755.82	9287.74	-392.582	12660.8	6191.29	10518.1	23621.2	4669.36
28917.5	7760.66	2686.8	3850.46	-571.785	9554.65	8135.98	36505.8	16245.6
9783.33	-1900.96	-2582.01	3712.62	-10967.3	8230.49	8759.09	808.369	-626.639
5556.34	-213.344	-2118.46	1175.36	-1805.01	8328.09	-5587.95	-5357.98	-8533.73
7018.27	6227.59	741.02	-4524.57	-3430.1	2717.21	-5036.21	801.258	4220.79
4896.52	2993.47	2217.3	-2712.29	5951.83	-2406.53	1310.13	19993.3	3604.3
32976	6681.04	5398.45	2455.41	2741.09	3502.98	2722.86	8447.21	11885.5
7065.46	5218.78	-967.812	-9637.96	-7019.96	-5669.82	967.865	5333.41	-401.715
-2289.99	-1162.03	-4826.92	-4313.32	-9094.79	-3923.99	-2728.57	-2479.62	-10263.4
-3621.24	-6708.42	-6680.9	-19807.6	22790.6	-9447.63	-10190.8	-4442.68	-16227.6
6538.14	2821.43	1877.05	-6287.72	82224.6	-4476.44	4917.15	27868.3	3442.71
6763.46	5118.18	61076.4	6517.9	2.23E+06	9371.15	104051	1.09E+06	4318.26
7162.38	5103.75	41337.2	199.248	81256.7	49775.5	11753.8	78166.7	-860.174
28223.7	13805.8	15403.1	9721	99834	21390.6	27067.5	86435.9	16049.2
4974.91	6872.75	37581.1	8735.08	824440	30019.1	48166.2	881081	5250.02
5048.21	-585.605	1341.08	-4769.47	19343.8	475.436	1090.2	28228.8	7671.44
8151.01	-4622.17	-2281.93	1088.91	5976.28	-489.656	111.977	7504.81	-834.787

26398.7	-5591.21	-2344.3	-6263.9	5731.57	169.937	14699.1	8173.76	2666.25
4662.25	-4985.68	-3804.37	-1968.42	-2384.09	7194.48	-2215.56	-1076.17	-6214.21
3115.05	-463.561	-3454.12	-2285.07	7698.05	-5357.45	186.49	-827.219	-3161.5
11200.2	3736.69	1185.97	-4827.69	22491.9	-7012.42	-4924.25	31396.8	7530.94
11047.7	1521.2	563.402	-3131.71	8370.34	-5017.72	-4625.24	-230.582	2244.28
11711.8	1800.37	3158.7	-4065.14	23603.5	-457.795	10747.4	20410.4	3464.95
3966.79	3052.77	38451.8	5015.51	806441	11503.6	42176.5	827351	4587.35
11846	-3014.7	11482.5	-2902.14	38115.6	-4153.95	4930.07	75376.6	2337.77
14648.5	-3288.41	5640.42	-2602.44	45400.2	-6387.67	2148.87	28200.6	1492.93
16889.6	421.135	8399.61	-1473.44	109694	2620.42	3085.48	66490.6	8122.08
13295	5702.68	50923.2	-2394.01	2.65E+06	15194.1	44557.3	991898	4728.4
39114.4	15683.9	144685	17481.7	2.00E+06	113042	201289	1.34E+06	19006.2
8992.84	1078.24	31166	-8106.35	281762	26416.6	13663	232679	7627.75
14654.9	4800.76	3543.17	-6399.62	19527.4	13695.8	11427.7	47771.8	-2516.65
-624.064	5311.82	1400.26	-2977.28	-3756.12	6918.79	2646.8	-1388.08	1748.85
352.859	2160.49	432.701	-12764.7	-2530.97	556.623	3983.63	-9993.48	-805.803
3404.14	1517.15	-1989.11	-5671.47	-8390.46	-1727.6	-3623.1	-10461.4	-1453.29
3075.83	2212.79	-2890.53	-5311.15	-3389.56	-3984.75	159.705	-184.521	-3477.5
5426.43	-4554.82	-2548.05	-8642.42	-8874.67	1458.67	5755.87	-6876.54	-232.27
3959.75	8036.44	-5421.01	458.176	-13651.6	-4308.65	268.172	-3253.92	1562.14
4732.27	3360.68	-4502.39	-7613.48	-7236.45	-3164.34	-4030.3	-9056.53	3507.28
-5099.1	367.887	7106.92	-7911.56	-10401.3	-548.959	2822.34	-11039.3	1012.68
4807.76	1326.44	-4694.15	-6330.15	-3781.24	1263.93	-2418.12	-9034.13	-54.834
2008.63	327.699	1098.5	-7318.43	-16801.9	1992.92	-1296.8	-7353.51	-362.977
538.545	-3976.03	-3162.44	-4446.61	-9891.12	-548.346	-1879.66	-10423.7	-4862.08
4445.28	1346.49	-3879.87	1947.67	-16902.7	346.912	-1292.91	-12448.4	798.271
-751.037	1318.33	-1822.05	-6125.61	-11522.5	-13162.6	593.195	-9960.92	901.723
6692.88	-1790.66	-5143.59	-6367.97	-6578.63	-10068.5	4823.92	-9737.86	-9090.04
6295.44	-290.334	-2651.33	-1825.02	-14503.3	-3462.84	-2456.18	-2494.55	-8538.99
1899.91	1151.82	4155.83	-3301.22	-6555.09	-2264.72	1651.59	-13618.2	1874.12
9596.84	3631.93	-5368.17	-2145.48	-583.293	-7448.08	2100.71	-7062.35	-3486.11
305.047	-3336.21	-3201.27	-6510.07	-10142.9	-8168.47	-631.646	1868.34	750.125
2973.66	-3967.27	-3602.12	-7248.36	-8535.18	-6726.02	5255	-1918.53	-8456.57

2688.73	3897.66	-1170.13	-3717.48	-8029.49	-3248.3	-1457.01	-14838.5	-5231.02
-350.631	-227.717	719.953	-8717.77	-14146	-3749.47	-1590.19	-8135.46	-4676.3
580.982	-1562.45	2983.28	-9274.92	-9704.09	2005.77	-4606.18	-14975.2	-2651.06
-3259.01	-1819.1	700.797	-4997.38	-10300.3	-9524.02	1331.91	-12439.5	-1111.56
3290.64	-5113.96	-3010.9	-8167.6	-15665.9	-2729.79	3226.01	-11778.1	-2370.34
955.537	-9410.69	-4013.4	-4955.65	-7308.02	-6248.11	1033.57	-16726.9	-3675.58
2914.08	-69.8457	-2876.81	-1295.25	-6942.94	-4422.81	-712.463	-7579.43	-8371.52
3318.19	3923.22	-2186.85	-2384.32	-13562.4	-4225.51	6073.01	-4766.4	-5602.43
-1762.74	3563.07	-2605.97	-9895.44	-9874.74	-1735.29	5473.69	-3633.76	-224.107
784.428	-2685.39	-996.805	-5429.81	-7193	-7467.68	-8741.41	-11362.1	1935.13
2985.73	311.785	1415.8	-7152.4	-12769.2	-4400.49	-7134.45	-8480.78	-2899.6
7514.39	-3311.65	2014.02	-4188.99	-14935.6	2048.07	223.377	-9259.15	-845.303
8440.35	1949.96	3048.79	-7083.36	-12003	-4809.97	-3138.08	-12645.4	-3896.11
8984.44	794.377	-2439.22	-4416.16	-1311.92	-9132.33	-1102.12	-9138.77	-6648.63
318.729	-3405.76	1894.12	-5950.91	-14156.4	-3819.13	-4079.85	-9960.61	-5190.52
-3338.04	-3821.09	-695.674	-4500	-6639.98	-3316.32	-4435.28	-7226.87	-6289.99
1004.52	2099.69	237.814	-2614.88	-5451.36	-3112.17	1868.15	-2229.33	-3075.07
-4204.71	1332.96	1230.14	-7489.56	-12295.8	1281.17	1316.96	-8486.5	-6200.07
4010.84	3081.88	-3438.04	-5755.84	-9834.73	-6587.61	3745.6	-3254.19	-7128.99
-1710.42	580.992	-3732.39	-3429.37	-14310.7	-8454.1	5286.97	-10793	2747.6
1595.49	4756.77	-683.996	-3094.03	-9744.96	-8313.05	2765.96	-5271.5	-4339.8
3389.07	-3779.03	1028.83	-7860.18	-5379.45	-7970.49	4712.06	-2432.98	-4803.9
3066.13	-1914.89	9811.38	-8085.24	-8876.05	-3739.67	2606.7	-5252.04	-8955.67
-309.213	2191.9	7458.65	3569.54	440.369	390.533	-187.871	760.697	-3100.32
5835.86	-3543.58	285.65	-5358.9	15667.7	-6153.58	5526.76	17647.8	1125.78
2507.59	28.2598	6789.39	-6845.37	502239	-3423.87	-6811.31	106959	-2253.37
7812.41	1950.8	40455.3	-4555.83	352572	19168.3	37483	800814	-8161.36
2989.59	1091.52	6964.16	-4511.07	11888.3	-2736.46	-3711.27	11259.4	1560.93
-3460.54	-435.342	-5488.05	-8043.47	-2391.59	-2822.18	-3044.38	-5483.15	419.813
3503.1	-1398.82	-4174.89	1108.37	-8521.77	-5699.17	-362.477	1145.19	-3170.67
-1659.64	-7009.56	3961.48	-8271.18	22900.6	-3518.37	-650.986	19138.5	-4150.29
6442.15	6685.93	2692.95	-6835.32	195166	2415.3	10245.8	207996	5072.48
5453.4	-503.805	1356.26	-1562.04	-4623.36	8221.74	-1130.41	-6605.9	404.49

5819.24	4399.92	4871.73	-4381.94	-5723.66	1896.17	-1249.75	-6417.77	3046.85
6367.16	7483.96	-2335.49	-7710.89	-11870.7	2162.41	2061.01	-6045.75	-5625.88
7164.94	-2058.75	-2417.87	-10970.5	-10961.6	-3094.59	-2514.76	-12429.1	-4784.36
2543.14	-6501.02	-936.484	-5909	-3055.19	2476.45	-13173.5	-10985.1	-5880.51
3200.67	4102.86	-7162.27	-8186.03	-7741.76	-332.957	-743.727	-11596.9	-1197.82
7554.72	710.785	1417.64	-3879.28	-13.0898	-5390.39	-4008.15	-2208.53	225.291
1819.88	-4580.22	1834.2	-5575.86	73455.2	-1817.06	-5026.94	-614.715	-4393.31
7495.67	4352.54	13198.3	-2813.36	134858	1728.79	6691.77	215689	-2963.59
-2456.47	-1188.17	6899.97	-6035.6	-8660.52	3902.83	428.412	-6888.08	-6666.97
3769.43	-554.955	-4076.21	-9436.23	-12361.6	-4165.03	-4291.87	-15135.1	-1994.55
5414.82	-6791.03	-4046.55	-3820.95	-16372.1	3039.09	-5025.02	-17708.6	-8727.22
4876.88	1300.69	4342.47	-4582.29	-1311.35	-9778.64	-4250.72	-8209.02	-4666.72
3476.08	-1858.28	-3072.79	-2199.65	-14049.7	-5919.65	-5034.94	-13950.4	-2206.43
116.086	5712.03	-151.418	-5624.14	-11854.4	-3095.76	3637.41	-10218.4	-9264.92
3683.45	-3197.24	-3463.73	-6118.77	-5092.87	-3162.64	2717.02	-8247.84	-877.365
3081.02	-135.037	1161.45	-14333.4	-6884.98	-8483.97	-1303.13	-8202.63	-3387.91
3916.57	1215.33	-5983.17	-12654.6	-7340.63	98.6094	-897.852	-8765.23	435.17
1214.88	-3915.53	1934.32	-7975.77	-11461.3	-2204.04	1841.3	-6762.1	-7114.22
2214.51	-2803.22	-1590.89	-7613.2	-8130.43	-3729.99	-404.553	-6972.01	-3127.47
3105.38	-413.164	-9404.87	-5392.1	-15002.9	1531.78	8831.66	-10864.9	-4655.66
2268.78	-2301.5	-961.457	1611.74	-11226.9	-1151.71	2480.1	-9648.52	-1889.3
1680.4	3222.88	-808.746	902.268	-16426	-6157.65	3413.94	-15989	-6582.88
5287.08	-2818.21	2418.01	-5476.44	-11637.2	-7721.98	6788.74	-6219.98	2009.08
2851.04	1819.02	-2038.88	-2076.4	-14877	-11700.1	-451.818	-11997.7	-6320.71
4525.68	-141.525	-2343.44	-2509.43	-11631.1	-9561.09	2881.62	-14116.3	-2728.91
-2170.56	-7646.45	-3505.91	-2792.11	-16536.2	-7295.65	4091.48	-10327.7	558.059
-3052.29	-819.156	941.699	-12255	-9712.5	-5161.75	-242.52	-12087.9	-4872.66
5379.52	2873.92	-2385.12	-10718.4	-11096	896.178	-4990.17	-9159.03	-4964.04
9333.79	1551.18	-475.164	-7991.67	-9755.42	-7993.45	-3683.53	-5763.85	-7030.76
7898.36	274.031	8026.57	-2452.83	-14417.1	-6901.59	-2081.36	-10490.2	-9186.47
2071.88	-658.039	-1458.01	-6844.45	-13259.2	-8190.91	3700.16	-9539.27	-5374.6
668.174	805.648	-3629.31	-1148.89	-8388.6	-1111.21	3514.31	-8891.56	-6472.86
5635.53	-4031.96	-2041.88	258.562	-10122.6	-5811.72	659.801	-12973	-1194.26

6071.3	581.691	-2044.04	-365.021	-13783.7	3049.77	6221.18	-9515.08	-3390.4
3304.07	2469.74	-2070.18	-7348.35	-10037.5	-1645.21	-937.426	-10883.9	-3658.6
770.098	100.135	947.965	-12862.9	-8513.22	-7310.16	-524.85	-6531.94	651.309
-1874.95	3322.73	528.168	-2654.06	-7615.04	-13417.4	-3970.86	-16991.6	-9302.38
5357.96	1697.64	510.674	-6132.62	-4689.45	-5698.14	-1982.69	-12880.1	-5093.99
6698.87	-4847.97	-1930.81	-4921.19	-6833.36	-9362.96	1291.59	-9707.46	-5263.57
6434.49	1806.88	659.791	-4612.22	-11645.2	-1936.49	937.527	-10037.4	-6870.96
6390.63	-1929.57	7902.07	-2428.02	-12149.8	-172.074	-4098.33	-13206.5	-5815.9
-426.611	-358.209	3544.29	-4322.38	-8962.38	-5031.24	-4825.86	-9085.15	-6422.67
389.551	117.727	-6181.91	-8673.68	-8327.77	-11083.3	3865.87	-14576.5	-5948.37
4404.51	-866.979	-1337.53	-3075.43	-7016.38	-2298.04	-4996.97	-11976.8	-3180.08
-2966.89	-4468.63	-3068.44	-9516.51	-8210.83	-7319.76	1813.93	-9028.62	2451.04
-3029.28	1460.21	5016.58	-2647.44	-8921.32	-7664.96	2143.62	-9824.08	-5232.75
-5593	-4381.01	-2265.81	-320.721	-11493.9	3452.13	-2251.64	-4686.13	-12474.2
2886.53	-4126.23	-3527.4	-4066.13	-18439.9	-6767.92	1907.97	-8388.88	-8232.51
-4322.23	-3181.47	1955.47	-4795.3	-14581.4	-3992.42	-4715.51	-13228.5	-2946.99
223.045	-1927.28	4633.88	-3574.74	-7855.88	1823.38	-10674.2	-5934.99	-7325.72
4829.25	-1732.52	-2331.11	-11819.1	-8784.86	3959.06	-1417.63	-13478.2	222.629
9912.59	-1948.03	1792.66	-10666.3	-19152.5	-2202.93	-2768.17	-8420.27	1102.78
-1158.24	-585.746	694.711	-11647.6	-9922.2	-6093.84	-1791.52	-8859.05	-2582.5
778.869	892.625	-2029.44	-9081.72	-16419.3	-3764.89	764.559	-5645.21	-4559.78
220.605	2343.45	-9625.43	-4576.09	-10580.5	-10733.6	-4041.02	-11223.8	-235.244
-765.707	1758.72	-808.342	-9845.57	-11024	-7938.13	1935.21	-11195.5	4025.65
3000.43	1150.14	-3002.59	-7487.13	-14886	-7023.86	2711.96	-14229.7	-8974.14
6561.43	4993.84	-3097.12	-9000.42	-7354.1	-5502.94	-2547.81	-18645.2	-6396.91
-3840.1	508.643	4272.65	-2183.26	-11600.6	-5550.54	4098.03	-14585.4	-6870.79
9496.03	2369.21	1795.73	-4520.8	-10105.2	-2776.51	-2823.56	-17661.6	-2107.65
3729.4	-1223.38	4821.85	-8980.72	-15917.4	-4213.92	1644.85	-11930.6	-2516.34
6623.33	4955.53	1169.46	-5195.45	-10128	-7508.73	2471.14	-3318.72	-4400.23
1545.12	5415.89	-856.357	-8357.06	-8120.73	-6219.67	645.467	-4299.63	-5258.21
-4957.46	7964.82	-4799.7	-7429.69	-12724	-5742.64	-8277.68	-4556.62	-6012.05
-3513.01	6470.03	-2318.87	-6461.86	-5259.5	-9979.37	-737.826	-7137.56	-4228.57
-1280.51	1742.19	-618.465	-7487.17	-12065.9	-3165.01	-969.738	-3096.74	-3078.11

2156.2	6215.14	-1917.76	1279.08	-9356.63	-7205.36	-6659.13	-9191.18	-6005.48
772.629	1217.32	2088.55	173.027	-13674.9	-6748.7	2948.47	-3636.89	-5174.89
-862.998	86.0684	5046.83	-3955.08	-10467	5474.88	-1734.46	-3153.71	-12051.6
-1685.65	-2932.03	-2381.96	-13771.2	-7774.85	143.488	-4031.1	-5287.15	-2205.15
-442.764	-7158.15	-2402.84	-6941.61	-10732.7	-801.563	-9541.17	-1711.4	-6100.66
5369.39	-6519.48	-74.1191	-9496.1	-11070.2	-2573.26	-3397.3	-6876.14	-7701.1
5559.12	-1365.82	7332.53	-8339.76	-21611.7	-7934.03	-4300.21	-14641.7	-7540.27
6958.44	3266.6	3997.89	-3809.28	-20950.3	-1136.14	1342.26	-13474.2	-6633.24
5126.99	2996.58	-6752.54	-10084.9	-10066.1	-716.57	-1672.18	-8229.23	-8787.12
1499.87	1218.24	6800.33	-1994.92	-13653.9	-12137.7	4237.48	-10388.6	-4611.42
-152.467	2098.4	-2201.02	-1195.83	-12768.8	-1810.6	1479.7	-13376.8	-2402.58
-4889.17	1983.85	682.854	-2653.15	-11618.2	-3479.43	1349.67	-11042.4	-4098.07
5817.2	3335.62	-2585.56	-12465.4	-11309.5	-4815.09	6084.94	-3839.56	-5831.12
4688.6	625.572	-1964.07	-6431.3	-9072.25	-1933.71	-1335.54	-11300.7	-3545.01
4534.97	-2486.05	3080.7	-10173.4	-17655.1	-1904.86	-5157.77	-11070.8	-5548.95
-446.779	1927	10178.5	-9200.63	-5632.32	-11472.3	-7142.73	-13933	-6752.16
-932.461	-1282.28	-3853.86	-1455.25	-15779.5	-6356.42	-2414.26	-15450.7	-5169.28
3318.78	-2185.44	-5506.21	-11056.5	-5240.02	-14693.5	-7366.43	-20034.2	-4334.16
-4920.27	4855.97	-1292.91	-8630.3	-7648.99	-7323.5	-11254	-10761.5	-3378.57
-3102.45	7326.62	636.031	-5414.55	-11719.8	-4434.84	-5956.94	-8248.24	-10913.3
5567.15	683.715	-1847.29	-9451.2	-10238.4	-3660.09	-4106.73	-6853.06	-8894.24
-1914.85	4172.83	-100.934	-3325.33	-8243.73	-8161.39	-4353.38	-6955.91	1630.04
4043.02	3508.76	477.818	-5630.41	-12874.3	-4182.97	-1952.16	-7019.73	-8936.33
-39.4238	6915.34	5120.31	-1941.28	-10410.9	-5984.66	-1596.31	-12224.2	-4948.79
449.439	1763.39	1274.86	-1466.99	-13680.9	-3723.55	-2723.53	-13864.2	-8622.59
3811.35	-2092.25	-3073.96	-5771.77	-14035.5	-4199.88	-2493.13	-6351.27	-943.082
-4865.33	4575.49	3452.94	-3813.94	-10208.4	-10500.8	-4912.26	-14918.4	-7758.93
5968.88	4314	-3076.61	-5120.8	-10367.1	-3496.4	-5630.62	-15427.6	-8986.38
-203.893	-1288.07	-6485.27	-5852.93	-10625.7	-8847.51	-1749.69	-6009.89	21.5
1185.98	782.912	-4154.97	-7886.86	-13907	-10144.7	-997.605	-8202.49	-3148.57
-1959.91	-2192.15	-49.168	-5212.5	-11097.4	2731.04	-5284.68	-5912.98	-1496.62
445.27	2319.62	-3602.38	-823.105	-5075.96	-1457.23	-3355.32	-15336.9	-4780.07
1402.18	-2033.77	2838.63	-5578.46	-9704.38	-6379.94	-3694.8	-16019.2	-2827.66

2639.65	2718.4	4798.49	-1583.01	-10909.2	-8176.22	-3580.65	-3373.59	-8323.65
6526.19	-3198.64	-1303.48	-2985.77	-9625.69	-7995.65	-4777.51	-4612.43	-4405.59
7672.31	-2304.46	4014.78	-1001.64	-19036.4	3445.52	-2757.36	-10960	-3625.56
8156.01	-6334.78	-8465.48	-1068.61	-10100.9	-8608.2	1347.08	-11283.1	-8829.77
3187.42	-5323.6	-3700.43	-3062.63	-8457.54	-6885.03	-4228.29	-5572.36	-7305.46
-52.5508	-3832.42	750.742	-3255.87	219.488	63.9687	1219.8	727.07	-8032.63
-231.598	2307.23	4858.59	-3105.17	16561.4	-6811.69	-4504.2	6570.51	-4153.24
4989.49	-2139.91	5177.31	-7428.88	404399	-7738.22	-6816.87	41660.8	-4648.63
740.816	-3165.67	50286.1	-1381.56	1.03E+06	22548.2	71452.4	655387	-3176.3
6967.3	-1909.15	5003.44	-4259.01	15350.7	-59.3184	1544.95	5208.11	1506.97
17196.3	1391.91	9268.29	3896.55	-2764.31	119.498	2956.72	4960.05	7368.86
3202.27	1755.7	4585.24	-2267.17	-10779.8	-6391.65	-3539.91	-989.725	964.016
6844.38	-4636.85	2038	-2355.74	-17187.4	-3686.83	-4166.75	-9900.82	-3498.87
10237.9	2331.09	-773.264	-3325.15	-11074.5	-2557.2	2359.99	-1870.33	4970.1
5595.57	5709.59	11.5781	-4108.25	-10010.6	-4062.84	2232.37	-13835	-7746.45
-1497.33	-4167.71	1593.93	-2697.77	-10705.2	-5927.93	-2022.2	-5556.35	-5877.19
748.137	2987.21	-2493.08	-4015.17	-15859.2	-7538.25	-4913.75	-18666.6	-6374.53
42.5781	4013.14	5318.52	-4753.04	-9775	-7517.33	-2574.5	-10172.2	-2438.45
6249.76	6966.38	-1557.05	-5483.06	-11910.8	-7221.1	-384.441	2395.3	-3126.91
3678.6	-436.125	298.002	-13078.9	-13618	-6451.1	-1618.89	-12804.2	-5199.03
2295.25	-1071.92	34.7539	-6043.81	-7626.5	-7839.12	-1378.08	-12134.3	-7654.76
-4365.71	4259.69	2924.76	-9568.6	-12909	-10725	-548.359	-12956.9	-5071.12
204.02	-4280.86	2615.56	-3293.29	-13949.5	-12778.7	-9654.63	-12945.9	-6298.47
-4321.61	4281.48	-2018.96	-2875.21	-14323.7	-11885.1	-3381.02	-4376.23	-4282.52
-3104.92	3813.18	2309.9	-3590.43	-10084.2	-9004.24	-471.066	-8536.77	-2319.67
4359.54	3262.6	-892.805	-2775.72	-8705.93	-5889.07	-3803.58	-13589.8	3254.21
1551.39	581.721	2294.33	-388.775	-11971.9	-7633.18	610.557	-9416.16	-4947.85
1937.4	3451.23	2052.85	-5707.1	-10732.1	-5658.06	-1431.31	-4576.48	-4050.23
-3909.96	3411.58	5912.79	-10262.7	-7629.46	-5589.25	2481.62	-4942.41	-8034.18
-1801.41	1168.6	-317.984	-6297.87	-8607.33	-3096.69	-649.873	-7826.77	-2267.72
-6637.22	549.418	-564.926	-7832.19	-13284.7	-7272.77	-2521.61	-13639.6	-8821.64
2534.09	3955.53	-1621.1	-11304.6	-8385.3	-10208.3	-9118.64	-1685.28	-2250.98
-276.443	-655.439	-2139.04	-5554.07	-5760.09	-5663.94	-2058.59	-4698.07	-730.193

3223.4	1354.16	-2240.27	-5346.71	-9428.92	-9401.32	-6968.53	-2129.74	-3377.71
3022.86	2760.01	-2795.67	-1711.75	-7653.38	-3464.66	3294.38	-5853.06	-3557.57
4623.4	3285.45	-1196.72	-5716.51	-7843.65	-7491.52	-4989.12	-5926.58	-2221.79
6484.34	4438.57	-4921.69	3035.61	-13431.7	-2137.39	399.807	-7730.4	-10249
901.084	5507.39	-329.684	-6924.62	-9683.47	-6162.33	-5314.55	-3812.41	-8489.81
2567.17	8025.51	-570.361	-4153.92	-12417.1	-5795.22	-1809.47	-10970.4	-5588.49
7224.46	3424.92	4179.34	-9921.45	-9398.22	-9353.13	-7963.12	-3548.71	-1013.64
8025.18	3820.36	2645.54	-11181.7	-9399.99	-4312.11	-3301.57	-24.4727	-4869.06
285.219	3484.04	-4209.34	-8219.23	-8888.18	-11525.7	-1658.93	-7267.02	-7123
-0.755859	-132.855	-2648.3	-6065.59	-2270.46	-2270.02	2600.28	-6093.56	-3510.87
1094.37	-2282.76	2870.95	-8933.35	2738.7	-2996.87	-1721.24	-10025	-1497.21
2831.95	4407.58	3488.46	-2889.73	17668.2	-1906.94	1238.61	11099	-2705.39
3494.55	-3813.37	6558.96	-7042.23	62745.8	-12496.5	2528.67	21912.1	-1219.23
14537.4	3275.85	56067.9	3719	2.23E+06	4438.52	77133.2	1.08E+06	-4098.69
7852.53	3653.46	51824.3	306.139	54854.5	46253	33533.2	57916.4	5312.76
17780.9	7442.04	6638.23	-5270.65	21704.2	-1775.63	5387.04	20459.1	4688.73
10832.5	8613.42	5515.16	2490.16	-6133.38	3891.57	-3945.44	-4402.88	9592.96
5101.73	-1574.59	3385.81	-1418	-3059.39	563.439	5269.7	-3589.94	-773.117
4319.11	-4243.75	2016.8	-37.6523	-12721.6	-6998.72	-1268.01	-10743.5	-5348.69
7682.44	9133.04	737.92	-11234.5	-7364.17	-1626.02	-1680.15	-1481.32	-1382.35
4825.36	428.016	5328.45	-11043	-8443.22	-10385.6	-2699.37	-5433.29	-9579.91
8242.06	1636.79	-4454.89	236.041	-10986.9	-175.76	-4811.15	-6640.29	3500.82
9569.66	3422.23	-262.088	-4879.88	-5826.27	1570.25	3390.61	8432.19	10384.9
3552.95	-3267.49	-1983.29	-4879.32	-6004.03	-4924.13	-3058.66	-7562.48	-7559.99
4142.66	-1734.05	-4673.9	-3859.96	-13053.9	-7277.69	-5220.76	-12782.9	-3852.95
-1921.34	2394.4	-1308.38	-2019.8	-13503.4	-3738.91	-6605.32	-6060.82	-2959.06
1167.94	1978.96	72.4609	-7421.83	-5830.14	-5423.48	-1186.54	-10217.3	2157.36
2861.91	131.402	1871.08	-2460.3	-2257.04	-9024.49	-6652.49	-8391.29	-5306.35
7667.61	761.32	652.064	-347.541	-5443.62	-3571.68	643.305	-5258.04	-3134.49
113.832	-2330.33	-10014.9	-8041.5	-1784.72	-4183.23	-193.307	-9792.2	-7039.65
4840.71	-1494.1	2957.68	1767.02	-4416.81	-3036.83	-5135.03	-4990.3	-2600.18
1875.26	7776.86	-6423.08	2644.91	1367.1	-812.328	-1313.67	5524.27	-1376.5
4308.52	1938.94	-2184.38	3215.1	29993	-8804.51	-5209.64	28336	125.367

11651.4	1299.88	968.086	-3184.33	80125.1	-2970.69	-4352.64	80126.5	-6634.8
4821.59	1445.19	32371.7	6268.73	1.76E+06	-731.316	26945.8	1.80E+06	-724.002
12950.1	4787.76	46126.4	7883.41	30980.1	31085	51916.2	54966.9	-988.219
-649.422	-1207.84	2346.03	-4137.5	13934.4	-3174.72	3129.57	15152.1	-4642.24
2910.93	424.52	-3018.11	-5668.37	-6248.36	-9565.04	971.387	-8953.81	-4997.17
4522.74	7356.74	310.891	-8609.5	-10592.4	-3031.86	-3005.3	-9158.98	-1128.54
-3642.61	750.219	-6822.36	-6569.41	-9187.22	-6469.97	-60.0039	-10821.5	-1549.37
4210.48	522.514	2523.06	-2613.11	-8758.4	-16161.5	-7033	-11918.4	-2710.72
7134.12	-2261.7	4027.6	-5228.01	-10138	569.029	-1390.59	1202.16	3023.33
-655.908	454.389	5669.35	-1958.69	-10538.8	-10681.7	-5236.08	-7140.42	93.8926
1845.55	2196.12	-8139.89	-1191.07	-16493.2	-8332.89	-3022.42	-6215.11	-12287.1
-490.977	-1069.37	-5345.51	-6466.61	-12481.2	-7780.29	-3144.17	-4389.42	-9977.11
4989.98	-3210.47	675.15	-10585	-15718.1	-7336.55	2230.33	-6385.26	-9081.19
-1116.39	-782.352	-603.104	-1228.61	-12317.4	-15369.6	-4240.16	-15837.3	-1922.19
1994.02	-3971.52	-7097.42	-10020.1	-12082.8	-15661.3	-7056.26	-4060.74	-6698.79
2000.04	-4056.54	-8611.67	-5197.95	-18466.1	-10218.9	-60.3437	-9744.21	-2049.85
-202.256	239.939	-588.664	-4249.71	-14057.4	-8096.26	1821.22	-12301.1	-7404.93
521.896	599.787	4395.7	-5841.06	-7743.67	-9744.88	-6529.23	-13090.5	-2918.04
5232.19	1572.32	3977.99	-8646.79	-5956.86	-7090.62	6626.34	-7468.08	-712.451
-5456.58	692.971	-1953.04	-9735.22	-15109.5	-10540.5	-4697.62	-5942.9	-7844.4
2268	-373.145	-1157.58	-5572.26	-9102.95	-8174.76	1009.45	-5864.51	-3180.53
-4139.23	1247.04	718.09	-11119.4	-7606.35	-6761.6	2007.5	-9034.57	-2660.15
1603.59	-181.32	588.578	-3869.13	-10887.8	-13437.4	-2317.32	-6982.61	-2055.01
4729.25	-422.422	-1381.12	-4498.03	-13308.4	-8636.57	-1053.15	-9587.61	-904.793
3238.47	-3127.94	-7020.18	-8556.39	-8709.56	-12067.4	-7009.92	-8608.67	-13042.4
3099.21	-3212.17	-718.086	-5421.84	-14644.5	-264.496	1223.1	-7171.14	-6846.34
4688.6	7096.85	6660.69	-2451.31	6533.82	-5919.94	2060.39	11372.5	-1993.68
-9.83594	1717.95	-420.502	-4241.11	-5548.38	-3101.17	-2740.24	-6574.06	-1470.41
2153.76	2250.32	8289.14	-9672.39	-13900.3	-2249.77	-9354.88	-5363.93	-2993.72
2690.31	-605.184	-3349.81	-2713.45	-12497.2	-2261.38	2897.28	1666.79	-843.504
4344.41	472.203	560.035	-3725.02	-13843.4	-2791.46	656.217	-9051.61	-5050.99
11639.6	5187.01	3065.21	2086.7	-11177.6	-7024.35	-1922.72	-1865.27	-8417.79
7998.25	1605.04	512.582	224.307	-13616.1	-4454.35	2110.16	2510.99	264.756

6585.71	103.547	-4842.08	-5528.49	-14420.8	-6665.02	-3911.95	-8569.14	1068.02
-3016.86	6392.09	1799.03	-4375.67	629.354	-1004.98	-714.934	1432.11	-8372.04
-4764.76	6507.39	6023.55	-10594.7	50046.8	-8923.26	-4906.15	19127.9	-5504.13
4778.29	3159.04	27849.8	4566.94	585192	11195.4	27265.9	637159	-158.355
-387.447	876.824	2951.26	-8185.11	-5121.75	-5048.94	-1729.12	1465.42	-4037.82
587.283	-1296.74	2004.5	495.252	-8150.67	-6801.18	-333.191	10022.2	-3869.1
8578.25	5988.11	1961.65	3946.21	-2608.17	167.48	7510.77	5027.04	1857.9
5615.7	3319.09	4719.02	-1605.42	-6102.92	1666.51	-1004.25	-14348.1	-3012.15
4600.23	-1850	-10196	-5147.97	-10693.9	-4103.36	-1427.32	-5341.48	-6294.73
6565.79	1128.24	-4262.09	-3904.33	-13629.6	-4032.44	-1456.83	-8424.07	-7370.38
4782.31	-603.756	-1651.82	-3749.23	-9299.05	-4567.31	-4405.62	-3916.56	-4226.11
1433.37	2407.29	3195.62	-5358.48	-12692.3	-3804.27	-368.871	-10550.2	-4220.27
235.275	650.908	-1704.8	-4689.68	-5042.73	-6382.37	14.9414	-4585.29	-7929.97
7882.27	2361.23	2257.71	-7766.04	-10444.4	-9044.88	-4320.45	-6423	1367.07
5245.29	1493.43	3626.49	-2212.33	-13042	-3760.38	-3974.72	-9176.29	-3739.12
7711.19	1132.94	5009.98	-7163.96	-9803.74	-3926.33	-1765.51	-6563.6	-5592.57
-1053.07	5803.92	-2527.01	-4798.65	-8221.48	197.613	2716.1	-3576.39	-533.01
7316.16	-2102.65	-1544.03	-1859.27	-10754	-5240.16	2189.93	-12699.9	1965.71
1021.63	-810.178	-2157.47	-2030.45	-7474.04	-5661.66	-2259.12	-12594.8	-5599.59
1911.18	-1159.96	4994.73	-10353.9	-11839.4	-11018.5	-5514.4	-10141.5	-3642.89
-492.398	7087.9	1075.77	-5863.68	-12777.2	-1623.38	-4157.42	-15684.8	-1553.45
2758.01	4917.88	3510.13	-6415.05	-6192.45	-6490.12	-3657.58	-16612	-4537.64
847.713	1154.7	1505.76	462.404	-14617.9	-246.592	-310.268	-13798.1	-3291.28
9472.5	5086.19	-300.625	-7265.7	-5239.16	-6089.86	-6671.06	-7765.46	-1742.92
7849.53	3803.03	2744.79	-6879.8	-8825.39	-6742.96	-8024.94	-10401	-6562.17
3523.67	10285.9	792.178	-6452.73	-2662.85	-6953.35	-3363.17	5555.84	-3268.14
5540.09	1391.1	-1234.52	-10094.7	-9316.38	1723.94	-3233.66	-8629.11	-7256.96
4196.8	418.227	2932.66	-2378.36	-9481.92	-8193.77	-368.779	-3307.32	-4827.38
4523.54	3553.67	-6011.62	-4935.85	-12990.7	-14830.8	-774.619	-12379.4	-1182.98
2051.43	1751.59	2089.39	-5784.24	-9727.15	-13532	-6548.69	-9350.38	-1398.81
-135.541	2471.12	-654.195	-8762.25	-9890.36	-7853.72	333.232	-8234.06	358.523
8965.1	2842.46	3342.96	2016.04	31338.3	-10718.8	-2329.74	3074.98	-5129.96
-237.65	-1502.16	9112.1	2529.03	109521	-3410.82	4581.75	146273	-4632.45

5585.65	2952.45	2938.64	-8257.82	-10745.9	-3814.3	-4630.4	-12096.9	-3736.49
2925.04	-441.551	4002.36	-7142.46	-8577.68	1213.55	-6574.09	-6093.16	3692.05
6244.51	592.207	1270.23	-1122.6	-13024.6	-7158.96	-6226.97	-11610.9	-6938.72
2564.63	-7443.16	-501.686	-5077.52	-6886.31	-12021.6	-7288.02	-9845.19	-3998.7
-3549.83	-2451.29	3703.2	-11996.9	-10684.6	1864.96	-2899.03	-11921	-1813.52
3044.6	2628.01	-119.078	-7110.92	-11400.1	-13040.3	-7967.54	-13422.4	-3823.1
-912.693	1432.94	2493.62	-4908.64	-16373.6	-10824.5	2413.22	-7449.38	232.623
4189.98	-209.512	-6133.07	-61.6523	-9240.61	-8301.32	1811.71	-8685.28	-8114.29
8841.46	3870.82	-3512.44	-6139.1	-15796	-8587.98	-3985.13	-10086.4	-2744.17
2419.99	1064.23	758.895	-8041.89	-12674.2	-4633.58	66.5332	-18673.4	-5552.58
1756.1	4475.12	-1831.73	-9330.46	-12253.3	-8017.27	451.428	-10625	-2278.51
9268.34	6848.14	928.809	-1690.25	-9390.44	-9602.13	882.137	-9425.73	-1551.65
1064.69	2491.7	868.779	-7208.15	-19457.8	-3274.96	-853.551	-15324.2	236.602
1845.14	-5477.72	476.875	-4291.93	-10144.4	-8211.15	-3409.63	-8386.78	-4769
5149.73	-5629.58	-545.883	-2083.95	-11237.8	-9723.4	236.877	-14351.4	-942.424
3004.63	4003.29	-1327.8	1522.5	-10096.8	-3559.01	-357.967	-13796.7	-3287.05
-367.311	-3088.23	-1686.47	-5892.12	-10878.2	-6690.06	-2771.32	-6072.72	-6654.65
2605.62	-4957.93	1729.3	-3984.16	-9841.32	-9402.6	-3544.6	-7168.37	-11175.5
-7772.13	-572.658	1936.14	346.281	-13599.4	-6909.14	-1697.58	-6162.29	-2345.34
-1014	-989.191	5477.29	-10083.2	-16717.6	-6810.84	-2350.78	-9957.7	-2443.77
7007.52	3109.98	-747.797	2260.2	-5275.64	-3412.11	1951.93	-12051	-1506.27
1281.63	5316.07	-3142.66	-4327.81	-9164.59	-10048.5	-5058.14	-10598.5	-1998.04
2056.71	2269.59	-2786.8	-12621.9	-10548.8	-5667.92	-4134.75	-16275.4	-7291.55
4759.21	-4183.79	3736.73	-11210.6	-9658.73	-9319.16	-3449.88	-1855.97	-8484.48
1035.75	7362.87	1305.94	-3534.88	-12094.5	-5392.88	-6651.53	-9292.55	-645.107
3198.88	2958.43	1439.65	2236.17	-9788.75	-15613.4	-6820.09	-9690.91	-3734.45
117.168	-2579.6	-785.549	-3013.19	-11756.9	-2840.96	-4948.59	-4206.47	-7430.7
3648.98	4298.23	7957.74	-7358.92	-6668.15	-12816	-3561.19	-6677.05	-3941.18
4500.75	5964.37	85.7109	-5212.86	-11882.4	-11473.8	917.57	-9550.58	-1347.31
4186.98	3726.29	1272	-2436.55	-20156.3	-7308.03	-824.914	-8668.73	-7947.13
-1612.69	-4963.41	-3520.03	-3628.59	-11914.6	-4119.57	-10144.2	-15384.1	-4861.59
-3075.45	2823.61	-2111.71	-537.463	-10254.1	-7662.65	-7888.61	-12088.9	-10234
7953.31	-3639.28	-3274.69	-4680.23	-10675.9	-11021.9	-2830.82	-13534.7	700.318

3011.06	505.455	7065.19	-7380.21	-3132.82	-17192.9	-94.2344	-996.656	-5810.16
-1853.94	-4323.06	-4522.58	-4931.8	-13554.9	-9708.66	-1571.81	-8967.14	-2961.79
2338.73	5767.43	-4558.4	-7011.32	-7423.76	-14835.5	2884.89	-13562.7	-8580.98
-1037.6	3575.29	-3933.14	-7042.83	-13009.9	-9410.74	-339.523	-10818	-3447.58
852.314	3478.08	-3562.34	-2629.5	-12061.7	-7464.72	-6064.32	-4744.62	-2823.89
-1713.26	2804.9	2800.16	-7817.43	-8932.21	-10168.5	-1992.48	434.877	-5806.87
1439.77	-2585.67	-522.303	-4522.7	-8012.49	-10909.4	-4178.96	-11148.2	-8941.87
-3201.5	14.8086	-6565.04	2222.25	-9126.92	-727.455	-1189.73	-6123.97	-9674.12
681.33	-4637.58	2295.07	1666.5	-9354.68	-2810.07	5739.74	-6496.65	-5372.76
-821.611	160.207	-2995.58	-2372.72	-17111.8	-4029.57	-3950.78	-5538.06	-350.836
3969.14	1680.7	-5958.64	-9929.38	-10968.5	-5826.7	1829.93	-3381.86	-4715.84
4436.11	-1407.96	-3428.18	1051.94	-12282.9	-10026.9	-4033.02	-6712.24	-2396.73
-3478.55	-419.932	-4862.2	-1217.86	-7275.94	-8923.58	-6563.77	-13435.1	-1487.35
-237.465	4684.17	1098.56	-4726.82	-10432.9	-1493.16	-599.271	-10224.6	-6590.15
9304.63	98.2051	2581.34	-353.533	-8296.59	-9451.11	-6849.25	-13185.3	-11838.8
-1444.83	6117.6	-2982	-3133.51	-14747.2	-6611.22	-2889.7	-9099.24	-6079.18
-7517.47	2291.96	-2544.21	-6267.95	-11526.8	-5061.81	-5886.1	-5341.79	254.83
4585.53	489.291	-4070.35	-6737.52	-7535.82	-8011.45	-449.842	-10801.4	-2415.34
-5618.76	4339.34	4342.79	3426.77	-3383.88	-12960.4	-571.221	-1790.64	-8621.48
5873.26	4962.63	4166.9	4261.07	-13846.1	-11792.6	-6457.46	-7319.61	-7424.19
4110.6	2125.42	-4318.28	-6904.75	-15448.1	-9231.92	-3039.07	-11462.2	-5325.97
-4777.46	-3181.44	-5865.22	1012.65	-5538.88	-8878.62	-3470.27	-5293.81	-269.313
5583.15	14.8652	6810.48	-3130.33	-11085	-9756.87	-3445	-6795.69	-2340.63
2872.84	6820.26	5723.43	-7745.25	-9951.28	-6012.3	-2413.03	-7208.13	-1510.35
-611.988	3151.77	983.334	-9133.92	-11498.7	-6258.56	-5785.79	-5847.56	-2000.84
-241.232	353.26	-3728.98	-6675.39	-8851.99	-4761.61	-5515.35	-10118.2	1925.38
-5642.94	3012.8	-5342.28	-9047.22	-12375.4	-9337.57	-2340.24	-6247.3	-2650.37
-3545.94	-2382.43	3263.48	-8225.15	-18365.8	-6046.07	-2431.84	-9169.54	-7673.64
3981.59	1757.8	-207.74	-4215.67	-2904.59	-4482.35	-4760.98	-8901.67	-8973.76
5013.3	-1888.07	1979.77	-8367	-11366.6	-7529.18	2716.9	-11358.6	-5134.59
-2003.62	7332.19	-6191.73	-476.424	-7758.62	-6917.36	-2676.2	-4619.41	-609.828
441.316	-101.896	2713.79	-3563.22	-15354.7	-9277.56	-755.559	-1804.32	-11088
-146.414	2444.57	-1482.99	-2903.76	-11194.1	-9301.84	-5749.62	-7427.03	-2357.48

-2218.49	1573.22	-838.975	-860.322	-9852.21	-7141.23	-5262.19	-14874.9	-1341.95
-94.9258	-3869	-4411.68	-2097.83	-10204.2	-789.977	-959.668	-12663	-3963.45
6911.25	-1578.17	-306.891	-7461.2	-8553.9	-5948.87	-826.828	-10254.5	-3059.74
2285.02	5448.66	4828.68	-2213.43	-7228.66	-5805.22	-3069.47	-9165.96	-7295.22
2451.22	3006.54	-5207.73	-566.574	-10732.4	-5575.88	-247.369	-5833.25	-1404.31
-5047.83	-2107.77	-602.049	-1900.44	-11792.3	-4908.86	-6020.61	-5407.13	1791.11
-1635.18	654.713	2529.66	-1437	-11467	-7712.09	-5558.88	-14628	-2010.8
4132.06	2990.2	-2138.12	-3861.27	-14698.4	-7024.35	-9514.94	-5453.05	-2989.19
2198.73	3384.53	3497.78	-8665.19	-9653.77	-3810.41	-5658.33	-7987.75	-3543.28
2569.98	2419.94	-3179.53	-6924.17	-9972.58	-11680.6	-3432.03	-11586.3	-1962.69
3554.1	2736.55	1758.03	-7802.41	-11221	-9704.56	-7988.89	-8241.58	-8978.22
3629.86	1832.25	3044.75	-11172	-15993.2	-10608.4	2460.36	-6439.14	-11791
1738	3706.48	-3944.43	-4578.98	-9496.53	-13905.4	-9453.74	-13444.9	1835.38
-718.143	554.312	-3848.95	-8402.07	-13670.9	-11122.7	-3634.69	-18387.3	-4650.33
5707.31	-1465.88	-2192.78	-5223.25	-11514.1	-6083.51	-3976.3	-13731.6	-1440.24
4248.87	-2053.46	-97.1152	-7811.58	-5978.11	-7132.95	-419.691	-7760.4	-5407.25
-1454.53	774.912	352.527	-8260.57	-14076.6	-7943.72	1099.41	-4546.11	-1888.22
1626.19	4273.56	-346.91	660.574	-8182.09	-1804.66	-713.359	-10150	-3053.04
-629.068	1259.46	240.959	-4966.97	-12164.8	-6804.08	-11122	-8211.79	-4550.54
-3409.11	2482.72	2164.37	-9784.71	-17434.1	-13261.1	-9605.48	-6114.22	-2595.89
10010.7	642.631	-1893.15	-11681.3	-5659.87	-6204.17	-8756.21	-5282.99	-7409.63
2724.65	-5433.08	450.307	-5866.75	-14323.2	-5532.79	-6756.91	-11311	-5700.55
3443.28	3100.64	4000.45	-1143.46	-11652	-10149.1	-6500.37	-8844.16	-5663.89
3713.5	2147.07	1758.59	2261.54	-18983.4	-9245.35	-4307.6	-5645.08	-6618.51
-4119.37	-2889.27	3283.67	-3331.43	-12606.4	-5129.45	-3697.99	-8971.12	-7979.64
-5627.99	-3032.74	4559.5	-3730.16	-8721.64	-5324.8	-5878.75	-11399.3	-14244.4
-269.74	3468.04	-1769.05	-273.637	-16895.7	-11442.9	-4984.8	-6546.83	-3596.82
2755.08	2540.86	6349.65	-6743.58	-6545.78	-3525.51	-1042.34	-3859.68	-2703.77
4452.88	-4638.08	6538.54	-5875.73	-9313.8	-9562.11	-6489.53	-4407.8	1828.4
-1430.96	-5009.08	2090.11	-919.459	-12611.2	-3822.06	-4268.98	-8572.09	-5756.51
1000.01	3719.52	-6131.83	-7057.07	-15812.3	-11284.2	-7481.5	-10652.5	-2493.75
24.9414	-1913.57	-2267.18	1926.07	-13069.1	-15647.8	-3013.77	-11850	-8365.4
900.961	4958.1	-4461.92	-6494.88	-18011.5	-2066.42	-146.443	-6481.22	-6694.88

8109	698.703	1279.1	-4145.32	-10977.9	-10258.8	1410.07	-6270.6	-4062.55
2784.94	4986.91	-112.678	-2746.58	-5646.21	-12268.3	1061.56	-4995.09	-869.236
-1421.76	4117.23	-237.406	788.398	-17491.1	-9867.08	-946.508	-3388.08	-6319.26
5251.43	3448.8	-2777.64	2351.34	-13183.5	-8506.47	1043.06	-6678.26	-5321.47
2313.81	197.99	-807.937	-8191.6	-6906.13	-10591.5	-4.27344	-5465.53	-1363.36
182.121	-1893.88	3720.65	-9262.2	-15443.5	-7857.82	-2058.96	-3347.12	-2934.4
917.084	10722.5	-561.064	-8168.9	-9474.04	-7685.32	-4887.89	-12041.6	-8144.66
2519.63	-303.676	1103.17	-4531.09	-9440	-2606.99	2678.99	386.045	-9647.87
1109.66	4563.38	6010.59	-3070.84	-12994.5	-6728.45	-3745.75	-3803.83	5072.17
8226.34	2835.21	4159.96	-3648.65	-19212.1	-8181.05	746.732	-1711.4	5047.96
6732.11	2297.92	3902.11	-3592.51	-12990.2	-13519.8	-4167.12	-6368.89	-3868.72
8297.96	216.195	-5149.97	-4810.42	-13331.3	-12822	-2035.51	-1989.63	-416.934
8171.02	409.445	-2730.18	-2944.46	-10688.5	-8909.69	-1411.23	-8819.92	-6464.49
3679.59	2492.71	-6229.21	-10451.9	-16092	-7914.81	-1139.62	-10067.3	-677.863
4915.33	6511.37	1093.58	-5188.5	-11210.7	-6445.54	-10487.7	-5866.66	-1894.07
8710.94	5488.32	5571.64	-4252.18	-5888.53	-9690.14	-8001.15	-4643.33	-3392.17
6052.59	5928.51	1897.31	-1221.87	-11866.5	-6994.8	-80.7637	-9243.54	1417.22
1256.89	3261.93	3472.7	-243.752	-12234.5	-4906.69	-992.662	-11513.6	-6688.24
-1341.02	3113.54	2255.91	-4435.51	-9180.68	-10263	-9542.37	-7055.86	509.049
3925.04	-898.748	5056.55	313.293	-6683.34	-5088.9	-3686.77	-4217.48	-3797.05
792.15	4258.24	1594.22	-1758.23	-10576	-11137.1	-3561.78	-9566.68	-5996
2320.82	-1237	1690.5	-154.307	-9624.88	-9452.13	638.639	-5656.21	-50.9961
8173.68	7790.28	-2721.45	-2718.42	-20038	-10613.6	-4809.39	-10366.6	-6697.16
-1944.25	1710.37	-1363.51	-1259.69	-9694.73	-5630.45	-5063.67	-11459.5	-2096.62
3346.23	2841.25	2070.14	-6226.22	-14214.1	-9969.95	1787.45	-10892.4	-4316.66
1740.87	-5716.5	-3764.1	-2329.38	-18572.2	-12380.2	-1387.35	-13478.4	-1897.88
1951.64	3710.99	-6789.52	-5388.47	-11220.3	-9278.03	-549.41	-10604.6	-4006.24
2425.4	972.916	4855.31	-845.801	-9114.08	-10713.1	810.527	-9755.89	-1988.15
5450	8244.68	-6382.26	1204.9	-14595.2	-8053.19	-1589.14	-8210.77	-4960.62
4961.26	-516.92	-1018.14	4233.37	-8348.16	-6435.44	-2926.56	-4572.81	-1138.26
-462.408	7708.56	355.109	-1141.24	154.025	-12059	-6377.36	-10386	-3665.38
-94.5859	7313.34	-2955.25	-5251.66	-10943	-4308.98	-358.152	-6741.04	-5742.51
-5597.82	2115.77	6603.74	-2266.33	-11156.3	-6617.65	3541.18	-6874.3	-11492.5

373.521	272.465	4348.29	1455.19	-6948.1	-7582.54	-3907.04	1768.34	-5884.42
1540.02	-1911.39	-3475.3	-5494.09	-9467.65	-8910.74	2661.17	-8348.23	-6620.76
-1258.46	3088.87	1925.27	-1198.09	-6051.78	-11191.2	-3290.26	-6719.13	-5170.25
9075.36	-1099.72	566.727	-7589.44	-14886.9	-12479.2	-7947.18	-7980.87	-3953.98
297.572	2338.83	-767.672	-8395.02	-13351.5	-5700.72	-11034.8	-10119.5	629.898
-450.98	-5876.38	1631.61	-5248.21	-11607.9	-5139.22	-3132.58	-11402.9	-7325.72
5099.01	2550.82	-1969.98	657.191	-12517	-10748.2	-4599.38	-6050.94	-2400.75
-1139.64	59.5547	4617.06	-4497.85	-5761.43	-11993.9	-4038.98	-5922.51	-2114.01
576.75	2511.7	-3712.92	-8186.35	-6003.75	-3782.06	-5114.43	-14927.2	1151.55
-1297.53	8310.49	-5931.78	-2790.77	-10020.8	-3973.37	321.275	-14164.5	-153.029
-8377.55	-477.264	-4058.68	-1062.83	-6789.82	-6271.05	-3116.03	-7097.49	-2197.82
-1387.07	-6898.48	-1952.3	-5650.82	-5339.67	-8301	-3686.4	-5285.45	-6152.54
-750.543	1786.1	1689.29	-5896.32	-13077.3	-1955.71	-5699.26	-5317.69	-3179.73
4808.96	3107.47	1615.8	-2281.66	-10907.7	-11386.4	62.7637	-5323.15	-1141.75
4040.86	-5544.22	-9041.15	-5802.22	-11478.9	-11486.8	-1486.61	-10971	1880.17
59.3184	3587.4	-5105.53	-1412.47	-11538.4	-7589.91	-4693.08	-9197.66	-8934.95
251.799	-3936.34	-5453.01	-4457.46	-12446	-14846	-1316.27	-10190.4	-7402.68
10185.8	-1286.28	-463.711	-1759.04	-12950.2	-13172.2	-3482.11	-10824.9	-4581.85
6643.34	1208.48	1967.82	-3454.64	-12371.5	-12737.5	-128.4	-11030.9	-6219.4
6209.27	4452.66	5758.84	-1153.29	-14884.3	-13692.5	1061.59	-5838.57	-4016.03
7993.15	-320.707	1637.95	-934.754	-14614.7	-13850.7	-2570.43	-9505.82	-6074.27
-3053.12	7997.12	-3400.56	-4686.22	-14994.1	-4206.9	-2121.98	-11396.7	-3243.92
-2249.93	2890.42	-1989.63	-4731.76	-8734.53	-3520.51	-11716.4	-6774.94	-10506.3
1715.89	8134.86	3787.84	-3358.74	-18553.2	-8150.42	-7148.69	-2108.59	3536.89
-2340.68	2630.44	2625.33	-5071.85	-10739.2	-1441.79	50.623	-10320.7	-1253.37
-4656.23	-4656.31	-2346.05	-4589.48	-7242.94	-6672.77	-1136.22	-7895.68	-7923.95
-2502.61	-4015.63	-3396.2	2160.78	-5817.48	-8715.6	-4355.91	-6402.92	-5871.21
-1549.9	1064.12	-2312.63	-4155.29	-8869.46	-15646	229.029	-5263.54	-4908.49
3086.64	7420.5	-889.773	-6649.35	-4956.07	-6599.02	-9055.84	-7658.89	-3549.02
972.338	-2273.62	-855.908	-1987.03	-6325.71	-12032.1	-6583.04	-12756.6	-8190.69
1092.42	9084.35	-552.17	62.6836	-10072.6	-11339.4	-5403.34	-8629.78	-4723.79
994.762	733.719	428.906	-7378.73	-7971.57	-5434.98	-4166.54	-11949.8	9.87891
997.85	4756.94	3384.82	-4024.09	-12578.3	-13024.1	-1847.5	-15055.9	587.943

1790.57	2864.35	3866.56	595.094	-14812.9	-8838.36	-9585.77	-12438.1	-2360.45
-3947.73	2719.54	-3844.25	-2668.9	-16143.3	-9484.26	-1770.82	-14418.4	-4240.02
-2217.63	-832.943	884.297	-3869.3	-10465.4	-13518.9	-2776.67	-6486.59	-3452.7
-3700.28	1200.2	3485.45	-1704.8	-13329.3	-10217.8	-1221.21	-8023.93	-7557.36
5230.43	1401.9	6126.38	-1658.07	-10302.3	-10304.1	-1025.78	-13718.5	-9418.31
-2516.73	1365.29	2573.7	-3508.8	-15655.2	-3237.56	-9970.51	-8487.8	-4407.52
6540.06	3191.59	1325.7	-3256.86	-6963.86	-7804.8	-5595.86	-5887.05	-3643.41
4503.69	-3059.48	1321.01	3074.8	-20047.3	-9901.02	-5466.82	-5422.69	-7028.17
625.088	3877.19	2591.54	-7078.74	-5783.92	-5795.1	-3593.91	-3079.29	-8751.77
1814.01	2033.1	-3659.72	-3471.66	-9786.36	-9263.9	6835.63	-366.883	-4771.18
-2054.21	3632.48	-4445.73	-2857.17	-16403.7	-5362.66	-5456.1	-9848.59	237.984
-5200.07	-2131.12	4748.1	5156.83	-13304	-15610.9	-5666.98	-7354.49	818.539
-359.279	436.74	-1924.41	-6769.63	-11009.4	-8084.94	-5519.18	-9085.91	-3230.05
-399.184	3091.69	1294.24	-1701.42	-13669.1	-12040.1	-435.816	-11171.6	-3702.63
-413.422	4641.18	-2400.73	-8254.79	-5581.42	-6739.13	-6263.89	-6396.8	-8284.77
-5131.56	5469.86	-2243.31	-8714.39	-5452.4	-6850.77	-5062.68	-8652.9	-1022.36
-3461.99	2012.74	-2116.74	-6406.49	-5648.6	-8032.83	-7601.28	-7948.67	-5641.51
-1241.26	4602.14	-2426.72	-7967.28	-12417.1	-7177.01	-824.141	-11366.2	-3137.57
1519.92	367.16	-646.672	-844.145	-10517.9	-3940.02	-3024.64	-10237.2	-8365.5
-2026.78	5915.51	2110.14	-809.924	-9783.45	-5414.38	-4559	-10209.4	-5196.61
3952.34	7282.77	-1917.72	-5936.14	-10490.8	-14139.5	-1546.76	-10197.6	1031.08
1673.85	6267.3	5000.75	-5054.72	-11877.7	-10533.7	-1276.04	-10352.3	-5502.19
-4268.73	4575.69	6924.34	344.588	-9230.72	-11110	-7765.47	-9186.44	-3140.02
-7131.06	4897.97	1310.99	2560.12	-13894.2	-2830.14	-4491.29	-7211.93	-6845.06
3066.65	3403.58	2442.33	101.592	-6253.42	-8757	-3490.57	-12265.5	-6114.6
2205.65	-261.67	5000.59	-783.422	-14708.9	-8539.88	-2573.36	-14809.3	-1394.07
-1517.1	3666.66	-3019.05	-6284.65	-15058.9	-5500.72	-3576.39	-2781.2	-5891.75
-766.727	3097.06	3432.59	-7627.47	-19533.2	-12276.1	-22.1602	-11223.3	-9270.56
4400.71	2774.13	2916.09	-4311	-10512.8	-13558.8	3104.67	-10873.9	-8001.71
4665.9	-3120.86	9552.58	-1484.19	-8340.75	-6140.95	-9374.5	-5801.01	-9998.42
1178.84	4076.31	466.006	296.061	-14848.6	-6435.79	-1846.45	-12394.5	-1736.25
3084.1	5391.25	853.418	-9976.21	-6750.76	-5592.97	-3506.94	-5923.09	-5818.01
-2094.24	7243.92	155.148	2626.16	-13869.1	-11094.2	-1734.89	-8697.75	-2019.14

2690.99	2557.95	2842.38	-1593.51	-10702.9	-8834.96	-4356.99	-13566.5	-4125.46
4279.33	6459.68	224.094	-10030.7	-11364.3	-5773.54	1134.9	-12033.1	1406.22
-249.664	8549.35	4474.95	1318.91	-14125.4	-4444.67	658.873	-4814.92	-6239.19
2249.38	-317.07	19542.9	4309.84	-11966.1	-5626.29	-903.969	-3722.71	-2366.61
-3438.05	2489.18	1276.4	-636.426	-8780.41	-8547.43	-5888.6	-2544.86	-6507.79
-365.178	-4908.99	3532.07	3983	-13668.2	-9494.32	-10467.1	-15569.2	817.744
-5150.65	5299.32	3229.17	-4730.98	-17645.5	-8047.61	-4555.2	-9182.95	-13914
-318.17	1426.03	2626.41	-5161.93	-11195.8	-15429.2	-3844.39	-3643.2	-1574.24
1678.02	-2171.56	3216.14	2618.12	-13319.4	-14094.1	-175.729	-5251.24	-5765.02
196.637	-401.25	4249.57	52.498	-17424.5	-5062.19	-9572.43	-1996.34	-5058.67
1889.96	914.635	-1719.78	-868.639	-12460.6	-9652.63	-2712.69	-105.279	-1497.38
3387.37	5592.19	1374.93	-7394.48	-5094.39	-9728.46	1251.68	-4492.14	-5811.27
142.896	-1439.29	-3963.57	-6149.82	-9729.92	-12531.6	-7844.53	-8171.59	-4129.96
1503.88	2138.54	-1743.34	-6859.46	-7275.89	-9144.47	-6183.78	-9209.92	1606.07
1677.19	8955.7	4407.2	-5452.61	-13822.9	-11845.3	-6504.48	-12859.9	-6631
-4165.39	4650.69	1269.91	2354.37	-14561	-11118.2	-3541.67	-7817.51	373.465
920.172	626.158	452.867	-5051.06	-14390.2	-10383.9	-3199.96	-4573.44	-4835.48
4299.65	-2185.13	-1458.19	-4618.88	-6840.19	-15554.8	-6723.45	-5380.34	-4069.05
-4339.03	2190.22	-1693.97	1704.39	-4292.81	-6681.93	139.996	-3781.78	-515.646
-2446.24	2611.76	-1856.07	-1076.04	-11397.2	-4826.79	-3.00781	-8862.99	-9530.93
10313	-5215.09	2423.24	-5460.79	-17883.5	-12081	-8431.55	-3350.92	-8121.86
1303.84	8375.82	6145.04	-6287.4	-17726.4	-7653.55	-95.4688	-8985.79	-4101.67
1910.59	732.627	-6110.7	-1383.32	-13343.5	-6520.32	-1525.01	-10274.4	-1410.05
3041.67	443.09	5020.11	1096.84	-14491.7	-2525.78	-564.732	-16854.9	-6943.84
-4848	-3140.36	2624.95	-1612.01	-9754.85	-4700.87	-1676.79	-2350.52	-2130.13
3585.35	2664.08	-2080.22	-5895.68	-10393.2	-5189.02	-4894.25	-1069.91	-6082.54
-6929.19	1289.47	-3030.21	-5671.04	-12399.3	-8679.23	-4756.35	-4805.75	-3399.95
-3474.9	-957.143	-538.584	-5728.98	-15152	-5295.89	-8296.43	-9034.44	-375.393
-1763.45	4186.59	-1411.5	-3108.27	-8055.75	-8067.15	-4166.63	-7237.33	-2698.72
3909.1	-1913.82	-6045.73	-7275.61	-14750.3	-7791.55	4052.06	-7520.22	-7412.3
1920.41	-3331.54	-2707.07	2208.71	-11346.6	-12073.5	-23.7051	-8477.06	-3051.06
1535.3	-576.518	-4855.17	-4085.62	-6770.48	-5434.63	-4720.94	-3398.45	-3649.12
4052.53	-2339.95	-1736.37	1140.93	-9800.21	-10198.9	2851.53	-4124.15	-1856.16

6136.44	-3519.75	-2287.32	-1284.43	-11373.4	-7736.46	-2726.28	-10474.7	1148.06
-2060.26	5652.68	-2114.29	-4724.96	-10887.2	-12928.9	-1974.01	-9750.47	-6235.66
-975.764	-190.354	2299.5	-7688.05	-12477.8	-10032.4	-5896.05	-6640.02	-2534.32
1629.87	8028.58	-1456.4	-508.74	-10236.1	-14996.6	-125.344	-5458.46	-4028.51
-2785.37	4609.72	-1834.92	-3461.65	-8267.65	-6477.08	-4490.11	-3539.41	-1226.67
-4359.32	5077.94	4678.57	-6445.43	-9470.61	-13295	-7682.65	-7202.13	-689.449
-2591.44	2231.19	-2812.38	-2465.8	-17679.1	-9055.03	-9228.07	-8290.19	-3493.34
-56.9668	-1682.26	-5880.67	-1341.14	-4063.04	-12908.4	-4435.37	-8978.63	-10599.9
1453.14	5535.86	-2854.26	-6132.09	-14207.1	-10587.4	456.988	-10952.3	-9044.42
-1988.98	2474.83	2149.19	-9064.13	-12489.7	-11376.7	-3533.36	-11400.8	-2850.97
5411.58	11610.6	11296.3	-2835.49	-13393.7	-10602.5	-4795.09	-8662.56	-2035.53
5030.54	1322.33	6697.32	-2226.08	-15435.9	-8650.15	-3960.02	-8443.84	-6315.8
690.072	6746.57	-41.3535	-7421.86	-10145.6	-13934.3	-2324.08	-14922.5	-6679.66
2419.13	6866.4	-3642.2	-12239.2	-6033.3	-8661.62	-4519.55	-5599.06	-3165.34
-378.459	1391.94	6985	-7019.11	-12694.9	-9789.44	-2972.56	-4318.27	-3910.33
-3629.83	-244.385	4011.9	-1341.24	-15106.6	-6427.6	730.576	-560.783	-1331.26
-4210.6	2146.34	7854.01	-4694.51	-11353	-5997.44	-5155.61	-6256.83	-3900.09
5448.24	-1698.34	1660	980.33	-11765.4	-2452.67	-2490.38	-9859.99	-2015.75
-2697.13	-2127.8	-453.537	-3631.13	-9641.69	-9236.89	-7406.53	-9961.77	-517.203
4657.78	2923.64	604.68	2287.06	-14066.7	-6250.57	-5243.74	-9329.76	-3149.22
-103.637	-192.676	26.1992	-2126.03	-9389.67	-16993.1	-4093.85	-7762.73	1928.01
2743.96	8271.86	-651.949	-5587.8	-12293.3	-7612.71	-8474.62	-8892.64	-5383.55
-262.699	3243.78	3789.86	-5019.48	-8190.49	-9865.01	2956.72	-12597.9	-4926.34
-3926.45	896.232	-1859.19	-4993.93	-10167	-8147.2	-4650.11	-6091.23	-5597.38
4684.46	3535.33	5427.3	-2780.9	-8266.84	-5608.44	-3543.46	-9161.62	-2906.83
3685.95	-520.725	3341.16	-5843.28	-7311.55	1311.21	-2134.4	-6288.53	-6595.58
-915.844	6369.94	4948.29	-246.57	-12047.3	-4363.74	-3133.66	-8701.69	-5856.09
-2894.96	6214.78	1481.08	-1214.58	-12504.3	-9992.42	-9341.07	2161.06	-2835.44
-3367.82	3071.33	3257.4	-2567.09	-9266.29	-6712.62	-1138.41	-11881.6	-4843.5
1335.74	-1447.03	-2786.42	2619.68	-9218.93	-15988.5	-878.018	-8355.34	-7514.81
3524.51	2363.72	-4173.05	-3478.43	-9087.77	-6442.2	1122.3	-6888.88	-800.58
35.041	4084.65	1092.01	-6040.07	-11790.2	-9428.67	1185.57	-7587.21	-1146.77
6496.97	1404.19	-3289.26	420.643	-7600.14	-11359.6	-2706.85	-7201.06	-3162.19

470.813	6528.37	2601.78	3025.99	-10812.5	-9090.08	989.057	-12471.1	-6657.18
955.66	-1647.86	1689.69	-2256.53	-7557.46	-12097.4	-8202.54	-9454.79	-4903.72
4767.14	7758.03	-479.768	-4436.11	-12653	-9392.63	-6885.55	-9473.04	-5385.91
-4099.6	2533.12	488.867	7127.38	-9335.43	-6962.53	-5776.49	-5960.74	-3254.3
500.246	-2458.47	2748.67	-4636.78	-11718.4	-9691.28	-9030.35	-9863.84	-7497.49
2124.91	8186.31	-5723.02	-6368.29	-11793.3	-10000.9	-3249.64	-10663.3	-2605.44
524.77	-2129.11	-2505.79	-6868.52	-18049.8	-11108.8	-7831.98	-8331.91	-4146.08
217.27	-2030.25	-5549.54	-3566.43	-11278.4	-12288.5	-1999.03	-9678.52	-6409.7
2308.56	1929.35	-1968.25	3889.96	-16071.6	-8432.16	-8457.84	-9330.79	-4639.09
8592.03	-548.635	-3225.91	320.236	-14949.4	-10200.8	-5639.51	842.443	-3764.26
-911.088	-3418.09	-1863.37	-300.457	-16891.8	-6584.8	-3291.93	-4674.28	1932.16
4826.66	4730.93	-5736.87	-6746.09	-16228.4	-13036.9	-4465.74	-8252.98	-10452.1
1156.05	944.699	-1434.07	-5807.65	-15738	-6909.72	-5790.58	-7638.63	-6139.88
2183.44	2383.61	1307.28	-2907.46	-7047.84	-7686.67	-1663.78	-9495.4	-6450.17
1792.76	3571.52	-708.744	-2762.51	-12334	-8383.62	-7245.98	-13978.5	-9050.47
-3232.2	7254.1	3660.33	-13449.1	-8505.44	-9021.53	-968.102	-7684.7	-3013.4
1624.96	7751.07	4263.02	-4846.65	-16820.4	-7868.61	-6324.53	-8920.81	1150.3
2747.72	-1394.79	-2088.44	-4411.09	-16293.5	-8827.71	-2856.15	-12221.5	-6085.82
2831.37	-3208.73	446.49	-1998.3	-12641.8	-5555.87	-6049.09	-7715.28	-14426.6
-555.838	5834.38	1761.42	255.143	-14440	-10573.6	-11223.4	-8825.11	-9688.72
-267.826	7551.11	2971.27	-6947.92	-14206.6	-8297.75	-9973.6	-10811.8	-3132
-585.086	5927.2	1178.83	4651.38	-18312.9	-5924.12	-2275.18	-9951.31	-8777.82
-134.801	-486.576	-475.77	-6699.64	-8890.04	-7635.26	-9997.52	-10981.2	-11096.8
-1766.88	6641.89	4784.32	-4447.81	-14085.9	-15297.7	-2852.48	-8948.36	-12222.9
-591.326	3843.97	-1605.22	3492.5	-7269.82	-9160.83	-5673.65	-10395.4	-11175.1
5115.94	2596.54	7644.19	-11084.2	-14471.8	-7304.81	-3651.92	-14108.3	-5377.05
2935.7	7350.53	-4754.12	-6800.96	-9680.89	-7521.57	-4080.19	-8349.3	-9257.13
2462.25	-741.771	-9535.44	-936.975	-5525.79	-12221.4	-3736.94	-2244.89	-7266.02
2265.81	-492.143	1743.07	2959.03	-11096.5	-6096.8	-416.973	-5649.73	-7371.38
204.545	-1731.76	3575.69	2093.64	-11310	-11582.7	-5978.04	-159.926	4953.74
3133.96	-2931.42	1878.04	-5609.72	-16416.5	-6049.84	-5596.2	-4476.26	-6473.27
2343.09	1664.22	-71.4473	-6508.69	-11931.7	-9192.28	-6801.36	-6528.67	-2478.98
-499.687	-4644.43	2230.33	-4526.4	-15865.1	-15728.6	2189.28	-13497.7	-4739.98

190.738	-3873.33	2881.64	-6026.71	-11927.5	-4922.84	-7571.8	400.4	-3699.37
3567.1	-4986.24	2458.47	-5377.93	-13513.4	-13941.2	-1836.07	-12929.7	-3286.36
2254.35	4009.67	1659.55	2986.41	-11781.2	-3324.31	-7267.26	-14183.7	-1681.56
1229.74	2508.51	-356.117	-5563.91	-14410	-11121.5	-1029.98	-7465.35	-8392.58
-2113.57	12181	-2859.7	-8083.37	-8449.67	-8939.59	-3926.19	-8653.66	-1793.49
622.369	6939.68	-392.902	-204.914	-13347.8	-6496.3	-6173.93	-10189.2	-5182.29
2453.81	2909.21	-3503.57	154.42	-14765.9	-10955.8	-9427.05	-2216.45	-7099.55
-5008.88	-298.418	-708.676	-2699.71	-11049.4	-17469	-5817.33	-9181.47	2417.91
-2884.05	6519.14	-4852.52	-1532.08	-10366.4	-9642.98	124.611	-2073.97	-8104.15
-3766.13	-1969.48	396.275	-6529.8	-10208.8	-8175.55	-2884.64	-3117.49	-6355.99
-7503.51	9540.43	-5908.9	-2801.77	-11659.6	-12487.2	3221.2	-5436.15	-4962.66
-1301.24	-1692.24	340.76	885.986	-8233.04	-11764.1	-4647.85	-3115.56	-7625.39
259.182	4193.38	2041.46	-2534.21	-13481.4	-6640.91	-2994.06	-8337.99	-5818.98
-3700.96	4211.64	-7334.72	-7928.05	-16736.7	-14830.6	-10218.1	2507.32	-389.264
-3150.13	-2897.52	2330.29	-8171.58	-6110.22	-8195.65	-2305.43	-11571.5	-5943.91
2301.05	4770.84	699.877	-5026	-11807.8	-4540.43	-5770.19	-7863.26	-2915
-2837.45	4322.87	2273	3636.6	-14468.1	-16778	-3469.07	-9502.11	-4072.02
146.818	7749.17	-4397.69	-738.84	-16214.3	-12633	-7180.08	-8048.75	-4010.11
6802.65	2851.52	-2278.99	-2512.39	-16517.1	-17915.4	2273.92	-15474.7	-4336.96
-359.465	2954.21	-1332.97	-10578	-12368.7	-10087.5	-4667.5	-3470.85	1673.79
-3321.74	2827.07	874.326	-6881.63	-16868.6	-14020.5	-2584.11	-6127.77	-5623
3709.48	-1971.38	-8830.05	-3759.94	-11409.1	-6757.59	-5902.66	-4188.68	-960.756
-432.078	3632.15	-5987.07	-3031.87	-9424.34	-11666.7	462.611	-8665.24	-1979.43
-1599.01	239.408	-4650.23	-576.195	-6632.77	-11295.7	53.4961	-4430.74	-5674.58
-835.201	6440.5	-2348.95	-3215.62	-8408.79	-9232.5	-1416.17	-9415.44	-4910.57
-73.584	307.311	-2520.07	-2399.54	-6464.8	-6710.87	-1578.98	-8989.86	-4687.89
-5887.81	5873.28	670.885	2200.54	-11140.8	-14520.4	-8619.38	-7548.88	1309.17
-5030.78	965.98	3493.36	6074.16	-16149.1	-4649.26	-7521.6	-9306.78	-11984.8
1653.22	-2652.84	-3359.44	-8441.04	-15627.1	-7383.77	-6007.73	-8342.78	-5266.48
-510.064	6484.8	-4588.94	5602.19	-9408.73	-11380.2	-1019.57	2695.81	-8386.8
-4463.87	2927.78	-6604.09	-2209.94	-13036	-8398.8	-9836.72	-1835.22	-7752.85
307.92	8232.63	-5476.5	-3337.46	-7839.21	-10580.5	1971.33	-6207.1	-8881.55
1929.06	679.24	4746.12	-3268.38	-5188.07	-11032.1	-3157.36	-4833.83	-4250.95

-3135.43	4797.91	139.227	-2758.03	-9485.07	-8876.54	-10674.7	-3831.82	-7227.84
-7191.32	6127.8	2250.23	-4640.2	-15034.3	-5869.82	-4669.2	-7748.06	-690.902
-1955.37	7552.98	-4019.71	-7737.99	-15000.1	-10269.8	-6627.07	-11652.7	-1527.89
-4315.31	5783.93	682	-4485.52	-12672.2	-10155.2	-2806.23	-6819.93	-9129.58
-3178.61	6998.35	-945.355	-7441.6	-14472.6	-12916.5	-4424.96	-4055.1	-9513.29
-2572.94	7989.6	457	-1241.37	-17018.6	-11450.6	-5273.77	-7362.77	-6080.6
-6149.41	3582.4	-594.637	-6275.81	-14249.1	-4060.64	-4984.4	-13022	-8655.45
4064.42	4286.87	2029.11	-4806.9	-14462	-8086.81	-7129.42	583.656	-6570.33
-526.836	-169.016	97.1641	-5493.88	-13783.1	-9847.66	-5217.21	-13252.9	-6363.49
787.609	-312.568	-244.908	-4803.58	-14816.7	-12116.4	-4160.45	-9923.09	-11156.5
-7803.29	2737.19	-4176.64	110.895	-17050.7	-14790.4	-3418.87	-7485.43	-9737.35
999.209	2260.36	-3693.76	-6299.17	-11110	-6367.25	-2864.96	-7626.43	-5081.26
-3239.43	-1581.53	-344.012	-6319.65	-15148.1	-3642.01	-6060.37	-7893.35	-2749.74
2310.69	-914.242	-3955.44	-4295	-11401	-13419.2	-5117.21	-4884.5	-2698.93
-3298.26	-1055.84	3324.86	-2606.88	-10571.5	-3206.29	-3406.79	-7905.87	-4332.57
-3991.62	1805.82	-7638.01	-3744.47	-12587.5	-8525.08	-8501.53	-8126.83	-3576.38
-177.393	1833.59	-3335.75	-8129.36	-3956.09	-9636.83	-444.684	-11592.5	-1049.44
1229.37	4803.45	-7937.03	-1909.63	-8528.79	-12002.4	4605.47	-3229.24	-4144.17
3462.03	1581.44	-3250.3	1719.72	-13714.3	-6410.49	1185.54	-10784.7	-5243.09
-6046.81	6518.13	638.32	-919	-9386.24	-8824.52	2241.02	-9799.26	-10172.5
-847.309	1859.79	-2672.2	-3608.22	-13320.9	-10488.1	-3568.88	-6554.12	-10855.5
1370.3	-1502.46	-7430.77	-6818.58	-14323.8	-8703.25	-6708.41	-4961.19	-11812
-2358.5	3929.6	3260.97	1501.29	-10772.9	-11226.9	747.877	-12031	-9652.51
-7086.64	-2185.52	2291.19	831.289	-8723.02	-10762.2	-10163	-7532.25	-3203.28
-1960.38	8521.63	2643.59	5056.35	-15447.3	-12809.7	2298.05	-10851.7	-3551.34
2939.5	3386.63	-91.3887	-1551.18	-4571.61	-10773.6	-4990.67	-6094.77	-1714.94
4820.95	8674.75	5676.04	137.539	-11333.3	-4051.72	-4442.38	-12456.1	-8887.91
-649.793	10018.7	1957.61	2108.31	-19557.2	-7294.28	-272.332	-5611.89	-10922.5
-4165.7	7674.25	-2036.81	-7827.75	-11976.7	-7288.95	-2860.51	708.914	-9637.18
-5452.56	1621.43	83.9375	-4275.61	-12505.9	-10497.2	-570.17	-10440.2	-5476.94
-3170.95	-199.555	-4891.64	-1055.74	-14104.5	-12568.6	728.506	-12519.9	-6987.59
6505.96	-1705.5	1187.41	-155.645	-13328.3	-9356.82	-3752.23	-4437.63	-2561.61
-4005.06	4800.48	1529.28	-5901.43	-9884.15	-7293.19	-8317.34	-5087.66	-4786.54

1619.34	3693.12	6275.48	-4712.97	-14316.3	-6945.83	-4575.37	-5511.41	-3461
-302.695	2653.04	-1933.55	-7380.17	-17087.6	-14104.6	-546.537	-4799.85	-7684.09
-8948.33	1770.86	3560.57	-5154.25	-9902.53	-7014.32	-5048.19	-6300.52	-7612.18
1221.31	-2480.87	-6513.09	1116.9	-17110.8	-16037.6	-8738.23	-2253.12	-8141.44
-4839.17	4900.77	-1157.35	-2772.76	-13468.1	-17696.5	-2784.88	-17455.6	-3259.38
4926.08	3523.67	1481.37	-476.564	-17075.4	-8995.44	-859.668	-5049.38	-5394.24
-4828.73	-908.258	-1268.25	-3110.67	-14153	-9369.63	-3276.14	-11028.7	-5928.97
-6917.48	2006.73	-1523.52	1906.29	-16279.5	-10593.3	-6960.53	-11647.8	-7020.69
-3039.84	4680.76	256.207	2559.02	-17778	-4657.69	-1941.26	-9787.73	-615.051
-1208.77	417.5	22.3574	-6652.87	-9038.44	-5963.88	-5327.46	-6050.27	-7790.72
1547.37	3851.66	4738.13	1164.12	-18693.3	-2936.84	176.82	-5052.82	-8299.51
-4970.03	6956.31	-1382.06	-904.867	-14266.4	-6446.56	1895.58	4.99609	13.6836
173.904	360.951	3050.07	-4789.31	-16349.9	-14110.2	-4157.55	-8927.43	-5397.77
-1469.38	-7309.39	6678.03	-11578.9	-9631.28	-9969.13	-6437.6	-8131.52	-7800.99
1496.63	5828.28	11176.7	-2971.25	-13263.9	-14018.3	-7690.56	-12510	-5479.12
-4181.6	1437.02	4841.7	-2113.9	-12718	-14506.1	-1556.58	-11572.6	-11926.5
-1710.51	6611.93	-4014.25	-1888.77	-8415.58	-12570.4	2183.93	-7071.29	-910.535
-59.5625	5460.97	-369.547	-9069	-11746	-10130.5	-1692.17	-12532.3	-6727.94
-5788.45	6821.85	-5770.45	3292.67	-13593.6	-14020.4	916.861	-6965.48	325.389
3533.53	3549.87	-776.453	-1993.09	-8864.12	-11112.1	-9285.34	-6032.34	-2702.77
3893.72	-796.559	-8109.57	-828.744	-6386.63	-18391.5	-5267.16	-14581.2	-3287.88
-3722.58	1942.38	-3344.75	-4354.52	-6846.4	-11099.8	-8995.5	-7733.62	-4823.34
-448.016	1276.41	1492.65	2251.32	-14953.7	-10741.6	-12472.1	-5995.98	-8356.02
2307.6	1097.59	1220.13	-1888.6	-14497.7	-5958.47	-8421.39	-7489.36	-6524.84
1006.38	837.727	1868.52	1693.32	-17934.5	-13351.3	-3398.85	-8868.31	1280.08
2649.35	-509.537	1552.38	-3674.69	-14343.1	-12654.9	-6323.27	-5391.65	-3595.61
2227.61	7960.79	-308.664	-3391.38	-11235.1	-12179	-859.729	-8735.9	-4282.42
1662.24	1406.15	-6647.05	-5574.3	-13400.8	-12652	1991.72	-7623.12	-5495.83
1273.48	-805.145	-4674.41	-6341.25	-11253	-4954.38	-7420	-4832.36	-2780.21
3559.96	5299.71	-4365.67	-1403.62	-14135.9	-12130.5	-3163.79	-11032.5	-5851.28
5618.88	-5108.28	-10672	-1495.39	-16401.5	-15510	-10682.8	-6767.43	-8765.37
3415.88	6763.9	-8267.2	-3180.66	-18444	-12466.3	-1693.38	-9293.99	-26.6465
1498.57	4753.25	253.918	-687.139	-17921.7	-15493.6	-5788.08	-3121.42	-31.4883

63.3633	3158.67	-938.82	-615.139	-6348.34	-7524.32	-7363.37	-7507.25	-9381.93
1094.46	2761.69	-3056	2451.96	-7285.58	-8288.74	264.217	-14201.1	-11125.8
2230.88	-931.049	-11495	-1582.3	-12308.9	-8839.64	799.961	-5351.01	-5359.07
-2761.37	381.562	-6271.88	1385.16	-12529.4	-10615.4	2240.1	-8023.89	-6041.27
1600.34	1637.12	-3911.83	-8610.67	-10584.1	-4994.46	-7297.78	-6949.88	-8292.66
6080.91	6142.82	-2481.58	-5636.51	-10011	-14846.3	-7153.67	-10807.5	-8507.69
6573.63	963.604	1454.81	-2169.66	-12781	-9632.06	-7596.42	-7526.97	-7150.74
3686.16	13905.7	-2101.53	-2309.46	-11580.3	-7337.72	-3079.17	-8417.99	-4703.04
-1436.15	3695.35	1555.51	-2148.65	-12088.4	-13572.4	-2072.21	-6117.3	-6594.23
-3289.46	6663.5	-720.668	-1827.24	-13598.3	-10207	-3138.95	-8206.34	-7841.74
-1182.36	-838.285	-1145.99	4472.73	-16200.2	-8881.83	-4418.69	1228.19	-4492.09
-142.43	6951.28	-1366.9	5.55469	-17726.8	-10097.6	938.795	-12376	-8729.36
-891.691	10841.9	-7803.99	-4214.15	-9329.66	-7973.44	-6558.64	-12384.6	1104.16
1456.38	4028.94	-374.195	-7265.91	-8309.62	-6071.05	-2916.58	-12270.5	-3030.07
7054.46	-2529.62	678.678	4293.03	-16564.2	-12135.7	1591.1	-5213.72	-6322.75
-1974.55	4010.2	-3143.4	-2102.73	-16010.6	-17236	-2636.32	-9832.76	-1089.53
-2827.35	-1164.29	-4737.34	9218.93	-6958.37	-13726.9	-710.332	-6768.1	-6805.21
-4397.9	-2136.33	-4140.96	-2983.44	-13959	-6434.39	-10063.4	-8972.46	-9382.76
4404.14	2092.62	-4840.44	-1254.93	-14180.7	-7232.98	-3057.58	-9569.23	-6326.66
5851.15	10591.9	4290.84	-5120.48	-12702.6	-13019.1	-4554.52	-5764.83	-10663.9
-2117.83	-1782.83	-1280.85	-3612.7	-16855.2	-10225.3	-450.213	-5988.96	-6263.26
3919.38	869.412	-3547.48	-4016.62	-12042	-8042.57	-189.662	-9746.58	1326.94
-2536.62	2277.7	712.678	-5167.96	-9251.06	-11198.4	-6139.42	-9242.29	-8829.25
-1239.81	1716.27	841.135	-2342.38	-12175.8	-11205.1	159.396	-9446.75	-6311.44
1044.77	2540.86	-794.148	-461.939	-12087.5	-12875.8	-7373.66	-14242.9	-1223.31
9211.66	-2140.91	-8612.24	-4419.12	-18485.3	-6567.07	-3511.95	-8996.65	-4475.38
2288.79	-3071.11	-3657.68	3735.74	-14036.5	-10948.8	-5092.49	-8957.03	-7728.64
1636.08	-670.652	1783.65	-4765.38	-9303.06	-11814.2	-1774.81	-10564.1	-10151.5
7888.01	381.088	1695.75	-1143.85	-11611.4	-12371.7	2454.49	-13132.1	-6820.6
-7814.19	-1190.66	-4661.07	1881.13	-8367.88	-17419.4	-6083.23	-6093.34	-8606.64
-7930.85	3696.19	2833.09	-3576.01	-13869.1	-11726.8	-7191.7	-15773.8	-11935.2
-3537.69	1419.01	-2618.66	-2621.39	-8705.12	-18618.4	98.5371	-5815.36	-3453.15
-7132.85	6462.98	-2457.19	-351.102	-13636.8	-12518	-2122.23	-9035.29	-4077.32

-4118.13	-3744.29	7067.82	1110.9	-7939.77	-6320.82	-1296.4	-6240.15	-8302.2
-1644.38	-3030.42	-5178.92	-3714.73	-13122.5	-3281.71	-5272.84	-9687.38	-5857.51
-1366.07	6825.79	-2283.19	-6714.19	-12275.4	-10628.3	-1500.85	-6274.36	-4188.07
1766.04	-6122.83	1444.12	628.045	-13464.4	-14063.7	1391.07	-4556.53	-8348.27
3998.85	-390.875	-4999.96	2624.68	-16385.3	-6710.57	-4945.01	-8905.47	-5166.45
-1697.69	5308.63	1650.48	-5340.4	-11597.9	-7868.61	953.592	-10127.3	-1236.07
2637	2051.98	-4703.59	1323.98	-21408.9	-10894.6	-5529.66	-6543.88	-6587.79
402.738	6010.23	2013.91	-8579.69	-13521.2	-13295.8	-3866.51	-12981.2	-5457.26
1338.77	8102.53	-1921.78	1276.72	-12034.6	-11140.6	-5988.48	-13535	-2199.48
-689.512	5053.24	-3363.6	844.646	-11494.6	-11889.7	-1635.76	-5320.96	-6459.89
4540.81	245.617	2305.7	270.615	-13163.7	-14711.5	-7121.42	-8264.92	-3728.33
-1871.61	8660.12	-916.295	-97.5645	-14199.3	-10403.8	-2628.6	-17849.6	-8774.6
-7063.29	9025.03	-2270.57	-4223.84	-13224	-9340.14	-9210.89	-11286.2	-16209.9
-5299.24	6534.13	2493.4	-4110.4	-6780.74	-10209.9	-5027.09	-9604.29	-4393.93
-1947.47	-3781.99	2505.01	-3295.59	-10373.9	-8764.72	-4976.61	-8980.33	-5455.84
-7265.75	2942.19	1316.45	-2942.1	-9938.49	-7340.53	-2712.07	-16721.1	-7175.2
-3102.17	-295.732	1669.34	6907.12	-9970.76	-13787.8	-4513.56	-7178.57	-9367.23
719.258	3223.42	5130.15	-3364.1	-6919.83	-10857.7	3168.43	-7584.03	-4669.49
2358.84	-3737.05	-3306.24	2087.02	-11680.6	-6555.95	-6024.78	-8419.02	-4193.18
-250.561	-490.799	426.031	-1576.95	-15608.1	-11898	-10202.9	-12762.6	972.281
-6000.85	3641.38	255.174	1576.32	-15036.3	-9466.76	-1429.61	-8855.83	-1919.15
-2871.91	-7340.41	-3885.6	-1265.61	-14576.5	-12640	-4577.94	-4820.87	-4598.69
-7880.51	26.0234	-5233.13	-2247.15	-6114.66	-6431.52	-5660.61	-7944.2	-2928.9
-6268.54	1602.7	-2366.37	-4149.34	-17570.8	-18172.4	-3643.34	-8690.46	-5634.7
2187.3	9385.52	-4462.08	-2032.98	-12524.1	-11819.1	-5868.77	-11005.1	216.334
-6441.93	2070.24	3557.9	-2840.5	-16157.5	-7258.35	-1054.45	-12089.3	-249.805
2006.96	-1932.17	-104.57	-2256.11	-17397.3	-13490.5	-11438.4	-10867.5	-8142.68
511.797	-258.564	510.65	-2172.12	-7890.35	-16723.4	-1413.86	-9130.05	-14395.5
-3782.15	-542.453	-862.488	4450.36	-11654.6	-6036.18	-5121.47	-15499	-2489.22
1881.65	-2870.06	4645.67	131.582	-12422.7	-14808	-721.422	-7989.32	-8194.32
1418.08	-549.92	-7182.03	-8166.75	-12942.7	-16173.9	-5509.7	-9995.09	-7402.72
-2227.05	2337.02	954.365	-6165.15	-14297.6	-13131.3	-4903.61	-3769.71	-5510.42
-11447.8	9819.48	-463.598	-398.256	-17833.9	-15675.5	172.742	-6601.79	-1530.9

-9453.25	5579.88	-1324.68	-2211.17	-10819.1	-11858.1	-2558.01	-9234.96	-11358.4
-2987.55	-4372.9	702.168	2710.99	-8069.74	-12059.3	-4524.83	-14640.6	-7004.2
-7154.22	-887.365	-2179.55	-1299.66	-13894.6	-18399.7	-692.166	-4861.74	-12396.6
-1279.62	-469.125	-1201.71	-260.387	-10581.4	-9611.44	-5322.24	-6178.97	-15483.2
-4301.66	5001.03	5566.79	-5674.57	-13640.5	-16215.9	-1139.68	-9068.52	-7421.36
2947.59	7681.29	652.537	-4793.57	-15125.3	-21233.7	-1003.75	-14364.8	-1284.05
-1147.33	8074.76	-2165	450.281	-13354.3	-6042.03	-7173.44	-3963.96	-10291.5
-4012.93	5472.44	-4296.95	-6694.6	-11386.2	-13808.6	-5372.29	-6877.48	-5772.01
-494.838	3063.17	-6329.17	-335.629	-10774.8	-9389.2	-172.764	-7720.11	-8609.13
-11895.5	213.316	1303.46	-5973.96	-17359.1	-10937.3	-5585.23	-12033.1	-5574.41
-6524.43	6037.42	-3594.17	-2600.14	-13885.6	-13911.7	513.492	1026.73	-8250.84
-623.973	-681.895	1871.82	-5424.45	-6974.98	-13982.8	-9571.56	-12059.7	-6094.37
-1350.63	-1091.97	4935.24	2518.28	-9978.26	-11641	-3100.39	-8488.83	-2380.23
797.152	1625.27	231.168	-608.654	-21094.3	-14560.2	-411.258	-6241.89	-7261.53
-5968.36	4493.69	49.7559	-2120.32	-11810.2	-8887.19	-823.646	84.418	-11731.8
-4691.41	5346.89	3611.53	3704.09	-12948.1	-9455.91	-6684.87	-12943.3	-6610.52
-545.027	4597.81	741.199	6898.41	-11505.1	-5652.09	1459.34	-10561	-5765.78
-2450.17	2109.81	4861.76	-5566.13	-11637.2	-14095.1	-1612.1	-10256.9	-1351.25
2845.94	1774.97	1976.18	-8795.2	-14087.8	-20159.1	-2605.12	-11892.6	-6063.17
-252.877	7008.33	-1962.86	-7174.14	-13043.8	-11589.4	-10292.4	-8086.22	-3808.91
-582.338	5021.98	-8231.47	-4272.2	-11530.9	-15245.5	-10523.9	-13157	-1811.82
800.211	4713.56	-1345.66	1034.53	-12731.7	-12439.3	-4079.82	-7197.26	-9441.8
-1085.93	251.854	2720.69	-2496.04	-5292.55	-12706.2	41.0781	-620.707	-5014.5
-6968.6	2950.63	-1215.7	-2565.99	-13036.7	-10689.2	-2965.61	-2114.31	-8208.4
722.873	53.9902	-5649.18	-3654.39	-11247.3	-15958.6	-5343.71	-15627.9	-5783.01
5774.73	4628.54	-7516.99	-2861.22	-17683.7	-5129.01	-4422.76	-4688.86	-11487.5
3756.86	2951.08	-1638.39	-6103.19	-6601.98	-9948.59	-2613.71	-11801.9	-1019.42
-374.197	923.164	248.303	-4501.63	-11576.7	-21208.5	-12091.5	-13977.8	-8281.07
-852.744	-502.824	-4741.65	-1221.84	-17034.7	-9219.08	-6459.26	-8083.68	-12614.3
2661.71	821.701	-1177.5	111.916	-7606.3	-16632.3	-2703.94	-6404.44	-11089.8
2376.88	4075.92	-119.168	-1096.38	-10504.1	-11735.1	-6112.61	-11336.2	-6182.95
448.357	3851.74	-1407.01	-319.875	-8693.9	-10247.5	-2510.37	-8420.68	-5253.23
1173.65	-1791.83	5410.21	6773.09	-7730.02	-8652.2	2159.44	-4737.96	-7827.25

496.262	4017.58	-5894.03	-1435.73	-11360.5	-7640.24	-6744.89	-10659	-8910.15
-2769.09	-1678.4	-5796.88	-1503.57	-15650.5	-14678.9	-4818.13	-10228.4	-5819.26
1849.13	-0.349609	-3862.96	-2930.47	-11461.6	-11528.8	-1728.38	-11424.1	-10581.3
-653.469	3562.96	-4825.75	-3390.67	-11135.8	-10944.8	-8389.01	-8241.53	-6662.54
-6348.5	-2659.08	-3884.65	-2331.31	-12634	-10052.7	-1116.85	-6420.27	-37.4375
-1403.56	-3842.57	-3837.33	-3493.02	-10705.8	-8185.11	-5720.17	-7740.1	-2833.58
-2947.09	1128.78	-1132.36	-7843.68	-16866.7	-17294.2	-5539.36	-505.377	-4059.57
6344.61	1938.18	713.463	-3162.16	-17895.8	-8974.61	-2125.78	-8173.56	-3705.64
-4557.3	3853.53	-4341.75	-3649.35	-17852.8	-12815.5	-6655.24	-10606.8	-11780.4
877.363	3670.55	-3068.71	-4804.75	-15977.5	-9222.03	-3444.2	-3920.95	-10442.7
681.33	-375.186	2345.54	-4191.28	-12306.5	-11720.9	-4872.32	425.801	-9951.14
4672.54	-2397.34	2636.67	3106.69	-14393.1	-7647.35	-10810.4	-12500.1	-7001.5
-2271.61	4326	-1976.33	-1952.14	-14216.3	-10594.6	-8126.39	-7452.43	-3952.97
2358.47	10241.3	-866.35	5297.45	-18904.4	-12476	-6404.3	-7850.24	-5414.23
-1609.08	3536.25	1463.05	-995.055	-8967.25	-12430.9	-2044.29	-4460.57	-6056.46
-9465.45	-2541.7	-4807.16	-1193.75	-12105.2	-10681.8	-8537.7	-9390.94	-9172.81
-1346.95	1074.99	2564.25	-1080.38	-16194.4	-10251.3	-7391.91	-11243.5	1194.34
-4675.47	7366.72	-4457.45	-3509.65	-24392.7	-11411.8	-8139.71	-8269.08	-5515.58
-5082.73	1738.39	2980.23	-5202.92	-8351.02	-15965.2	-4101.17	-6017.09	-8834.65
-2246.5	-3949.93	-747.686	-2750.03	-4321.17	-6987.61	-5721.87	-9432.52	-6060.13
-4154.45	5360.4	1281.03	-10769.2	-14512.2	-14341.9	-3090.45	-6379.94	-9057.7
74.9766	-2909.91	-4329.49	-2817.77	-13598	-6484.16	-2301.5	-8483.01	-4869.79
-3159.46	-1161.02	3457.47	-4934.4	-18517.3	-17323.2	-6873.72	-9085.82	1598.63
-6587.87	1257.73	-2155.38	168.023	-9906.64	-10618.5	-6559.38	-6143.15	-9054.21
-1289.19	258.285	1731.91	392.342	-12753.3	-14022.4	-4560.73	-2763.45	-8424.92
1854.81	3931	-4556.53	389.91	-13070.1	-7863.73	-3761.1	-3693.78	-7453.56
273.234	-1080	316.035	-469.621	-10338.3	-14653.2	-5604.67	-4240.73	-3403.72
8.91211	329.518	-4136.62	-738.594	-7809.23	-8877.33	-3725.63	-8020.62	-5923.92
-5378.22	2579.68	4639.47	-415.947	-13984.6	-10469.3	-8205.26	-2344.12	608.178
2756.78	1569.84	2556.07	-5124.79	-16477.2	-8304.63	-2785.96	-4482.75	-7529.13
-1595	-2016.15	-5269.51	807.148	-11021.9	-12355.5	-8808.63	1637.68	-646.043
-3090.4	-3464.94	-4438.43	-2416.18	-11460.4	-14084.2	-7003.78	-5967.59	-11775.2
350.141	-98.5117	-5540.33	39.459	-16136.7	-14581.7	-4288.57	-8866.21	-5568.74

455.369	3011.26	786.271	2722.13	-12068.9	-13466.3	-3446.42	-6672.08	-8071.5
-3552.7	8176.67	-2267.16	2336.85	-15805.3	-10235.3	-3603.58	-6874.17	-7704.87
-5747.49	6688.45	4660.2	-6712.36	-9267.78	-10113.7	-3693.01	-10944	-5998.9
-18.4844	5642.56	973.477	-1055.07	-13207.6	-7853.52	-3360.33	-15864.2	-6083.78
-5024.42	4076.04	-95.543	-6886.04	-12491	-6471.66	-6314.15	-2722.53	-4255.06
-1890.51	8722.6	-5491.98	-1776.63	-13486.5	-8401.26	-8661.37	-10827.3	-2934.85
656.127	-7068.85	-2595.21	-5320.49	-14797.7	-11302	-2037.06	-13373.4	-9427.41
1184.62	2799.78	-5680.87	-2296.14	-11293.5	-7417.27	-4770.19	-7365.94	-6858.93
2507.49	-277.051	-3725.52	1381.18	-13122.8	-10848.4	-3437.9	-10290.1	-11133.4
682.164	3318.92	4472.5	50.4883	-13699.9	-5874.15	-5474.51	-8266.98	-8682.88
310.482	3120.16	595.607	-3317.23	-7398.17	-7364.17	-5050.29	-7364.06	-8649.39
-4567.86	-1690.29	-1590.67	-1952.81	-17256.1	-10179.3	-10459.9	-3112.97	-3529.41
-5057.19	1902.11	-878.77	-9967.79	-9995.74	-17399.3	-10646.9	-11334.1	-6779.89
2637.4	-586.67	-7472.77	1166.38	-16394.9	-9647.98	-5421.25	-8157.19	-3870.79
4886.52	-4950.33	252.412	-1604.22	-13780.1	-12383.6	-2237.49	-12818.7	-2651.16
-2919.17	-134.588	-12888.1	7221.13	-11123.9	-10883.6	-605.203	-4443.62	-2542.24
3215.37	717.021	-2787.79	563.023	-13817.3	-14159.7	-1468.78	-6122.36	-7475.28
-4780.14	-887.98	1728.16	1775.62	-14726.2	-13617.3	-6302.32	-10628.1	-5334.51
-2201.72	8385.02	-2511.33	-2338.68	-15536	-16344.7	-8506.31	-12321.1	-395.697
-207.691	409.166	1019.96	-2092.97	-12154.5	-10055.3	-3894.68	-6015.12	-6947.75
407.527	-857.219	-2539.47	4799.36	-13611.9	-10473.4	-8852.13	-6383.25	-9433.91
-2364.71	4589.78	968.648	-2290.4	-17036.1	-8104.51	-7117.71	-6289.79	-2830.67
-6597.69	1746.56	-7438.95	1212.62	-10820.9	-9156.5	-8018.39	-8193.68	-1254.47
2590.92	-1084.59	-6796.67	-7367.06	-15397.5	-2418.29	-1348.86	-9484.62	-10479.2
-2487.35	4052.63	1723.13	-4983.07	-12643.6	-11250.5	-7690.74	-9546.29	-8612.46
-156.977	4218.19	-5595.12	-2440.78	-9859.18	-16133.9	-4532.46	-8186.44	-2822.85
-6466.33	1843.15	1041.34	-319.242	-14495	-12791.2	-5063.11	-9665.73	-1220.54
-6214.55	4215.67	-421.625	-511.242	-11332.8	-15538.8	-4398.48	-8575.22	-7034.08
-6636.79	6799.25	2223.57	-5716.9	-11359.1	-13961.9	-7621.97	-12852.1	-6929.73
-8352.17	2766.53	7052.28	-3127.62	-16441.8	-9692.92	-10088.2	-8803.11	-6869.51
-6189.62	755.168	2763.49	1465.13	-15413.2	-15471.3	-2165.51	-2934.5	-4370.65
-5940.74	-3133.03	-2208.68	-2603.35	-14396.2	-13693.6	-3541.61	-7472.33	-6932.56
-8049.79	1756.93	-4980.51	-5577.3	-13531.5	-11358	-8435.01	-7872.47	-9637.18

-3274.27	836.748	-9213.69	-5319.08	-9300.67	-14589.8	-9461.21	-6742.43	-8987.52
-171.586	-2629.41	-3170.33	-2192.67	-11113	-14227.4	-10193.6	-11178.7	-7439.55
-3189.08	-401.027	-4884.68	-10151.1	-17048.5	-7981.81	-7614.96	-10731.9	-1408.77
2939.25	-4140.33	-6197.37	-6.07617	-10383.3	-13108.4	-4349.92	-728.523	-12499.2
-3091.17	1759.64	-118.383	1747.35	-10969.8	-11402.4	-2870.3	-5808.3	-6612.25
-2358.37	2509.12	3974.67	735.781	-13510.4	-3131.07	-4460.67	-4988.61	-3136.67
-8919.57	37.6582	-3300.15	-4650.95	-11183.4	-6581.61	-3483.77	-11542.7	-5690.87
-2140.5	2965.2	-1521.19	3128.23	-11919.8	-12623.7	-12248.1	-9022.14	-12739.8
-3964.29	-536.412	-4408.83	-1412.19	-8620.22	-7549.25	-6430.25	-3752.8	-1251.12
-2072.12	2713.22	7456.31	-7352.08	-12679.5	-14259	-6260.06	-11396.9	-5816.11
-5401.26	-1578.95	-5616.41	-1387.55	-11193.4	-13629.1	-9567.86	-11820.1	-9634.83
1658.22	-2222.38	-6476.56	1037.32	-13468.4	-14778.8	-1463.69	-9894.33	-2215.8
2991.17	-898.076	-1750.1	-3413.54	-13234	-10307.1	-1314.91	-10276.7	-8556.15
2930.79	7157.34	-4647.51	-6734.24	-10955.8	-7637.28	-793.094	-7522.23	-5807.6
1591.63	-5988.81	-7244.17	-2005.42	-18902.9	-7792.84	-1997.24	-12868.9	-5953.42
-3886.67	-163.311	643.24	2759.45	-11581.4	-8571.27	-4389.28	-9140.6	-11156.8
-4722.42	-1011.29	-4039.03	1566.63	-12684.3	-15927.9	-9984.11	-6833.61	-9427
-590.707	9539.03	-8709.01	-5381.07	-17918.1	-7631.78	-6797.01	-9374.79	-5010.04
1158.55	2428.02	-3673.31	5448.14	-16259.6	-10476.5	-6984.9	-7741.26	-6001.91
-2324.46	-1175	-2887.16	4350.09	-16026.1	-2107.61	-11403.5	-2550.1	-2795.88
-3423.07	4139.16	-10488.9	2967.06	-10516.5	-8012.67	-8907.86	-9136.22	-3417.97
-3515.83	-2096.5	-3093.63	-1838.69	-15244.6	-15112.6	-6468.49	-15266.1	-8635.97
-1303.65	-1488.93	-63.4512	-2361.62	-5595.55	-6638.72	-5500.09	-10606.6	-10824.1
-1607.97	-560.102	-4768.26	1505.45	-9244.57	-12089.9	-10068.3	-8036.09	-8894.21
230.057	-814.736	-3503.46	1770.51	-14670.5	-21556.1	-3304.87	-582.07	-9423.95
-1017.15	3770.3	-6181.17	2668.7	-13417.5	-15581.9	-3308.27	-4230.98	-5920.84
-2083.58	-4452.21	-3883.8	-5745.16	-12434.8	-10044.9	-13074.5	-10770.3	-9933.22
-3418.53	-5383.25	-2683.88	-6007.61	-14863.6	-11448.6	-792.197	-7023.66	-9878.88
-2750.13	653.287	-3381.31	-6889.31	-17989.4	-16145.3	-11700.1	-18327.7	-3745.38
-1681.88	9372.83	708.299	-6622.88	-7482.25	-15698	-3935.15	-9840.67	-2693.15
-7138.62	-2634.16	-4222.41	-6902.32	-14828.4	-8523.47	-9792.14	-6431.5	-3835.3
-8054.86	565.387	-1789.33	-3652.88	-13628.7	-4817.14	-10139.1	-8383.74	-2840.7
-5086.9	6472.72	1170.94	-7903.24	-12216.9	-6181.5	-8033.41	-13163.8	-439.9

246.58	3250.68	-8870.54	-1894.55	-10791.1	-8043.54	-5392.59	-7793.32	-7378.54
7553.71	-3934.36	-5663.62	-5910.77	-11802.9	-7749.07	-4025.14	-11134.9	-10172.7
2701.27	-4672.51	-6383.02	-1846.9	-9558.65	-6743.37	-8103.66	-7158.8	-8788.96
-1317.21	2451.57	2018.04	-2156.76	-9327.75	-11305.2	-3875.22	-12877.9	-10466.8
-622.365	-3243.78	-3379.97	2145.17	-15142.5	-4936.91	-3509.72	-4729.86	-9616.57
771.795	1190.27	1137.33	-3802.59	-15333.3	-16834	-5833.24	-10519.5	-8389.33
-6363.85	5562.29	-10196	-2049.62	-13684.8	-9228.41	-3973.49	-12370.8	-10626.2
-1166.76	-2619.31	-5499.95	-4466.31	-15642.7	-9253.25	-8168.87	-14368.7	-5322.89
-3174.75	554.004	-6180.05	-98.1992	-18790.5	-14828.1	-3716.55	-8342.06	-13208.4
-2747.07	5741.78	-4632.73	-6178.64	-12868.7	-12138	-4218.56	-13726.4	-9680.18
-4529.97	3976.45	-4395.99	-349.867	-16488.8	-8587.57	-6170.32	-11480.6	-2519.17
-7582.8	2927.87	-625.943	3371.22	-10879.1	-11422.9	-3216.06	-3488.61	-6946.47
-13418.4	5453.7	-6970.82	1967.83	-16778.9	-6187.96	-1378.02	-14323	-7880.34
-2998.11	4944.06	-5777.28	-2287.86	-12254.7	-13363.9	-5255.58	-10612.2	-14916.7
2438.22	132.072	-938.326	-2271.01	-17718.3	-9737.3	-3317.11	-8068.69	-6134.59
-664.896	2958.63	-2047.52	-6528.15	-12246.9	-15595.5	-3701.54	-10518.7	-9836.93
724.879	-2976.92	470.406	-10805.6	-15610.4	-14411.9	-10667.9	-9680	-6957.5
-431.553	3995.44	-6845.8	833.229	-14957.2	-16049.2	-8588.64	-7573.79	-4808.22
-6631.04	-6799.17	-3434.09	-4281.75	-13855.6	-8454.95	-449.688	-9269.57	-11015.4
-1724.66	2385.04	404.023	-4660.15	-12578.1	-9317.85	-1368.87	-15034.3	-6847.06
-3692.4	434.057	-2086.04	45.9082	-8214.46	-11206.7	-4092.86	-9522.28	-9933.71
473.932	-6731.97	-882.99	-1579.13	-23225.1	-15028.1	-2773.39	-7327.04	-10213.6
-2193.78	2195.11	-1319.43	345.398	-19573.5	-13062.1	-163.496	-5823.86	-11966.5
-5692.82	3890.17	501.195	-136.861	-13820.9	-13444.8	1710.44	-8265.32	-4590.11
224.496	-2605.91	2472.82	3484.71	-18845.4	-14415.3	-4273.77	-9762.86	-3088.52
-10.9785	-905.209	-2377.2	8.375	-15422.4	-16508.8	-6193.45	-9292.06	-7947.71
-3787.53	408.607	-2635.44	-2167.19	-15159.7	-18889.9	-7416.13	-5663.14	-4443.08
797.246	-1263.43	-224.404	2810.16	-13580.1	-15392.1	151.643	-13685.9	-3447.58
-7487.3	1952.56	-385.109	1320.63	-15314.2	-10509.4	-10142	-5757.54	-6101.53
39.8594	3324.85	-11088.3	-5805.25	-10848.8	-8406.76	-336.279	-9619.85	-10906.1
3358.99	3343.11	-2394.85	-8941.77	-9088.87	-15350.4	-4714.02	-4986.01	-9236.66
-2928.03	1338.16	-8191.23	-4367.56	-11819.1	-10429.9	-11675.1	-9524.24	-3797.54
-2417.92	2139.63	-1743.94	3529.36	-12431.3	-4781.36	-3824.09	-4477.52	-11886.4

-3454.73	6983.75	-6427.06	-2896.68	-13587.6	-14825.5	-1354.51	-10400.3	-7595.89
-4539.32	-487.219	-4368.7	-6231.19	-13010.7	-11490.9	-7352.22	-12725.9	-6466.77
-456.293	4318.2	-1774.42	-361.92	-9501.02	-10345.1	-3346.15	-11906.8	-9124.11
1404.71	2738.59	3116.19	-3217.7	-16633.7	-7522.71	-2990.79	-10126.2	-5764.61
1732.44	-3391.27	377.051	-3109.79	-13128.4	-9687.36	-10465.7	-12638.9	-5653.1
2812.25	2165.41	-9144.7	-4405.13	-18502.6	-18842.4	-1771.13	-3638.14	-12199.2
-3951.5	8177.48	-5073.75	1691.56	-13830.4	-16704.9	-2673.21	-5810.4	-7458.44
-7725.86	2043.42	-3994.56	2016.64	-13762.3	-10061.3	-7697.91	-7901.13	-6282.63
-134.398	-1078.69	-423.602	720.17	-18629.1	-10877.6	-9580.49	-10538.6	-8802.1
603.498	1870.42	1642.84	-4662.54	-11115.4	-12836.5	-4439.45	-8254.63	-8636.11
805.369	1692.33	-2341.89	1438.87	-13964.1	-12324.8	-10597.6	-6285.49	-6285.67
1558.8	678.4	2636	1986.15	-11664.1	-16945.7	-13298.6	-10132.1	-9813.96
3756.34	3700.3	203.389	-1112.31	-12191.3	-12210.4	2708.4	-10098.2	-6862.01
-1307.45	4319.01	-4381.32	-2159.12	-15886.3	-10463.3	-6254.56	-10291.4	-11968.5
-5295.01	4543.58	-6574.96	-3571.57	-15977.9	-18715.2	-8273.97	-5693.82	-9387.98
-5474	-3407.21	1531.84	-1901.56	-15086.8	-13458.8	-737.178	-9944.02	-5679.69
-2794.42	-3284.47	-1617.82	-311.17	-15474.4	-13290.2	-7905.01	-10572.5	-7364.91
2361.31	-3351.53	-2522	-554.203	-9567.63	-17113.9	-7650.33	-10132.3	314.492
-3376.22	584.74	-3762.19	-10606.4	-19166.6	-17521.7	-3274.07	-11142.4	-7036.85
-1524.76	1297.92	2034.1	-10608.8	-16811.8	-10789.6	-8284.41	-10445.5	-8020.97
2238.17	-2343.5	-2879.41	1204.69	-17589.9	-13913.2	-5644.36	-6309.91	-7453.97
-1364.56	-2604.63	1718.43	-485.975	-6511.3	-11879.3	-4089.58	-11829.1	-5857.51
-2612.13	1897.27	2212.23	-6811.6	-10386.5	-7232.92	3452.74	-11349.9	-4560.68
-40.5664	4089.93	-203.787	-1733.81	-22709.1	-8537.6	1761.6	-7377.21	-8054.73
3076.35	1508.51	-53.4121	-7663.52	-12809.2	-15389.4	-4566.32	-4369.21	-9838.21
-1669.55	4240.51	-3546.08	-7684.28	-15400.7	-14070.9	-6367.72	-10907.2	-6162.3
1397.67	6969.37	-1224.21	3065.67	-9529.38	-11256.6	-3968.95	-5302.26	-5714.42
-4116.34	5050.62	-2736.47	4434.6	-12508	-16326.2	-6775.82	-7713.05	-7411.4
866.955	-4790.05	-2351.48	-5848.88	-12503.3	-8226.92	1494.31	-13569.1	-2172.36
691.584	454.389	-5031.71	-7347.5	-12560.4	-18141.9	-1202.83	-9532.74	-7420.18
-6577.58	-521.367	-999.883	-6722.79	-13776	-15172.7	-7992.1	-5761.43	-6462.24
-4860.11	-2722.79	-7472.09	-8009.18	-11136	-16197.7	309.783	-7385.08	-6515.82
-1512.16	3200.65	463.85	-295.699	-18198	-9331.66	-10404	-8366.25	-7824.69

-1442.85	8473.2	757.771	879.008	-5774.18	-14850.9	-5477.88	-10762	-5980.85
-1218.62	-2310.56	574.924	-1481.01	-8222.05	-13552	-8715.12	-5628.35	-5346.2
-2011	-3310.28	3736.62	-2594.44	-10169.6	-13611.7	-2397.02	-11093.8	-6241.92
-2304.75	1182.41	-2579.36	1827.04	-18912.2	-11307.2	-4675.93	-12982.5	-9543.69
-4213.14	2608.01	5678.51	-1558.05	-16096.7	-14085.1	-9658.19	-10185.6	-3310.92
3583.07	1717.28	2827.59	-2688.99	-10020	-8422.77	-3922.21	-4357.98	-4390.89
-1415.27	3202.47	4936.18	-3648.54	-12238.3	-16882.8	-10103	-14267.6	-5225.04
-6249.17	2176.26	-4303.08	-2523.28	-17115.7	-12466.2	-5564.17	-7963.79	804.947
3569.57	1331.87	1312.79	-702.434	-11720.8	-10353.8	-5420.45	-6457.53	-8229.64
2421.38	1199.36	-1779.22	-2415.54	-11309	-15042.1	-2685.5	-11832.7	-3129.75
-1210.62	3652.76	-56.1973	-6370.12	-19195.4	-9779.61	-3266.41	-5573.17	-4966.12
-2474.81	2737.02	2483.72	-1405.07	-9389.34	-15073	-7856.95	-10150.3	-9378.05
3950.24	2293.14	3047.85	-3843.86	-9601.05	-12739.4	-4642.38	-7268.45	-7257.27
-4026.22	1919.5	695.094	-4283.79	-17825.1	-10883.7	-5969.02	843.92	-7238.39
-7335.86	4936.62	-1498.8	-4840.24	-17113.7	-12114.3	-7760.28	-14188.9	-2283.98
-2412.83	1762.75	1130.75	201.469	-13094.2	-16941.7	-6665.31	-11381.5	-10120.2
-3311.98	2024.49	631.359	-496.477	-9695.59	-12196.7	-8905.82	-4632.96	-10013
-1572.26	-762.02	111.717	-4099.4	-12557.3	-9655.09	-5909.58	-10675.4	-9244.51
-3377.95	3984.67	-2313.13	-3677.48	-13381.2	-8867.73	-4853.78	-6621.87	-8284.88
1157.35	2188.63	5089.03	-5521.72	-16368.9	-13009.8	-3478.31	-10294	-2452.63
-2766.59	3522.24	-541.459	-1374.72	-9291.22	-5080.48	-4858.91	-9040.39	-8725.59
1873.19	7684.4	-334.67	-4865.96	-11155.9	-6835.62	-3598.6	-2723.42	-4635.56
-1769.72	1524.87	-3854.63	-1639.46	-11534.4	-12911	-5908.53	-6798.28	-2676.86
-548.115	-4684.48	2564.03	-2135.86	-11543.9	-12118.8	-1355.68	-6594.1	-4707.02
-851.447	5870.79	-1520.47	-2833.84	-10817.4	-12022.4	197.24	-7941.25	-7013.15
-3305.86	7042.29	-3799	-1744.17	-16337.2	-8184.18	-1997.3	-10794.4	-8307.63
356.596	6689.63	-6303.97	4228.43	-8506.96	-10981.1	-4714.76	-3477.56	-7228.53
2071.63	-2743.62	-76.1621	3187.26	-9858.17	-12105.5	-8913.17	-8704.37	-3582.81
235.492	-1323.98	-620.912	4191.07	-10879	-9119.69	-4601.82	-8717.34	-6962.83
-3542.26	5871.85	-1032.11	1689.13	-13226	-10811.2	-8897.33	-8085.6	-4895.25
3854.43	3977.93	3064.51	-1894.52	-11652.1	-7728.21	-917.129	-8286.74	-4222.2
1046.65	8502.37	175.227	259.336	-15527.8	-12709.6	-3910.81	-16661.5	-4638.5
3206.51	1222.63	-5946.99	50.1016	-13615.3	-10849	-3834.59	-7678.48	-1459.41

-6327.07	3240.62	-9419.99	3309.09	-11199.8	-2984.41	-3242.41	-10255.3	-11336.6
-1502.92	3298.84	4175.82	9172.8	-8856.71	-11043.4	-7986.26	-7296.04	-9460.89
-4307.77	3731.57	-1462.48	2703.91	-6029.44	-9835.87	-9363.81	-4834.41	-10479.2
-1358.59	3411.6	898.939	4303.15	-5717.98	-10096.2	-6997.07	-1656.08	-6237.94
582.928	6238.38	6367.15	1661.18	-8003.28	-6564.67	2097.56	831.398	-769.209
139.684	5420.5	6190.45	6804.17	-13131.2	-7357.59	759.955	-3538.42	-4224.21
12462	8262.5	-5685.18	3798.68	-14825.6	-14425.6	5994.92	2670.1	-11780.8
963.104	5242.07	582.223	7495.18	-7617.76	-9833.38	2981	-669.227	3117.93
883.541	7306.48	-4456.13	3625.08	-11624.1	-11947.2	34.5898	892.707	-4021.91
452.064	3449.53	271.816	5246.8	-10159.6	-13518.5	-5956.57	-1268.59	-2710.72
-5931.76	5238.77	898.559	6734.85	-9826.71	-15449.9	-7964.14	-897.203	-8855.68
-1692.32	5843.8	-3573.08	1214.24	-5181.1	-9132.18	-2805.03	-7155.63	-10045.4
4753.64	9001.35	2116.7	8008.45	-4012.48	-15878.9	-909.869	-1317.56	-3888.26
3619.88	19743.8	10432.1	15943.1	-7547.48	-5815.81	5663	-12337.9	-814.727
1094.77	5142.88	8758.62	-4706.17	-3990.23	-3438.71	-7451.91	-7867.37	-8661.64
-5169.21	4304.41	-1236.31	1836.3	-7068.57	-9821.18	-10224.4	37.418	-11935.1
-1429.63	4429.31	-1382.92	-2419.91	-13660.5	-8750.88	-9028.06	400.355	-9166.41
-413.361	7757.47	2205.56	-2162.92	-14614.2	-5623.45	-6636.64	-176.605	-1482.03
-2649.35	6057.61	1683.33	4269	-10668.2	-5072.31	16.2402	-4929.26	-9006.58
-565.629	2882.06	-1860.41	5121.27	-6976.18	-963.146	-3459.12	-9812.41	-2106.44
-1114.6	19529.4	658.779	16951.3	-7033.95	-1246.03	-6854.19	-9630.85	3324.9
5414.45	20508.7	-6587.7	10232.9	-12047.2	-4832.91	-4103.76	-3901.72	581.752
-3499.7	11049.6	2654.26	3333.94	-9647.46	-13717.7	-3970.58	-842.512	400.547
4178.58	5444.8	4067.91	-1081.29	-6276.77	-6273.68	-10867.1	-5293.27	-728.465
3283.26	4684.7	4679.25	4640.84	-9494.19	-13031.2	-3562.4	-10719.2	-6304.38
14624.7	4629.8	-281.693	-3328.26	-7431.45	-10596.7	1371.97	-2233.98	-8597.55
10252.3	-555.709	-5876.63	-5891.84	-6431.52	-9668.66	2559.56	6980.66	-4114.91
5639.24	6028.3	350.596	-2762.22	-10369.6	-10279.3	1588.29	1897.94	-11017.2
6456.29	1837.22	-3972.75	-1013.31	-4701.29	-2617.09	294.184	-990.262	-4397.49
-3063.93	8554.16	3659.68	5803.53	-5784.25	7891.87	3315.97	-1541.11	13321
6401.44	3642.24	2712.69	2201.31	-8482.23	2203.87	6838.42	33960.4	-1421.53
8565.37	-1958.21	485.543	2603.39	-14340.9	381.609	8052.38	6999.67	-3642.55
1913.43	5601.08	2058.12	-1090.7	-14740.7	3797.33	580.406	-306.246	-9624.04

-924.461	6904.44	7241.66	-244.633	-10400.6	3430.6	-6379	-872.562	2243.62
-3998.7	16402.9	13118	6017.06	-15318.4	-1737.08	2453.32	-2595.62	18014.5
8492.21	8880.3	7562.58	1850.33	-12009.5	-4382.85	3061.97	-7151.25	-632.76
17995.3	4036.77	4129.64	2777.81	-14679.9	-6992.37	5879.84	-4845.33	-7784.11
-1874.64	15603.6	987.49	18875.2	-5270.77	-10833.3	-192.658	-6682.68	-5316.28
-6622.24	7551.16	3609.78	1740.94	-12584.9	-12091.6	-8229.42	-6538.69	-7092.08
-4446.14	-6232.34	5681.93	-3949.02	-10747.4	-5528.31	-2655.29	-5784.11	-8242.85
7077.1	1371.83	12.5898	-2126.38	-10739.7	-7872.1	-3556.53	-1405.39	-6673.75
-3441.85	-1116.27	-2562.58	-2318.06	-9418.42	-13882.6	-2800.89	-4852.44	-11310.4
992.631	2488.51	-6627.76	-1924.68	-7245.95	-5592.76	-8806.87	4647.11	-6209.89
1152.96	6725.67	492.078	190.648	-6358.32	-6017.89	-5514.98	-1100.67	-10609.9
1693.4	4492.57	-4416.04	-1144.48	-17542.5	-13551.1	-1921.61	-5389.87	-9130.13
-2468.33	-1670.07	1396.46	3075.01	-8536.57	-7303.61	-2618.43	-8555.59	-8264.99
-7241.78	-5146.06	-1410.18	1737.7	-5698.97	-3704.79	-5262.22	-7823.46	-9252.5
4340.67	-4085.91	-1723.01	-1460.4	-11469.3	-16953.9	-8661.06	-12335.6	-5377.22
-2211.02	2330.11	32.9805	3623.67	-18960.9	-10127.2	-6024.04	-11454.7	-7717.15
4421.87	-3884.38	-5104.95	-3818.83	-14917.2	-16516.8	-7067.91	-16317.3	-6190.07
497.744	-3138.29	4474.91	-9282	-12526.5	-6815.4	-5211.55	-6980.11	-4372.31
-6088.16	116.971	-1318.85	-2351.93	-11240.4	-8811.41	-5245.32	-12224.5	-12023.6
-6094.43	-1162.25	2354.79	-3010.69	-17911.1	-7285.82	-9443.91	-14366.4	-15053.6
-3359.05	1566.37	-2360.2	-3801.78	-18908	61.7168	-5907.32	-12679.7	-10081.9
-11258.6	5297.64	2964.67	3185	-10369.2	-3856.49	-4192.4	-11455.6	-1295.01
-6767.59	1131.24	3568.16	3350.15	-10298.6	-10350	-3980.13	-6341.17	-4249.49
-8617.38	2544.08	10236.2	-2621.99	-5566.33	-8118.03	-3124.06	-6884.72	-595.475
-5998.62	1567.65	-318.568	-10730.7	-8919.27	-12055.3	-4985.94	-15053.3	-7187.51
-99.6836	211.189	3261.01	646.512	-16005.7	-15242.8	1079.76	-10583.3	-7885.84
5977.29	1294.42	2129.11	-2133.81	-7294.51	-17452.3	-1336.35	-9366.88	-7979.74
9776.13	2006.62	1090.79	2088.82	-20654.3	-7055.25	-3582.75	-8697.93	-10424.6
2562.26	2731.09	1767.19	3094.25	-21084.3	-12180.4	-5527.43	-10450.2	-901.715
-7337.1	2121.62	1272.29	-8986.35	-20732.8	-15248.9	-7676.41	-9786.52	-4094.23
-4319.29	-3142.54	-1826.38	-5027.41	-13426.9	-10734.2	-7791.21	-10643.4	-10975.1
-5138.6	731.006	-3300.13	2551.31	-16916.8	-20210	-13103.9	-10160.2	-15568.8
-4085.61	-5578.57	-2151.39	1016.77	-8884.84	-17874	-8861.95	-3439.59	-9865.46

-1438.71	-3534.8	-5482.07	-3174.32	-10837.9	-18270.6	-6116.34	-4678.39	-17302.2
-3751.73	-3254.54	-5300.5	1854.07	-11107.5	-8112.47	-5806.83	-14210.1	-2987.35
-1461.41	-352.477	-3516.55	-4057.6	-15794.4	-12646	-9258.09	-11317.6	-10834.7
-2752.35	1228.36	-6516.93	-2177.94	-13649.9	-15686.8	-4116.43	-8063.6	-6966.63
3703.15	766.104	-3076.3	482.281	-16688.9	-17793.9	-10051.9	-13915.9	-11852.8
3325.76	1452.49	-4754.77	-2332.58	-12870.6	-11906.8	-6804.79	-15035.8	-17769.5
-4734.74	3135.91	-6504.11	-5634.39	-13294.6	-22027.3	2495.67	-10437.1	-10443
-4913.45	-6213.33	2047.1	-6648.39	-13723.6	-16641.9	-2837.53	-12547.5	-6759
3630.17	3303.37	-3931.41	817.051	-16284.9	-10380.7	-5456.47	-10854.1	-10193.9
-2625.75	5607.32	1099.48	-264.756	-16005.7	-18532.8	-9262.57	-9864.96	-10234.1
6643.03	2702.99	-11409.9	-3474.41	-20303	-16196.8	-2309.35	-4603.98	-9862.32
-8986.25	-2711.68	-10275	-2153.73	-18215.7	-13872.8	-7963.22	-7866.83	-3171.71
-7776.08	635.164	-1438.72	-698.734	-14130.1	-9288.09	2909.3	-9377.43	-11112.6
-6740.54	4192.54	575.26	-3757.97	-15995.7	-9214.86	838.361	-7226.91	-13050.6
-4299.59	-6852.64	-2696.63	-6534.88	-18141.1	-6984.33	-8068.93	-14080.1	-11893.1
5159.92	390.707	-2757.51	-1979.91	-7082.51	-15786.9	-4502.53	-9538.87	-6224.69
-318.818	-4742.51	-2431.84	-5012.99	-6854.8	-14559.9	-2707.1	-20783.6	-7758.52
7551.79	-1462.78	-3883.96	-6496.71	-18378.6	-10999.1	-9657.79	-9647.89	-5659.43
867.049	2399.8	-9113.58	219.406	-16416.9	-18867.2	-3224	-3450.68	-9544.84
-3370.23	-222.32	-5442.1	-4280.65	-20648.6	-15061.3	-14941.2	-6910.39	-3113.08
-3724.83	527.52	-1304.54	-3390.74	-15764.9	-13900.7	-8821.39	-9232.95	-5038.13
1040.75	-2895.06	-2100.16	-9363.34	-10609.1	-14899.4	-12906.1	-5683.98	-10781.8
-4148.49	1489.63	-4726.22	-4737.22	-15269	-5430.45	-17647.8	-12332.1	-11851.9
75.1621	-6598.48	967.367	-12830.3	-17576.7	-8272.62	-9998.44	-2801.95	-2994.86
-1712.92	371.803	-1246.73	-6667.95	-17681.3	-10527	-1779.88	-5014.77	-7213.24
-4753.33	-808.975	1641.47	-3098.62	-8549.32	-7393.54	-8199.76	-13587.4	-5400.54
-11306.8	-1936.7	2075.46	-3371.29	-17154	-8489.59	-13434.4	-9232.5	-5371.66
2145.05	727.537	1483.21	881.686	-16033.9	-16531.9	-6980.3	-14995.8	-7271.84
-3391.97	-1551.63	-4198.67	-1686.97	-13411.1	-14583.6	-6425.15	-9715.91	-11206.5
-2473.36	-8247.22	-3189.21	-3475.5	-13354	-14419	-6225.77	-7870.81	-11325.6
-1081.7	-525.59	2374.51	-5962.54	-16207.9	-7770.11	-254.105	-9797.92	-6880.79
-1081.24	-5016.94	-4875.36	-3112.01	-18354.5	-10021.2	-8217.34	-10143.8	-11150.1
-3722.61	-126.953	3223.46	-4930.42	-23843.8	-8305.83	-7829.67	-9786.16	-7429.45

-3121.97	-1309.75	-3272.82	35.4766	-9996.55	-10433	1106.42	-9433.33	-468.918
-6779.82	-5109.12	2808.72	-2338.75	-16833.9	-5594.49	-7130.59	-4105.81	-12713
-1070.86	-4408.75	2153.17	-671.879	-10175.3	-10761.5	-3956.31	-6590.12	-13040.1
-4304.1	1500.79	-1291.54	-1313.51	-16388.3	-13232.7	-3849.05	-17292.4	-5310.99
-7959.73	60.6172	-6483.47	-2086.73	-11507.9	-10606.2	-5691.57	-7036.59	-12217.1
-8725.17	5324.38	-2771.15	-2361.87	-19923.4	-9855.06	-5084.92	-9489.18	-7606.51
-8183.93	2031.84	-1521.61	1119.96	-9821.93	-13614.9	-6261.63	-14985.4	-8740.71
-2031.85	6684.2	-3589.61	-6861.47	-13785.6	-14821.7	-8423.95	-9969.06	-10927
-3519.81	-3400.25	-2187.95	-10901.7	-10702.4	-16705.9	-12141.5	-4980.6	-10560.5
-1230.42	8427.36	-9192.36	-3312.12	-17816.7	-12769.3	-9234.27	-6841.43	-10677.1
-1966.89	-13.7461	-5377.96	-1946.39	-14362.6	-7695.41	-2295.48	-13350.4	-9906.35
364.441	1701.31	-3212.5	2349.54	-11767.9	-16489.6	-9064.27	-15565.3	-7139.64
-8353	-478.83	-3272.28	-6363.77	-11597.6	-20597.5	-11456.3	-11538.7	-15331.8
-5299.95	-2311.68	-3709.96	-2595.7	-16690.2	-12950	-2169.44	-6808.07	-8914.79
-5918.32	-5204.99	-7184.68	4440.17	-20808	-7858.52	-2772.56	-11102.6	-13985.6
6685.01	2594.31	-8221.12	-4622.61	-16031.6	-10976.5	-11959.6	-15274.6	-9774.5
-5309.09	2515.05	-8797.8	-2955.6	-17459.3	-11802.3	-7775.45	-11126.4	-5888.33
-2963.86	707.57	-4604.71	-2589.25	-14222.4	-11910.5	-5017.39	-8905.11	-8259.21
-3602.24	-2258.12	-2505.05	941.67	-11988.7	-9748.07	-4095.27	-10860.7	-8320.74
-351.65	2459.87	-7400.84	-1646.79	-14870.1	-11738.1	-9377.74	-2218.73	-7882.76
-3568.73	1003.18	-3848.79	-239.451	-14362.6	-9439.32	-1872.46	-1423.27	-5825.24
-7373.7	-312.205	-1530.6	-2520.14	-18276.4	-13409.7	-3659.21	-10568.2	-11537.3
-1530.69	-5537.57	-5680.04	-3982.78	-11835.5	-8573.88	-10005.7	-12284.5	-13927.5
-10203.8	-415.85	-8416.54	-802.559	-23746.4	-5552.24	-13225	-7024.33	-4657.59
2300.87	-1181.15	-2623.17	-3134.39	-14222.6	-12846.3	-1020	-16477.4	-8865.71
1319.8	-5940.46	-3386.88	-5830.55	-18448.2	-9685.72	-8476.97	-9450.9	-7222.51
-4547.72	-6347.48	-623.113	-3273.84	-12078.5	-14429.3	-7520.43	-10260.4	-18697.5
579.006	6491.48	-5513.69	-4.34766	-15191.6	-16917	-2608.86	-8441.83	-14230.3
-6335.68	-84.4453	-7536.57	-10337.3	-17462.6	-15855.6	-5737.44	-9943.3	-3448.65
-498.76	4510.41	-456.053	-4192.05	-13743	-14526.3	-5240.35	-16745.6	-10639.7
-3583.84	149.355	-5102.34	-4348.43	-20317.6	-17262.4	-7323.86	-11864.9	-4830.71
-5448.61	-9302.49	-1229.87	-2830.46	-16755	-14743.5	-6916.69	-9357.8	-4901.85
-7657.48	80.9219	-669.039	-3790.53	-10467.1	-10357.6	-6546.44	-9488.24	-6613.04

-2761.25	4681.09	142.416	-2104.49	-20969.3	-13848.2	-6014.89	-11819.8	-5199.58
-303.5	-1364.62	-664.346	3164.6	-16596.5	-10375.1	-5628.73	-9363.39	-7944.43
-1219.67	1724.38	-1527.83	-4365.38	-11200.8	-13073.7	-3578.83	-10516.7	-12515.5
-5320.43	2137.56	-3835.61	-6850.54	-15273.6	-11346.2	-2735.89	-13438.5	-7054.87
-5521.9	3541.29	-875.377	-1107.16	-18259.5	-17470.1	-7001.89	-10354.5	-10396.2
-4295.08	-2438.9	-2149.79	5590.1	-19385.8	-16974.1	-10298.8	-14684.5	-12290.6
-9226.05	-622.467	1840.83	-824.937	-25631.8	-9519.34	-11513.6	-5153.66	-14777.9
-5044	5278.91	699.766	-3200.01	-15124.5	-14425	-10128.7	-6551.12	-8331.15
-8321.09	4093.63	633.986	-527.066	-18271.8	-9236.51	-7999.67	-5392.91	-9692.77
-966.684	2976.89	2102.5	-5255.32	-15353.1	-17883.6	-9724.24	-7246.81	-4289.34
-3408.5	319.086	-1597.47	746.848	-16982.5	-16105.4	-2700.67	-6905.61	-7759.8
4932.88	1154.87	-4216.37	-1865.86	-13474.9	-12832.8	-7430.56	-7428.14	-8498.56
-6555.78	-3053.24	-1543.13	-8805.07	-10348.4	-14584.4	-10900.3	-4025.5	-7295.11
-5620.33	1037.44	-175.873	-7719.63	-15107.3	-12502	-8404.12	-10365.7	-9605.26
-403.354	408.998	-3491.96	-1329.29	-19179.8	-9888.38	-4813.1	-16279.4	-14986.4
1111.76	-1907.76	-1437.87	-585.746	-15090	-10172.1	-6969.11	-10588.3	-8454.49
-3900.17	-523.633	-4906.74	-8585.68	-14619	-15361.7	-4352.36	-7085.6	-11707.2
-3209.81	-246.623	-6719.88	-903.494	-14944.7	-12293.3	-2643.55	-6944.29	-11600.1
-116.33	-2168.01	-4464.53	-1514.92	-16614.2	-15164.3	-4822.09	-13126.9	-14184.7
424.637	-3507.22	-978.637	-4194.27	-16709.1	-12591	-13251.9	-479.396	-11975.6
-2369.46	896.82	3141.67	-10214.5	-19071.8	-8560.59	-10870.9	-13740.8	-6077.76
-4899.55	3450.51	426.996	-2600.18	-15567.5	-13381.6	-718.611	-10930	-9479.53
-8619.64	2052.37	-6724.28	332.713	-13323	-13851.1	-9804.84	-8909.18	-11817.8
-11386.6	-2713	942.529	-6904.08	-24102.2	-17192	-8820.16	-9427.83	-7033.73
2119.01	2845.28	-6105.92	-10505.5	-13883.2	-12998.6	-5682.58	-17447.4	-7751.39
-549.135	165.324	1338.46	-2876.23	-16853.6	-11805	-6087.55	-8685.32	-13297
1543.64	-27.1699	475.863	-3560.19	-26916.7	-7098.14	-759.42	-10686.3	-9531
-3437.34	-1344.54	-13571.2	4463.54	-17276.7	-12205.6	-13576.6	-8663.18	-16626.6
-5565.11	4497.55	-8583.02	-122.234	-16537.8	-11939.7	-10652.7	-9173.51	-4085.58
-1373.3	-1659.52	-7444.11	-3504.12	-13477.7	-14095.6	-9466.4	-10564.2	-11294.1
-2799.08	353.428	-5106.07	-3259.78	-13872.7	-15832.4	-8058.58	-9386.42	-2673.23
-5726.27	5004.27	313.541	577.473	-16855.7	-10115.4	-13149.7	-10296.1	-7475.42
-9080.33	-1784	-1113.65	-630.645	-16725.6	-10924.1	-10219.3	-5878.96	-8817.32

-1734.79	957.535	-2097.24	-7985.01	-19149.8	-11899.8	-7952.62	-12502.7	-11422.7
-5381.68	2064.76	-1653.98	946.285	-17860.6	-12273.5	-13559.7	-11556.3	-9038.54
-1859.01	905.938	-7953.36	-10464.9	-10344.3	-12370.6	-8011.01	-18365.4	-11228.9
-4305.39	1230.27	-7829.89	-7228.42	-14280.4	-19448.7	-8195.38	112.635	-10234.2
-3664.26	-4033.25	-5210.07	-362.236	-14857.7	-18130.8	-7152.37	-9923	-4993.16
-3660.43	3392.92	-7438.88	-1818.67	-15327.3	-12054.1	-6095.46	-12352.4	-907.664
-2436.48	-2367.72	-10602.6	-3195.11	-11780.5	-10846.5	-2329.15	-10611.3	-3449.37
-5937.72	-1189.96	-3005.4	-3044.98	-11805.7	-16160.9	-3776.73	-10062.7	-7625.04
-3638.63	5481.16	1255.41	-3875.86	-7614.46	-5937.76	1283.06	-5700.88	-11159.6
-6666.87	1878.25	-2848.74	-2435.7	-23731.6	-9443.12	-4093.11	-10540.7	-9940.62
-2182.91	2231.33	-5784.96	-1827.03	-19751.5	-9525.98	-12003.5	-8650.08	-7065.9
3434.91	-2504.93	-202.262	-8181.52	-16708.5	-15084.1	-744.035	-3814.96	-1046.05
-6169.92	1463.56	-924.492	302.086	-13672.1	-7371.86	-14726.6	-10662.6	-15547.7
-4559.49	-546.594	-4477.1	-124.773	-17548.6	-18971.2	-5483.04	-9124.19	-9770.8
1782.45	-22.8066	-4106.69	-10387.3	-13947.9	-17946.7	-4445.78	-7550.94	-9587.97
-4561.59	1297.3	1.83203	-2056.88	-23357.2	-19126.9	-4132.49	-9860.08	-9339.9
-9848.41	-1040.2	-8105.78	-236.033	-20012.3	-13871.4	-3372.78	-8094.94	-7488.46
-5319.35	2553.39	-2977.71	2234.3	-10395.1	-13959.3	-6423.51	-12166.9	-8194.36
1061.17	-1242.37	-5017.11	-6774.21	-21340.4	-10403.1	-10931.4	-11516.8	-10568.6
-6798.11	1128.05	-5545.2	3595.86	-17122.9	-10477.3	-7495.5	-16794.5	-9384.35
-3747.1	3361.24	-3974.57	-4329.71	-15440	-12607.2	-7831.21	-15196.7	-8505.54
-4121.28	146.531	-4012.41	-2177.87	-20018.2	-11473.5	244.568	-7251.01	-14291.4
-2957.84	-4755.76	-6385.13	-7482.06	-14003.2	-15839.5	-3283.56	-11983.5	-5624.29
-5449.56	-3545	-5295.07	-6896.43	-13731.5	-13852.2	-8228.12	-13708.5	-6912.54
-315.545	191.025	-4450	2118.07	-14308.3	-5424.54	-2797.61	-16596.8	-2484
-4636.99	-521.814	-1224.23	-3079.44	-18414.2	-10928.6	-1864.86	-16231.2	-7025.29
-1167.84	3851.46	-1216.53	2220.38	-12481.2	-14924.3	-154.166	-11090.1	-6785.05
-600.838	-2267.27	-405.209	-4500.46	-11325	-9984.78	-4726.16	-7695.25	-7949.51
1891.41	-3248.67	6274.53	-10234.7	-11035.2	-10864.1	-6657.06	-12731.6	-9422.95
-7740.01	9087.79	-7125.26	-8403.44	-15285.1	-9083.94	-7635.66	-13419.1	-9296.22
-817.133	3733.08	-3228.81	-6844.52	-16472.2	-13498.4	-8879.93	-16980.2	-8636.84
-2135.01	5714.51	287.109	-2795.63	-14008.1	-6000.04	-345.641	-13161.6	-9343.85
-9963.42	-53.709	-5286.13	-3721.14	-20151	-10708.1	-9070.33	-9434	-8323.68

-4662.03	4428.19	-1943.7	-1545.72	-18088.9	-11302.4	-10436.3	-11868.6	-11009.5
-5832.86	1085.09	1606.8	-862.154	-10530.5	-14644.6	-4390.98	-8177.72	-9372.76
-4864.83	3833.56	-5966.48	178.35	-17103.5	-11639.9	-2868.02	-1279.99	-2523.95
-1180.41	-407.264	-3445.07	2692.77	-13062.8	-10442.3	-9459.45	-9404.8	-10178.8
-5460.5	3832.64	-1715.38	-11074.6	-12128.9	-10472	-6306.92	-14109.6	-8950.58
-4446.45	5370.27	-7516.18	-5619.24	-15806.9	-12960.6	-1799.03	-10202.9	-12448.3
-2890.75	-3183.45	225.666	-2372.41	-15791.1	-9035.55	-5482.76	-12020.3	-8983.86
-6262.95	-6653.35	-1994.23	-7618.48	-14215	-10680.6	-6877.45	-8939.5	-13121.9
-2041.79	-2459.45	-7737.92	-3430.53	-9893.37	-10303.1	-5321.84	-8662.07	-13691
-6303.5	-278.141	-83.5742	1307.84	-16784.9	-13378.7	-3151.49	-8038.06	-3217.6
-4101.05	3510.44	3458.91	1441.83	-17231.6	-12297	-7167.23	-9577.28	-8606.12
-601.611	1423.43	-1576.32	-942.119	-16368.9	-12047.1	1917.79	-14663.7	-11460.2
-3377.73	-2037.49	-4851.8	2448.19	-15795.2	-15202.8	-6108.13	-2665.78	-7997.24
-7187.24	-23.7852	-8053.9	-957.52	-15088.9	-13566.5	-6801.89	-8140.51	-10386.2
106.945	4085.24	-9342.46	-5564.01	-20620.6	-18614.6	-5901.36	-11935.5	-15722
2865.28	5711.38	-5190.08	-5635.41	-15171.4	-12498.4	-69.2109	-18604.1	-5541.93
-5265.27	1212.62	-3402.24	-7015.76	-20920.2	-14833.7	-5218.94	-14372.2	-8919.49
-5339.52	3841.87	1321.95	-3048.89	-18807.5	-7186.73	-13671.1	-16130.1	-761.393
-10207	-22.1641	-3991.48	-734.893	-16257.7	-11065.1	-6292.99	-7349.44	-8441.9
-8999.04	-4194.86	-4286.8	-190.781	-14540.4	-15334.3	-7900.26	-9599.91	-16096.3
-3424.09	4335.93	-3624.75	-4615.21	-11710	-11502.4	-2440.4	-9334.95	-8601.21
-3426.5	-5833.12	-4391.56	-7345.71	-13338.3	-15022.6	-13084.2	-13046.2	-9766.65
2350.47	-5975.83	-3118.56	1090.92	-13610.4	-14300.4	-3229.8	-9431.72	-11252.5
229.098	-1382.35	-1533.76	-2528.04	-13459.9	-13379.3	-8089.91	-7693.06	-6464.35
-7788.13	-3208.79	-5947.39	4454.27	-16612.7	-11474.4	-5620.58	-3945.32	-13043.1
-5663.6	-4089.57	-1601.54	-3501.19	-14343.9	-16431.1	-3877.66	-11386.2	-7621.97
-1318.07	6634.78	3507.21	-4077.06	-16132.4	-4840.34	-1554.94	-11923.6	-4708.4
-2410.48	-1062.91	-11559.3	893.105	-23906.1	-10833	-4663.3	-16925.4	-6130.54
2232.05	3011.82	-965.904	-4762.98	-14869.5	-5777.08	-7472.61	-9792.38	-9113.77
-1124.88	2904.79	-4221.74	-2118.59	-12372.4	-12686.1	-6957.25	-9364.46	-10145.1
-2302.69	-5168.35	-2879.08	3158.29	-14460.5	-14342	-5366.2	-16152.8	-14998.1
-9915.06	7615.43	3052.63	-2461.61	-8187.15	-13626.7	-8487.9	-9599.33	-9145.35
-9344.16	-667.885	-1169.32	5940.34	-16516.3	-6637.02	-3879.39	-6661.67	-8693.84

-2171.58	2602.87	-3469.19	-5270.83	-14110.8	-12173.5	-8339.21	-9679.33	-10635.6
-3171.66	4128.92	-5398.62	-957.732	-17066.3	-8426.98	-4723.85	-4280.98	-8709.68
-2457.52	-4443.26	-11404.1	-4439.77	-11392.2	-10606.1	-6616.75	-12430.8	-12930
-4201.77	-2975.74	-9617.39	-7539.75	-13719	-18422.4	-5315.66	-5976.31	-11530.8
2006.62	-3423.49	-1959.13	4271.43	-13008.4	-11034	-5448.93	-10059.8	-10485.9
-6093.85	-2948.31	3902.51	-3839.63	-14170.6	-13696.1	-7230.19	-10935.9	-12532.8
-8990.42	3417.7	648.225	1443.1	-7413.97	-14633.9	-8238.13	-8237.64	-10424.7
-2493.78	-3611.87	-4196.95	-3961.18	-19540.7	-11072.8	-12042.5	-12877	-10872.5
-4396.75	-3074.33	1993.04	-4588.71	-15900.1	-18524.4	-11606.1	-16058.7	-11876.9
-4450.93	4880.05	-3381.45	-7418.13	-11863.9	-14471.4	-2718.49	-9791.84	-9872.07
-1675.14	-3200.43	-3109.74	2068.69	-20008.9	-13277.5	-6953.08	-10571.6	-10130.3
-2628.25	-4904.15	-4970.74	-1511.89	-15344	-13094.6	-1926.4	-10153.1	-5192.01
-2619.67	-5175.4	-8204.23	-4582.96	-4584.93	-10443	-10814.7	-8941.25	-9565.8
-2482.13	2242.8	-7984.35	2626.97	-16410.8	-14367	-7108.69	-10170.3	-10711.9
-5627.5	6051.82	-7474.75	972.436	-8724.31	-14068.7	-5876.46	-7636.67	-13218.8
-7425.99	-928.17	-3386.12	-5958.48	-17046.6	-14229.6	-5041.09	-4647.31	-9067.63
153.025	4056.77	-6897	-8695.25	-15721.1	-12008.2	-10178.6	-6584.3	-11529.2
917.084	-1832.97	-5567.32	1209.9	-17689.4	-11284.6	-6647.24	-6375.73	-8279.41
-286.635	-4898.14	-4877.63	-3885.23	-6270.75	-15444.7	-3158.63	-6360.4	-9010.08
-4520.92	-4092.17	-2183.84	-1931.98	-16073.9	-16204.6	-8680.09	-3305.88	-8908.66
-6600.25	-2999.99	-4621.32	-5070.51	-16451.3	-13654.7	-5967.63	-15753.7	-9671.67
-4519.4	1643.61	-7629.32	-5800.46	-12691.7	-9765.53	-12057.6	-5168.78	-14182.6
-583.666	5109.51	-7049.56	-866.594	-18315.4	-10407	-3676.27	-13219.8	-7321.54
-3744.5	99.0156	-987.441	-1310.3	-16644	-7097.85	-10583.7	-13568.1	-6800.92
-7291.79	-6114.8	-5357.84	996.119	-17401.6	-10968.3	-3405.58	-16401.5	-11135.6
-11058.1	-4536.87	-3536.52	3091.93	-19990	-12782.3	-6272.76	-17779	-7215.49
-6129.3	-2381.01	-1127.71	-136.191	-24162	-13989.6	-6907.88	-13723.5	-12040.6
-5129.25	-189.57	-1660.45	-4289.43	-24366.6	-14735.5	-1468.6	-6529.92	-12294.9
-4589.95	3986.79	584.49	-676.531	-17853.5	-12173.2	-8054.81	-14608	-11942.7
-7743.5	5790.22	-3792.29	1399.37	-18058.2	-9095.23	-3767.4	-8920.54	-14425.1
-5827.89	-3161.36	-7482.51	-834.842	-23635.1	-10322.1	-10952.1	-9655.67	-10084.6
-7815.58	-643.664	-482.529	4633.23	-16496	-7279.65	-8569.98	-18036.4	-12334.4
1624.96	-378.262	-3536.36	1273.3	-14583	-10640.9	-6513.57	-16110.9	-8132.93

-133.473	-3146.76	-3474.09	-6104.99	-13549.5	-10455.8	-5204.91	-13962	-7409.81
-6179.4	-6850.6	-1465.04	-6704.32	-18018.5	-19088.5	-9609.13	-12144.5	-9074.86
-8092.04	-1817.39	-1044.37	-766.188	-18006.2	-11242.5	-10309.2	-12630.3	-8601.97
-8018.38	-4074.36	-6185.08	-8562.07	-12368.3	-22425.1	-3998.85	-21237.3	-15599.6
-1792.45	-3738.87	-10234.4	5629.93	-15552.4	-16241.1	-3504.29	-12053.1	-7493.47
-5675.31	2453.38	-998.086	3692.29	-12644.3	-11334.2	-9695.07	-12077.6	-4619.96
-6844.1	1919.5	-3313.65	-475.508	-10199.3	-12312.6	-5512.91	-13540.2	-14540.2
-1153.64	-1141.36	-3083.26	3851.62	-18971.6	-14409.8	-5744.42	-14135.8	-14354.9
-4755.74	368.754	-9237.81	5516.51	-13653.6	-13356.9	-1226.18	-9039.54	-7117.92
-3.07227	1321.3	-2582.53	-2152	-14849.6	-13110.5	-12401	-11815.5	-4996.31
-3312.13	-1953.2	678.408	2268.03	-13450.2	-14875.6	-4258.82	-10737.3	-15523.2
-3457.26	-4051.7	-3043.15	-429.691	-18676.2	-15764.9	-12761.2	-10930.9	-6069.64
-9013.62	7129.65	-1521.43	-8986.04	-14385.7	-9244.53	-9556.98	-10608.3	-14133.6
-3308.3	2326.11	-1428.17	-5821.04	-13144.5	-16237.7	-12370.6	-6513.87	-9725.07
-4571.79	-3309.5	-3226.96	-9669.25	-17143.2	-10580.2	-5932.59	-11161.4	-5118.16
-4023.25	5316.1	-37.7832	-5464.66	-18057.2	-12693.8	-10350.3	-17158.4	-8895.66
-3112.83	4034.31	-734.547	-2181.46	-17739.4	-7497.9	-2209.84	-9238.58	-14406.2
689.361	2489.35	-7219.2	-6764.87	-12053	-10318.4	-19804.5	-12390.3	-2935.58
-5432.08	-6205.21	-1819.2	-260.809	-17898.3	-15324.8	-9362.36	-3368.31	-8218.43
393.381	-3447.6	220.748	929.828	-21878.7	-9480.34	-3261.87	-12907	-7507.97
-2969.76	43.6699	-5212.45	-4660.04	-18047.1	-9159.33	-7666.24	-10107.4	-2538.13
-4985.97	116.914	-2347.35	-3489.39	-12853.9	-10376.6	-4098.82	-8260.63	-15230.8
-2202.43	-555.15	-371.164	-428.281	-5889.49	-14334.6	-8152.74	-7350.6	-14215
-5160.16	-4798.86	-354.814	4143	-10033.8	-15212.9	-7470.17	-9624.01	-9437.37
1007.05	-7902.09	-2773.21	-2379.95	-13224.8	-12232.1	852.541	-9661.48	-4115.67
1253.99	-2089.06	4147.34	-5284.12	-15080.2	-9120.42	-7298.9	-10207.2	-8534.46
-7075.62	-4721.64	1035.75	215.424	-14543.6	-10725.1	-10657.7	-14455.1	-15157.1
-2631.62	-1187.76	-2822.57	3268.74	-17958.2	-14404	-4334.32	-8953.72	-20286.8
-8434.85	-1464.29	-2352.27	485.842	-24683.7	-13873	-4985.42	-6867.46	-11013.3
-11535.6	1111.74	-11143.7	-5765.39	-17617.3	-10425.7	-9316.45	-14516.1	-15783.9
-9220.28	1854.12	-8799.22	-10810.3	-15082.1	-11662.1	-13909.1	-11545.4	-12120
-7128.9	926.828	-4388.73	-2844.23	-10445.1	-12761.7	-12786.8	-6776.23	-9412.19
-2085.65	5653.27	-4487.97	-371.859	-12306.9	-9587.54	-4482.39	-11874.8	-12217.8

-7736.24	474.133	-9203.92	2762.06	-19305.3	-11993.7	-10920.2	-16116.6	-8227.77
-1273.57	114.678	-3505.21	685.033	-23385.9	-13412.3	-8204.09	-18065.8	-10820.6
-3305.8	2388.03	-13589.1	-2142.27	-14177.4	-12594.7	-5536.27	-17466.1	-11335.5
-10621.3	2582.45	-5289.41	-4116.88	-11569	-12878.9	-7080.92	-19838.3	-9325.9
-5939.32	-5341.02	-8807.64	-8207.77	-14334.1	-21246.6	-10884.3	-8426.22	-15158.1
-6135.33	4273	493.291	-6496.6	-18727.4	-12930.4	-7649.28	-8653.39	-10691.5
-4723.44	-1231.8	-6553.76	-4172.49	-14839.1	-17175.8	-10476.5	-6769.57	-6598.97
-4341.44	-3400.19	-4829.75	-2541.46	-13766.5	-10264.8	-3507.38	-10222.5	-12423.7
-9225.13	-4853.56	52.541	-4994.95	-14783.4	-3004.48	-1597.3	-6561.76	-11808.3
-4890.07	-2566.31	-4789.31	-7367.94	-18632.6	-16014	-6295.31	-11496.1	-7819.95
-3234.7	-4741.67	-8629.35	-3054.49	-17934.7	-17728.7	-12663.7	-16080.9	-7067.08
-756.133	-6359.54	-4027.33	-6587.78	-16838.7	-14162.6	-7851.91	-15190.4	-10256.1
-6781.43	-1912.68	-4803.52	-4040.79	-19961.1	-16445.9	-5887.92	-5618.15	-15948
-2670.85	3017.02	-3157.44	-11130.7	-12803.5	-14949.6	-10642.3	-13247.3	-11723.4
-3245.11	1129.84	-5856.73	-14115.1	-18335.1	-15846.9	-7333.25	-11214.3	-9824.72
-1986.1	1911.17	-4337.48	2333.47	-17107.5	-18240	-12098.9	-14161.6	-7934.74
-7529.58	897.016	-5523.08	363.268	-8609.34	-6884.53	-12301.2	-6812.99	-12711.6
-6671.01	-8966.69	1471.36	-5604.93	-12957.2	-17018.5	-10110.2	-6130.68	-10953.9
-416.264	-7290.4	-6175.47	-6297.03	-13538	-20208.6	-10060.6	-9528.22	-8601.35
-6969.65	-2942.83	1493.68	-2188.79	-19904.6	-12942.1	-6736.06	-7396.71	-3247.59
-7857.25	-1341.57	-8191.72	-4323.4	-14013.7	-14595.5	-4538.12	-11268.8	-10997.7
-6946.14	5832.2	-9613.05	-3922.87	-7829.66	-17819.8	-4267.09	-3126.92	-15556.1
-797.828	523.744	-1079.76	-675.227	-12627.1	-15388.7	-8399.08	-11725.1	-7962.62
-8426.66	928.785	-5899.65	-2372.72	-16245	-15026	-6433.06	-9649.86	-6353.08
-4442.56	-4777.35	-4777.88	-4602.38	-17545.5	-16127.4	-6010.38	-15496.4	-11637
-5385.14	-2357.54	-6601.15	-1647.36	-19249.8	-18774.6	-12162	-8983.33	-11580.6
2567.78	-4283.94	-5531.88	-32.3672	-16787	-13323.2	-2064.65	-7741.04	-14055.6
-3909.5	-4743.54	748.947	229.697	-18282.1	-15539	-6260.21	-13557.3	-13828.1
-9408.28	-1350.74	-4097.42	2238.99	-20493.5	-15263.3	-4688.17	-11430.9	-12329
-3070.45	4807.14	-2080.83	-8524.5	-18229.7	-15252	-8474.68	-4978.5	-16620.2
-4079.37	-445.689	-10304.1	-3103.13	-13851.6	-16108.7	-10057.1	-9372.78	-14047.9
-740.752	-5290.2	-3410.42	-2834.22	-20025.7	-10941	-15151.8	-9154.82	-13828.3
-3105.17	-3767.23	-3423.17	-715.615	-16087.5	-15667.1	-14898.3	-11066.2	-13659.4

-4099.94	-279.148	-1826.7	978.674	-13379	-11593.4	-12367.8	-10537.7	-16088.1
-6486.84	-4391.33	-5858.84	-2224.28	-17868.8	-12390.7	-4620.32	-14525.2	-11706.6
-1341.51	-2295.23	-3928.36	-8622.09	-15356.1	-13032.3	-9800.91	-11433.1	-14507.7
-1208.24	-4201.94	-1856.97	-4021.41	-12917.9	-12423.1	-8813.48	-11939.7	-7071.3
117.816	-7734.74	-6747.03	-5147.87	-15925.3	-13123.8	-3526.34	-10717	-5964.42
-136.592	2618.67	-5027.42	-4179.37	-14864.6	-7549.63	-3380	-10987.9	-8382.45
-6444.86	-1374.43	-1776.84	-9525.46	-16579.8	-16986.6	-3099.44	-7881.55	-5470.4
-1891.96	-2737.94	-4699.7	-6295.4	-19892.8	-10153	-13872.4	-17215.8	-8437.68
-1837.95	5174.17	-7229.62	-6040.39	-18617.5	-14597.8	-11965.9	-15842.8	-4503.96
-7630.92	6805.94	-4912.87	-5512.21	-14158.1	-10935.5	-6277.11	-16386	-10379.4
3003.8	2331.28	-4604.64	2099.92	-9400.32	-5805.05	-9497.76	-15565.2	-9882.72
-354.924	-4061.24	-1194.09	-3898.62	-13976.4	-16073.9	-13206.9	-11012.2	-7676.82
-7301.3	-7142.54	-9318.61	-6072.6	-17025.5	-14560.8	-14088	-15060.5	-7567.98
-7240.92	531.994	-5702.36	-102.711	-16871	-12203.7	-3291.37	-11954	-15327.2
-2520.15	-286.111	-8439.4	-4852.75	-20043.7	-10259.5	-5293.11	-9242.61	-11106.4
-2540.38	-2051.84	-7294.43	-615.139	-16662.1	-17649.1	-4233.05	-10582.1	-9518.83
-3300.09	5826.16	222.746	-2057.09	-19169.5	-16342.4	-2924.46	-9117.57	-12854.7
-782.047	-4660.59	3561.94	-8850	-16177.3	-11561.3	-2732.55	-10542.6	-13437.1
-5568.78	-2531.77	-4763.75	-5690.75	-5929.36	-13616.7	-8088.43	-12660.1	-4293.21
-268.383	-2857.28	-5472.64	-3678.5	-16190.6	-13407.6	-7653.55	-12242.1	-9790.72
-4292.45	-6774.95	-5029.96	-3842.87	-12276.3	-13766.8	-10679.3	-11446.5	-20156.1
-877.236	-4271.21	-11573.8	1904.18	-19747.6	-13168.3	-4460.76	-8988.11	-9211.06
-3732.55	-567.455	-4414.09	-6902.18	-15519.8	-12960.9	-4439.17	-8380.7	-10709
-784.084	-3005.3	-5910.25	-4420.18	-14943.7	-7201.3	-6279.12	-8323.19	-12818
-10161.7	-3208.54	-175.762	-2485.04	-14355.6	-6139.92	-11855	-6668.96	-12815.1
-1854.16	3460.52	-8002.34	-1554.53	-18197.3	-12047.4	-9503.66	-18560.1	-7781.66
-4561.25	2230.04	-4899.71	-2919.48	-16629.1	-9202.6	-6223.82	-15538.5	-13608.4
-3241.53	-2571.43	-4196.63	-3367.16	-16865.1	-13941.7	-3588.41	-8903.19	-16831
-2851.9	-667.885	-6132.42	-10641.3	-20108.6	-17880.4	-8218.48	-16438.7	-10453.2
-4728.07	2195.98	-12196.3	-373.516	-14875.8	-17746	-9913.7	-13756.6	-9062.89
-4180.52	-4112.47	-9547.7	-2365.92	-9311.8	-4938.26	-7593.03	-9963.25	-6515.16
-7193.48	4099.89	-2216.13	-1644.86	-12061.1	-12449.1	-6889.13	-12155.2	-10581.9
-7445.69	-845.389	-8204.27	-2753.62	-18064.6	-7413.44	-10446.2	-13927.2	-17270.6

3914.78	-635.359	-5035.44	-2412.02	-12791	-17610.2	-8409.22	-14195.2	-11896.8
-10823	-572.238	-403.098	-294.713	-13795.8	-9951.96	-11771.2	-9025.32	-14174
-2801.22	-9188.19	-2817.39	-13179.5	-16874.3	-11137.8	-4700.06	-11745.1	-4452.52
-4273.21	-7691.05	-8969.76	-7292.63	-8275.1	-18065.1	-5791.75	-11227.1	-14320.9
4560.24	-1809.82	-11558.1	-4441.89	-12567.8	-20905.5	-8637.73	-9225.43	-13561.3
-3596.34	-2703.21	-7032.96	-494.326	-18153.5	-13729.7	-4701.2	-10405.5	-14277.5
-1764.04	1177.24	-4952.77	3244.35	-12407.9	-15749.2	-6879.09	-7777.93	-11524.3
3184.92	-582.139	-7228.88	1049.58	-18626.9	-13935.3	-6329.2	-13350.8	-14298.4
-6064.01	-1133.39	-14450.9	-529.287	-17703	-18887.7	-9732.36	-13464.3	-12213.9
-10203.9	-2054.47	-7968.61	1242.25	-16887.7	-16338	-4680.07	-8366.61	-10977.8
-11984	2447.48	-2565.64	-5845.78	-15159.9	-11890	-7306.87	-9191	-8220.78
-50.2969	-1011.43	-5935.15	-1560.63	-19989.6	-10315.4	-10283.5	-1480.11	-6228.08
-3743.92	1009.33	-4715.38	-4136.65	-15393.5	-15139.5	-9845	-13724.8	-4535.5
-1167.35	-2532.33	-6296.4	-9978.93	-17811.7	-19237.4	-8001.99	-13837.3	-12613.9
-5086.93	3212.04	-10582.3	-3424.29	-18202.4	-13512.6	-6562.59	-14184.2	-12539.9
-2827.47	-413.221	-9545.25	-4097.88	-17049.5	-11375.8	-8627.69	-7732.86	-12463.3
-6257.82	4335.76	-7179.88	-965.768	-19127.7	-8427.83	-8887.9	-6790.54	-4488.91
-3630.41	1324.65	-1973.79	-3288.01	-19889.7	-11814.1	-11424.9	-12050.9	-10503.2
-239.195	-439.789	-7891.85	1902.14	-17101.7	-11238.5	-9412.96	-7594.68	-3221.1
-3488.52	5404.14	-5643.61	-5577.58	-27882.3	-11707.1	-13307.8	-11287.2	-11977.4
2559.45	2081.9	-8619.6	-9385.55	-18024.4	-15222.3	-6809.92	-16674.1	-13251.2
-3550.54	-1655.08	-5130.82	-2396.3	-18117.6	-15426.5	-7477.39	-5570.35	-11801
-2832.04	-265.053	-5705.41	-6876.34	-19086	-8334.35	-2285.04	-9175.75	-6905.62
-5289.79	-4814.27	-586.889	-8540.96	-15138.9	-10409.6	-12176.3	-10616.6	-8523.12
-3311.61	-9893.12	-1133.26	-5073.96	-12720	-11206.1	-10995.6	-9820.68	-10117.2
-671.598	541.42	-2782.82	-2710.1	-15261	-11421.8	-11918.6	-2288.85	-6470.65
-5417.2	-6026.9	-3799.04	-3971.65	-24764.4	-17093.7	-13529.8	-13568.3	-12656.4
-4680.72	-3462.33	-3632.93	-317.832	-17371.2	-11390.6	-9937.95	-8384.27	-17916.7
-11128.6	-1528.44	-6410.49	-4586.07	-10751.3	-11630.4	-8642.4	-5154.91	-9462.27
-1799.06	-520.275	-680.672	-3216.26	-16429.6	-14993.9	-5613.96	-5198.25	-11190.1
-4151.24	444.627	-6111.31	-4105.32	-12342.8	-16364.7	-9079.07	-6460.07	-7642.58
-7412.12	-836.271	-3852.04	-5390.58	-6986.82	-14904.3	-8994.39	-10838.7	-16035.5
-3179.04	-1391.04	278.553	-7272.82	-12379	-18020.6	-15636.6	-9938.52	-8750.7

-3980.69	4127.38	-7209.07	-4639.49	1853.94	-17249.1	-14197.7	6272.37	-6998.32
-3818.38	-2961.54	-424.68	2381.96	-20743.4	-16579	-6572.6	-15226.5	-9283.39
-5567.67	-452.094	2389.51	-1631.04	-13470.5	-18431.1	-10858.1	-7588.33	-13877.5
-6227.43	-2231.64	777.535	2261.58	-16668.5	-14232.6	-4905.5	-9017.09	-7771.52
-10345.4	268.326	-10194.6	25.2207	-10774.6	-17351.9	-1693.62	-14200.5	-12292.3
845.768	-1390.93	-2050.04	-3800.93	-23003	-15810.1	-9555.68	-7646.28	-14750.4
-3203.26	-3851.55	-4183.02	-3056.57	-23629.2	-14121	-13601.4	-7366.21	-10811.4
-3693.2	179.84	-8910.92	-3733.12	-15552.3	-12276.3	-4336.36	-12471.4	-5848.97
-7476.64	103.994	-8113.25	-2841.1	-15016	-15174.7	-6549.71	-11816.1	-8600.07
-4717.23	-1401.64	-2117.7	-8742.9	-19710.3	-15278.6	-10664.9	-12316.3	-8418.25
-7713.41	284.937	-3174.14	-1452.89	-24733.6	-19627.8	-8964.36	-15627.2	-14679.8
-1603.95	-2379.92	-4077.97	-784.938	-9631.76	-17705.3	-2003.82	-10194.3	-12348.4
-8749.58	-6328.32	-5197.38	-5012.47	-19466	-10363.5	-14323	-8497.86	-12255.5
-11219.3	-3150.42	-8361.86	-11367.2	-22852.7	-10794.3	-9709.32	-10361.8	-14137
-1481.09	-551.934	-9178.25	-4188.14	-14869	-19006.2	-11770.6	-10891.7	-11047.3
-1584.56	-4551.94	-5905.98	-5602.78	-10244.5	-15620.4	-7629.39	-8532.92	-6963.59
-2160.49	3165.69	-5296.91	-5054.65	-16598	-20333.3	-6966.43	-9650.22	-14584.8
-4471.68	-458.135	-3055.84	-5514.57	-12555.9	-10130.5	-9647.96	-12206.6	-13197.3
-7307.91	-1243.74	-8028.77	-3643.57	-16677.6	-6121.78	-4984.06	-17093.8	-10906.8
-3298.29	-1489.4	-3649.88	-74.0586	-15929.9	-6149.28	-10742.4	-11382	-7474.97
-5764.08	-2657.04	-2839.71	-6252.62	-14265.9	-11091.4	-4137.75	-16868	-11748
-3191.89	-4110.94	-4195.49	665.156	-14976.5	-18418.7	-4533.64	-8933.64	-5930.73
-7709.68	5797.6	-4647.31	-4452.71	-16486.5	-12223.1	-8250.55	-12000.3	-14131
-5611.5	3.45508	-10238.8	-4137.96	-14061.9	-18659.1	-11211.4	-8208.35	-8303.59
-7625.39	48.3398	-4421.57	-864.268	-18513.6	-15811.8	-6571.55	-9844.56	-9121.41
-4069.42	2062.89	-7955.92	628.363	-15649	-10614.7	-920.063	-15101.7	-11457.6
808.025	3719.57	1446.7	-5600.17	-19486.9	-12997.6	-8861.92	-12034.8	-12433.9
-6757.31	298.25	-10082.6	-4766.83	-12452.7	-8424.43	-12288.7	-10672.9	-7837.03
-6526.31	-576.77	-5913.79	-5095.22	-11667.1	-9623.52	-11205.5	-12455	-13498.8
-6728.03	-2459.09	-5466.71	-1418.21	-13685.1	-11620.7	-5539.57	-11921.6	-11798.6
-5169.86	-284.826	-7667.32	-3579.04	-13556.1	-19064.1	-9108.42	-5226.73	-12166.9
1658.56	884.318	-3414.46	4597.18	-6475.83	-12619.2	-7507.61	-9307.67	-10257.9
-4935.59	-1259.66	-3090.13	920.877	-16711.6	-12942.2	-4096.1	-8137.38	-15509.7

-8743.71	2161.19	-7835.91	-4122.52	-14315.3	-13340.7	-10874	-10719.8	-15274.8
-3474.9	-4089.12	-6075.13	459.445	-15883.9	-10822.1	-11038.5	-9732.63	-14887.7
-3100.47	-1120.52	-5421.48	-685.236	-14435.3	-12174.9	-9256.67	-10619.9	-12232.7
-5680.65	2107.61	-2104.14	-4712.34	-12588	-8827.62	-9111.75	-5033.55	-8866.88
-7104.12	-707.541	-700.322	-1302.44	-20006.5	-5485.62	-9394.55	-3725.26	-10072.5
-2644.66	2005.44	4767.35	-763.158	-18095.7	-10585.7	-9441.44	-13746.1	-5980.85
-4724.45	2319.37	-5656.21	-5606.45	-9807.51	-12449.7	-3407.84	-4703.48	-5744.17
2337.22	-5672.03	1707.61	1725.22	-12295.5	-9518.46	-7763.49	-8395.63	-14222.1
-1611.71	-737.939	604.59	-165.795	-7478.34	-13399.9	-7610.6	-4237.11	-9567.01
-2253.95	1027.17	-609.998	-3653.26	-9888.59	-13777.4	198.26	-9634.47	-5953.49
-3757.17	1152.13	-3617.39	-471.561	-6969.97	-18494.8	-12590.5	-5559.13	-5041.55
-466.824	-7.28516	-2423.21	-6807.83	-11945.3	-12833.7	-6250.36	-5632.96	-8948.99
168.562	525.172	-659.338	747.834	-6809.49	-10724.7	59.3652	-6045.04	-8606.71
1740.44	-1763.34	-2301.79	3457.04	-11345.8	-13905.1	1690.08	-7905.38	-9515.96
-647.971	869.412	-2327.12	-1646.58	-9965.75	-7879.5	-6095.86	-5793	-6698.65
-3885.75	4499.45	-354.521	301.451	-12487.1	-10274.3	-1247.53	-548.709	-8739.7
1496.16	596.318	6025.19	1027.52	-5556.73	-10319.3	-4836.17	-2923.27	-2724
-3688.94	2763.93	-2704.58	-3732.53	-3512.37	-5277.49	-177.027	-3536.63	-7703.84
3661.18	3386.1	-4362.12	-2821.43	-4384.15	-9808.69	1831.69	-1496.57	741.721
5344.96	2355.31	-2768.94	1820.9	-7816.44	-9601.29	-4250.47	-1194.09	-1349.38
2158.45	5648.04	5589.06	2539.08	1005.75	-7653.4	-1590.29	3186.06	-8160.15
6436.71	7994.27	143.045	-726.893	2957.86	-7218.09	-354.537	1720.45	-8980.92
5922.65	4561.79	4546.37	2310.95	-2748.03	-6509.14	-2785.96	2128.46	-5174.82
3135.04	5657.15	-3446.87	570.881	5390.65	-6789.92	-2185.47	776.574	-4973.45
6840.98	2377.6	-5133.11	1702.95	-2233.74	-3479.2	-2690.32	2665.85	-1250.43
-188.047	5848.03	6568.48	2342.95	-1786.82	-3013.61	-850.863	3253.94	-3041.28
4201.34	6468.27	3807.38	2822.71	3980.62	-543.635	-1221.24	4991.27	-669.354
8703.59	3945.77	5615.36	2284.87	143.617	-2851.32	1224.52	2631.01	-908.32
2083.8	8591.44	3458.64	3770.7	8783.17	-1162.91	-2788.34	6567.02	-2816.94
8136.83	4806.38	3386.66	1954.33	7207.64	-5898.85	4081.78	11498.6	2591.68
4891.09	8440.03	4011.18	4514.81	6772.47	-3887.09	-16.4766	9684.63	-245.137

**S19**	**S20**	**S21**	**S22**	**S23**	**S24**	**S25**	**S26**	**S27**	**S28**	**S29**	**S30**	**S31**	**S32**	**S33**
2017	2018	2017	2018	2017	2016	2017	2017	2015	2017	2018	2017	2015	2015	2015
Meteora	Meteora	Markopoulo, Athens	Macedonia	Drama	Ioannina	Korinthos	Korinthos	Korinthos	Kavala	Kavala	Mantinia, Messinia	Naoussa, Macedonia	Naoussa, Macedonia	Naoussa, Macedonia
Assyrtiko	Malagouzia	Savatiano	Malagouzia	Assyrtiko	Vlahiko	Agiorgitiko	Agiorgitiko	Syrah/Merlot/Cabernet	Merlot-Cabernet Sauvignon-Agiorgitiko	Assyrtiko-Sauvignon Blanc	Moschofilero	Syrah-Xinomavro	Syrah	Xinomavro-Mavroudi - Sefka
14.0	13.0	12.5	12.5	13.5	12.0	13.0	12.0	14.0	14.0	13.0	12.0	12.0	13.0	11.0
Dry white wine PGI	Dry white wine - PGI	Dry white wine- PGI	Dry white wine-PGI	Dry white wine-PGI	Dry red wine-PGI	Dry rosé wine-PGI	Semi sweet red wine-PGI	Dry red wine	Dry red wine- PGI	Dry white wine- PGI	Dry white wine -PDO	Dry red wine-PGI	Dry red wine-PGI	Dry red wine-Table wine
-10169.5	-10180.6	-8285.03	-9425.01	-5188.19	-6445.92	-11402.3	1732.79	-13529.6	-6493.97	-10312.7	-8679.17	-12328.8	-2352.91	-335.301
-10673.1	-12805.6	-13715.9	-11942.5	356.672	-3281.93	-13114.2	2484.83	-15078	-7125.82	-12787.1	-7058.83	-8439.6	-5442.37	-7010.06
-5278.5	-5111.62	-10506.1	-8549.43	-7392.96	-3672.36	-14544.7	-3377.54	-17788.6	2594.32	-18987.6	-6372.17	-7539.47	-11021.2	-5735.24
-15165.3	-11179.9	-16430.3	-16378.2	-7846.17	-8757.93	-14863.6	-7437.17	-22693.1	-8612.96	-12466.6	-5958.64	-22341.8	-5210.71	-10459.9
-4197.45	5622.15	4663.03	-2460.91	543.195	2210.5	-8571.14	3943.04	-7935.19	-6357.93	-13265.8	-3098.74	-6642.79	-669.154	1569.45
-4261.7	2210.84	-5666.93	-4028.94	-3710.78	-10742.9	-10736.1	5492.59	-8399.9	-853.904	-11218.4	-10798.3	-9783.65	2600.02	-1687.53
-10418.3	-927.838	-1523.13	-3286.02	5849.09	-2690.64	-16192.5	-520.516	-9175.39	2835.31	-7777.3	2718.73	-14669.7	-1973.19	2783.31
-7903.63	-8441.32	-1325.22	-3649.37	6299.7	-1294.7	-4008.71	-2150.24	-10718.8	-1309.86	-3981.99	-1764.08	-11808.3	-8791.85	-7044.16
-7984.07	-4310.05	-10615.1	-2822.58	1690.38	-8038.72	-11679.1	-2467.92	-13689.7	-1641.59	-2247.57	-5892.38	-7849.24	-6145.19	-1580.44
-4720.2	-5174.13	-3678.62	-6731.87	670.748	-1720.05	-6561.87	2642.15	-7828.03	-5847.61	-11263.3	-3134.36	-4790.92	-8976.58	1438.81
-7273.06	-2673.5	-4625.34	-4463.5	948.031	-413.02	-9949.56	3379.79	-10085.8	-6107.26	-11645.5	112.042	-8645.73	1510.67	3162.51
-11243.9	-3991.43	-7506.49	-285.305	-4164.1	-851.877	-5844.73	-4425.41	-62.25	-4987.52	-13887	-5684.89	-6908.42	-4060.55	-1904.05
-3335.04	-8779.69	-1275.55	-8749.63	-2610.15	-6960.77	-14054.1	-4953.56	-8521.45	-4494.89	-7706.37	-5651.43	-7209.23	-3188.49	-2191.31
-9858.88	-5842.88	957.957	-2682.64	-385.211	-12382.3	-9187.46	-2645.59	-9026.57	-961.624	-8261.95	-8970.5	-9903.39	-6833.36	-6519.24
-3293.06	-7360.95	-8273.59	-8134.32	-2607.5	-2862.95	-7730.69	-5286.54	-6910.25	-1683.31	-8829.75	-8633.17	-7634.41	-7832.91	2086.31
-3780.12	-4522.22	193.32	-424.916	-1809.9	-8827.52	-11629.3	-881.791	-5546.29	-132.324	-16914.9	-9468.17	-8095.47	-5765.79	-7135.91
-3063.18	-1273.62	-6091.11	-4324.48	390.152	-1154.48	-14504.8	-1942.02	-10910.5	-237.828	-14244.5	-6217.44	-11084.1	-7067.76	-2667.75
-3622.33	-4874.58	-6017.75	-7556.55	-862.799	-3130.64	-11331.1	-4088.61	-4614.14	3525.76	-15798.2	-210.295	-4282.17	-5666.33	3991.12
-4724.73	-8009.04	-689.82	91.334	-2193.34	-10061.3	-15937.8	-10156.9	-14176	-3652.45	-6460.68	-12650.3	-7852.13	-4757.45	4445.34
-4133.24	-2133.93	-3101.08	-2581.5	-7036.58	-3874.73	-11943.5	-6618.3	-16102.8	-1393.13	-8766.92	-3048.75	-10567.7	-8739.29	-1327.15
-1290.13	1234.16	-3326.96	-5083.83	-6301.2	-5760.56	-5940.32	-4405.59	-9297.36	-5060.86	-9443.08	-1198.88	-10667	-9436.71	1544.25
-6461.25	-9277.52	405.174	-2811.14	-6663.86	-2139.55	-8520.16	-4357.03	-9841.63	-1088.62	-8869.23	-471.238	-8529.24	3336.87	511.664
-9002.12	-6784.9	-2101.38	-9726.25	-1943.56	-4456	-5546.66	-191.553	-14786	-112.271	-11738.9	-1394.04	-4973.46	-5747.17	5209.63
-9627.04	-5323.67	-5428.45	-5103.51	4240.03	2265.99	-10035.7	234.168	-13823.6	3644.19	-10111.3	-8079.67	-9324.36	-8344.86	-1901.22

-6787.77	-3937.54	-4262.12	-1754.61	-2470.99	406.564	-10663.8	2534.82	-7229.86	-3038.25	-12282.7	-731.438	-13228	-3691.73	3248.35
-5248.5	-10474.9	-6817.96	-2560.56	-593.889	-4230.11	-4616.45	-227.553	-6098.84	-214.01	-16753.3	3966.84	-11537	-6657.9	-316.43
-5399.96	-4178.05	-4764.67	-184.824	-177.893	-4989.1	-12255	-6016.12	-13230.3	3710.77	-9376.86	-6039.17	-7403.47	-3420.35	-5695.63
-6183.27	-11217.3	-4279.09	193.893	4300.99	-6792.28	-6592.24	2510.64	-6728.94	-2187.98	-9632.63	-4284.08	-7838.58	-6366.16	8528.3
-5803.81	-4364.57	-4263.04	-7120.77	173.422	-5329.33	-12359.5	-2478.72	-14960.5	-2523.47	-7589.55	-6859.3	-8662.84	-3306.27	-870.602
-8633.27	-917.025	-6434.5	-61.8594	2080.26	-4610.67	-10084	5467.79	-15819.7	-3841.78	-14393.1	-6667.75	-10831.9	-5153.89	-515.328
-7141.86	-5701.13	-7993.5	454.687	-8429.67	-8069.64	-12596.7	2339.94	-6406.75	-2945.6	-11594.8	-6803.95	-7446.79	-4304.23	1095.57
-7322.88	-7221.96	-5917.61	-4029.93	-2915.38	-2960.21	-8003.4	2910.41	-4070.63	-1427.96	-10405.2	-8830.31	-1989.82	-3093.33	2418.71
-1794.34	-7024.71	-4955.62	-7233.78	899.145	242.215	-16325.8	2938.04	-8182.51	-7202.85	-1590.65	177.763	-6710.62	-1229.03	-2861.11
-9366.35	-6510.65	-10631.7	-8544.98	1549.05	-1547.33	-10221.4	-137.564	-10007.7	-7312.49	-11023.9	244.836	-8364.77	-6755.74	1982.39
-5295.23	-6922.98	809.051	-7692.77	-1510.71	28.9707	-7719.5	-4275.12	-5538.38	-1662.75	-14810.6	-102.684	-7095	-7755.31	711.131
-11688.7	-2469.78	-6050.9	-10543.6	-3403.31	2002.25	-14254	2058.17	-10518.1	-5942.59	-13655	-5452.74	-8455.04	-8661.76	940.066
-2403.62	-4363.21	-7309.1	-7278.35	-4930.82	-1965.3	-7667.15	1904.06	-14871.7	3499.65	-15349.2	-5867.11	-10444.7	-5689.85	5026.6
-6178.71	1250.41	-4302.14	-4683.52	-3364.33	-2092.32	-9530.03	729.984	-9841.93	-2260.58	-10009.9	-11205.3	-9975.71	-10524.8	-2712.71
-8430.15	2907.83	-1062.86	-6585.1	7109.98	-2150.73	-7521.21	-446.152	-11939.1	1818.45	-19940.4	-5177.63	-6871.51	-4727.39	4395.11
-8994.44	-3993.8	-991.406	-3993.01	-450.277	-5851.34	-9614.46	-3496.2	-14323.5	-1288	-13253.2	-4674.98	-11498.6	-726.938	-422.543
-12546.7	-1145.8	-9570.62	-4623.68	-6601.99	-781.766	-12437.7	-2911.43	-12997.2	-4948.41	-3930.88	-5849.25	-11678.2	-2977.95	-2020.82
-1267.88	-3061.53	-9277.86	-4974.58	-6115.78	-2457.6	-7452.09	-196.242	-4692.11	-756.672	-8480.42	2818.32	-16630.1	-6059.92	-1532.99
-7584.94	-3434.88	-7833.75	-6129.08	-2981.32	-1073.47	-8311.44	-2691.87	-8292.61	-465.639	-4651.58	-7172.91	-9972.34	-9609.02	-4434.43
-9171.5	-6810.51	-9786.85	-9343.61	-6210.54	-5236.01	-8295.82	2553.53	-4688.88	-4696.52	-3330.06	-9706.14	-3852.43	-2832.55	291.137
-9936.2	-2690.48	-5677.3	-7502.85	480.242	-5834.03	-8384.64	715.34	-9696.17	-4538.06	-13372.2	-3453.86	-8062	2041.08	-7248.98
-10417.2	-7977.27	-3878.59	-13895.7	267.984	-3937.3	-7373.86	-2548.03	-6966.02	-7007.53	-8377.58	-1686.11	-8314.52	-1917.96	-10135.5
-9849.02	-3744.37	-11491.5	-3117.29	-3815.96	-1306.57	-10632.5	-4973.31	-6775.74	-7694.29	-7348.15	-2303.01	-9692.38	-3836.08	-892.473
-6788.08	-380.77	-5989.85	-2412.84	-4041.47	-6939.74	-8363.16	-4835.24	-7166.86	-280.333	-4040.42	-4607.84	-6494.38	-6292.05	2384.92
-6230.63	-3696.31	-4262.56	-5850.03	-2678.16	-4099.35	-12098.2	-1797.88	-8962.97	-4594.89	-3953.96	-3311.55	-13337	2308.52	-409.945
-5862.12	-4255.2	-666.691	756.986	1439.22	2344.34	-14468.5	4730.29	-16374.5	-2804.9	-10758.6	456.653	-5994.33	-5812.2	554.461
-3631.82	-4093.82	-5131.35	-3134.54	-4617.46	-1556.03	-10788.2	3310.55	-8248.14	-6598.59	-8654.69	-2842.75	2206.94	-6389.08	-8093.78
-9042.42	828.148	1107.78	-7077.29	53.9805	-1403.42	-4670.01	-2427.29	-12520.5	-3559.98	-5452.66	-8967.39	-3084.04	-3353.63	3479.5

-7631.01	-3619.8	-10460.8	-4029.5	2364.64	2216.6	-9141.36	-4232.53	-16984.6	1472.21	-8108.64	-7519.08	-11961.7	-7758.7	2158.28
-9988.77	-1765.55	-6315.21	-2252.82	-3873.87	-7678.43	-5686.47	-2411.07	-5637.13	7527.95	-12236.4	-2857.57	-5930.2	-4690.44	-725.854
-9328.18	-4172.06	-2829.93	-5395.03	-337.787	-8441.83	-14899.8	-5431.76	-11543.1	-3297.45	-5096.01	-4613.54	-7067.53	-2427.22	2769.9
-9162.01	-7429.35	-7547.98	-2599.04	-6907.36	-6482.36	-11728.5	2421.21	-4259.16	-1042.87	-5067.93	-3667.41	-12288.2	-1825	-963.954
-6603.88	-3773.07	-7842.01	-3957.31	1178.73	2595.8	-11590.9	-608.08	-4225.75	3714.28	-12580	-8938.51	-9660.09	-2856.36	3795.35
-461.971	1139.73	-5659.06	-448.85	-7447.17	-4082.82	-9485.01	-1563.17	-13409.1	1391.93	-9874.38	-9769.5	-1449.45	-5440.98	6463.15
-4435.95	-9733.22	-2278.22	-1732.39	-3008.8	62.6113	-10635.1	2883.79	-6335.8	-3273.04	-10400.5	-1787.25	-8963.5	1303.55	2671.98
-12130.4	-2060.97	-6394.49	-2600.42	-4247.3	-2186.84	-8681.27	-3242.02	-3834.92	1674.4	-5051.84	-10259.7	-7279.17	4513.45	4875.28
-7069.06	-2382.48	-12333.7	-6834.99	-2702.76	-3189.85	-8478.76	78.3516	-2609.14	2436.27	-10531.1	-5457.77	-6187.1	323.193	9794.67
-5358.32	757.924	-5129.56	-3315.32	1567.85	-4352.06	-2110.88	-4733.17	-9604.74	1948.81	-7133.76	-3849.74	-3487.42	-3496.97	508.961
-10609.7	-5964.77	-11085.6	-8386.8	-3957.92	-366.814	-4523.19	-5438.13	-10716.9	-3420.94	-6845.48	1199.67	-3926.82	-4775.13	8167.65
-12939.4	-6399.39	-4809.46	1175.03	-3154.71	-6111.56	-9465.3	-610.162	-11300	1584.51	-8965.09	2708.65	-12258.6	-4858.29	4688.06
-5157.76	-8000.44	-3284.6	-8278.97	-5190.79	-1507.06	-8199.78	4942.08	-4711.14	3148.65	-4052.01	270.65	-10866.2	-3724.92	3587.15
-812.996	-2903	-6517.72	-12908.6	-2681.52	-10557.7	-11235.3	1075.45	-11800.2	-4397.14	-10447.2	-8691.86	-8790.88	-6397.63	-6845.31
-5355.98	-4895.82	-6277.09	-7307.36	-10196.4	-5321.37	-13192.7	-2599.66	-14366.6	-3887.97	-1972.74	-8413.44	-15148.7	-6583.48	-7138.94
-12374	-12551.3	-12492.6	-2118.82	-1049.54	-7538.4	-8483.09	-3817.04	-14279.7	-10732.7	-17810.8	-9458.3	-12973.1	-6492.29	-930.862
-11439.9	-5138.84	-8318.1	-7328.03	1862.78	-8625.09	-10187.6	-1102.25	-7947.23	40.7227	-15138.2	-8692.71	-11949.1	-3470.79	-6118.79
-7003.54	2612.81	-4388.42	-13888.4	1009.32	-6679.13	-12973.9	-1264.4	-10257.1	3086.06	-8115.73	-8910.42	408.941	-11505.7	-5084.82
-6551.06	-12878.6	-5684.25	-7843.82	-6926.08	-4424.58	-9275.07	2919.11	-15313.1	-4435.92	-12012	-5095.7	-6553.33	-11409.4	-61.918
-12014.6	-1884.38	-9391.04	-2529.77	-2451.03	-2301.74	-13770.4	-859.975	-13122.1	-2235.69	-18354.3	-8589.39	-12153.7	-5469.09	-5097.96
-15867.5	-16893.9	-7311.37	-9494.3	212.391	3036.16	-11050.6	-5615.45	-9477.09	-3920.07	-12350.2	-11044.7	-4984.53	-2653.64	-5885.09
-4301.31	-2324.62	1674.79	-3930.97	-903.533	-5367.86	-4273.99	-1043.94	-15124.2	-1352.51	-11548.1	-5673.14	-12111.2	-4385.56	-389.127
-9766.67	-4152.9	-5280.85	-2048.89	-4634.86	-14451.3	-5010.83	2173.76	-5422.77	-4181.37	-17929.3	-3864.93	-12460.6	-5848.89	6464.99
-8268.29	15.9453	-7955.15	-3584.39	-3204.49	-8602.43	-8884.8	-1699.3	-9259.33	148.923	-10810.8	-6997.98	-2376.64	-1533.86	394.953
-5891.99	930.996	-12361.6	-10290.1	-9462.84	-6494.79	-13933.2	-877.604	-12953.4	-2224.76	-7260.8	85.6631	-4451.08	-1773.47	2931.41
-10496.5	-55.8281	-8154.33	-4385.63	-6862.48	-10164.2	-4664.82	-4053.83	-10011.9	-903.868	-8240.61	-7503.86	-9267.49	-3977.96	-2377.12
-15696.6	-2442.59	-9111.15	-4238.8	-4609.49	-10847.6	-1612.58	-1655.75	-9496.32	-4771.12	-10332.9	-8377.01	-17234.5	-912.678	4649.43
-10997.6	-7324.44	-11254	-12401.5	-1420.98	-5987.87	-12107.5	-8216.79	-10403.8	1918.01	-10370.7	-3962.74	-9743.4	-6743.98	-1303.06

-7459.39	-8738.11	-8414.23	-6922.84	-2722.66	-11305.9	-4573.94	850.102	-7986.88	-272.282	-11128.5	-9810.44	-14076.8	-2888.93	-452.309
-4742.36	-13184.3	-10560.5	-4525.91	-5861.28	-8790.05	-8959.36	-7.08984	-10729.9	-3718.95	-8786.51	-4624.9	-5547.79	-3074.35	185.699
-9781.56	-8965.31	-9178.11	-6310.66	-5600.81	-6397.08	-10593.5	3597.97	-19093.7	3388.65	-7595.99	-2659.05	-8321.52	-11227.6	958.207
-6111.29	-472.258	-1428.27	-9526.91	-1829.48	-4531.44	-9371.84	2445.21	-7485.85	-1177.03	-9725	-1159.33	-11341.5	-8361.42	-5019.07
-9178.56	-9354.12	-2293.21	-9480.49	-2964.12	-7088.56	-14502.3	2291.52	-8324.8	-1586.97	-8745	-684.679	-5405.57	-7835.46	-4142.94
-10542.2	-2265.15	-9688.89	-10534.8	-379.275	-1060.46	-10503	746.191	-12210.5	-9747.14	-8546.81	-9213.95	-16526.1	-5955.33	901.596
7045.74	978.955	5614.56	5231.52	6477.65	12784.6	7456.36	13454.5	5407.94	16021	67.041	5839.35	4659.32	1805.09	3806.95
-307.949	1953.42	-2688.7	-1238.42	808.893	-2464.12	-9736.93	8.13477	-12190.4	-592.008	-4950.1	-1470.01	-7920.11	-3307.24	1295.74
-8176.83	-3078.38	5515.97	-9559.41	1528.72	3516.59	-8171.84	864.646	-8874.61	1547.99	-7412.37	-1596.18	-10864.7	-5969.94	-2139.34
-10197.6	-3996.8	-7382.78	-7817.18	-3197.99	-7889.23	-8882.77	-1565.3	-12173.9	1927.28	-8248.17	-3540.54	-11208.3	-11217.2	-9801.36
-7185.9	-6292.36	-335.617	-1749.1	-1918.09	-488.678	-4347.01	517.875	-18494.9	1495.33	-9281.75	-8288.88	-12433.5	-6406.53	-4150.86
-11303.9	-3079.11	-5952.58	326.746	-2469.54	-5508.83	-9273.95	4679.05	-9057.87	3032.51	-12927.5	-8456.05	-4246.03	3409.76	-983.689
-9507.54	-777.061	-891.381	-916.078	1936.26	-2232.68	-4709.77	1808.32	-12744.7	4145.31	-13448	-7733.19	-7426.99	-5176.83	-1881.02
-11709.8	-8674.2	-2468.14	-6823.68	-6992.45	-13148.4	-3795.63	-9889.7	-8180.3	1492.63	-10647.4	-8876.24	-342.078	2479.34	-8772.09
-10890.1	-824.627	-8930.09	-2210.82	3.77148	-3730.63	-5824.54	-2305.96	-14172.2	1829.61	-9495.3	-4915.07	-13467.3	-842.847	-5522.63
-5643.54	-2868.86	-3333.52	-6277.73	-6411.07	-2043.37	-6767.27	5761.61	-11529	-670.518	-10280.1	-5273.36	-9898.8	-5322.39	-4212.85
-5408.27	-10072.9	6448.46	1825.68	2408.5	-4633.75	-5783.18	2008.41	-7228.64	-2727.98	-4365.6	-7325.97	-8419.39	-7410.24	-834.563
-8155.29	-4644.56	-6264.78	-3188.96	-783.686	-5373.4	-7594.02	-7031.86	-4730.17	-354.042	-4521.3	-7841.7	-10134	-11021.3	-4078.56
-13013.6	-3317.06	-5389.78	-4699.7	-5711.33	-1524.95	-13443.1	1781.12	-11068.3	1218.37	-6686.11	-8242.97	-9650.42	-8006.97	-576.564
-13183.2	-937.648	-6116.98	667.055	-5936.47	385.764	-5833.71	-6257.78	-9820.52	-2309.1	-7398.17	-6690.07	-9617.43	-5437.28	2025.13
-7250.77	706.348	-8864.76	-6663.79	-3449.03	-6330.41	-8948.2	-2177.22	-6165.24	7039.79	-8091.46	-7898.29	49.5566	-6935.66	-2095.22
-11804	-4402.69	1643.51	-5893.64	-2632.08	-2580.68	-5974.18	-3619.1	-6586.68	-2022.24	-12068.7	-7262.89	-354.891	-9594.55	5177
-7274.28	-5529.36	-7536.49	-4701.35	501.422	-5930.57	-8972.29	-1328.35	-15619.7	1499.54	-9167.16	-7620.53	-11178.9	-13006.8	-2534.66
-10688.9	-8241.07	-6888.97	-4126.42	-2411.37	-7312.43	-16517.2	3166.46	-8365.47	-3811.79	-6820.65	-4266.67	-8172.16	-11588.6	-963.873
-13437.9	-3009.13	-5289.2	640.057	664.264	-3495.7	-12520.6	-2078.32	-16890.2	-3420.39	-7393.33	-8648.93	-5051.77	-3775.87	1571.8
-6559.46	-6335	-4667.15	-11689.7	-4205.98	3590.11	-8302.23	2351.21	-8067.13	8631.11	-4980.96	5921.06	-3193.57	930.531	2490.79
-5550.11	-6114.52	-4043.87	-6632.51	2260.13	-4526.89	-6572.61	-150.727	-1589.51	1664.76	-7950.54	-4291.84	1415	2317.88	965.426
216.576	719.318	-1995.51	-1457.42	-4548.29	4409.14	-7268.69	-1634.07	-3183.16	6222.9	1217.05	-3644.32	-914.998	417.094	185.51

579.838	-587.102	1617.92	172.432	4567.03	-2543.16	-2048.78	2572.26	-4511.72	3809.19	-9237.53	-490.876	-1612.66	-1309.35	6233.08
-5887.75	-5940.44	-5725.74	-6132.48	262.029	2415.67	-11076.7	4782.64	-8340.7	-3225	-1945.63	-4716.08	-4369.25	-3885.39	5517.21
-2727.45	-4334.93	-8503.88	-2894.91	-3907.8	-4324.41	-11230.7	-11976.9	-4968.48	-3107.86	-11711.1	946.256	-6417.59	-9663.47	435.613
-7545.48	-13561.3	-7586.25	-2219.73	-2501.36	-3452.4	-13873.9	-1397.84	-7870.54	-154.48	-3517.74	-2865.19	-9148.93	-8107.07	1520.06
-2515.56	-7254.64	-2708.29	-1015.24	-9609.91	-8627.86	-4117.96	-3944.51	-9828.11	-6087.43	-12308.2	-3511.17	-7361.15	-3432.3	-2597.57
-5036.83	-1917.21	-9537.08	-4694.17	-7244.51	-3355.97	-8204.45	1988.82	-17494.5	-965.206	-8291.4	-4950.62	-10720.1	-1349.14	3810.12
-2823.72	-1300.44	-457.857	14.9512	1041.6	-2483.92	-2337.67	-3777.1	-9098.7	1224.02	-7378.79	-1064.42	-8816.43	-3406.82	169.721
-3453.26	-2683.09	2226.06	-9579.62	-4212.97	-3859.81	-5049.19	286.795	-8731.29	-1637.78	-8832.76	-7631.98	-6747.24	-3849.33	1011.36
-11613.1	-4507.51	-2116.88	-6328	-607.367	-9087.99	-1452.86	3903.09	-12886.3	871.685	-2101.53	-8526.08	-8619.7	-453.688	754.631
-4147.82	276.738	-2798.5	-6826.58	9136.73	-5173	-6443.68	1527.2	-9170.64	-1600.85	879.479	-3789.54	-4619.3	-3411.7	2323.82
-6275.83	1050.76	5072.51	-5079.14	1778.4	-11914.8	-6743.04	1978.9	-9221.47	-1904.77	-6099.92	-6692.41	-7740.61	-4420.53	4280.67
-4354.19	-5979.23	1190.91	-1793.04	102.623	-1493.44	-929.473	-4780.29	-10390	-2584.48	-7097.63	-3946.44	-6094.9	-12067.5	-3467.43
-8250.84	-8418.57	-7695.37	-8525.79	-5392.5	-15390.2	-10590.9	4295.54	-9175.19	-11493.3	-9263.36	-5197.09	-12555.3	-8230.23	-334.408
-16040.7	-5595.82	-2787.01	-6209.59	-4122.57	-3135.52	-7168.04	-4181.14	-11929.4	1294.22	-9207.83	2166.34	-5424.86	-4830.67	1561.83
-8580.64	-3696.41	-4199.37	-5041.6	-8064.69	-6831.25	-3934.88	641.699	-5167.67	3707.19	-7004.8	-4072.38	-8051.74	-3942.76	2958.99
-9147.87	-2674.8	-1528.93	-1469.81	-1095.14	160.4	-9897.49	-3061.32	-10314	-2315.6	-5000.2	-7668.01	-3994.8	-1460.27	1851.54
-8194.84	-2972.17	-5186.47	-10535.4	-3256.69	-9955.83	-10199.6	2017.2	-6749.52	-8384.45	-13489.1	-5289.06	-7961.55	-4804.16	5734.41
-5577.43	-9028.64	-6465.34	-2727.04	-2920.85	-6113.89	-9574.46	-4799.98	-7020.91	725.079	-7358.03	-5060.87	-6109.49	-9659.66	586.715
-6598.35	-5750.7	-11575.1	-10718.1	-2377.64	-3338.67	-11186.5	-3325.55	-14931.4	-5599.05	-1730.3	-4566.74	-6824.22	-8262.17	2894.54
-13996	-4545.85	-17629.4	4532.7	-4662.12	-2414.67	-7042.46	1126.34	-9210.02	1558.63	-1668.37	-1588.9	-13815.5	-5464.06	-4989.09
-3663.59	-3900.31	-1421.71	-3253.08	-2926.28	-1532.46	-8201.66	4929.98	-1802.58	-3189.96	-7451.82	-9131.18	-2330.21	-12871.3	6543.04
-3658.73	-4975.31	-2476.29	115.268	3168.56	-5703.99	-8797.58	-556.734	-5610.65	544.721	-6346.87	-4945.53	-5067.92	-4612.91	-516.141
-4570.46	-5367.13	-5655.92	10.4023	-5683.79	-4854.4	-11425.5	-2047.23	-7923.88	-996.152	-8952.19	-5729.31	-2856.22	-3757.23	-2514.89
-2146.33	-5587.31	-6534.25	-7424.82	-3376.62	-6211.12	-2683.02	700.436	-5317.26	-420.919	-9815.21	-1909.99	-6953.11	-10014.6	-451.984
5252.93	10938.3	8506.16	13401.5	14049.8	8104.81	19134	4826.85	9150.27	14472.9	25707.2	18478.7	1962.6	11171.2	4099.42
18234.7	14006.8	19708.8	11833.6	19390.5	19161.6	7562.83	24698.2	15736.7	12890.5	8511.59	10933.6	20320.2	16101.3	12771
1257.01	-5825.63	-5194.98	-1861.65	4854.95	2666.47	-5898.12	3452.89	-9815.15	5106.11	-5605.19	-5333.18	-5890.4	1880.35	5044.69
3581.43	2692.75	793.195	1351.07	1126.3	7185.33	-2679.92	3273.73	-3492.58	2793.21	-4540.96	-3633.82	-3735.61	-3273.7	6259.76

-6987.31	-3569.08	-4039.1	-5821.15	-3326.36	-1857.39	-7589.42	1648.56	-9174.53	2239.4	-8761.43	-1196.58	-4033.86	-3528.39	-1807.13
-7364.58	-2929.85	-8497.36	-2470	-2648.02	-4809.11	-5976.58	-10090.9	-15795.4	-4514.65	-11981.2	-11400.7	-6124.34	-13684.5	-5229.73
-8855.84	-7084.36	-4818.88	-11793.8	-3891.09	-6662.18	-7056.9	3415.59	-7367.08	-7434.87	-8866.36	-8913.23	-8978.75	-8784.42	6381.53
-2033.36	-5083.98	-9149.46	-10019.6	182.203	-6519.63	-8639.22	-1986.93	-1807.5	-3701.23	-15204	2324.75	-6133.33	-995.704	828.979
-1110.36	-2945.74	-5926.75	-6442.06	676.156	-13242.3	-4168.11	1134.33	-9358.09	-2560.33	-10713.7	-7603.87	-4691.65	-8308.87	3462.25
-7282.89	-5463.33	-7397.36	-4029.93	-1146.95	-7453.04	-737.111	944.096	-7426.74	-2162.2	-5686.99	-888.605	-9397.2	-10950.2	1297.93
-5823.07	-2850.73	-3793.03	-3273.16	3173.52	730.576	144.98	-34.8359	-9668.04	2042.24	-4383.44	-8913.6	-4819.5	-5625.97	-1862.56
-1877.13	-3596.66	-3158.62	-1406.19	3853.12	1029.99	-3313.88	6052.87	3878.15	3213.5	-4585.49	-1751.05	-4241.36	383.717	-2833.86
-9792.08	-7921.53	-1631.85	-1495.95	3067.86	-5522.25	-6325.18	-4052.17	-12051.8	4294.09	-6388.1	-6029.5	-6595.36	-7041.71	-5638.18
-6288.44	-567.389	-864.953	-7661.05	2140.57	-7505.87	-7171.95	-5536.19	-8139.4	-1517.43	-3838.43	-4690.58	-11014.6	-8427.81	-683.354
-1996.21	-4564.47	-7813.76	-4465.18	-1366.16	-720.309	-9844.41	908.137	-5177.07	378.174	-6041.74	-1945.84	-10393.5	-4011.05	1528.3
-4945.86	-3622.57	-1870.17	-1174.57	-1812.14	5074.86	-6755.7	2605.13	-9839.26	-1553.4	-4395.3	-560.52	-1528.46	-4252.96	2204.89
-4692.57	1487.63	-5878.86	-7825.88	908.191	-598.004	-9144.26	361.898	-5733.94	-460.949	-10916.1	-2449.74	-4107.32	-2425.69	-1589.34
-2144.15	607.512	-10064.7	-9286.45	729.758	-1516.65	-8114.51	497.701	-6709.9	8003.29	-2948.75	-5234.77	-5417.53	-6690.75	4716.12
1271.87	-4412.96	-10378.4	-6448.39	-1354.37	-1594.17	-12859.8	7854.5	-5552.46	-7153.22	-5635.39	-2566.95	-2563.07	-858.022	-1347.78
-4616.56	-2962.26	-505.664	-9794.1	-569.943	916.281	-6639.53	2756.62	-9362.94	-1902.15	-11323.5	-928.421	-6393.49	-7573.92	-3327.06
-7662.2	130.305	-5033.95	-8926.29	204.504	-3315.15	-4342.24	2786.09	-9986.89	661.599	-6345.23	-5408.48	-7517.74	-9768.97	-4980.55
-10016	-3498.46	-5677.94	-1509.4	-3669.84	-1156.37	-8214.04	-137.225	-5785.46	-1595.2	-17458.7	-10279.6	-6021.66	-6451.32	-1168.21
-3229.91	770.014	-6186.77	-6481.06	8235.77	-7327.1	-7329.82	-3126.35	-7265.52	8690.49	-7399.09	-9772.97	-4655.84	-6984.19	180.67
-3237.34	1677.62	-3644.48	-1570.29	4050.37	2496.07	-4260.63	279.521	-267.344	9575.25	-2301.26	-3818.44	5094.68	802.471	-3567.97
-10124.5	-985.73	-4345.02	448.027	2417.59	-1453	-9575.96	-482.473	-3451.52	1084.83	-10639.2	-2723.56	-6355.31	-5453.97	1126.37
-7025.79	-8234.47	-3113.24	1826.71	2489.75	-6461.23	-11387.4	1835.85	-3118.91	-3342.21	-11375.1	-4525.79	-2203.54	-5048.57	-687.949
-4583.1	-1230.06	-4240.94	283.23	-1366.3	-8925.14	-11223.2	958.439	-8515.25	5180.04	-5102.57	-2987.66	-3571.58	-2018.74	-2201.2
-17.2656	-7837.36	-3916.38	-3052.98	-2996.32	1485.54	-5311.69	-256	-5696.14	4516.59	-7831.2	966.547	-4867.34	-6507.39	-1118.76
2490.02	-4294.23	3163.6	1658.84	1816	-3355.03	-8034.3	-238.73	-4051.76	-65.04	-3838.51	4560.96	-4271.21	-15089.6	-2466.31
3344.31	-1117.74	3844.79	8340.49	4482.53	5146.22	673.072	4475.37	-3419.69	6887.42	1684.79	2633.58	-3248.15	-844.489	713.564
-4591.46	-2154.1	-5092.17	7457.85	640.949	-2576.27	-5500.18	7459.93	-1781.77	5430.67	-3263.97	8151.75	1846.48	-1876.84	6950.22
-1157.47	-6221.01	2343.49	7760.19	11454	5520.54	-3035.59	4930.56	6421.74	8067.88	5356.24	13316.8	1519.46	-4330.54	1180.41

9602.13	2053.01	8601.3	6694.46	12253.4	3120.83	7939.01	9079.69	8153.79	16898.7	18402.2	56171.1	1290.71	-4887.57	7296.81
15489.4	10474.6	16265.9	18133.2	20558.4	17133.5	6959.9	17200.6	12155.4	21549.6	13975	131941	17200.1	-1101.26	15670.7
30265.6	24852.2	24716.1	42448.9	49729.3	30915.5	37628.3	27651.2	39695.8	40359.8	41349.1	279425	25785.1	13847.5	20545.7
155541	179897	136377	173328	513978	131484	159457	135408	170082	174700	186714	684015	120603	115176	123960
5.82E+06	4.15E+06	825986	8.83E+06	5.44E+06	5.58E+06	9.34E+06	4.95E+06	8.83E+06	9.90E+06	5.34E+06	6.04E+06	686925	6.98E+06	4.32E+06
5.72E+06	8.13E+06	1.07E+07	1.84E+06	3.59E+06	4.27E+06	1.30E+06	4.87E+06	1.35E+06	1.02E+06	6.45E+06	2.98E+06	9.92E+06	3.00E+06	5.01E+06
148600	252563	175607	102822	179711	132822	107533	126284	90386	121972	111765	133447	168177	141368	131301
25932.4	50805	27361.9	4119.67	21503.9	22986.4	13180	20705.3	3252.84	21175.6	2589.47	27606.1	24247.5	37979.3	32514.8
6216.89	19689.6	6548.05	-1793.24	10816.1	6557.64	4372.18	9719.61	1091.86	3797.81	-9382.71	14045.7	10232.8	19315.8	15459
-3900.9	13644.6	2807.77	-9339.1	287.193	958.465	-1670.64	6076.49	-5437.75	4196.97	-2680.19	5284.57	2518.52	7279.62	5083.32
2710.47	2243.03	-6006.03	-5418.96	-2401.39	978.654	-814.809	5662.91	-9477.19	-110.314	-7629.39	-2890.33	1755.65	8405.95	6659.08
2212.41	5542.48	-4668.62	-10796.5	4783.84	12778.8	-5238.73	1666.63	-3433.94	6085.97	-13425	4159.56	1655.68	8077.05	8362.01
-83.7246	18344.9	-2419.38	5009.19	39108.8	-1105.06	10708.7	8304.2	-6290.45	19901.6	-4993.38	8040.92	-7104.04	5047.38	2392.79
39746.6	45811.4	51775.7	71201.3	63653.6	42077.8	27080.1	37509	28325	53061.9	35799.9	31272.5	37448.5	66903.9	63020.4
-5747.74	11366.9	545.615	-10264.2	-1266.23	-3428.3	674.92	-4382.63	-11627.5	-1406.46	-17314.4	52.2949	-7525.4	1360.16	7426.5
-10339	720.291	-805.145	-7782.93	-369.66	-5745.92	-10650.1	-11198.2	-20653.2	1488.57	-13420.6	-8598.32	-5500.28	-4429.96	1328.51
-1865.36	3464.53	-2733.8	-3201.23	-5825	3697.27	-2708.48	10110.8	-17465.6	-3262.63	-7761.01	266.75	-162.498	-3522.23	10278.5
-7321.88	1111.81	-923.133	-10866.7	41.9668	-3081.97	-9866.21	1783.2	-13186.6	2016.28	-10741.1	-9629.06	-12496.8	76.6973	10040.6
-1156.72	11065.9	-8352.71	-8744.03	-1235.48	12523.9	-6579.76	-666.176	-3206.74	8715.64	-6063.74	-2242.54	5292.66	4163.09	13419.8
-13767.2	2549.54	-3420.39	-7605.08	1414.72	662.27	1043.15	-1282.67	-9712.3	-4538.43	-15035.7	-1871.73	-4431.64	-6785.28	-689.437
-8320.8	-4082.64	-1661.02	-10781.6	-1232.08	-3557.27	-9551.65	-372.391	-8015.15	-7950.24	-16329.5	-7569.01	-13132	-2274.76	1202.47
-9194.38	-3827.77	-3354.82	-4843.8	-9954.43	-1671.63	-5792.91	1525.07	-7134.57	1879.94	-10424.6	-4055.58	-8136.42	-1908.34	2668.2
-5998.19	4860.55	-3751.9	-9612.55	-320.406	-3690.64	-4636.85	-3858.67	-8733.14	-3412.78	-12195.3	-1835.75	-8552.98	-4693.15	398.602
-4792.5	35.6289	-523.549	-7467.58	-3686.57	-4644.42	-6685.18	-112.463	-8608.96	-3900.16	-15470	-3410.84	-9538.79	-4620.14	-1778.29
-3068.55	-2990.27	-1040.68	-3343.15	6336.88	-3359.94	-7042.88	3433.68	-6385.18	2313.07	-8704.43	-614.562	-8488.7	-964.1	-2193.44
-9727.96	-7177.18	-1418.41	-5774.17	3754.63	-3235.5	-9980.91	-6428.56	-5933.53	-4009.91	-10386.5	793.328	-2705.22	-8724.89	734.625
-10447	-3163.52	-4545.07	-8123.21	-3158.54	-5865.26	-9999.63	-3014.16	-6362.38	-3511.31	-5902.04	1126.96	3398.21	903.568	-2023.61
-8564.38	-1362.34	-5215.52	-5316.86	-8559.4	-7301.47	-5350.99	-4252.2	-11122.9	-2771.41	-14745.2	-1707.4	-9433.08	2153.31	2203.81

-2885.28	-3216.89	-622.223	-6250.89	-1702.39	4096.72	-10377	9768.69	-2264.39	-6582.9	-9033.21	-3295.23	3590.23	3325.38	-4280.98
-8839.67	1116.06	-8392.29	-7934.87	1752.73	-4135.76	-9180.18	92.5137	-9570.04	-2062.61	-9783.79	-4043.38	-10866.6	2285.75	-5079.93
5866.73	241.838	-4609.25	-2376.97	3554.23	-1783.53	-2258.64	-4189.86	-13477.2	1421.29	-5936.75	-1505.03	-12594.6	-1236.62	878.209
-6790.96	7373.22	2247.96	-13327	-2838.05	51259.4	-8644.87	20049.8	52176.5	9436.26	-4001.53	-74.5693	-6683.3	1378.65	25632.6
-9436.46	-7777.07	-8661.65	-10252.5	57.6387	44250	-9562.36	46162.8	-5419.08	46449.6	-10781.4	-6612.35	50723.1	17463.3	3851.56
-8423.62	-3622.63	-7218.97	-279.766	-2853.67	-5623.45	-4894	923.684	-12406	893.177	-11919.6	2213.26	-1224.21	-4700.89	-5060.65
-9042.39	229.293	-3758.82	-11422.1	-436.15	-221.02	-10316.4	634.848	-16845.6	-6338.13	-15532.7	-5493.07	-9044.66	-4149.57	-797.795
-5759.51	-3932.17	-8744.07	-1503.44	2069.83	-6599.54	-15377.8	-1701.52	-15415.9	-1526.92	-15378.3	-5733.27	-10399.7	-2292.52	-4549.84
-11912.5	20.8047	-6637.62	-10580.7	-7914.7	-5943.55	-6088.77	1921.71	-8344.92	-1888.71	-17051.3	-9577.12	-7372.04	2782.16	-2028.63
-9925.5	-312.064	-9471.59	-1389.93	2722.96	-4357.24	-13905.5	-2948.51	-14800.8	-4512.88	-13783.1	-7129.12	-14044.5	5881.42	-3291.59
1030.26	4728.97	-2206.77	-10135.8	438.266	-5078.37	-6418.26	3296.87	-11542.8	-4726.54	-13193.8	864.82	-7273.36	-699.063	-3274.45
-9032	-5567.96	-5101.79	-4260.23	-592.771	-5186.31	-5261.75	734.631	-9415.08	-1905.55	-9187.66	-10007	-5943.87	420.354	-973.281
-3593.17	-3520.96	94.5664	-2353.57	7384.99	-6609.49	-12768.3	-9982.82	-12056.4	-3459.24	-5309.28	2651.46	-3338.46	-2963.89	4178.09
4995.49	19313.6	6747.19	21475.7	17989.2	10525.3	14076.1	3391.77	19006	215.062	5953.94	12802.2	-3096.63	2457.67	13685.4
-10293.6	2678.45	-6254.92	-8319.03	-5025.96	30861	-9132.08	16243.5	-11236	13386.8	-6949.54	-6849.13	16621.7	21168.8	701.75
-6329.77	1599.35	-4002.02	-5978.53	-5816.87	270.004	-7056.79	-131.695	-285.023	6710.46	-16389.2	-1255.47	-2511.94	-65.1035	1945.3
-3239.3	879.814	-4451.64	-3637.01	-6932.65	2254.65	-7052.16	5223.3	-3853.89	-4471.74	-8099.59	-1325.13	-6350.5	-4401.93	5422.32
-3071.55	-6711.73	-7617.6	-2735.38	-5650.22	-4351.86	-7164.17	4547.21	-8443.9	5633.15	-9968.4	-2764.95	-5837.23	5089.63	-2452.28
-9715.07	-2600.48	-5170.14	-5730.09	3109.04	-3512.5	-13132.1	202.115	-15283.6	-560.988	-11320	-3056.47	-6599.62	-7888.01	-2373.2
-8880.04	-3005.52	-5747.44	-12971.6	-7411.8	1408.5	-10766	-3140.71	-11061.3	-6241.31	-5271.29	-6749.95	-7262.29	-2315.8	-6421.29
-5660.96	-5616.32	-10948.8	-12471	-1988.48	-133.576	-8905.48	-5181.61	-12729.8	-3161.41	-8537.08	-1814.67	-3894.57	-9673.98	-593.65
-4882.65	4529.6	-888.002	-8663.62	299.289	-1609.01	-7081.45	1855.22	-14734.8	-2536.8	-6838.92	-2754.53	-6313.8	-6596.91	194.295
-4187.5	-6183.8	-6254.84	-8795.49	2700.95	-3885.35	-7345.16	-473.898	-7978.14	-2560.1	-5537.12	1257.59	-8956.68	-9254.31	217.789
-4085.04	-386.48	-3228.17	-8337.49	-4021.36	3470.96	-7252.54	-1127.25	-5384.9	1179.78	-12035.7	-2482.66	-5857.74	2727.82	1080.81
-13279.6	-3841.26	-11130.1	-3655.5	-6121.86	-4205.23	-11168.4	9023.64	-11661.4	-3742.03	-10159.2	-5994.24	-6515.23	349.244	-4696.08
-6448.51	-2218.95	-5883.43	-7689.44	3090.26	-3984.97	-5111.9	2128.47	-8027.58	-1892.88	-8703.42	121.195	-2900.46	-5129.14	-2518.11
17992.2	16347.3	20387.9	8829.87	39.6289	31363.2	11766.8	28120.5	24736.4	30268.4	17101.1	8207.89	32500	29906.2	18194.4
-5423.22	11011.3	-1678.31	-4092.27	-3867.34	829.031	-5964.14	2193.21	-12421.4	-4081.66	-4182.11	-8400.82	46.5586	3477.09	4089.23

-5372.34	-4591.53	-1278.92	-2553.84	711.322	3846.34	-3473.99	-766.98	-12637.1	-221.949	-12162.4	-3275.84	447.486	-2113.32	-1833.66
-6978.16	-3570.87	-8558.45	-14507.5	-2342.62	-3013.8	-7407.8	-2348.91	-8264.83	-720.962	-7752.06	-5972.15	-4613.93	-1099.77	-2086.81
-3246.3	-2737.62	-3562.42	-6042.35	59.3457	-7553.54	-7000.93	4053.92	-4917.46	-4626.76	-7402.2	-2942.5	-1532.94	-6081.65	24.1348
-7309.05	-3398.56	-4636.71	-6936.39	-606.473	-1806.83	-14529.6	-5423.11	-9859.34	-1496.72	-11513.4	-1909.36	138.682	-3756.16	2329.9
-10248.1	-1300.47	-5753.96	-7734.21	-5371.11	-274.961	-11672.4	-2473.35	-8990.28	-1563.81	-6834.94	-5159.28	-2851.18	-1524.16	-3206.57
-4750.89	4347.65	-5155.87	-1924.35	2301.07	-751.314	-9125.95	-6996.18	-5558.04	-5764.3	-7711.57	-9016.79	-9045.22	-3615.56	3362.95
-1865.07	-4998.85	-4762.65	-4589.83	-2578.58	-1416.34	-10338.3	-7614.5	-13125.9	-3067.68	-10119.3	-5505.76	-1883.22	649.926	8187.9
-2743.84	-556.059	2857.45	-6884.07	-1114.84	-10401.3	-8034.16	-1732.15	-9793.01	-2234.77	-6073.37	-1394.31	-7613.6	-4643.5	-7049.59
-9123.27	336.605	-6111.58	-6559.92	-1354.98	1408.5	-6633.25	-5719.68	-14474.7	-2072.76	4333.17	-7877.51	-3654.37	7401.65	6873.28
-5066.57	2449.55	-2155.51	-678.623	-2708.04	354.203	-386.422	3225.19	-6420.83	3167.37	-10421.6	-3132.85	-5523.5	501.814	11633.4
3325.58	-1530.13	390.789	-2155.71	2362.71	-1946.3	1344.18	6686.99	-5087.46	457.385	-4628.82	1043.54	-76.5195	7599.23	-2976.09
-10489.5	-7252.88	1434	-6093.78	2041.49	-963.227	-8748.83	-4923.39	-7451.81	-3805.29	-5972.51	-3257.1	-1206.47	-4514.58	1643.34
-7223.96	-5492.94	-5245.88	-9027.44	-1669.53	571.941	-12137.2	-1662.44	-10599	-5766.52	-12096.1	1742.75	-14060.4	-1541.53	-910.126
-6813.4	-2984.41	-7080.68	-12589.2	990.354	-2403.63	-9964.38	-694.182	-11164.7	1001.6	-9353.7	-1386.14	-6339.2	-6792.71	1408.94
-7894.54	-4468.12	-2623.09	-9168.33	-7471	325.859	-9889.43	4449.23	-10444.2	-3121.12	-7968.5	-2344.74	-5603.03	-99.8359	-6819.3
-8543.78	-802.574	-6308.09	-4779.12	1575.15	1696.29	-12571.1	874.521	-9922.71	-1417.76	-16905.5	-84.7822	-7131.66	-12542.5	1942.46
-4071.93	3560.39	220.105	-6433.39	1769.46	-2525.44	-8891.56	1005.34	-6310.6	-8208.55	-14955.6	626.58	-6124.52	-39.0801	-2792.95
16822.4	19563.7	35279.2	12581	14708.3	13871.5	16058.7	34206.4	52519.2	70784.9	7510.85	35101.4	55885.5	23121.4	16185
-5454.34	-3392.09	3557.7	2130.98	1391.14	623.996	-4951.02	7570.57	-10611.9	10066	-7319.11	4243.3	-2097.57	1274	3912.79
-3429.75	682.717	-7735.87	-6830.27	2065.3	-4397.43	-5437.09	4839.83	-5167.44	2908.1	-8613.13	-2363.21	-6511.94	1053.45	1465.07
5189.69	7126.52	994.795	-4424.6	6680.9	15917.7	-128.322	6322.01	2955.58	5937.07	3085.4	3731.17	9805.63	14685.7	6174.68
-9069.58	-8325.87	-1111.66	-3600.88	198.344	-3496.28	-11050	-4462.37	-14362.8	2694.58	-2936.56	-4731.34	-6535.78	-5165.41	-439.225
-4386.22	355.012	-8503.09	-7184.1	-997.182	-7659.6	-13209.6	-7261.96	-6287.02	1599.62	-2969.99	-7582.52	-5134.68	-1627.42	2679.55
-8583.48	-2405.38	-3603.27	-13677.1	-5249.62	-2207.86	-9394.58	-7193	-14397.6	-1752.15	-545.934	-4512.45	-6592.17	-3333.6	-741.668
1281.55	-4886.95	-95.6289	-12308.5	-6159.77	3556.83	-3944.4	3383.65	-8872.73	7811.56	521.459	-6131.81	-7360.12	-662.505	5981.73
-6547.59	3757.52	4089.58	-7555.36	6787.25	-4572.48	-5852.58	4198.8	-10779.8	-151.083	-4067.82	-6535.88	-8592.82	-9771.09	3627.44
7356.93	-1006.63	-3056.29	-1006.97	4356.64	-2528.46	-3527.03	7908.29	-7432.09	8363.71	-8533.51	104.782	-5592.78	-169.746	-2046.94
-4083.3	-5985.3	-3542.67	-210.471	-1730.77	1175.84	-7834.72	-201.85	-8320.18	1944.2	-6306.78	-452.887	-2833.78	-624.616	-3261.18

-13658.9	-4142.57	-4626.81	-2386.24	-8756.85	3972.25	-3795.84	1860.75	-8165.78	841.883	-4172.53	1899.17	-10884.2	-557.888	2231.01
-9913.17	-443.098	1899.12	-4047.21	-662.473	4656.63	-2318.84	-6233.72	-7682.11	3292.49	-6022.38	-2882.55	-4644.77	-7462.11	3235.72
-11497.2	680.621	-2236.86	-5912.63	-553.805	2882.01	-11422.7	-383.791	-8684.16	1553.34	-13822	-2379.58	-7372.11	-8484.5	5777.56
5165.53	353.402	7571.79	-2691.87	719.371	9333.8	-3107.57	13197.8	-1891.54	14744.9	2980.33	1373.41	6954.59	13428.6	17752.4
-6686.54	700.334	-9391.51	-3872.02	-6703.52	-2260.13	-3962.04	-2892.98	-6771.72	4389.33	-14562	-5735.39	3127.91	1130.2	5724.06
-10374.3	-2552.12	-3344.25	-11346.5	-7272.13	1617.11	-5101.5	-4342.65	-11037.7	-432.256	-13144.5	-346.989	-5797.05	2856.63	198.756
-2812.83	-4800.99	-7467.07	-12434.3	-1980.57	-6666.68	-5084.69	-1645.37	-5628.56	985.571	-8627.27	-6961.29	-6520.64	758.115	4869.55
-7657.24	-5134.86	-848.979	-9307.32	-1498.04	-5131.76	-5877.72	-2974.36	-7217.86	4995.03	-7864.2	-8457.04	-6481.72	-7364.38	-3914.57
-9556.18	-792.309	-945.309	-7384.96	3712.49	-1005.22	-5132.05	-500.543	-5593.13	23.957	-1368.13	-582.366	-10635	-2365.82	-3775.36
-6729.24	-7409.82	-4544.31	-7702.95	-1944.31	-4413.24	-12760.9	235.25	-8647.45	1236.28	-13069.6	-3145.7	-2992.59	-5805.45	3375.33
-13898.7	-8045.85	-8895.4	-12490.3	-4397.05	-1432.62	-10553.4	2628.91	-10631.7	-7512.01	-15229.4	-4769.56	-7093.63	-2638.07	3721.98
-3239.68	-5789.79	-3042.38	-2332.14	-4205.41	-4836.34	-9626.8	2874.21	-12059.6	-3847.43	-6024.13	-2058.28	-5406.68	-6255.94	-4153.53
-3173.5	-3016.91	-6954.06	479.115	-5619.53	740.977	-10619.2	5204.93	-1454.24	7772.86	-8955.82	-3539.76	2868.09	1172.44	1771.76
-1649.65	-5779.52	-4500.52	-5265.47	-362.361	1505.59	-11143.9	578.594	-7578.04	3291.19	-5972.3	-4218.63	-3213.08	-1797.49	5606.29
2477.56	-2343.21	-5697.61	-12634.8	-2155.94	-2573.61	-7535.82	348.816	-12422.1	2967.88	-2627.94	-2275.35	-4383.18	528.359	-2910.04
-10153.3	-5567.14	-312.41	-4717.18	-7261.2	-2622.23	-6432.38	3342.59	-11294.6	-2159.4	-8661.02	-4521.42	-8260.69	-367.794	-3741.6
-10108.7	-366.768	4153.21	-8581.54	-3849.74	-344.102	-10245.6	1288.7	-9422.24	3620.78	-3752.85	-2534.89	-7356.19	-1604.13	5105.79
-6305.79	-7686.28	-4300.43	-10659.8	-7050.95	-588.129	-5821.99	100.889	-10141.7	2740.22	-7708.5	518.744	-2836.26	-5559.11	190.402
-4594.74	3149.55	-2991.4	-8144.14	-3354.79	-4053.75	-14254	-2134.43	-15663.8	-960.959	-6031.35	-2469.13	-1588.45	-3393.44	-6238.39
-11519.5	-3403.11	-7470.49	-14447	-7256.79	-4967.99	-10817.7	-4057.02	-5732.26	8144.83	-8005.07	-3443.6	-2434.11	-4356.12	1897.36
-9196.63	-5780.86	924.893	-1704.1	-7890.12	-8209.94	-12477.4	-1304.13	-8291.75	5782.05	-5455.37	-3228.78	-8812.46	-4178.28	-721.527
-14690.1	-4186.37	-3041.11	-5927.2	-443.061	-3422.87	-10579.6	2373.67	-7278.42	-4090.3	-1981.79	-1552.42	-10217.2	6239.5	-1204.62
-1932.94	-11962.6	-6913.45	-6511.56	-4260.29	-5799.17	-13438.8	271.51	-11298	-5258.24	-9172.96	-5023.17	-5209.66	-5105.07	-2681.51
-9372.22	-8971.41	-8076.92	-5987.89	-4564.38	-1277.84	-15522.8	-5166.47	-7429.81	-4502.94	-9540.59	-6296.35	-8077.7	-9102.91	-5672.49
-5163.09	1393.84	499.834	-7865.44	911.789	-7067.01	-6841.07	-987.865	-7072.36	1587.5	-10226.9	538.448	-3082.6	-198.091	-1508.61
2030.14	-5751.64	683.791	-2689.46	5570.14	2426.9	843.045	970.801	-9955.03	5582.23	-5926.97	1541.73	-1977.86	-214.728	3827.88
-4523.66	-4035.87	-5327.35	-5219.12	2723.13	-3427.83	-1288.01	-207.941	-7332.18	-1400.74	-6862.36	-82.708	-5820.01	-2934.43	437.398
-5076.22	-4837.26	499.953	-4749.62	-2101.36	2925.11	-5791.58	1758.34	-4800.99	307.013	-11523.3	-1570.34	-2610.1	2760.52	3177.81

-4552.1	-2245.13	-4642.31	-6011.56	-392.102	2422.08	-4965.11	4440.81	-8373.65	5564.84	-7230.95	-2246.26	-11311.5	-4644.88	3308.58
-2305.91	4806.27	-5747.16	-5797.58	-2403.97	1135.71	-4423.42	556.178	-1834.25	6885.73	2175.37	-1440.93	-7228.37	6015.17	1981.42
137.162	-1758.23	-136.043	-7984.81	-1405.92	5925.97	-2395.84	7929.8	-7337.89	-1188.44	-9105.89	2630.8	118.908	6160.57	3018.93
-6860.51	-2662.14	-279.426	-479.871	-1066.42	2193.75	-4101.54	755.947	-3727.43	4821.1	-11330.5	-487.787	-4897.48	3699.57	5752.45
-3262.87	28.459	-873.061	-1334.72	749.801	-1418.36	-4859.41	1720.46	-7146.31	-1699.56	-10153.6	-1806.55	2362.38	-5853.58	1984.52
-8802.43	-7844.71	405.492	-3536.13	-280.605	-5502.09	-5138.02	-4032.72	-8885.82	-813.8	-2704.31	-3004.68	-9217.58	-3582.31	2840.87
976.814	-7015.87	257.818	-5276.64	1275.95	146.674	-4188.65	-1482.78	-3919.72	-278.818	-3466.6	2142.96	-9306.48	-5458.06	912.139
-9156.24	2330.72	-9661.31	3802.96	-1676.27	-3478.78	-5937.56	-3368.67	-8624.26	317.168	-3331.53	282.149	2583.54	-4913.67	2111.94
-6091.03	-3419.82	-5013.37	-1677.83	-4993.37	535.221	-10100.8	4072.91	-7544.66	-1181.46	-7839.26	-1498.69	-5176.59	4833.06	4603.31
-2641.61	5346.69	-5098.37	503.115	-4478.24	489.045	-4182.27	-517.811	-10940.4	-4725.8	-4919.76	-6695.07	-3058.68	-3279.96	4198.4
-2093.05	-7851.18	1447.67	-3420.06	-1552.18	835.742	-8036.15	-5173.46	-7092.78	2112.14	-7790.76	-5326.87	-7746.71	-1043.97	-592.002
-8401.37	586.463	-696.139	-250.889	-2804.88	-6754.9	-2956.57	-891.848	-7912.07	7981.94	-4655.91	-1763.4	-9512.65	1520.27	4213.78
886.537	4835.71	2299.42	-6065.46	8598.85	-1033.98	-1451.39	3516.95	19.3184	1976.62	794.535	2178.81	-922.516	387.42	5290.28
85121.7	30200.3	39679.5	59675.5	40402.4	22361	34052.1	37651.2	57618.3	72086	24137.5	37533.5	63501	26791.2	20417.1
8853.59	35474.7	51534.9	6781.39	2754.78	9562.11	22956.4	11667.9	15363.9	22611.4	45405.5	38634.2	9550.29	6091.52	19249.2
820.609	35466.8	8135.54	-3196.81	285.689	47540.4	3534.1	9614.43	60254.8	12844.4	-459.826	4041.83	9738.5	18329.9	70227.9
649.107	1516.15	-2157.3	-8452.14	2914.2	32860.9	-4955.03	50073.3	-11552.6	94190.2	-5092.28	1319.79	77206.7	74394.2	3120.85
-7313.14	1702.71	-1674.26	-3411.29	-631.982	-7298.75	-8113.15	1.52344	-6042.44	2528.99	-9069.67	-1061.91	3630.67	1083.1	-1996.54
-4975.21	-3332.86	-878.068	-8033.47	-4627.79	2907.67	-7217.11	5569.67	-9462.78	5876.92	-3836.63	-2059.13	-3844.13	640.174	2986.97
-4450.65	-7561.57	-3374.29	-5506.06	-8406.15	-3584.94	-1141.64	-150.064	-9909.35	3674.95	-7417.81	-1236.51	-8468.19	2156	-3020.67
-1662.67	-2837.85	-2312.56	-1360.63	-12270.6	-3587.55	5116.38	905.252	-7099.25	512.039	-5753.48	-4461.15	-3068.34	1164.72	-1998.76
-3613.68	-411.447	3930.9	-4564.54	468.105	-4023.36	-4810.41	-1777.45	-12018.9	173.443	-6462.02	4769.76	-5718.41	-6637.61	-1453.56
-4608.2	1801.15	1465.71	-4114.92	1562.75	5557.51	1241.62	3196.03	-8004.56	4155.1	1486.4	1104.87	-2742.21	1741.26	-124.586
1436.97	6377.25	-2815.95	-3894.67	1229.24	-1542.31	85.627	-3668.5	-8611.56	793.84	-4168.42	-2801.36	-3894.01	4847.64	2107.54
3997.7	-3222.6	-619.123	-7391.19	1016.37	129.312	-7735.61	1963.17	-6374.09	-3967.44	-3344.46	1619	919.211	-4329.42	-726.881
-7521.48	-5386.36	2876	-6747.1	-3112.47	-4054.09	-9018.36	8192.93	-4789.64	3812.14	0.521484	-5220.63	-1300.41	-2598.62	-16.7422
-4132.37	-5033.53	229.443	-1889.73	-1534.17	-5944.24	-8090.48	1217.75	-7627.29	3496.52	-3996.79	2848.57	-4581.34	-2812.11	6718.07
142.469	-948.219	-5774.46	-7756.29	-2903.41	-6055.21	-5975.5	-509.777	-8942.09	509.602	-15482.5	-6674.56	-11207	4889.36	-4956.43

-2483.75	-3108.82	-5702.26	-3564.12	2686.15	1725.33	-2407.9	-1962.73	-9130.93	1059.24	-7545.86	-1698.88	-11179.6	-3479.65	7824.65
3372.75	-6709.91	-927.227	-2257.57	-1437.81	4857.51	-8204.38	-15.543	-10005.3	1453.71	-6158.31	-5481.19	-7572.13	1192.72	7700.8
-6018.82	1920.77	7063.72	-3588.55	-609.541	-761.049	-10945.5	5404.8	-11097.6	-2654.24	-9990.55	-892.122	-6209.09	4817.6	4534.58
-2888.53	-3225.58	-234.916	2496.97	-4682.2	1609.93	-4609.4	-2311.4	-12191.4	3761.92	-10402.4	-1840.08	-7806.37	-3884.27	3381.82
-8170.24	1571.34	-9470.28	-2802.21	995.496	-3057.09	-10577.9	1582.03	-11428.2	6942	-5244.25	-2735.39	-1549.38	-5598.33	3592.32
-6318.28	-7279.67	-2749.78	-12.6074	4963.86	7791.48	-10325.7	-4598.99	-8629.54	2590.7	-10524.2	-1103.49	-12979.6	-5915.33	4491.95
-7089.82	233.545	-168.035	-4659.88	-3011.14	-2386.52	-8021.12	-872.275	-8421.28	-2162.72	-8494.43	118.558	-12270.3	-4420.68	5100.3
-12824.4	-328.951	-9024.91	2113.97	-2746.48	-6596.98	-4245.57	1079.56	-9581.32	-151.563	-8577.88	-218.275	-3094.89	-3835.3	1740.12
-10805.2	2014.96	-710.842	-7610.71	176.086	-680.232	-10030.5	-7331.65	-7780.4	5067.19	-5765.32	-10369.8	-5468.88	-3795.98	-77.0039
-9620.23	-4152.81	-4447.95	-8382.52	-4110.03	-1515.71	-2430.43	7448.57	-2855.5	-1478.51	-10075.5	-2856.15	-4186.6	4378.51	6421.16
-3485.63	-2837.94	-2077.3	-2952.23	2375.45	-2514.09	-7002.88	-3519.83	-4578.71	-135.13	1877.39	-3147.46	-7009.39	-7102.42	3007.62
-6849.52	-638.068	-4982.96	-8265.39	-1430.96	2531.02	-7405.77	-887.641	-12589.5	6576.27	-7248.33	-1636.13	-3949.7	-6520.14	-891.12
-3412.15	1768.96	-2313.83	-2306.46	-6559.52	-1332.95	-13523.4	-3144.7	-8369.76	-488.756	-8693.49	-3427.66	-2281.74	3280.58	849.254
-2743.28	-125.627	-3896.24	-7214.59	-9926.67	425.951	-10204.3	-4546.28	-10286.1	-6053.49	-7295.72	-3245.33	-2617.58	-354.964	1798.93
-8219.31	-5698.6	-1464.95	-8088.29	-11351.2	1060.91	-11229.7	2166.97	-1916.31	1634.51	-11318.9	-6751.87	-6819.11	-551.316	-988.826
-5660.59	2005.84	-463.779	-6015.78	-2137.23	-2284.1	-12249.4	-5454.36	-6520.54	-401.273	-6014.19	-6060.46	-7146.91	-1090.46	2987.7
-1979.51	-2683.28	-6927.52	145.465	-5685.21	-4294.59	-9713.81	1191.26	-11346	8724.61	-3321.65	-8419.19	-5796.79	87.9883	3087.35
-1425.33	-5992.35	-1499.44	2646.08	-2734.26	-2313.25	-13924.7	-3975.16	-8636.66	487.224	-4177.52	959.986	-1732.74	-1654.93	4777.85
-8349.83	-5723.33	-4846.93	-2554.13	-3603.38	734.016	-9444.38	-4696.77	-3751.8	1927.02	-11819.6	3656.86	-8447.86	10882.5	6494.38
1691.32	-1650.89	-5828.43	-1737.83	-3828.24	416.937	-1695.05	6401.68	-4878.7	730.766	-7663.62	-3227.05	-6879.73	-4260.24	185.617
-9271.14	-4617.41	-4365.13	-3435.19	-2640.94	-3475.95	-937.879	-8536.86	-16370.8	-3501.93	-7804.01	-6165.45	-10211.5	-2711.14	-2276.33
226.314	-3890.89	-3849.94	-3747.51	-11176.5	-2446.37	-5800.2	514.689	-10040.8	-3533.87	-4230.81	-5312.37	-10090.5	-3961.04	-6405.61
-4050.74	-5907	-2323.77	-13213.7	-10974.7	-1542.47	-7442.22	-511.682	-14166.3	-7233.42	-5292.2	-723.706	-15274.5	-2882.25	-2512.68
-3413.55	944.998	86.2207	-292.162	-7905.12	-2802.74	5070.49	-5870.41	-7996.18	3589.76	-3225	-1654.78	-6174.17	1262.77	3938.45
-4343.3	2940.91	624.301	-2550.61	-477.068	98.0566	-2390.57	-3042.33	-8712.66	3027.78	-2855.09	2504.1	-9879.51	1605.56	-10815.8
-3957.93	1899.23	-11112.8	-5428.45	-2441.04	3920.78	-14140.7	1212.52	-6638.89	-79.5527	-3555.27	3671.24	-7345.6	-49.0137	4900.24
-10697	-2417.47	2188.5	-3182.07	3663.71	-4247.56	-1781.44	-6886.86	-5416.87	-813.246	-7604.7	-2892.63	-5565.23	4930.8	-480.129
-1762.03	-2066.17	1127.61	-5622.82	-1637.12	2053.94	-9040.23	-6559.94	-2997.43	259.153	-6466.41	-2487.21	-329.822	2697.15	1348.41

-3673.83	4219.51	-4736.1	-10055.3	2734.27	2159.8	-6564.73	1970.57	-5627.37	8628.96	-6952.2	740.909	2515.52	99.9844	2700.29
-6412.43	1015.01	2053.47	-2443.5	3345.65	-3973.44	-1924.17	4547.59	-3988	9298.48	687.316	-1922.42	-3407.66	1935.21	-494.025
-19452.3	-7253.42	-8659.19	-12953.3	-9890.56	-12607.6	-15348	-5881	-11145.9	-11779	-13199.3	-15530	-6590.43	-4675.91	-10371
-6184.24	-6638.07	-3683.87	-6178.9	-5766.82	-5064.01	-7810.59	-4435.44	-10805.8	-376.014	-146.559	1547.12	-3047.65	-11946.4	-6468.14
-4271.69	-6742.01	-1006.55	-13170.2	-4191.14	2641.79	-5531.46	-493.971	-10384.2	-4400.17	-11370.1	1559.3	-3225.71	-4046.02	4344.5
-3439.03	-2397.82	-4230.85	-7548.64	-7460.53	-4326.9	-2009.09	192.439	-12775.1	4733.06	2499.61	2306.75	-5852.93	-5090.63	708.4
-1125.81	-4375.48	-131.553	-7858.29	-5484.94	-3019.57	-7329.12	60.8223	-8540.51	-3172.16	-7268.05	625.903	-9045.25	1461.96	1743.83
-6516.66	-4117.85	1478.83	-5917.31	-8389.83	-4157.56	-11692.8	-6340.6	-7068.9	-1576.04	-7732.91	-6700.08	-6294.03	5426.09	1911.29
-5065.61	-6300.59	-5643	-2821.95	-5117.08	-3965.81	-9870.64	672.609	-10466.8	-1498.67	-5758.6	-1645.89	-4898	-825.219	567.709
-6290.94	3028.66	-1801.03	-424.752	726.445	-188.322	-3672.66	-1371.32	-15128.9	58.0049	-5995.16	-236.74	-4743.52	3075.62	-209.234
-3063.21	610.396	3584.53	-5880.62	-1126.99	-824.309	-1294.56	1251.66	-6564.21	-1028.91	-4920.42	-10742.5	-4915.63	-44.1641	2230.55
3689.28	-7878.46	-6867.27	-6296.65	-2639.68	-1188.54	-1133.06	795.932	-8145.99	-36.7529	-1822.62	-7285.91	-10900.4	5367.36	973.779
-2912.97	-614.469	-3454.45	-8990.71	-313.842	-3296.29	-4057.18	-272.186	-1777.94	-1029.35	-2510.33	-6562.69	-7193.27	-9492.8	3262.81
-6197.91	1308.7	-7075.31	-4479.79	7527.83	80.1953	-10474.9	-1368.39	-2790.85	2348.71	5442.27	-6699.81	-4986.98	-782.271	-2847.75
-5817.35	-8350.93	-10603.8	-4616.76	-7051.36	-4686	-7480.65	-2027.44	-13897	-792.455	-9774.69	-6788.89	-7788	-1262.36	-1580.77
-4000.57	-1061.76	-2349.72	-10284.6	10.4805	-5328.83	-8217.63	2121.26	-3201.2	-5352.44	-2218.78	2785.24	-9593.77	-6401.83	2683.63
-9385.8	-21.9922	343.775	-3225.36	-629.869	-3927.34	-4115.73	1649.46	-8918.64	9683.97	-3979.58	-3625.72	-5699.78	-4130.3	-780.52
-5721.02	-2836.33	-611.334	-6901.42	-512.844	-5103	-4576.66	1143.21	-3883.71	2920.36	-1581.5	-2263.62	-2593.51	-344.169	4041.35
-4169.3	-6181.13	-2493.46	-3761.12	-1300.95	-7296.15	-8425.16	-2298.5	-5780.75	3703.94	-8141.68	1569.64	-12097.5	-253.815	-490.645
1812.97	-336.545	287.186	431.018	-6939.28	-2962.88	-6576.55	-3081.49	-11454.2	5533.85	-6382.4	-3150.53	-2817.52	-604.407	-3964.12
-9809.87	166.389	-6683.28	-46.2344	-641.842	-649.311	-14618.8	3727.44	-6814.56	-3131.94	-4300.17	-3017.26	-3195.05	-5364.24	-1650.03
3198.16	-731.561	-5116.93	-4080.5	-4133.39	-8630.33	-14714.3	-1260.63	-7488.16	655.838	-7244.71	-3039.99	-2808.15	-5671.76	948.447
-8264.48	486.592	1569.32	-10138.7	2331.08	-3085.74	-6.05664	451.465	-9337.14	-2696	-7970.91	-7196.99	-119.73	-391.861	3452.38
-2830.47	1691.9	225.271	-5355.83	4894.72	7672.14	-7711.02	-5643.41	-5352.65	-804.789	-7297.98	-11728.5	-6486.76	1048.03	-1396.87
-4632.67	-3226.73	-3300.77	3069.96	-3918.51	2333.17	-9494.67	2898.68	-9310.52	1135.76	-6049.72	-2419.3	-7263.7	7525.75	-2350.46
-4741.99	4688.45	51.6074	-6594.83	-1854.14	2270.96	-6116.01	-4474.09	-10294.9	1822.26	-2934.34	-95.7168	-2009.89	5986.2	-962.575
-4622.74	-2471.45	-3138.99	-6702.1	-5086.18	-14.8477	-4736.24	2913.36	-4900.21	2260.37	-9020.51	-3710.09	-4519.95	-538.018	-166.465
574.781	4808.31	-3718.04	-4443.72	733.764	-5553.48	-5589.73	-2403.68	-7360.05	1546.7	-8315.52	-7637.19	-602.156	-832.911	2284.46

-6271.89	1305.39	-7015.54	1293.64	1718.56	-4134.21	-6733.87	-2752.89	-10646	2708.65	-5054.88	-458.095	-3546.7	-4789.11	-1421.88	
-2454.65	-3637.57	-5770.33	-6226.6	-3757.72	2165.29	-5645.88	-4590.36	-8068.68	5728.72	-7993.74	-3206.8	-4125.88	-888.558	2812.89	
5155.07	-7817.04	2150.07	-8763.64	4580.53	-517.131	220.936	-2437.09	-13456.6	499.446	-5342.88	1709.9	-8313.78	-5334.96	1284.44	
2110.24	7559.23	-5312.53	-8925.41	-395.598	-860.002	-3546.18	-1051.11	-5942.96	11585.7	-4706.14	5247.01	4780.84	4709.39	7803.92	
-4087.98	-2084.66	-1398.66	-2936.87	-3591.44	3636.45	-1809.26	711.434	-3417.21	667.766	-5871.85	935.861	-2082.2	-335.381	6733.53	
-3739.95	2958.71	-4215.39	-5859.92	-4064.4	-4336.89	-8945.16	2735.77	-5352.88	-2729.79	-692.203	-2345.28	-4438.31	-8200.5	-980.58	
-3751.41	-4446.46	-7626.54	-7754.78	-6869.21	7869.05	-12806.3	-273.789	-10162.9	3347.62	-11843.7	-3543.57	-4192.56	2345.34	6225.19	
-977.85	-5776.18	-6227.42	-6943.45	-7517.81	-4871.95	-14727.3	-689.955	-10224	-2277.94	-7565.05	1991.23	-9165.22	-3839.71	2097.91	
-7534.84	-4757.31	-2302.11	-6486.96	-5365.38	-468.32	-2194.55	-2160.26	-8555.03	-4591.31	-7706.98	-5466.06	-5008.16	-1167.47	6318.73	
-254.852	795.467	220.264	-7921.32	248.836	-8510.05	-4169.05	3191.28	-7577.68	557.092	-6665.21	-1967.46	-5489.99	-5303.04	2251.9	
-4020.15	6298.61	554.199	-10537.9	-1373.4	4735.98	-8806.23	-3126.87	-12767	5348.51	-10564.9	2.96582	29.0801	-3114.85	2571.08	
-3642.8	-3228.07	-8279.79	-3727.93	-2746.05	2356.91	-6795.28	1614	-8405.21	4988.75	-5710.33	924.5	-9974.04	-1342.41	-2556.26	
2161.47	1811.48	-5133.7	-871.41	-6775.42	2624.51	-4143	632.725	-11339.4	-2366.45	-3553.45	-8187.6	-125.582	-7835.96	-2955.79	
-8330.23	-2681.15	-4258.43	-6001.54	-1310.53	4112.78	-4074.23	-1445.16	-8967.89	-4469.41	-5536.92	593.844	-5086.8	-1710.94	371.918	
1574.88	-239.682	-4085.83	-11656.5	-942.014	1129.25	-6781.78	2603.27	-4539.89	-1240.58	-5408.34	-3515.9	-7151.88	-4614.92	2126.89	
-3052.94	-6524.44	-9185.02	-7819.62	-2180.45	1824.25	-5770.17	-4274.92	-5440.71	-1154.32	-4944.52	-65.6191	-3797.59	-1217.82	1670.62	
-1493.07	-551.139	-1230.52	-8320.25	-2789.03	-3197.48	-7759.36	-2536.23	-5461.03	-2066.3	-13153.8	-2441.33	-4293.94	-1844.27	1644.55	
-4550.51	-5340.01	60.0723	-7510.3	-5523.93	4039.42	-1806.93	-3612.17	-13843.7	1955.79	-3621.46	-1035.4	-5760.4	105.93	-5071.76	
1122.56	4361.38	1179.03	-10510.6	1083.27	-5770.83	-4140.53	2224.99	44.5195	29.127	-4430.93	-69.001	-1584.74	3369.34	5494.39	
-2495.36	-6515.61	-2752.6	-4530.88	654.121	-3509.48	-3776.06	-3089.17	-5435.73	-2666.2	-4241.23	-1648.1	654.172	-6708.15	-784.33	
-1244.9	-35.082	1025.36	-5881.61	-1751.32	-5366.14	666.307	-3284.97	-4299.9	1066.37	-11603.4	3642.16	-3074.64	-6318.96	3224.23	
-1474.25	-2485.85	2443.55	-7940.8	-2467.14	-9268.51	-6814.74	-1433.76	-5524.13	1422.03	-2630.8	-6315.81	-5195.47	1334.37	1047.18	
-4444.87	1484.56	-4672.44	-3083.67	-212.164	3081.47	-13947.7	2735.99	-7188.34	777.887	-9267.59	-2222.61	-6559.15	-4547.05	-2461.74	
-7960.78	-2567.19	-461.832	-5943.25	1300.34	2475.13	-3357.02	1432.52	-7316.28	-2567.08	-12816.1	-523.319	-10443.1	-2340.63	1273.98	
-4810.01	2159.29	-1650.06	-6595.19	-1123.11	-1064.98	-4514.55	-3983.08	-7687.22	-596.033	-7363.66	-1686.02	-373.514	630.631	1206.77	
-11884.2	-5966.32	-6621.24	-7492.73	-2084.18	-1170.84	-10238.2	867.049	-15817.7	-2568.82	-8363.59	-1399.15	-2605.65	-4456.09	7773.04	
-958.369	-1644.09	-4104.67	4.83008	-1775	1590.4	-664.504	-981.414	-10604.2	-5658.73	-10102.3	-1434.19	-7846.98	595.975	3751.5	
-3717.07	1123.54	8890.4	-6998.9	-4539.36	1111.72	8382.33	-620.641	-6235.53	2079.13	12036.6	13673.8	-8281.39	3590.8	1407.78	

-3773.88	12511.8	-1914.76	-7134.49	8525.04	5986.24	-1614.77	-112.623	-9212.86	-6490.91	-7412.29	5895.13	-5774.58	-5084.79	-1978.02
19849.4	2186.87	-5274.62	10041.4	14235.2	5855.28	2935.21	7618.21	-4455.35	754.77	-191.615	11562.3	605.777	-901.961	3632.84
14878.6	4588.37	-3110.74	38092.3	4388.86	5952.99	69862.9	6050.06	-8461.29	2284.01	118256	9786.7	568.121	5586.84	5351.37
779.34	38943.1	51296.1	-3858.28	3185.88	2197.55	-1532.54	-5647.3	-6041.62	-8307.89	2366.62	51828.3	-2154.81	831.494	3248.16
941.197	56201.9	6000.12	-3132.03	-3681.53	-1716.11	623.48	-55.8105	-6523.18	-3349.41	752.949	10027.7	-2639.35	1494.95	14710
-5091.11	9156.57	-1706.92	-6744.1	-1085.5	1790.56	-5258.02	1668.67	-8524.02	-5929.6	-1622.32	-7429.19	4711.56	11841.7	9531.07
-975.352	2676.53	-6325.94	-5157.27	-964.779	3862.56	1810.96	1872.45	-7219.21	-5275.41	-3611.98	1846.1	-10335.1	16994.7	5656.25
-6279.7	-2107.42	-625.918	-5041.04	-4989.16	-2523.05	-8151.02	-1131.9	-7797.98	-1220.53	-6169.36	4803.92	-3281.51	-1532.48	3568.07
-9520.62	1452.55	-3847.83	-1521.9	-7314.66	478.895	-11634.4	-3256.92	-8328.1	2606.98	-6483.51	6141.6	-2887.88	-5536.4	3443.06
-5355.57	5438.02	-359.264	-7741.03	-2336.64	-2138.38	-3248.32	-1561.15	-14943.6	4165.03	-3924.95	543.544	-5410.38	-1011.04	1446.95
-1318.79	21.4121	82.7246	-1086.22	-5203.98	-3497.33	-3728.88	3676.26	-4977.19	-4321.92	-6491.37	-4186.86	467.666	4530.69	3304.85
-3408.71	6616.2	4324.6	-10136.8	2354.41	-6618.89	-7151.69	5855.38	-1899.62	3495.11	-12462.2	-5828.75	-1695.68	56.0195	-801.066
3613.27	4087.53	-3554.19	-3050.01	-4153.21	-1755.16	-2127.9	-6382.17	-8402.18	-2631.27	-5061.85	-5631.07	-5459.7	-525.605	1869.25
-3195.54	-1733.75	763.826	-1804.68	-2862.88	1853.76	-9279.25	-4165.62	-8061.75	-509.252	-5863.57	-3509.5	-2745.73	1666.97	-642.205
-7648.65	-6795.87	4494.1	690.758	5152.51	-444.857	-12720.2	2512.32	-4892.85	5825.85	-10021.4	-1480.7	-3016.54	1306.31	2229.33
8467.95	-988.07	-1705.02	227.188	-263.186	8796.57	1142.44	18784.4	8623.98	17893	7570.78	2601.58	998.561	7578.35	6583.38
-2823.32	5937.62	10670.8	-3716.65	-6811.95	5966.88	-2293.45	4508.49	-754.154	7725.4	-822.203	2152.09	8907.54	110.648	1059.21
-11153.3	-4353.88	7171.89	12.5117	-1734.1	3972.31	-5359.57	-290.096	-9058.56	-4674.11	-4329.79	570.419	-431.609	-5737.31	-1370.03
1528.12	-2922.29	230.875	136.926	2009.7	-6704.09	-8288.18	-1655.75	-10873.7	-1696.72	-3877.63	-2771.51	4697.79	-969.55	2551.94
-1882.18	-6400.25	-3573.27	-4146.07	754.699	1763.65	-4209.51	-6475.8	-3103.8	2299.41	-8809.93	-7537.45	-9295.15	-3088.98	8295.1
-5443.26	-3036.35	-5003.03	-5016.05	-5924.26	1769.67	-140.912	-1705.99	-4734.36	-1122.67	-7051.33	-2620.03	-10523.8	-1218.84	4841.73
-17882.3	-12256.1	-5136.64	-20731.3	-11385.8	-8919.43	-17392.1	-17507.6	-20969.4	-14474.2	-13333.6	-5788.53	-12120.9	-14255.6	-6963.62
-5230.05	-4676.79	-5797.87	-6840.23	-5847.7	982.537	-5667.33	-6890.16	-8998.13	1785.77	-9001.3	-1365.88	-17572.1	-2150.64	-8817.65
-5812.05	-3608.11	2076.68	-4163.11	-9884.77	134.637	-3324.28	1782.1	-15116.2	-1086.92	-7495.05	830.394	-8470.52	-3874.62	-280.607
-5621.6	2227.76	-2671.97	-4380.29	-3827.3	-4521.06	-2739.62	-1344.29	-13053.8	-3161.11	-4927.95	-1111.79	-2299.33	-3168.1	4329.17
-1884.24	2456.72	-7530.25	1548.57	-2338.11	557.99	-7207.69	-5990.94	-12501.7	-4938.14	-3996.08	1132.19	-2474.69	-4412.94	-4336.89
-304.766	36.4492	-5024.25	-8161.51	-2419.5	967.256	-2435.77	-456.811	-11029.1	-3590.74	-10664.5	-4189.23	-1046.89	7312.06	1637.07
-3315.88	-4080.91	-3229.4	-2634.9	-4694.17	3590.28	-3779.93	-1762.06	-8718.76	5513.69	-1297.2	-3387.21	2241.19	-408.186	-1937.98

-3325.9	-4328.09	-1905.78	-8261.33	-2878.3	3803.19	-5225.2	1202.76	-7033.84	77.5029	1904.71	240.8	-9258.08	-889.652	1180.55
-1253.8	830.822	2663.24	-11233.1	1140.43	3979.79	-6616.38	-2224.64	-13508.6	2891	-3229.36	-11362.7	-2179.03	-2927.34	-2543.96
-4807.32	4789.72	-3778.45	-2255.43	3220.76	1812.41	-7398.83	-6071.62	-8884.24	1677.05	-6285.12	-1270.64	-9909.98	-3701.14	3635.71
-1041.94	-6968.91	-922.576	-4575.19	-8059.94	-272.355	-11835.8	-10010.4	-7603.47	2509.57	-7594.64	-2100.5	-14562.2	-5994.26	-1300.33
-6156.05	-3104.11	-2135.6	-2531.52	774.723	-966.473	-9901.4	-3984.86	-6157.49	1564.13	-7913.28	-1058.49	-4646.03	-8234.51	571.764
-484.041	4080.73	-4734.91	-6566.58	1727.42	-5386.6	-12026.8	-812.375	-6859.09	1139.71	-8733.07	2686.65	-3475.35	-4735.71	2482.6
-865.471	-2545.53	1645.86	-5378.31	-199.582	1974.46	-4438.28	5804.02	-8413.59	-5145.61	-10497.8	1906.5	-9759.1	4985.9	2583.17
-142.629	-2770.18	3895.97	-4274.9	-3182.62	-1024.41	-4475.59	-1913.51	-14340.3	-830.86	-9096.79	-3570.78	323.854	-6744.5	175.102
-3302.95	-4718.22	-958.264	-7816.46	-3981.08	1596.42	-8416.03	6141.68	-8112.25	7161.84	-10873.2	1178.48	1286.56	3128.42	-1028.78
-54.0684	-2320.61	4232.77	-3227.3	-3646.27	-6032.8	-3581.57	5053.14	-17660.2	2805.88	-2696.48	-4681.61	-1535.2	608.752	4332.2
1028.01	538.836	6144.89	-2973.46	-230.703	-1589.95	-2125.18	2116.87	-15866.4	-701.242	-4204.36	-227.857	-6148.52	-3035.6	3289.25
-1749.14	-6423.72	-2993.94	-10433.3	-6487.81	4340.66	-774.461	729.424	-8595.86	3475.65	-14318.9	-4959.78	-2632.46	1335.02	5285.46
-9392.51	-153.055	3782.31	-2126.6	-2944.51	3877.35	-5216.59	4273.58	-6689.98	2423.67	-5941.21	2490.71	-11156.5	-3536.55	1889.09
-412.18	-8621.17	-6392.98	-4176.6	-5059.9	625.467	-6022.97	2952.82	-4389.02	-1164.84	-11867.2	255.342	-4529.32	-4969.09	5983.46
-10054.3	837.504	147.303	-9132.93	1875.14	8488.67	-8877.93	8532.07	-3256.55	-4854.87	-9376.53	-945.826	-2030.62	-2230.82	2623.34
-5154.54	243.418	-5397.49	-7617.77	719.939	7726.97	-5149.94	3265.1	-9793.9	-436.945	-3008.1	3603.2	-7144.54	-4013.48	689.125
1408.22	-6883.16	45.7266	-6833.64	-1227.98	7870.69	-4657.6	-1857.44	-8947.73	6702.86	-7075.81	5832.63	-5025.78	-56.707	1096.79
-8005.7	-5962.95	-1665.08	-2514.18	-3660.37	5390.16	-2570.28	-1232.95	-11020.9	6629.78	-1914.51	-2208.52	-2501.5	614.359	4376.21
4593.36	2257.04	-2709.84	3060.76	5523.85	6909.16	-4897.88	-7054.7	-9696.01	4884.69	-5201.96	-3701.21	821.682	4868.74	-488.617
2888.52	3727.99	16435.8	9014.35	-2155.35	-1301.3	-684.348	-3103.45	945.344	3279.08	8733.05	8877.68	-3231	11548.7	1362.33
-2855.85	2903.73	-1588.46	-2304.68	-6992.7	498.197	-5869.66	-128.85	2601.73	46.1514	-706.926	1491.41	-3474.16	11284.4	17023
-553.059	6382.35	504.088	43.9277	-1068.35	-759.857	-1783.14	-1794.29	-5606.33	4250.07	-7614.79	4704.31	-747.154	645.91	3962.84
-1780.45	1990.5	5219.75	-4967.42	-4963.67	-2598.37	-6345.83	-3533.76	-3223.04	3914.8	1970.69	-3559.6	-9817.82	664.164	12518.8
-5750.55	-629.504	-3910.03	-3557.89	111.73	2632.69	-6662.17	3232.62	-6086.51	4376.85	-5215.54	-4623.87	-579.236	-5221.5	5561.55
-4588.62	-5941.75	-1625.38	-3741.11	-71.1543	-1439.66	-6900.21	8977.59	-9342.49	-679.307	-5080.73	-3350.42	-3499.08	-6942.7	2695.1
-3939.3	3374.65	437.205	-10652.3	3080.74	-4003.61	-6026.66	2213.15	-8858.84	5205.08	-9874.13	-3617.25	-8843.83	-3383.41	1617.98
-5072.01	3568.74	1682.89	-4305.03	3786.1	-4379.96	-7375.12	-2335.34	-12806.7	4396.09	-4260.23	-380.177	-1566.86	749.98	4324.84
-759.68	-2490.13	211.521	-9455.9	-2786.51	-1066.31	-6713.22	-4712.72	-3586.82	6618.62	-4643.97	541.831	-10550.4	3866.09	-3777.53

-4951.48	1678.41	-5802.12	-1576.62	155.027	-4752.28	-10379.9	-4495.78	-11557.5	1602.2	-7328.16	-28.1924	-5430.67	-3510.16	-3128.68
-4362.56	122.346	-2459.88	-5484.33	-1948.86	1182.52	-10372.9	758.352	-7620.06	3009.02	-3612.79	-915.051	907.104	-2768.79	2099.18
4806.91	-5937.25	3389.05	-10346.6	-5100.07	500.111	-4681.38	4666.07	-12706.5	7305.42	-7008.22	2418.65	-7138.06	-4630.72	2882.12
51.6914	-1533.41	921.873	-1747.75	2586.04	-1216.44	-11120.2	-1026.81	-12476.3	4103.17	-4626.72	-4094.58	-6968	-4894.93	204.002
-3756.65	-2987.05	-5011.3	-6027.35	-2744.47	-1217.29	-7009.47	2150.72	-10266.6	9165.31	655.488	-5000.22	-1030.74	3563.01	-5397.4
-2050.62	-6863.3	223.562	-6574.95	2046.98	1962.67	-10371.3	4263.34	-14169.6	-1228.58	-8881.11	-9084.7	-4734.56	-351.574	-8056.68
-11021.8	-2815.28	-5642.96	-8823.08	2011.45	475.982	-9930.62	-5931.77	-9376.36	-3262.37	-2053.07	-2213.44	-3258.78	-4852	1017.52
-4570.96	7305.09	-4939.37	-4817.39	-10740.2	-1635.46	-2691.04	3265.82	-19333	2784.5	-3930.17	3224.73	-8456.12	-2446.86	2968.86
-2658.84	-1152.45	-3814.33	-4827.15	-3721.38	6701.49	-10566.8	2532.53	-5367.72	452.954	-770.127	-3892.53	3842.13	-4196.71	-853.541
-2096.86	-5901.38	-3293.38	-8986.89	1047.82	2254.21	-10921.3	-981.695	-9692.08	831.47	-7470.88	-6665.67	-6506.57	641.451	-660.428
-1579.32	-3601.82	2120.95	-12033.3	-6056.63	-12223.5	-8393.18	-2059.49	-10737.4	-5966.67	-7168.79	38.1592	-5661.42	-7901.38	5115.73
-4956.69	3660.14	-1968.89	-6016.87	-7865.16	-373.277	-5888.63	1733.54	-4499.81	-5398.16	-1011.76	-2951.81	-1756.73	2686.64	-3379.56
368.566	-4071.22	-6084.83	-3774.18	-4052.22	-2996.91	-3075.31	1769.37	-11333.5	484.564	-144.709	-2210.64	-5866.96	3586.5	316.955
-4762.69	4602.74	-4921.41	-11245.9	-3514.5	2972.2	-4837.23	2094.01	-11935.3	3127.9	-5499.49	-1913.44	-5541.2	-7928.76	-6962.27
-4367.96	7524.45	-4984.67	-4811.33	360.189	4968.86	-3592.21	1397.08	-3621.68	-3081.9	-8113.66	2418.9	-2476.8	2456.19	-4081.21
-9881.14	1417.41	1372.76	172.398	-2311.29	-2017.3	-4991.79	1924.01	-2952.96	339.178	-4502.95	-2643.77	-352.225	1992.21	-585.432
-439.775	-993.689	-4198.74	491.18	2494.45	1565.03	-3728.46	-4646.13	-9519.28	9703.17	-3731.82	1047.37	-9605.7	2347.66	-2474.96
4361.4	4596.54	4505.18	2529.35	7737.02	9813.62	6157.39	11047.7	22776.4	35942.5	8082.62	14242.8	6696.29	12370	3999.17
3645.02	18780.2	33996.2	3259.22	3289.65	14885.9	14215	11546.4	18186.3	27753.5	12185.4	23732.5	57860.9	10261.1	10149.6
3799.57	8.68555	-2617.01	-9998.72	2633.85	5303.74	-7202.53	1586.15	-2309.32	1894.86	-8634.54	4792.38	-539.137	2123.66	10864.8
-4999.08	-2914.67	5046.56	-7635.37	-4178.25	2814.4	-10107.6	289.178	-5838.96	837.156	-3432.62	-387.594	-1228.06	-6164.75	4979.96
-1295.66	603.076	789.539	-7758.63	-3547.37	-206.1	-807.277	5622.58	-6565.47	7613.25	-4708.11	5127.97	742.963	-2826.24	536.023
994.826	4400.2	532.699	-3467.86	-6405.69	-922.959	-13355.5	1143.51	-8115.94	1549.32	-5199.91	1861.23	-2044.25	-446.569	7431.15
4198.48	-2361.98	1991.55	-4508.66	-2436.8	2524.61	-2039.32	2444.95	-10969.5	288.217	-3797.15	3959.96	-2688.04	-3518.27	8159.21
-4396.52	-1089.64	2547.99	-10027.9	410.742	3687.65	-1811.18	-6389.56	-7308.93	197.558	-5056.6	2459.64	1865.25	-1814.78	5645.57
-2777.3	-1549.29	-207.576	-1774.88	174.865	5486.31	-3987.78	-2382.44	-9491.64	3476.87	-4819.08	4130.43	-5005.04	-2241.72	594.609
-189.328	-646.635	3249.16	-6721.65	1883.49	68.6582	-1070.85	4804.23	-3628.81	5162.98	-1218.06	-2345.37	-2599.73	7527.24	-2100.66
-4211.19	-1605.3	2310.94	-7505.03	-3320.32	-698.205	-12707.5	3961.95	-3199.22	264.028	-6771.82	5022	-6793.12	6857.55	6465.42

-3149.75	-1648.13	-26.7988	-7799.68	-8317.64	-5785.41	-4647.31	-6214.59	-9464.23	-903.72	-8171.41	-7520.45	-1141.01	-4454.22	-5769.25
-8055.4	-4584.06	-1478.46	-11083.3	3150.32	-86.0684	-11139.9	-4191.18	-7936.78	-3327.7	-6086.31	943.348	-7078.23	-5989.75	-5489.84
2499.41	-2886.66	-990.492	-9038.38	-7993.14	-2769.85	-13735.2	233.105	-7296.03	-4834.89	-7501.72	-305.302	-7934.22	-344.352	-5499.3
-3841.28	687.395	841.559	-6510.8	-6070.53	780.221	-14323.9	-82.0742	-6146.6	-4229.78	-10649.8	-4608.9	-4936.18	-5524.74	-5158.84
-2754.99	-1.64062	-4058.81	-9528.49	-3318	-6916.25	-6458.89	-5924.63	-13288.2	5982.94	-4558.37	-1060.94	-7091.26	-4836.39	-4132.23
-1970.52	970.238	5495.66	-2358.05	-1820.27	-3986.36	-11275.4	-4874.22	-10911.6	-1335.82	-9856.69	-1978.17	-3310.17	-2282.55	-5738.51
-7061.79	-7584.53	6969.14	-9339.76	-911.461	4433.41	-8662.44	-3061.92	-6076.71	-5555.73	-9746.46	526.995	-6369.49	-6861.68	-390.02
1740.55	-3294.53	-2522.35	-6986.27	3532.39	-3940.24	-10338.6	1657.05	-11061	2687.38	-2957.07	-1188.55	-1281.31	352.32	-180.01
-8215.85	-4656.65	-5554.34	-12626.8	-2795.04	-865.467	-14235.1	3048.68	-9259.1	7734.63	-6049.29	-1658.83	-7828.88	-2507.98	-1278.37
-2792.04	732.896	1227.75	-10993.9	-4250.21	-5293.28	-2920.72	5698.62	-9384.64	2978.89	-2479.89	-1615.68	-6068.06	2140.4	1396.5
-5489.33	1787.12	3465.59	-8667.94	-4598.43	-6596.01	-10471.4	-9111.6	-5114.21	4901.97	964.066	-444.454	-3599.31	2369.74	873.533
-10275.3	-7061.88	1112.03	-7192.77	-5936.74	-1627.31	-9689.85	-94.5137	5967.75	196.818	-9109.61	-10710.7	983.861	-904.333	-5826.4
1542.54	-7034.09	-605.93	-10708.3	-1126.3	-1925.33	-6278.83	-8554.12	-9703.3	803.108	-4299.08	1004	-13440.9	636.289	-5571.75
-2735.51	-15633.8	-3129.37	-9647.99	4085.68	-237.992	-4910.95	42.4316	-10324	-1195.86	-2703.65	-116.257	-4971.98	5147.78	-5824.56
-5920.34	-44.3164	-4018.12	-3791.32	-1445.28	1473.12	-3677.51	-2708.72	-5920.86	-507.959	-8623.52	838.622	-3830.32	-3794.81	-1929.5
-9603.19	-2415.68	-408.699	-12828.4	-1141.87	-583.941	-12712.4	2775.51	-4501.4	-7486.5	-4769.08	-746.454	-9799.01	-4810.73	2786.91
-8797.69	-6892.94	-796.402	3347.5	-561.406	-3771.73	-3312.73	-2711.98	-12947.6	4224.78	-4133.43	-7568.45	-8176.93	1674.66	6470.59
991.33	1657.73	3064.01	1416.31	2165.1	10678.9	-7945.2	16564.4	927.896	20492	3749.74	1012.7	-2041.03	2696.87	434.803
-7981.82	1590.14	1545.07	-6544.19	-2647.71	-3141.29	-10578.5	-2717.67	-1116.55	1394.55	-4454.17	-728.846	8469.59	-3285.46	-739.939
-6280.57	-4223.06	2888.24	-7416.97	-5630.49	3167.81	-5828.09	-837.117	-4498.92	337.035	-5021.94	5560.96	-5778.43	-2594.42	2247.5
16476.9	11182.9	6850.83	12580.3	7025.2	6189.8	6451.66	14253.9	5017.12	16624.3	5376.06	9394.25	13510.7	12031.1	10518.6
-1237.22	-820.404	-1082.69	-3347.76	4730.68	2995.97	-11572.5	1589.82	-10730.8	-1510.79	-9742.99	-1335.84	-1003.38	4042.73	2144.17
-1066.66	-8026.26	5288.38	-3556.21	-2763.05	4545.87	-9090.2	-1901.05	-14199	3364.05	4475.71	1993.69	-5548.75	-3510.42	7416.5
2037.07	705.104	2083.39	-11403.2	1456.29	-2137.36	-13210.6	-4331.15	-12892.4	-725.209	-7033.19	-1824.18	-3602.53	-12228.4	2168.39
-3869.53	-3266.76	-1111.7	-3360.12	-9636.39	-2986.04	-9652.99	-2359.62	-4466.73	-194.216	-11286.5	-4075.28	1181.47	-1178.26	1277.12
-6322	-5237.13	-2639.94	-6275.49	-6552.55	-5990.78	-14563	393.85	-7929.75	-2121.84	-6145.03	-5957.83	-6469.69	-3349.4	10578.2
-4494.04	-1320.24	-4662.98	2892.4	-3983.76	-4489.37	-8351.54	-7646.91	-7900.1	-483.55	-9302.74	-717.1	-1661.95	3693.67	10984.7
-12500.4	-10028.5	-3721.22	-17910.8	-10791.9	-2407.73	-9171.14	-1406.19	-15063	-5470.17	-10621.7	-1105	-7856.28	-5793.97	-503.975

-2690.8	1807.83	-1901.09	-5655.76	-2998.74	-1516.71	-3729.65	-3299.35	-6836.36	1359.18	-11512.6	-888.854	-5998.56	-288.393	2889.13
-7956.47	-788.086	-5324.69	-2159.76	-5097.16	-1403.94	-2541.64	85.7227	-4757.71	3397.14	-6297.43	-419.767	-5056.66	-226.123	4451.26
-4210.75	1104.92	-3563.49	-5972.79	-5445.14	-7769.23	-8910.92	421.215	-8688.94	-2602.09	-8489.77	3693.04	-10316	-3464.4	-4799.44
-2106.84	-1567.15	-4820.47	-6973.08	2005.33	1483.43	-7379.51	-1466.75	-13271.4	3824.66	-7094.64	-1573.86	-3512.93	-1232.58	-1655.41
-6859.48	-3124.34	-1989.12	-8228.04	-550.41	-7188.6	-5786.35	-1091.9	4278.64	6115.36	-4592.91	-1377.28	-11794.6	-5747.77	3186.17
-9740.04	-812.871	-2227.28	-5706.1	-7180.58	523.156	-10188	-5154.39	-7868	-2424.54	1100.33	141.238	-4592.75	-3055.19	-359.686
-7988.78	2160.51	-927.146	-5657.67	-1517.38	-2509.93	-4767.03	-5997.41	-917.26	-5126.92	-5498.43	5381.25	-5502.39	3942.05	2817.11
-4650.49	178.508	-4393.58	1362.51	-6167.71	6575.05	-3264.22	-3963.32	-4508.82	978.924	-5014.99	47.335	-8261.72	9501.37	-1863.39
-3961.33	1272.31	-2065.3	-2619.97	-4890.86	2566.63	-9689.99	-42.5273	-14451.6	3552.46	-11356.8	1120.71	-5544.53	1743.21	701.992
-6274.8	4187.92	-5274.46	-13837.7	-5199.61	-1866.37	-6972.43	-108.217	-15074.6	1296.03	-1052.88	1830	-390.139	-4764.62	-4539.14
-5818.85	-9208.36	-3344.92	-12075.5	-7718.76	1526.23	-10030.6	-4422.2	-14686.7	340.212	-7417.18	-3931.46	-6102.01	-3667.27	3771.1
-4746.67	-1683.36	-7870.9	-6384.51	446.275	-1031.65	-7791.62	-12415.6	-8520.23	-4482.45	-11075.6	-3193.72	-7074.78	-3153.57	-9362.76
-7699.88	-5084.53	-10182.3	-12514	-1151.36	-4122.06	-5358.42	-6601.51	-13001.5	-12.5654	-5556.58	6211.38	-13166.3	-4201.36	-254.383
-2601.75	4016.85	-3931.84	2411.69	-3261.98	942.018	949.863	2981.67	-2110.69	8250.67	-2839.13	1839.38	-276.021	3943.43	7281.65
12400.4	5045.13	2899.73	606.76	6013.72	4334.23	2120.15	8852.04	-23.7246	5419.48	-4563.24	1195.52	2102.3	801.846	1250.05
5507.16	894.76	4889.39	-3412.54	4614.07	-334.533	-9206.64	415.928	-1392.46	2635.97	-1571.92	-1550.21	1575	10543	-3551.67
-221.887	8233.5	1021.82	-4287.79	7512.36	2718.44	-6589.98	-1676.28	-4413.49	6775.9	-634.629	2447.64	-917.85	2211.62	13472
-2084.15	-2756.24	1106.86	-1627.46	-981.793	2000.45	-8295.33	3817.79	-6450.42	6093.39	1388.86	3461.41	-6067.2	-208.756	5290.57
2050.18	6156.28	1915.61	-27.2129	5669.05	-1763.56	-3858.19	1276.52	-4450.4	8257.61	-6732.44	-4571.18	2843.99	743.227	7774.39
-2327.2	8869.56	1092.32	-2757.6	-823.811	-2991.39	-2627.12	-1478.29	1177.95	4870.47	2679.99	962.871	5320.03	737.672	903.002
-4924.54	6116.61	4849.65	-624.064	-6060.18	-1115.4	-3984.92	1785.26	-7389.8	-899.03	-2925.92	-4513.01	-5870.15	10379.9	-2468.72
-4110.7	3626.36	-1218.52	-5685.23	-3243.89	-544.699	-9173.2	-1175.59	-8463.56	401.992	-6466.2	-3459.72	-5726.07	3107.88	33.0566
-2115.4	-736.602	-16.4668	-2083.21	-4069.56	2679.34	-6665.44	-7894.9	-5621.1	-3146.67	-7642.03	-6110.06	-1741.29	-2842.28	3570.99
255.814	-3989.45	-1563.82	-5227.23	416.027	-5029.62	-10163.6	-8224.96	-7301.24	-2527.9	-4183.73	-441.275	-2446.47	-110.527	3927.5
-6450.45	-1089.76	-2883.86	-7111.57	-282.455	1482.1	1137.8	607.924	-11721.6	8200.82	-524.801	-2234.85	-2376.64	-3158.68	3734.04
-2639.8	-1493.74	-6033.85	194.254	-3201.73	7858.76	-7023.32	-4492.7	-9572.15	4803.78	3667.26	-4402.24	-6453.62	-1439.02	2450.45
-2258.93	-6937.93	-1952.67	-5727.03	3584.03	3063.72	-2908.79	-1971.4	-8810.32	-2187.94	-4316.69	4398.21	-7640.74	-2293.17	2342.99
-4279.4	-2686.53	2953.18	-2513.55	3737.11	285.508	-2833.33	-5294.38	-9240.33	5478.13	-4110.44	-1268.28	-6861.84	-2918.91	-120.883

-6286.25	-240.168	5024.5	-3653	88.5977	-298.092	-7138.71	-3592.79	-8027.65	9660.19	1139.66	-3819.5	-5657.87	703.67	3647.55
-7514.02	-2159.32	-2917.6	-9290.93	-5201.46	966.201	-9617.73	3256.77	-10885.1	626.738	-1298.41	-2515.86	-170.791	-4770.36	-5126.13
-881.672	-1101.49	-1561.36	-10035.1	2637.65	-4749.31	-1485.11	-1267.61	-8303.36	-6119.89	819.648	-4091.43	2006.29	-269.878	-669.998
-1856.33	-4529.02	-1849.47	-1227.87	-4870.55	-7626.01	-7024.43	-724.131	-12047.5	464.476	-1719.94	-5955.3	-5155.04	-2712.57	-4910.87
-2004.89	1664.16	751.35	-5068.79	-221.373	-2131.81	-2162.12	3102.29	-6025.85	7197.47	-12803.9	1608.99	-7424.32	-1161.76	970.67
-2915.31	6086.54	2572.31	-7786.92	-7296.95	-3841.34	-3180.49	3914.25	-7963.66	5459.18	-3861.9	2896.71	2322.65	861.299	1597.49
-3087.81	-6307.33	10139.3	-12202.5	4347.21	-6063.78	-19477.4	-10368.3	-7239.96	-575.464	-6141.74	5366.46	-3909.71	-5170.16	2542.97
-3684.79	-3851.13	1553.26	-3868.56	3029.44	-485.072	-7477.09	-4303.85	-6128.36	3308.29	-5662.76	-747.536	-7182.98	-7990.56	624.754
-814.369	852.691	-168.074	-5132.12	1522.24	-1782.59	-3166.58	-2137.14	-12160.5	3600.06	-1361.67	731.711	-3792.74	226.635	4649.91
3017.32	-6184.38	1889.7	-7872.96	-3386.5	3923.36	-5526.16	71.9805	-9177.63	-2375.13	-14392.5	-5395.52	-1622.29	-1316.83	5277.46
5059.77	-3131.78	2360.7	-8177.07	3015.05	11020.1	-7294.46	4187.54	1351.05	7782.71	7105.62	3950.36	935.318	4986.73	-778.979
6730.58	2086.67	5473.21	-3119.04	-1775.25	8221.82	-6657.53	-1087.43	-9529.8	286.37	-4983.52	-1333.59	-5884.47	6518.49	4782.71
-3306.33	1915.18	-672.533	-3596.56	-226.049	-14.1543	-5495.19	1118.52	-5182.65	-2925.4	-8651.95	802.054	-4813.54	-2613.15	4714.8
-5162.72	-2892.49	-349.328	-6084.28	-4374.43	-1531.32	-16084.9	-7098.23	-12321.5	379.023	-4423.51	4640.24	-3455.05	-4070.9	939.391
-6346.66	4020.28	-4602.18	-2997.63	-5439.59	-1128.3	-11601.3	-2587.6	-11492.6	937.048	-784.344	5312.78	-5578.74	-5698.33	993.029
1731.71	1865.36	3765.03	-782.83	937.99	2883.93	-6121.03	-2885.71	-11206.9	1198.09	-6964.38	-7980.56	-3139.66	2254.33	4493
-7589.37	5660.76	-337.207	-4429.28	-598.768	-1832.01	-11490.5	-1387.64	-6696.68	326.179	-2389.6	-862.068	-6856.1	3815.84	353.723
-5606.02	-3433.58	864.887	-2250.52	714.818	1223.68	-6637.93	4805.68	-9195.01	7300.03	-3107.44	1145.2	-6398.15	-1456.34	3038.39
1007	6102.12	-900.283	-6120.08	-2261.27	-4636.83	-2610.1	-1341.19	-13895.1	3661.58	1337.14	2069.6	6411.1	2559.34	-1132.63
1997.02	4828.84	7161.04	-7541.45	-1015.64	-1292.34	-3904.12	4932.77	-10930.4	-646.44	-6607.53	577.002	1754.91	680.148	1626.14
-2199.09	-4632.56	-3540.56	-10667.3	-2281.48	-1893.91	-8527.76	-5289.23	-4777.67	2458.98	-12491.6	3793.55	-3769.64	-5839.4	-1272.56
-4918.11	-5755.16	-3359.47	-13728.4	-5447.72	-63.9355	-10065.2	-2617.81	-7910.42	-3216.8	-669.143	1914.19	-8753.89	-3050.86	-2202.55
-673.928	4878.59	-299.057	-10311.7	-676.824	-2890.36	-14643.7	-586.865	-8515.35	145.783	-2593.75	-3696.02	-5626.88	-1107.67	-2584.08
-1804.73	-1750.4	-3477.26	-14070.4	-3652.38	-2054.3	-3542.52	-494.373	-13355.7	3170.7	1510.85	-4890.31	-11595.3	-5664.79	-888.229
-11936	-5825.72	-1483.39	-14771	-3773.05	197.203	-4081.8	-2707.36	-11181.1	-767.012	-3249.07	-2123.12	-1050.11	-2482.97	-5622.04
-7342.39	379.584	-3814.17	-7331.19	-757.441	-4770.5	-1541.85	-5293.33	-9630.47	-4732.3	-5352.08	275.362	-4671.06	-2861.52	-4083.94
-3047.26	2564.03	-1702.39	1740.53	-4262.93	9382.2	-1381.12	8610.2	-10481.9	7414.65	-30.9531	1687.81	8297.11	1925.83	3708.84
9068.23	1196.92	14249.1	-2419.07	-1710.65	14880.5	1515.96	14442.9	1439.35	6929.01	2249.45	10847.7	8913.21	9413.71	9027.64

5474.1	-3907.69	12446.7	-1542.24	4302.25	320.867	-3350.22	2663.87	-7323.27	7996.79	-1689.58	5090.32	7528.21	6078.64	6485.54
2483.09	3836.03	5422.66	804.656	-1423.54	4187.46	-7577.74	4037.91	-3413.62	6561.68	-11231.4	4564.57	1249.46	3977.72	4793.77
4605.91	3366.18	4961.52	-1360.13	2996.51	4921.52	-3924.97	2177.41	-12619.9	8083.42	-3686.33	1772.76	1907.24	2010.94	6979.61
1083.95	2985.98	510.684	-67.6621	4084.85	2497.18	-1520.79	1401.11	-3202.75	6306.65	-5920.63	5155.52	1026.11	346.506	850.498
-4333.49	3388.38	6718.34	-5232.4	-1304	6432.5	-4792.38	730.184	-1499.36	4511.64	-2546.41	12331.6	2593.95	2059.05	1214.69
-3041.61	1891.27	5670	-11561.2	-5113.06	5762.9	-2099.55	7283.48	-1306.64	9808.83	-3263.85	5745.77	5907.83	707.764	-1468.35
-2203.02	8010.43	7961.56	-3156.79	1335.53	7669.92	1894.31	3585.01	1913.42	9421.48	-7090.76	4075.96	405.906	727.424	7448.62
-7517.08	1995.46	8885.83	-4882.4	-3725.54	-2032.44	-1600.09	2964.48	-3821.76	13774.9	1215.43	7045.98	1596.25	4084.4	5007.03
-2846.2	3696.1	1648.91	-7432.17	375.982	3310.42	1392.16	9509.92	-6832.74	10696.2	-3408.19	7636.2	-774.48	9467.16	780.883
4529.62	6746.27	2550.34	-4409.9	3772.01	1232.14	-4503.49	6556.72	-5210.62	12413.6	4804.13	6157.31	515.506	12153.4	7554.65
8336.93	3593.86	7206.35	-3824.29	-482.211	7570.72	-130.66	3742.48	-1046.85	14608.4	8877.01	6504.47	7623.78	5094.89	7581.33
3983.43	8476.01	10118.7	-5456.74	3063.75	9180.96	4327.82	-539.527	1667.92	12853	5877.79	11892.1	4045.85	6569.49	10579.3
2940.41	955.447	16690.6	-3434.07	7244.63	3776.15	4583.62	3409.9	6717.87	15134.4	4453.67	14854.7	15610.2	9840.13	9894.37
12256.8	6888.78	10520.3	4419.36	11360.6	13801.9	5057.13	14451.8	2305.44	16750.7	12410.3	23790.4	18017.2	9331.54	15922.4
13359.6	21831.3	22229.3	-1506.67	17949.2	6867.78	-3963.2	5514.94	5056.56	16824.2	17638.7	54517	17687.6	7991.63	18352
21300.7	23004.5	29682.4	-5057.49	15059.7	15400.1	6682.31	13574.3	6454.72	28630.8	18167.1	122414	24473.6	15244.6	16514
22996.9	43255.7	41458.3	4752.81	39657.9	26212.2	15239.7	22812	17346.9	44147.2	29564.7	237812	30994.9	31314.9	22307.6
132746	180723	133815	89876.9	239690	109065	112974	109097	120377	169371	137590	568627	128367	118163	106411
1.55E+06	3.40E+06	539108	2.02E+06	3.67E+06	844027	4.28E+06	3.09E+06	4.73E+06	8.12E+06	3.49E+06	3.12E+06	508046	2.72E+06	686849
8.36E+06	7.33E+06	9.47E+06	7.01E+06	4.27E+06	7.42E+06	4.80E+06	5.36E+06	3.94E+06	1.29E+06	6.78E+06	4.71E+06	8.64E+06	5.73E+06	7.24E+06
132826	208564	144862	146114	198422	149888	125930	128100	114263	92492.5	120527	132958	122344	111401	109367
33293.2	35538.7	19408	31904.3	21684.3	33852	31771.9	27178.5	18430.4	11468.8	13721.2	18102.6	21897.2	26691.1	15728.9
15937.4	8862.46	7858.6	18961	5064.42	15563	15476.4	16419.8	5180.29	4095.2	6257.4	8245.16	-657.92	11054.2	11224.3
4127.46	3154.44	3046.49	3410.83	7439.27	5679.84	4724.96	7494.08	-6041.55	-0.341797	5320.36	3521.56	-508.219	-3983.36	8964.87
4548.54	4529.17	791.447	7450.77	-261.031	2894.13	9816.61	8438.38	-3277.06	6059.56	2501.31	-257.122	-2626.98	8538.86	2835.49
-479.045	5999.4	544.225	10938.3	2991.71	3746.89	3530.4	1082.2	-3897.96	7758.38	1745.66	-1375.55	-7304.09	3348.22	2116.29
-3259.59	4545.27	-330.492	6128	1674.81	7200.47	5031.05	11188.4	-4972.57	2760.02	2120.81	-6381.01	-8534.28	4391.57	7261.91
4974.2	-1541.27	2823.47	-865.41	-1162.14	5305.1	1471.96	992.617	-4649.4	-92.8848	-7238.04	-3538.74	-8123.98	5605.72	3700.35

2575.83	-3599.75	90.9512	-2579.62	1421.82	5226.51	9117.11	5614.01	-5113.28	4961.13	-7702.29	-2658.31	-4461.75	3585.75	3424.27
-5950.74	1527.09	-7255.25	294.934	-3699.81	5307.04	4655.21	-2334.78	-5285.56	-664.941	-6793.38	-1202.71	-8030.56	6652.52	-5214.24
-8247.25	-1001.83	1161.86	1502.95	-3572.76	5811.79	-176.273	8297.99	-9239.21	3255.59	-7380.11	2635.38	-3265.03	-579.739	-2727.01
3113.34	-479.455	-2268.93	-1987.74	374.744	5416.59	-9116.08	2618.53	-2456.72	3480.01	-4530.42	-1706.97	1357.39	1374.61	3903.85
-3140.16	4127.35	-2123.32	4549.64	-10472.8	5084.31	-3943.63	-3685.79	-10158.9	831.506	3089.3	3958.23	-3953.81	8131.57	4150.38
2005.36	2455.47	-303.35	-5798.37	732.238	5743.77	-2360.89	2550.52	-10934.6	-2396.07	-181.021	-1704.29	-6467.91	-454.444	2339.8
-4599.3	2425.7	1625.19	-2975.84	-2556.77	3878.04	-703.877	2115.81	-3925.96	2422.31	-6898.42	912.955	-4872.04	-3159.88	1398.99
1480.08	-6177.72	-1995.79	684.23	-4481.45	949.811	319	1752.71	-4679.64	-8839.92	-2013.84	1946.16	-1872.96	-5188.2	4706.5
-215.301	594.875	4629.05	-289.953	-1981.47	5950.05	-835.141	5457.53	-6361.66	-4027.53	-153.4	2009.33	-5276.12	-2199.66	189.105
-1761.59	-527.203	-1684.95	-865.18	-262.291	1165.58	-2092.16	6156.68	-2088.72	4054.32	1666.75	-4387.57	-3064.23	10447.5	1982.58
7806.04	4365.33	9562.69	3746.62	6755.93	11765	-3302.72	14196.2	3047.61	13219	9272.74	-6107.24	-1190.59	10922.6	9022.89
1439.81	1513.08	2031.05	6246.91	1442.29	6897.65	-6271.83	20313.1	505.238	12526.9	-5513.5	4428.76	9564.25	872.643	5224.25
-7285.89	-8102.44	1023.96	-9363.53	-1877.94	4004.7	-5187.43	1689.95	-13719.8	-7248.23	-3298.61	-5403.63	-7749.12	-3662.63	1910.23
-8012.91	-5186.98	-3383.15	-9426.89	-6005.24	-3899.22	686.254	-6223.23	-4269.88	583.791	-14961.6	-4856	-10071.8	-3431.1	-5765.44
-10643.9	3570.75	-8383.39	-13557.4	-14.543	-774.639	-8015.43	-5894.24	-13465.9	364.215	-6427.08	-8233.89	-4774.11	-3364.6	2945.14
-7890.8	-5796.13	2485.8	-6104.62	-5734.75	898.033	-7620.42	-7755.79	-6447.58	-2243.59	-3232.85	-4888.53	-4142.5	-5399.08	1655.18
-858.791	2926.63	-3924.85	-8206.87	-565.146	3758.01	-3340	-72.498	-9529.24	2008.75	-8876.65	-1322.36	-2467.95	-2664.91	-2550.31
-629.945	-3577.25	2253.76	-2641.72	1250.87	-1385.39	-3445.98	3219.08	-12657	404.171	-11567	-115.467	-10379.4	-4854.77	719.242
1401.92	13230.4	4059.9	-1205.46	4264.05	8083.49	4317.43	13717.5	9289.56	15105.9	9027.89	6154.16	14393.1	7325.59	7450.7
-1955.07	1841.85	-1747.38	-5839.18	5149.81	4226.4	-3424.78	-1144.9	-1874.75	1471.03	-3502.21	5672.61	3077.33	-585.867	5605.16
2340.68	-618.295	953.229	483.598	3640.66	1866.52	-2162.12	-654.535	-8986.39	2414.4	-3205.71	5888.64	-8128.32	3606.06	7281.51
-2264.64	1375.64	-1994.96	-9859.07	-3163.05	2032.37	410.822	3165.07	-5999.99	4719.43	-5677.11	2565.31	1626.87	4215.32	29369.7
21421.9	7576.66	249.314	2920.82	458.857	5752.92	9094.93	186952	9258.79	18948.9	4114.98	17201.8	11979.6	16346.3	34281.4
3187.48	12223.8	1328.45	-3612.91	-4336.84	8508.92	1219.4	85958.5	-3824.9	10810.1	6594.56	9400.99	71833.1	7292.16	20626.5
18896.1	6501.09	7301.48	8361.12	2854.58	12571.8	30279.4	383056	12483.1	4715.63	36309.7	29535	114337	18732.2	32407.9
2462.92	6296.88	6002.46	4080.11	-7591.43	10332	-3768.39	21635	-11651.8	6195.98	-5388.57	11666.4	9052.58	9113.88	2660.17
3323.27	6548.5	4238.85	-2098.9	7285.65	9518.92	6496.75	13461	-361.877	12822.4	5901	9898.51	4945.54	6858.67	2472.57
12969.4	19768.6	7569.05	3557.59	3939.65	7775.17	9006.49	45229.5	12263.5	24698.7	14452.1	13852.7	14976.9	6188.1	7123.73

9989.86	16819.7	37675.7	1177.24	2700.71	13154.1	8898.77	28456.7	28448.2	53868.2	3300.01	11463	54166.7	22857.4	15798
-876.928	2728.87	3621.92	-5081.22	9376.69	695.826	1591.75	8244.68	-4049.65	6157.09	2752.34	11275.2	14243.1	6604.43	1000.41
-2064.17	-2559.93	-2333.9	-4990.76	-1042.86	5535.65	871.258	13442.1	-8076.76	1158.1	-450.678	-717.641	-292.758	2284.73	8179.76
-7410.6	-801.27	2424.36	-3074.83	-3912.92	552.527	-1427.47	4612.62	-5800.83	2453.99	-4024.46	5750.88	5637.5	-654.238	4422.36
-5346.33	4944.35	2444.43	-6364.2	1737.46	-52.1211	3266.37	8918.85	-1448.4	5362.62	1885.47	4099.77	-4383.03	-20.957	-1690.53
-161.484	3753.81	425.998	-1861.09	-4015.31	2867.56	8203.08	6991.88	1169.27	6451.45	-3514.93	5956.37	-7406.58	7822.73	9354.26
5695.76	10641.3	8679.19	3545.99	1709	9735.74	-5502.41	12652.2	5682.57	19973.2	-381.674	7767.76	17461	16963.2	9763.33
-5362.57	13080	5293.11	470.775	1342.12	4861.17	855.461	1206.53	3588.58	-2802.13	7184.55	13310.8	-9630.02	6409.88	4353.66
7431.1	11990.4	7495.33	6861.17	2622.69	5636.6	2724.79	2624.16	4155.31	9781.65	6397.91	10761.3	17974.1	11262.9	6709.33
4559.53	4250	10225.6	453.633	3008.68	5374.13	-962.883	2666.17	-3234.91	8254.7	10193.9	8804.34	6494.19	2739.5	1519.38
2001.92	2111.45	615.279	2369.59	706.707	17145.6	7247.65	14557	5332.71	12765.9	1967.4	8054.79	4574.89	5388.61	8623.22
4141.82	7826.55	6614.18	-4889.36	4148.88	10963	3127.68	7698.16	-595.799	13499.7	1502.36	20103	5658.08	9245.36	5149.45
19167.5	33085.9	16660.6	7628.82	39752.8	24529	26027.1	163425	15944.6	38193.2	30648.2	52861.7	48614.5	24044.2	42736.6
222223	307900	45762.8	444397	437545	173783	551888	578841	772641	1.15E+06	307110	383808	85571.2	584130	188759
797635	768925	834070	495636	374090	858469	223267	380079	230447	123852	676508	321909	1.08E+06	508634	828772
35429.2	37948	33343.4	23845.4	28844.1	37639.2	27159.4	165063	20930.7	25237.6	35968.9	29230.3	84253.2	30686.8	50194
8640.67	13339.6	5198.45	198.275	5019.51	1623.16	3430.21	18566.2	785.174	10655.6	-1362.83	3207.39	9009.22	7296.7	9471.62
10247.1	6871.83	8634.48	3002.54	-720.447	15129.8	-2265.27	25133.6	942.375	10086.7	2136.3	5874.01	6745.57	7414.04	11326.1
10602.2	6247.98	2622.82	-420.729	-2131.42	13538.7	826.131	54345.8	5165.58	10408.8	-668.154	9351.64	14994.3	10752.2	11154.3
9354.42	10456.3	514.578	4725.85	3222.95	13906.2	-362.812	14923.2	-2000.85	4723.5	-1446.28	8090.05	3315.05	-561.955	-425.625
-1220.74	4528.08	-877.988	-2607.28	-2866.45	4096.86	-3143.39	14361.1	917.275	12405.9	-552.168	2014.72	7143.43	358.109	6354.93
16623.4	28265.2	8266.17	7120.74	11797	11615.5	27074.7	416903	7939.86	18305.6	29918.2	30756.7	129666	19257	87864.5
3829.22	7006.94	8006.67	-912.914	2190.25	6215.35	8307.7	19242.1	-1750.11	8887.5	233.787	1055.94	4021.56	5828.7	7578.6
8086.92	3607.59	-1497.37	3954.34	1859.48	2562.49	11064.1	13073.8	926.939	11967.1	-396.092	952.141	4884	8158.51	5042.31
15545.3	6738.28	5440.42	3207.92	-30.459	1893.06	5344.77	8886.96	2063.93	9039.24	4396.29	13877.4	2779.08	2620.41	6695.84
-4398.24	-6179.67	-826.246	-5524.02	-2130.04	-3540.82	-6023.21	728.662	-8306.16	-3597.38	-3976.13	4014.25	-3191.68	-4492.31	-4292.41
-3588.46	-6007.6	-11582	-10808.1	-1712.11	-11705	-7225.16	5991.85	-7669.11	-2202.79	-795.873	258.994	-6412.11	-2393.06	-994.747
896.121	5114.33	-2450.58	-7116.85	168.545	-5748.33	21971.2	390347	-7490.73	3228.67	13047.4	25887.4	15801.4	2521.12	54962.1

6839.77	10537	6044.79	-6549.3	-5550.09	2003.5	-2262.52	52073.8	-13484	-4572.74	-324.783	3028.31	101937	-4660.19	23077.2
-5594	-2517.8	-2800.84	-6944.57	-4434.92	-1176.03	-6205.71	3398.7	-8449.61	-69.4346	-9734.02	645.698	-3171.35	2687.95	2489.33
-1044	-3595.08	-4085.28	-3546.38	-6273.05	3608.61	4188.64	6152.15	-8837.44	-4059.77	-9279.32	-5433.42	-3603.31	-63.9297	-1350.96
-1283.61	417.066	2922.02	-9075.14	-5809.63	-193.285	-10339.3	-248.488	-7810.78	6343.73	-5425.01	-3628.34	4427.01	-5677.57	4182.07
-5432.37	-6446.78	-2431.06	-6187.7	-2518.55	461.283	-5669.45	-2617.89	-9962.29	-3829.85	-10532.7	-5727.34	-5256.12	-2861.29	1328.75
-6038.08	-5476.66	-1553.53	-6080.29	-5829.23	2653.85	-5590.54	2524.54	-5735.1	-7338.78	1530.21	-1440.5	-10346	-5691.96	-1978.81
-6378.71	-6083.99	2497.12	-2396.92	-4214.82	-6146.03	-4769.58	-182.459	-5384.54	3160.28	-4874.15	-4501.29	-6985.99	-2205.45	-252.086
-5586.79	-7376.5	-1371.64	-2760.7	-1562.89	493.926	-4571.49	-9102.75	-2018.83	2337.59	606.732	-97.7236	-5069.99	-4643.81	-2377.47
-2007.61	-4536.58	-1166.18	-10132.7	-11124.1	-453.539	-4101.57	225.273	-4625.09	-6726.25	-844.377	1486.81	-3763.86	-987.49	-5716.99
860.129	1339.5	-833.361	-5451.53	-46.4375	-2720.21	-8911.41	1229.19	-7661.56	8470.58	-6946.67	-949.591	-5956.23	-2296.56	-2206.93
-807.971	-5235.58	295.254	-13209.8	-5419.14	1451.15	-5637.33	14958.8	-7544.46	3817.5	-10172.4	196.543	1018.74	-2157.36	1446.47
-3470.21	-2266.24	-5521	-2487.67	-7750.37	1067.79	-6452.44	-6374.52	-13255.1	1133.47	-126.209	-1781.14	-5500.47	-1690.5	2436.61
-4472.59	-3819.3	-1434.07	-9400.74	-2907.57	-498.994	-3675.87	85.6621	-7680.03	-2962.66	-7188.98	-5562.4	2427.7	1217.55	-7590.51
-679.766	-7345.58	-737.229	-7706.18	-3537.9	-1391.49	-6257.6	7671.29	-4851.88	51.4316	-3515.43	-2786.73	-7565.69	4168.7	4996.32
-1101.84	-2424.52	-6031.74	-6488.22	-5022.95	7355.48	-2933.1	-681.6	-9590.16	-1416.84	-3993.87	-5693.37	-7761.34	2928.24	2064.01
-5494.11	3563.15	-613.838	-11351.5	-2931.62	2589.23	-2546.77	-2813.39	-9314.15	5919.83	3240.97	-439.585	-8135.24	5296.59	-1063.17
-3865.63	-2693.03	2315.51	-3207.29	-768.338	-2919.56	2508.92	2545.83	-397.334	802.702	-1224.32	-5832.86	-4761.37	1723.29	14746.7
14831.8	12090.9	15650.2	7777.29	2020.01	16077.7	8375.32	17437	5341.68	9563.47	9971.11	7797.2	11455.7	12934.5	20084.5
-864.627	7896.23	-1228.29	-5565.36	3560.37	-110.529	-5437.16	-4083.7	-5325.24	-2862.7	-8597.57	4243.86	1425.19	-1125.87	-3040.14
-1267.25	876.049	1945.21	-10980.1	777.426	5212.53	-6593.5	3932.34	-14685.9	6292.51	-4773.49	3402.54	-1032.08	633.838	1360.82
1887.02	2181.34	2463.03	-5563.65	2504.83	3111.79	-103.145	-1234.25	-1194.66	6615.71	-989.404	1603.49	-7757.75	7183.03	117.109
3159.26	5032.22	-1101.17	-3608.72	-2640.98	-2372.9	-7794.82	-1370.31	2875.76	1243.7	7120.9	482.107	3123.54	5423.43	-295.098
-180.807	1039.19	-2365.18	-5280.3	-3099.28	5954.21	-4244.69	1601.56	-7656.87	9730.61	-1235.39	-2302.65	655.949	-3191.28	3824.63
4583.87	1345.87	-1649.54	806.172	-5709.87	4683.56	-2812.68	2956.45	-493.021	2784.31	6401.18	5435.13	-1161.89	-4609.66	2232.79
2709.87	-631.082	-5148.48	809.666	-3736.29	5924.37	-3035.38	-5023.95	-5864.69	1869.19	1964.23	809.651	-3435.91	1168.94	-893.878
3536.32	1063.09	-2822.74	-5913.65	5188.07	7118.61	-4700.42	-4006.36	-8204.47	2719.47	-6582.72	-79.9355	-1883.11	-1892.74	-4801.17
4204.85	2278.15	235.207	-6084.94	3700.36	-1501.7	1633.73	-3375.76	-2950.62	516.396	9004.96	-2025.32	3189.71	-1086.55	-4485.83
12539.2	8312.35	53.7539	5252.78	7512.14	10573.7	1642.21	6011.88	4438.91	10337	9396.78	1488.32	3397.58	10723.6	-1684.5

-1810.07	9923.52	1802.07	-4620.82	4217.22	10967	-1536.17	2157.62	-2657.26	4991.6	-2699.04	5700.06	-255.324	2406.56	9030.32
-860.883	-1466.89	5018.66	-3783.21	302.053	8061.35	2144.53	-1719.87	45197.3	9021.85	-506.934	3577.68	6865.83	5914.96	23266.4
-437.154	292.229	3063.26	-2736.17	-4875.39	55470.9	-2614.46	34848.6	-3453	11301.4	2333.53	5469.62	27532	11341	2461.75
5586.29	951.012	3121.52	1272.15	845.928	1555.65	6294.62	6863.63	-6620.88	30716.6	6846.35	-2631.98	23522.2	8331.16	-1028.41
1802.64	8402.56	7810.91	-5546.41	9944.76	-422.838	-3488.18	4022.46	-1227.04	3043.18	-6025.47	7808.99	1462.96	-777.656	-1997.14
32688.6	5216.08	4535.66	25630.6	3937.84	-4520.21	9286.88	837.041	-6119.16	5766.35	-3216.86	5951.9	2225.27	1035.02	-3309.79
3879.39	1005.26	1858.22	4848.52	-6997.41	-3333.62	42001.4	6131.32	-6942.08	547.934	107489	16246.4	5281.33	-3361.27	148.605
1408.88	43684.7	40294.6	-12444.2	-12284.8	5379.46	-3094.84	23186.3	-5944.74	-2173.65	-2326.48	48063.6	-100.773	2256.39	-1249.47
14896.6	48476.2	6564.07	-13769.6	5895.8	3397.17	15948.3	414453	-2685.6	8081.06	21591.4	27038.8	115988	6907.78	86893.1
-1411.35	-6229.66	-4768.85	-11755.6	1602.63	-4124	-2357.51	6498.18	-13601.3	1665.79	-462.486	3176.3	-126.471	821.09	6029.69
-572.508	5938.8	-2390.05	-441.729	-2405.92	3458.01	1454.21	9315.34	1709.68	2223.67	6784.26	5200.63	4446.89	19413	4485.81
10910.1	13144.9	9896.42	11507.9	6032.7	10256.5	9105.19	15889.1	7515.32	16577.8	9868	10284.3	11424.3	13092.6	8656.5
9731.27	12268.9	7945.35	1176.22	8964.44	12128.1	-1358.42	12325.1	1815.43	16283.8	-568.969	1990.78	16406.3	19649.2	13958
3056.97	5771.89	-2213.13	-4339.12	2799.48	-851.156	4240.39	3821.36	-10409	10420.4	259.051	-802.143	-893.633	14021.8	5247.88
-1020.46	10508.2	8601.61	-8663.49	-42.7578	6585.45	-932.822	7182.69	-5177.83	3511.14	6685.51	-1797.17	1273.04	7075.34	5784.4
1039.59	2682.94	1215.55	-4264.25	5562.29	6651.65	1122.63	-827.461	-6018.96	-481.149	2237.08	7986.49	-4200.85	3138.44	189.051
4612.22	-3904.92	4013.04	-3576.45	-1582.93	8266.42	269.061	-313.535	-1901.9	6833.8	-2931.29	1195.86	1484.17	8514.32	6241
6962.2	4972.48	4833.63	-2130.55	2292.07	20749.2	6624.21	15913.3	12551.1	5551.13	-45.168	5779.49	2698.88	4378.66	4755.84
3879.7	12475	3360.83	-2025.22	222.859	12941.8	2473.66	6661.67	7221.47	16246	2445.36	7861.93	5267.45	-4754.27	-225.834
5825.15	11199.6	4338.43	-2396.59	-2206.57	14119.9	-3657.7	15915.7	8322.24	18449.8	4665.85	3041.25	14357.3	7787.14	9297.95
706.295	5728.58	3977.91	-55.5625	3714.1	1435.54	-559.256	7773.96	-1812.41	2702.74	1240.92	374.677	5495.05	1182.19	4519.93
11704.6	5267.96	9145.14	-3718.56	24.9316	12902.2	3377.52	4714.03	2722.45	10492.4	3554.16	6286.37	2546.66	-372.148	-2577.56
3254.19	17462.4	6296.38	-12347.6	-11123.6	17252.9	-450.625	12239	8759.34	14335.7	-8578.18	9449.37	1881.8	5927.32	330.662
3051.22	6246.04	2954.37	-5333.21	-2521.64	14415.8	-3178.47	13301.4	2704.35	7595.93	-3037.6	6068.53	14359.6	9013.07	10007.7
8301.28	17610.9	3723.42	-2336.06	2533.17	3187.14	19152.2	341174	6311.83	7089.31	2507.21	23124.3	14635.1	10941.1	25916.2
19637.7	24017.8	9681.11	343.592	6080.49	19972.5	13501.8	118380	6108.92	8011.78	23523.6	23345.4	120168	11721.8	73962.1
5402.89	9194.63	3247.21	2265.72	5481.23	8945.86	-2255.68	24190.2	-3665.85	5242.12	-63.8965	8874.86	7726.34	13285.9	19298.3
13925.8	5874.59	7279.71	-540.561	3138.41	11069.3	-3912.73	26127.4	-1125.65	14458.6	922.127	11002.5	4695.71	13049.6	21927.7

18142.4	18435	5526.46	3569.23	-1061.11	24264.9	15465.2	226209	9866.32	17921.4	10393.1	18020.9	16609.8	16024.4	41631.1
40541.4	36067.2	21147.4	11015.4	9499.94	19224.5	48471	669203	28760.8	22220	64291.7	51587	202933	18437.8	65223.6
27768	30586.5	11349.6	2599.2	5415.73	14700	12881.2	245583	8254.66	1150.64	25528.3	22801.1	139005	15661.7	85188.2
11119.9	9231.47	2828.47	7920.27	4525.83	10858.8	2032.48	26430.6	-5543.76	6457.02	5725.06	7142.29	5310.29	-3311.51	3522.46
6597.82	13704	10811	4345.68	-3992.72	12239	-136.482	25156.7	15786.3	11969.6	-2107.51	12282.7	7318.16	8043.57	19650.3
12085.1	9881.17	525.229	-4051.79	3471.27	15169.4	-1038.14	17638.4	1060.16	19062.7	3608.74	4335.35	19957.7	9018.08	12322.1
6015.54	9152.89	1241.22	-5302.06	-2052.18	7178.48	-673.188	5054.77	-332.521	4784.58	-10739.5	3415.71	2911.3	3087.96	440.994
22805.2	11967.5	1631.91	3239.01	879.041	17836.7	4871.54	9693.22	-2408.37	11349	-2887.46	387.775	8485.88	8267.54	6764.62
13725.5	12314.1	12431.1	4147.09	6619.14	7225.6	9200.43	14006.5	10834.1	8677.27	7189.82	8820.42	9304.29	11576.3	16019.6
8176.48	17120.2	13056.5	2558.13	-3124.51	24947.8	-2637.72	24071.6	96.1055	14216.2	1467.95	7625.56	22951.3	17673.8	2860.06
436.307	2666.3	6632.82	962.168	-1367.36	10529.7	-1457.21	4819.78	7437.28	9874.3	-2944.12	6548.69	16502.1	-2859.56	4271.34
12232.2	9921.57	7104.09	7059.75	404.604	2484.98	5910.94	15059.9	-1864.16	16289.4	4236.95	11213.5	13021.1	6936.07	13681.7
8437.67	12773.1	11137.9	4321.58	-638.975	22449.5	-2478.49	11256.1	3072.97	15134.3	12721.2	7306.65	8362.02	9133.94	12188.1
2382.29	1896.5	9213.53	-2133.26	2526.89	19090.2	5815.77	73.7637	-6286.16	13148.6	-2425.28	7472.99	15892.1	9600.23	9971.96
9931.21	9305.65	7974.92	-4196.94	8809.02	7656.11	5322	6945.82	-3505.78	16516.1	1527.93	14920.6	1884.69	7783.51	11132.3
48343.2	41073.1	24189.3	37761.4	25064.6	32275.3	13912.6	19664.6	13332	33787.1	24917.6	29914.7	34918.4	28867.9	33854.3
44058.6	47102.9	29236.4	31942.1	13627.5	47799.6	27077.6	41800.5	34965.6	40092.9	38670.3	36431.6	54217.9	42454.9	28433
7902.44	20438.4	9896.3	2143.21	3028.67	10737.6	5713.91	16287.3	1546.11	11471.6	4881.65	13573.8	13344.1	7137.43	14863.5
20334.3	23900.4	20201.6	10651.7	30320.6	16917.9	22359.9	246614	16233.4	24706.1	28537.9	37676.3	76569.7	18397.2	30848.8
61680.2	65117.2	59966	92673.1	79689	59827.3	43669	129408	58392.6	79526.4	54384.6	54573.6	72842.9	80199.5	94386.2
13858.1	13185.8	12048.5	-855.223	4627.3	8824.25	9517.36	195042	5774.33	10210.1	16678.6	20351.7	96605.1	8356.71	30806.9
3298.71	3568.92	4410.32	673.979	-989.965	9454.41	-10325	10271.2	-5209.43	6833.47	-6091.18	8496.23	6304.95	-21.8691	13837.5
4049.7	12084.5	68.9355	-291.535	-4910.9	11100.4	-1589.42	2267.6	-7595.03	9596.53	7814.05	11357.4	5012.92	348.488	28359.8
10847.7	11878.9	3785.25	5193.9	7583.96	6565.68	-2849.58	14418.4	15686.8	21604.7	3255.34	21126.3	609.629	8414.32	16076
33060.1	44981.3	20655.4	12619.6	44859.5	39382.9	10995.5	30306.8	17373.4	36172.6	29893.6	43800.8	43605.8	34384.4	29289.7
456467	359115	241586	411147	274959	442205	328861	416709	436606	567882	419722	279055	377660	485513	431931
37154.5	153772	173001	15699.9	92642.5	37808.4	14691.2	23252.6	19747.8	24808.9	21008.3	44980.9	141743	23982.3	35853.3
32622	34349.2	22244.1	23605.4	41486.7	28797.9	12968	12753.8	13620.4	15223.7	21395.9	19598.2	24623	29021.4	22074.5

31658.4	20757.8	16101	14921.1	129702	5411.29	12989.4	5653.61	7945.7	13725.5	8763.89	15618.1	-215.334	-851.139	11752
248317	47879.7	30939.5	153346	39296.6	54815.9	122781	45489.5	44678.1	42325.1	66887.7	29609.7	56839.1	48465.9	22148.3
29102.4	41163.6	139096	8183.93	687.396	9147.32	21422.2	5167.07	17155.1	7399.4	48855.8	79603.1	7394.76	22091.8	15723.7
10204	77903.1	29898.1	2176.02	-3652.04	27618.1	3956.1	31812.3	65511.7	22072.5	-2469.35	20487.1	23061.6	17104.2	73152.6
11074.7	15076.7	3497.02	-2495.32	-6326.57	71790.7	12756.6	260813	17145.3	63368.2	8296.67	21859.3	33177.4	56636.4	47730.7
5700.76	13606.3	16056.3	-3684.15	-4079.68	19055.9	9441.09	60244.6	2357.55	16157.7	12665.6	5921.22	122396	23103.8	19828.3
7578.47	8742.24	3558.3	-3397.34	-952.584	10339	4616.23	11949.9	-2789.92	12908.7	2634.73	5601.09	7877.6	9839.13	17151.1
5279.09	5395.68	6859.93	4568.57	972.77	8341.6	-2242.46	11051.1	3640.7	9709.89	3073.08	9854.54	9864.73	7489.22	27354.3
12286.1	9520.42	9410.2	14799.4	10575.1	15002.2	6529.08	12836.8	9468.56	8220.76	9268.88	10583.2	17031.9	11450.2	37440.3
582.523	7613.69	9066.09	5399.35	-8067.68	4381.32	-7402.67	9309.09	-4217.44	8598.31	4319.38	1745.48	13069.2	6718.44	9224.81
-1269.72	1707.39	3910.95	-8815.46	-1854.1	9278.2	-7241.87	-205.617	-2257.96	5770.19	-8.98047	3846.42	9382.79	1855.92	9093.5
7588.46	4904.87	4249.81	-1039.77	-5415.55	6969.01	943.062	9714.4	-8490.87	10719.2	6268.62	5889.04	-596.381	10503.4	5116.14
7128.37	5763.18	1912.91	1091.3	-1455.64	9976.67	-992.873	3933.86	-615.523	10243.7	-1785.65	10295.8	2790.04	4752.2	7853.45
24711.4	12575.7	14063.5	9806.66	8219.2	13890.7	1295.01	7733.82	11990.7	20118.3	3853.84	7593.37	12343.5	14110	9475.59
1894.13	201.531	2854.46	102.51	-1277.23	6017.08	-8568.84	917.994	-2156.9	-5038.62	-5540.42	-2442.09	-2590.84	4462.53	6593.41
-2868.3	-2264.36	5610.11	-2450.62	-11201.9	1439.14	-6289.33	3847.22	-11871.4	-4115.31	-2860.62	-3543.97	1598.99	-709.598	-2895.5
-5858	-13339.2	-7011.69	-14867.1	-8549.98	-8985.38	-16897.8	-5331.88	-13497.4	-9038.11	-4967.94	-3273.99	-12535.6	-3318.5	-6847.53
3406.56	626.252	1885.88	158.65	-570.898	10812.7	871.223	12332.3	688.861	10492.4	-1487.28	4190.2	6866.05	1511.22	9438.12
13683.6	5822.13	1674.59	2413.3	-989.193	6511.15	24166.4	342535	8429.73	9208.67	8428.93	12975.6	9404.11	-2348	27883.4
9629.63	8480.02	6469.29	-1089.38	4444.84	5335.55	2147.39	57640	-3354.35	6747.76	12899.7	8978.84	106331	-661.279	14710.5
25724.2	21720.4	1882.03	5780.77	2271.23	21241.7	17089.8	29223.8	10024.4	16096.8	23580.5	11347.9	8445.19	21615.3	12407.5
4353.72	27716.8	20741	-7628.12	954.211	2987.68	4626.62	156714	-10560.1	3129.19	11078.2	10751.6	61903.8	6757.87	36049.8
2632.24	4680.92	-528.436	-5524.19	-1179.64	-474.533	1186.45	4275.92	4724.78	3193.04	-547.15	3555.49	1324.96	-1642.21	5157.42
6324.39	-1743.02	3732	-5425.06	-6545.96	9769.75	-56.832	3449.2	-8463.69	6074.37	-4164.8	6662	-382.66	-683.365	-1872.8
2179.01	5766.25	7500.3	-8932.89	3633.5	5825.11	-2977.04	9830.29	-2088.03	8898.95	-2901.24	-1618.75	12803.2	6082.42	3563.79
295.646	1330.35	-1317.71	-683.932	-4173.39	-1990.65	2981.74	5070.87	-4804.59	6950.61	-3619.66	-6766.7	4608.96	-1593.29	738.248
-2466.67	1295.03	-3438.51	-441.268	-7405.24	434.492	-2043.68	-2158.05	-225.422	3284.47	-1835.87	4567.3	9814.52	132.812	-2165.03
3341.6	160.314	9074.96	-4082.58	1438.2	9769.72	5208.21	10077.5	-739.84	1137.31	11035.9	-3245.65	6003.25	7696.86	8846.91

2053.68	8164.49	3597.32	-13013.8	-2247.33	-2921.55	-2805.46	-803.941	-10758.9	7875.92	-2870.35	-2839.71	6932.78	2988.71	4225.11
1195.14	3119.6	3384.91	-1202.79	2451.9	4628.37	-731.705	16851.6	-3833.67	10080.2	434.412	4046.74	11014.6	8043.59	-912.1
1618.74	3925.27	-496.406	1051.14	-23.1816	1747.9	7233.63	147253	2960.17	9490.5	3461.74	8549.21	13637.9	4677.86	27873
-4313.45	2222.08	-1474.56	2177.27	-3561.3	3981.79	1219.37	19208	2712.49	6195.13	-886.723	3662.68	27308.3	14359.7	6193.23
3755.27	7146.6	4241.35	-6044.92	-4648.76	4546.45	-4190.99	10796.9	4818.75	9714.11	4371.74	-3703.42	6823.96	5826.74	1263.92
8075.15	10786.4	9220.25	419.15	2406.21	10804.3	2964.72	21702.4	159.732	10457.5	8965.71	11741.9	12134.6	8226.41	6830.07
8526.01	10545.1	12001	-5450.61	2995.12	8212.89	13737	203549	6825.79	8152.63	690.129	15868.1	16613.5	8201.85	11442
39752.4	30483.6	22412.6	9967.6	5827.03	13867.7	48855.2	602995	30514.4	22808.2	66004	40500.9	232253	29420.5	52534.5
6447.45	9923.18	10670.2	-367.291	2563.5	10217.4	1294.87	39103.8	1253.58	10348.1	8482.47	8871.59	17430.2	13572.6	458.648
10944.4	3491.44	-842.023	-6590.87	-65.9102	5536.82	-3532.99	39963.4	3069.84	9932.83	5608.08	786.158	21947.6	8302.79	24155.6
794.074	4936.21	1659.68	-3338.83	-3236.49	-2617.43	-8328.35	1154.66	-5521.26	3702.8	-1466.37	1005.33	4388.8	-1608.07	5792.65
3938.04	7114.88	-3753.33	-5132.71	-7027.5	-968.357	2206.04	5939.66	-9898.3	5.34473	-3434.54	4421.75	-964.836	3687.88	-1383.14
-1274.34	4949.24	7076.4	-1450.29	-10931.4	6931.35	2700.69	7019.3	-6255.32	1691.24	-317.814	1170.43	-3488.94	4204.45	-2533.09
-2882.19	2178.09	5175.04	-3894.57	-3118.19	4754.98	-2205.78	-3005.41	-2012.79	1274.28	-1646.73	1585.22	-319.492	790.502	-350.738
4049.14	1603.05	1261.13	-1214.16	2202.95	865.807	-4244.9	456.693	-4407.13	3989.54	1413.04	-216.945	-1992.71	1896.67	2198
-5304.35	-2886.51	-3523.12	-8610.71	1820.94	-4279.75	-4100.18	-3922.86	-6800.28	-3905.7	4459.72	274.055	-3531.15	4013.37	700.426
1351.66	-390.459	-1495.59	-7285.54	59.041	1326.38	-5228.34	1740.75	-7647.93	4376.92	778.342	-951.35	2203.46	-263.099	1250.84
-5350.8	3591.4	618.736	533.773	-6756.94	-1331.92	-6826.38	1236.96	-6440.95	5360.55	-1709.04	-7263.41	652.172	5146.45	2580.65
9832.84	4361.11	754.25	-3810.61	-11588.4	2522.81	-1458.71	-1145.28	-569.576	-3396.5	1811.14	7313.57	1627.84	-3189.61	1465.66
2811.36	7573.72	-1219.87	-6138.91	-4619.92	238.221	-241.801	10916.2	-5231.96	1702.72	968.578	2074.29	-2045.44	907.219	3717.25
-3397.23	1736.49	3935.67	-7821.3	-4646.3	653.172	-3148.79	-1787.88	-5130.86	5122.32	-3697.05	-703.099	-271.395	-979.484	-128.291
1177.66	1054.89	-3488.82	-880.508	-3325.1	6701.69	-7252.43	-1959.69	-3395.51	3036.13	264.271	-3576.17	-4447.05	7562.73	-1835.52
-615.305	3754.48	-2597.65	-5202.67	-2940.32	829.115	752.687	974.768	-4682.84	2177.88	-9341.13	-590.461	1154.63	-7.6582	923.926
-3345.53	-155.516	-6740.14	-6622.65	-2014.44	2472	-25.3066	2271.74	-5447.41	4152.66	-1415.59	1303.52	4991.71	4997.84	4973.83
-6631.88	-2504.1	2291.07	-593.043	2977.81	5944.81	3241.89	2909.65	-2551.71	-4068.55	2674.52	-140.471	2284.81	272.242	-83.0059
-3439.46	11185.4	2602.55	192.012	-576.529	9271.87	662.121	-820.408	-10306.5	-3517.07	-138.449	-871.943	-6082.38	-7818.88	4724.7
-142.316	3113.46	6388.1	-3695.92	-2711.74	4442.31	-1647.97	-315.479	-1598.35	291.909	-957.195	4571.7	5093.72	2004.81	2515.2
7530.24	-8335.65	1242.46	-2195.36	-2752.78	3699.82	-281.941	3239.72	-6596.08	4234.09	-3112.43	289.634	1780.31	-689.702	1528.3

3229.97	-2186.02	2101.39	-8650.3	-3841.45	5050.29	3252.6	-2583.67	-4329.38	4448.93	1616.65	6296.26	-3480.57	-1863.64	280.025
-2274.1	-2589.18	-73.6133	-8477.82	-4300.33	4728.85	41.6855	-5578.42	-10980.2	-379.264	-1750.75	5703.92	-990.57	-2340.23	4000.42
2476.38	4509.28	-4.54492	-262.063	-4734.36	6073.41	-1466.32	-814.359	-7668.62	-2924.18	-4742.91	-2910.26	-2589.55	-991.271	-4132.88
815.613	-1304.66	2170.98	-7305.71	-4238.72	-1020.8	-2798.35	-1154.12	-643.855	-454.856	-4623.65	1451.71	-3322.79	-4928.93	-2534.71
-2782.39	-1529.28	-2493.18	-2779.19	-3194.45	2834.45	-6287.66	-4152.52	-8595.4	4000.22	-3773.84	6508.44	-6217.72	11011.9	-39.1016
-7688.17	934.854	-1077.84	454.424	-2901.03	4538.79	-6197.72	2621.06	1169.14	5876.47	-3282.98	5901.26	1072.06	-148.129	4429.39
-3725.28	1616.9	8260.01	-4208.54	-5406.48	1904.99	-8367.48	-803.16	-618.293	7595.08	2381.75	9087.6	-968.613	3774.77	2329.22
-3160.73	-2269.89	-2518.77	-761.6	-4192.15	6969.9	1904.56	4961.45	-5096.43	281.864	-842.35	-3771.26	-2669.38	-902.926	-5418.79
6811.71	749.541	-2913.19	-3982.89	-1694.69	54.0977	-1215.75	1472.61	-3539.52	9263.91	-4785.78	-1980.34	-566.24	-597.419	3818.28
2184.38	1421.32	5416.42	-7412.75	-8130.39	-3890.35	-1689.89	529.814	221.049	7201.17	491.836	4474.12	-5348.18	788.52	4648.37
-7855.52	7853.67	8497.62	-4619.89	-4975.22	2845.07	1181.11	-280.82	1846.86	7136.1	-82.4707	3769.7	-5366.06	2970.82	1242.19
1510.11	4860.58	2638.92	-7645.13	1880.34	-1599.21	-6637.09	-1156.74	-3354.08	-5830	-914.166	3689.78	-5143.34	250.52	-9206.58
6496.02	1569.31	-191.998	-4186.55	-680.545	5866.24	-163.23	-559.479	-6787.19	6885.5	-5449.37	1476.35	3658.62	5713.83	2238.98
-912.889	4514.2	4463.18	-3575.26	-9039.75	147.145	1355.69	1886.79	-3319.68	-1511.27	4567.32	-4127.27	-4465.49	1963.58	1438.41
4695.16	4216.65	-728.766	-1863.2	-7993.02	1350.76	-4695.96	2473.29	-5612.99	4647.17	4116.02	-321.671	-3964.51	5114.95	-3548.56
4324.31	1216.15	-984.451	-4043.64	5955.99	-601.607	323.709	-3498.78	-4005.71	632.389	-2518.03	-1728.61	-2758.32	-839.091	3965.95
1343.07	-3384.4	2369.2	-9549.32	5.86523	-588.99	-8084.76	-2110.71	-198.441	-1095.16	2472.04	1928.26	-1598.93	-555.541	4619.12
-1422.3	-11546.1	1975.5	-73.7285	-8568.01	1986.11	-7675.59	-5076.16	-7712.98	-777.831	2582.3	8516.79	1059.62	2551.15	-2211.37
-3104.36	198.494	-110.65	-2283.71	-5361.92	1788.45	1894.03	-1544.18	-5813.04	-4528.02	-4285.02	-3541.04	-6005.04	6735.78	1886.93
607.371	1661.55	1496.43	-11198.5	-10856.3	-924.012	3465.5	1086.17	115.996	1205.18	5289.52	5853.33	3653.66	467.994	-74.8145
-5291.39	2658.52	6578.65	-4068.5	-1503.02	1948	-1611.18	-3084.14	-6938.19	-489.864	-3321.88	-159.747	864.67	4397.1	-5087.55
148.68	1107.2	3107.85	-5361.86	-5089.7	4208.37	-4748.44	-1583.55	-2087.99	5555.12	-4596.23	1472.36	-4728.42	94.7168	-1209.08
16.1055	121.223	3586	-10021.1	-1079.53	1878.39	6356.03	2927.56	-216.813	-2014.93	-2001.12	3387.64	-783.664	-2292.96	3869.21
-4991.84	5978.71	-2542.22	-11962.7	-2426.92	9945.11	1691.66	-2936.27	-8518.41	3701.8	-12072.5	-2805.33	1807.01	2867.98	-800.768
-1946.21	9641.67	6982.37	627.297	-972.809	-1941.59	2575.39	6768.19	-6118.9	3978.32	-6340.18	3520.16	-4587.82	1764.99	4012.23
-675.738	726.031	5718.21	-7.26758	-3516.39	-4030.04	-3818.47	618.561	-6416.02	3444.93	-94	289.432	6573.47	-2575.23	-3836.62
-3144.59	1981.27	4357.79	-6616.65	-2743.9	4342.63	3468.57	8340.36	-5468.39	-2221.36	2321.34	1206	1338.43	11107.6	-3983.75
-2912.75	6232.94	-4523.05	-3222.09	2880.97	4526.95	7190.04	145861	-8122.38	2587.45	13488	8513.19	45837	9145.2	25387.6

43.0762	4319.92	2635.22	-11250.5	873.451	3299.18	2842.42	2712.01	-4158.03	5777.51	2066.41	2949.76	10372.6	6107.55	4480.43
-600.633	2144.08	5374.97	-2144.53	-4575.2	3726.39	617.135	-1627.92	-4768.47	960.756	-3177.36	-2224.93	1257.75	1828.41	4595.98
-4728.82	-6384.12	778.889	-5024.45	-8523.8	6476.71	-10049.8	-5226.32	-2866.32	-737.062	1006.08	3405	-4886.22	1502.61	-823.072
1868.44	-2833.51	-4312.19	-4756.04	-4878.48	8794.58	-5293.97	9741.2	2499.55	3649.99	-5434.62	-5356.67	-1113.65	2120.69	-291.881
4079.45	5722.45	690.746	-3987.04	7441.16	-130.025	-3879.08	40346.1	1226.24	131.123	721.857	2762.95	10643.7	1308.35	9656.05
-3394.17	5243.69	6232.44	-98.4844	145.311	9126.27	-5024.47	5341.64	-3070.65	-1278.28	-354.457	1383.98	2949.48	3006.34	3581.64
-3823.14	6410.21	-3163.95	-1855.58	3098.25	5620.49	-3298.96	1038.25	-4964.16	7604.35	-5331.96	-1501.17	-1225.17	4935.13	-48.6973
-765.547	12688.4	807.74	-5680.05	459.752	10245.5	-1152.56	-4146.43	-3205.49	9284.81	-5484.44	3378.29	-3077.27	-3418.11	7245.82
-2589.29	2091.86	1290.38	-12988.5	-3228.3	7220.05	-1228.76	-3745.08	-6229.43	4920.69	-7389.41	995.676	-2746.13	580.277	-2844.29
3376.5	5475.08	2822.51	-4512.75	-4700.09	-3088.37	-4482.15	4705.79	-5702.31	1167.15	2795.22	1512.13	-1218.92	-1273.75	-231.24
3921.15	1726.74	6902.26	-4058.12	-1500.42	979.125	-1619.76	-4374.66	-5836.49	-1419.17	964.117	1467.04	-845.609	2798.2	9105.4
1471.84	991.197	1462.69	-8074.71	-2144.78	569.195	598.232	1310.12	-3749.39	6817.3	2051.48	-937.349	1017.37	6395.17	2386.89
2702.82	5183.31	3370.17	-7684.49	-10292.9	5119.31	2132.25	1734.3	-3349.56	-6211.32	-1846.01	9727.27	-5857.56	-2790.78	4788.5
5748.05	7430.29	13289.4	-782.83	-2882.23	6936.67	69.3047	36826.2	-9537.42	5192.08	6371.28	1327.73	6661.93	1027.87	6006.41
3421.67	4937.21	2424.56	-2086.54	2977.89	1901.52	-350.363	3538.79	-6500.29	5697.26	-752.82	9560.57	3022.35	8128	9616.23
-3806.5	-2971.01	5586.11	-7269.98	-5177.03	1613.37	-8999.11	-3757.25	-12429.7	-1816.4	-8269.91	-1634.87	-4304.42	2302.26	-1871.61
-3252.54	555.785	-890.547	-2506.56	-3716.19	6283.6	-5025.58	832.092	-14215.3	431.904	-4306.23	1179.83	1330.51	-1285.02	-3447.13
-3319	131.732	3084.68	-11318	-7222.25	-1090.66	-2987.12	1833.86	-5026.6	962.823	-1159.82	-5247.21	1719.22	1170.06	2108.4
5322.57	1203.03	-570.998	-5016.94	-6426.01	155.492	-8616.24	-5097.13	-4175.02	-1906.55	-4412.41	-1012.36	-2478.65	2664.66	6415.38
448.98	-4689.79	119.762	-4207.12	-1575.71	8709.41	-3836.6	1022.11	-7137.57	3184.03	853.555	-1659.94	-1552.75	-3368.75	7746.28
-256.818	-2826.92	-4748.58	-5648.77	1568.03	2864.76	-1943.95	7056.48	-13040.3	1264.6	6777.73	923.801	-9845.63	1730.31	-15.959
-4239.31	1938.35	3464.55	-3901.82	2745.97	6897.87	-2467.36	631.102	-9727.38	-2728.39	1844.7	-2710.32	-2784.72	-1013.02	-5006.5
-6371.54	-6416.98	7220.05	-3661.11	-6677.52	-1102.31	2868.36	-1171.69	-2935.05	-3950.01	-742.023	916.314	-4284.54	4151.18	-2983.12
3436.96	-2841.19	-681.951	-3488.3	-15482.2	368.764	4798.89	2109.5	-7277.13	-2278.97	702.039	-2254.88	373.1	-3082.36	-1494.96
-3608.87	986.52	-3503.37	-2424.81	1980.59	6731.89	-3343.46	-470.895	-4847.99	3679.68	2258.95	-2843.63	1932.16	212.527	-1522.64
2902.63	176.746	-1410.42	398.744	-1469.44	6246.21	4430.25	-5447.41	-12623.1	7693.98	-8139.3	5941.69	-7700.5	-4309.88	7.83203
-6197.82	-270.088	1608.14	-5337.96	-7058.25	7235.11	-75.3496	13100.6	-9924.72	3219.07	-1956.42	-2466.56	-6050.91	-40.957	1716.01
3266.68	-5243.02	-5821.95	-3520.77	-4585.61	7620.33	-1793.81	-1160.59	-9979.31	-854.975	-10732.3	-2589.91	-385.4	1703.32	5728.54

-5936.98	-6814.18	-4422.11	-5700.13	-6010.2	6568.87	-1135.16	-2707.34	-13791.4	5321.04	1357.64	3709.96	288.975	-394.365	-5808.58
-3236.31	-2848.33	-279.982	-2909.54	1427.08	7755.79	-3143.6	1486.19	-14720.3	-1117.94	447.564	-454.893	-2922.57	749.615	500.066
6985.65	1473.42	7428.17	-6218.92	-4272.67	-1530.91	-6893.73	-3561.22	-7845.44	-4643.64	2216.63	-8263.13	-1919.32	1878.52	2248.5
8530.13	-983.908	642.74	-1720.52	-2205.65	512.342	-5788.58	-248.828	-9824.15	2266.62	2436.77	-2203.97	-2788.23	2937.21	-2892.82
2598.78	-5436.11	-4313.94	-280.129	-4371.93	2162.77	-2441.1	-3090.77	-8622.94	5823.81	-9956.16	4378.48	-1712.63	-5315.14	-5442.26
-3037.65	527.719	-4294.95	-4914.78	-5518.95	2657.65	382.75	685.391	-7844.98	5665.5	-6853.7	-1193.85	1721.55	-5675.98	-365.932
-2241.63	5216.47	-1355.74	-6706.35	-5380.12	-7886.99	402.871	215.656	-3070.95	1536.1	355.018	5209.94	-3620.79	3991.93	-673.08
-608.375	-6289.96	-2762.26	-10756.8	-6941.74	-1635.35	-2556.71	308.951	-8997.11	-1855.66	4072.36	2996.38	-7360.41	-5082.39	672.174
-8195.56	3286.14	-35.4629	21.875	-7950.43	-2115.7	-4786.42	-3967.79	-6958.64	838.301	-4318.8	1402.74	-2455.44	3688.41	-661.428
-3059.97	4578.41	-2676.3	-6259.07	-10956.8	306.641	-5112.24	-2274.12	715.018	1223.43	1764.79	-3949.39	-1297.15	4885.74	-1712.21
-6224.16	4657.59	-15.752	-8959.53	-4540.13	7613.37	-768.428	-2883.07	593.701	-8502.25	4039.9	1186.44	-1926.8	-687.642	-2462.01
-2148.89	57.1641	-763.299	-6876.16	-2574.23	5159.25	-7295.85	6390.83	-13912.5	-139.524	-3063.88	827.778	-4547.17	-2721.49	6658.21
-25.9746	-1608.55	3354.63	-5521.55	1138.09	3403.41	-378.715	-2537.72	-2684.21	1885.4	874.588	663.239	5199.65	2535.61	-4883.65
968.105	2220.1	507.742	-8895.54	-830.863	335.096	-629.666	-2962	-6091.36	1771.19	-4136.04	4277.59	3255.4	-4243.57	-1915.82
-3134.45	-1614.66	1143.62	-1096.3	-11203.4	4230.17	-1240.37	329.465	-439.256	4250.04	-2082.57	-2058.48	-4582.23	-4882.54	3427.7
-1127.31	-7003.2	-784.957	-5638.09	-3750.54	4163.34	-5252.33	606.039	-3799.89	-612.171	178.01	1009.88	4235.32	-2151.52	-734.828
401.219	-4866.45	-740.488	2430.35	-3000.21	2495.13	-3618.68	-6203.25	-7460.85	1714.32	-3167.68	-5321.5	-476.895	-49.5625	1556.07
6182.83	364.457	4359.85	-7361.85	-10111.5	4268.17	-4982.58	702.439	-3481.14	2195.71	-3461	6400.31	864.707	3643.06	4965.36
-3656.29	-10493.9	5846.37	-5225.45	-7236.17	-398.709	2236.63	-4026.89	-8037.94	-977.835	-1827.44	4921	598.225	-620.809	-6463.11
4909.27	202.443	2170.78	-7047.03	-8477.33	909.236	-1436.95	-6537.8	-4064.1	205.607	269.771	3925.67	-6017.77	2090.37	-1214.6
-2791.85	-1771.99	3198.81	-4316.44	-7465.12	-2508.82	-2403.3	3002.57	-6183.31	1991.57	-4542.21	-1203.77	-12034.9	2259.94	-1520.04
-38.8984	-9255.8	4991.04	-1841.64	-9107.87	7854.57	-6193.57	-399.156	-11865.8	497.674	-423.842	4020.09	-9375.57	3856.73	1223.53
-2542.81	-5037.54	1246.35	-86.3535	-687.293	8783.18	-5752.8	-1568.48	-8772.39	2577.88	257.582	-4599.11	1188.29	2059.02	1344.06
-4360.78	-1458.69	-3837.14	-1448.12	-3839.74	-253.523	-6210.48	-1671.21	-6600.89	4727.23	-49.7559	851.97	-7087.6	-5242.6	-750.699
-1201.76	3055.33	4556.84	-3156.79	-9865.44	2862.18	-1102.13	-1930.84	-11625.3	3293.85	679.74	-853.299	-5380.91	1754.48	2136.54
2151.38	-1312.1	2599.14	-11914.7	-2022.18	3155.52	-2263.28	4903.56	-7463.92	4647.87	1694.83	-434.038	-1836.75	-4263.76	-6037.87
-4179.5	-6086.03	953.625	-6026.36	-8675.36	3135.92	1221.14	1451.17	-6225.86	-2027.63	601.867	-2672.31	-343.078	1490.8	-5314.24
-8442.92	-6639.38	2315.99	-3291.55	-6009	5428.24	549.41	175.711	-10039.2	1053.82	-2138.65	-2017.72	423.42	3385.06	1066.51

-1323.97	-8368.73	4910.25	-8205.19	-11147.3	-260.541	-4788.27	-5855.5	-9843.61	-1104.61	-1282.7	-2046.53	453.041	-394.782	7917.06
2229.71	406.07	4230.7	-3555.91	-1824.54	329.105	-10100.8	8072.63	-1924.26	675.041	4815.66	-4672.63	-2055.54	1035.8	-8730.19
4092.5	2346	-1229.88	-6384.37	-6105.11	-559.369	212.705	-1186.79	-2631.44	2530.17	917.996	-2061.73	-64.5605	-1243.01	594.283
776.531	-5389.09	2675.4	-12123.8	-9219.75	-4103.06	-2547.89	-700.23	-937.941	4240.92	-444.773	-5810.25	-5219.51	9468.12	-542.311
599.66	-281.295	-837.93	-7963.22	-7005.2	3094.7	-8556.15	4163.48	-10828.6	2182.01	-6722.61	2299.47	1418.52	-2739.07	4692.6
6227.94	-5527.14	-1164.43	-5561.11	-4944.22	-186.74	-792.316	1342.09	-3806.42	931.804	-2542.13	-2194.84	161.527	-1633.24	-416.137
1431.88	-1735.64	-214.172	-7078.28	-941.262	1423.97	-1097.39	-996.82	-2546.7	4694.1	-3128.42	-2299.9	3882.19	-856.197	3114.71
-1992.69	-8248.96	457.115	-1971.23	-5227.87	2178.38	-1741.16	-2415.9	-6830.59	842.954	-29.0508	5992.1	9333.5	-3175.53	-8960.69
-5483.59	2117.29	-4090.09	-5430.33	-4863.36	-4474.44	-90.9395	4745.6	-1908.63	2054.5	-3232.35	-1673.31	-4624.78	-7770.04	-4131.85
-5008.39	389.76	-1026.34	-2612.38	-4277.85	4294.32	-1437.13	2978.83	-3508.08	251.251	-2265.33	-281.472	-245.549	-665.399	-6067.99
1327.22	-8153.1	4772.91	-13505.9	2591.41	1127.97	-3438.49	-3983.64	-4083.72	9382.45	-2526.34	509.681	3222.89	279.412	538.023
-808.408	-656.414	5778.93	-9601.44	-5815.63	8491.56	-8048.63	-6055.33	-8323.35	5561.14	-2684.55	-2603.54	-6308.06	-7058.69	-4809.25
-8763.54	-505.031	-2270.24	-15749.9	3158.47	10651.2	-5668.06	3712.13	-1019.54	-4815.43	2426.22	-1739.23	2336.64	2598.14	-4267
-2258.24	-429.945	-4851.7	-6382.36	952.422	-503.543	-1443.02	4893.44	-12043.4	5248.95	1514.02	-8811.06	-520.437	-1966.07	-3397.25
-5244.73	6328.77	2230.15	-7103.86	-1989.68	-1633.83	-3379.24	-4798.56	-7865.63	4850.27	2213.81	-556.394	867.93	-1867.29	-1443.08
-3664.69	1346.63	2124.04	-7504.66	-6468.29	-758.137	-4275.04	-8672.62	-4752.3	-3248.3	-1854.12	4167.52	-5555.08	-5572.98	723.838
2968.62	1233.31	-4045.26	-10266.5	-9522.95	-111.75	1048.94	-3434.99	-2408.6	-2283.92	-3162.46	4104.57	-2593.8	2435.56	-3400.57
2711.78	3384.34	-2202.4	-4703.43	-9300.91	4578.45	484.58	217.74	-9227.87	-3095.79	1720.42	-1246.11	-4858.75	3206.5	-2134.67
-296.588	-5647.7	-3252.89	4512.89	-3521.64	6231.49	-3375.4	2504.03	-6750.54	5709.67	-1568.09	6194	-4547.5	-856.771	-3476.81
-2162.47	3074.13	22.4395	1.30273	-6537.87	6158.49	-4117.69	-4146.39	-8883.45	-1232.97	-1581.45	-4279.82	1507.61	1489.76	-1990.76
-1236.97	-2873.6	-983.18	-1504.99	-1299.22	-521.764	-7904.33	-3691.42	-2278.94	2146.45	196.941	4767.78	-1828.35	-2347.41	-6853.04
21.5996	-2868.38	9391.56	-9109.62	-2027.06	7235.67	-1071.72	-6281.58	-7474.7	1798.77	-3086.03	-2218.96	430.639	-8105.69	2274.21
-3013.27	-4708.83	10366.9	-3806.78	-6548.08	-1177	1749.3	-5538.56	-6605.77	4690.78	-8117.18	4081.19	-935.029	54.377	-5833.24
-49.8535	3494.23	-1008.29	-4989.61	-3384.04	-3060.03	-7147.71	378.445	-12157.2	-2282.07	-6050.89	-6193.57	1396.53	-1011.53	-4529.73
361.543	-1530.19	-3144.83	-3568.5	-3023.03	3375.78	-7901.43	297.672	-7398.31	-2448.84	5382.04	388.881	-6051.28	1585.79	-2788.44
-3002.44	-799.963	-6625.97	-3910.89	-5084.7	5313.01	-4932.71	-4884.28	-5899.42	-285.06	-348.4	439.112	-9805.53	2320.64	169.586
-3584.68	-4056.88	995.393	-4856.99	-3845.57	4341.33	1327.47	-2549.28	-10145.1	1174.61	-4140.04	2804.79	-1126.72	-3867.22	-2050.24
5059.95	-4317.58	4503.75	-8616.68	845.848	4751.51	-5380.74	3271.53	-1518.2	4633.5	-4757.02	4144.39	-5103.76	3805.02	1101.01

-3611.96	1998.22	-2284.58	-10166.4	1526.4	10696.2	-2922.53	-3527.67	33.4687	2413.41	-3068.92	-1465.05	-4507.47	-1907.06	-4067.1
-862.193	-466.668	2676.63	-9865.8	-503.23	-2740.23	6266.06	-1888.23	-8471.21	-3065.51	302.613	3944.16	-7122.66	1809.87	-1233.17
-2543.21	5769.4	4117.96	-4608.09	-425.641	1142.67	997.221	-1780.13	-8449.74	-4434.11	-6595.62	501.992	-5228.1	429.402	-3225.9
-2869.24	-300.947	3660.95	-9134.38	-4381.99	512.729	3009.25	-2061.31	-7794.85	-5311.34	-3455.45	2803.91	4070.92	-3417.72	-89.5762
4369.26	1165.18	-3233.33	-4464.03	-1108.86	1159.95	889.916	-1783.74	-9818.31	1742.64	-1781.54	-1690.17	-355.631	887.348	-68.0547
5316.39	-3555.62	4917.33	-9202.22	-3543.86	2100.98	-5687.45	-5629.99	-9551.37	-987.585	-167.719	2899.73	-1957.75	2292.71	-3356.77
-3580.15	-5423.57	-1679.03	-7260.09	-12820.7	2979.44	-4829.17	-1846.58	-14644.3	-927.243	-5024.07	3030.83	-627.557	-1677.23	-813.746
-2055.81	-2808.27	1525.8	-3037.15	-1031.84	758.781	-8007.73	925.506	-9040.22	-8669.16	-4548.62	2831.05	-3659.74	1947.29	915.33
-6595.17	-2033.18	1468.54	-2886.2	-5017.6	2317.69	-4620.81	-1292.69	-13727.1	-922.923	1027.17	-1027.42	-777.887	6028.68	-2765.1
-1395.74	-9592.01	-1620.81	-12153.4	2245.88	4695.99	-519.359	-4226	-9678.92	-2021.91	-7134.47	-2264.12	4845.93	-1711.99	-7084.33
-4875.81	2608.83	-2269.28	-6960.59	-1502.58	-1191.86	-3310.78	-3689.41	-6861.37	3405.74	-2696.69	-4539.97	-2090.24	1474.14	-4383.36
1887.58	-3367.97	2975.63	-5913.68	-5227.11	6245.69	-1910.22	666.721	-585.805	1995.34	-1080.76	351.973	-5021.71	9422.39	-2884.52
-1073.43	-6816.76	-5221.56	-7528.07	-6153.81	-1985.27	-428.898	-7672.73	-5661.48	5643.12	-1563.38	2778.43	4237.91	-2093.79	-1816.95
91.7734	-4236.18	-2845.08	-2862.47	594.502	-2242.58	-4389.11	-8.71289	-6691.1	-6571.19	-4287.96	-853.456	-6369.79	3033.98	-3495.38
-2758.79	311.637	781.471	-5392.98	-6072.67	1983.42	7127.44	719.666	-5911.56	3581.52	-3909.29	5531.06	3569.57	2109.48	2579.41
1132.24	5391.73	780.598	-7763.45	-6826.58	-2637.09	1836.59	-3841.82	-4307.65	5246.11	425.67	-3613.46	3533.54	-4311.81	3573.26
-3252.7	4512.31	-4633.41	-390.895	-5320.42	12288	-5011.74	-602.029	-6944.95	517.431	-2763.26	-2454.59	-47.7129	-3409.95	-739.264
274.045	-3990.27	2465.21	-9614.79	-9737.4	736.207	1149.48	-6181.98	-8689.11	77.208	3455.18	-3513.38	-237.291	-3321.24	2728.24
-3843.81	-2569.65	3656.22	-4618.08	-6686.32	-944.867	956	-4134.23	-7904.15	2852.45	707.285	-1050.64	2821.33	211.537	2537.64
1238.19	3068.45	149.805	-3118.12	-2136.56	2326.7	-749.072	396.535	-7359.46	2766.85	-5010.86	-7070.26	-3908.9	-1064	-1160.91
6673.14	2801.86	-3781.82	-8883.6	-2709.69	-1880.29	-5344.19	-1219.39	-5309.17	-3625.01	4797.8	-1946	-3897.86	-1754.65	1687.38
-2887.75	-3086.1	-5378.73	-5834.56	-7919.25	7651.37	-21.4355	-5621.94	3136.77	2793.66	-1401.68	-2368.57	4893.81	1425.14	-12388.8
-1160.71	-2650.81	3632.81	-8328.95	-9193.54	911.65	-6575.29	1817.44	1197.87	-3912.9	-1729.64	-1383.17	-2404.67	-151.102	-5353.61
-2946.09	1185.44	454.73	-6417.97	-6263.27	2127.63	-4816.31	1284.58	-3156.54	-1353.36	-4354.58	2800.37	-1295.01	2112.92	-6018.57
3115.46	-0.912109	735.293	-5932.74	-7102.97	-4222.29	-3859.97	-437.318	-6752.68	-4525.58	6128.49	-1058.58	502.361	7194.19	1647.77
-4589.5	2217	436.729	-9733.83	-2885.17	-896	-1177.39	-3871.85	-7856.06	5351.09	-932.361	290.152	4890.22	393.99	-5974.26
474.92	628.713	1867.88	-4131.86	-4508.55	-373.887	-6510.29	-1690.28	-12303.5	471.159	2528.35	-1049.76	-3371.63	470.158	-716.283
-393.67	-2338.02	-199.031	-11888.7	-2236.04	-866.881	-413.205	3629.98	-8261.04	-3205.69	2399.62	1242.15	-2458.77	5523.95	-1455.67

4484.61	749.42	1735.63	3437.89	-3802.01	3004.32	3059.78	7446.48	-5454.27	1950.4	-1217.6	5876.67	1568.96	879.031	-0.818359
8546.11	6717.65	3781.64	-8500.8	-3787.15	3223.3	12867.1	236870	1648.23	4260.86	11714.6	18393.5	61950.7	-1110.36	12327.2
-4468.6	3971.68	4277.99	-4015.92	-3212.48	4723.61	6005.34	5246.18	-1778.24	6483.76	-2744	1534.18	4309.52	3894.8	344.369
7578.13	6595.09	8480.29	105.607	240.869	3490.44	1237.95	13271.7	6268.66	6934.8	2870.83	1875.37	9053.54	10288.6	2418.17
-2244.88	-1176.9	-796.402	-1005.95	-4299.14	4766.68	-2285.43	6928.23	-8876.62	796.203	-3158.48	-67.332	720.414	3690.28	-379.205
1686.17	4885.46	-984.531	-11354.8	-3670.68	8028.19	-1464.02	4580.61	-7130.28	4701.71	-3868.64	449.123	1806.71	-4287.04	-3905.7
-1662.64	-716.646	2413.39	-8165.27	-976.102	11001.7	-2239.95	4798.54	-2087.43	1066.11	-4217.31	-4660.48	2670.29	1203.1	-2008.74
416.453	-1301.32	-4287.08	-6071.13	-7420.87	1640.29	7762.48	553.834	-8545.4	8537.42	-3708.1	-345.637	-620.744	1174.45	-2388.34
1840.41	2507.87	2204.56	-8729.29	-7059.57	6566.62	-5639.22	-6149.4	-6579.25	-1811.97	-3861.97	-6078.31	4707.89	-4847.49	-4574.64
-2667.83	1590.36	10584.7	-7254.35	-12285.7	-1151.29	-736.867	3227.66	-7220.96	5544.49	-4286.99	460.508	-2742.43	5341.6	-4367.41
-428.693	301.219	-276.404	301.725	-7303.76	3840.24	-187.398	-2441.04	-5228.59	1204.08	-3139.12	-1860.03	-8546.46	7423.06	3413.99
14281.2	5443.55	4442.2	-1949.77	-6089.21	-2329.83	3794.08	3880.23	-352.938	3766.54	-2872.58	6067.97	-751.746	13258.8	1471.64
2614.82	5331.26	-4911.51	-2831.28	-5098.08	-1692.9	-4357.51	-2460.33	-3289.27	438.256	-3155.84	242.491	-761.965	1569.31	6241.51
-1254.52	1707.63	4491.71	-9036.63	-5621.89	-971.824	-4491.08	4485.89	-2908.54	25.1387	787.135	2036.43	-3414.88	6366.15	-1258.5
-5885.72	4386.84	1280.64	-7643.91	493.699	5434.12	-260.877	2778.1	-7569	1781.41	-1014.7	-2050.81	1463.92	1036.4	3611.05
3276.17	3932.08	3123.74	-3950.52	-5759.02	-1661.7	-3947.15	-7500.65	-9304.59	3142.26	2379.57	-2233.34	1868.59	5556.62	3261.59
2102.41	2218.52	1228.79	-6906.2	-2915.46	-2005.35	2803.92	-3179.85	-2903.16	3192.19	263.207	1592.96	-233.553	213.205	-752.699
-3968.79	-1866.7	5699.88	-7633.72	-2931.44	-3410.5	2805.94	-4441.89	-1182.78	3035.83	1345.83	-7125.67	-1461.52	5936.42	-768.055
-1009.06	-2326.26	2348.58	-6236.29	493.637	6991.22	-6191.75	712.896	-3802.1	-1290.18	1648.35	-7228.8	-1779.03	-264.585	-47.5625
607.84	2674.22	-1478.54	-4557.55	4860.72	-387.672	-2612.26	-4353.35	-4060.37	2240.06	3134.79	-3408.92	-5309.74	-2422.17	-3667.22
4432.48	-3303.79	-735.957	-8419.14	1505.04	6736.21	-2133.24	-288.393	-6961.31	440.583	11.2402	-2698.69	-1915.84	-2940.92	348.561
1340.67	-6080.47	-710.404	-2900.35	-530.895	3711.39	-1250.07	-1453.21	-7492.58	-438.017	2905.58	-2163.29	-6670.52	5052.88	3729.52
-5002.89	443.006	-603.822	-5663.54	-2140.18	2166.49	-5461.75	-1262.52	-6081.56	83.6699	545.787	2587.38	2262.63	-2208.71	-2669.24
3482.13	-5829.39	34.3613	-10416	-11446.5	-2188.55	-6695.05	-2833.6	-7430.31	-3884.39	1555.86	-1469.54	3500.15	1883.69	90.9121
1771.64	-3474.8	-6381.77	-7446.21	-6417.11	2658.34	-5529.65	-578.531	-3176.4	551.516	-1954.04	587.012	-3584.69	-5764.95	-678.92
3083.25	5021.14	-2678.21	-7154.4	-2253.04	2144.52	710.107	2514.9	-8833.97	-2361.25	-638.074	-416.521	1287.93	-3061.08	-324.783
-2636.86	-981.965	723.293	-8410.18	-2922.45	9959.31	-5022.1	-2708	-4182.87	-4181.52	1074.45	-372.285	229.508	7134.12	690.016
-2901.76	-419.072	5979.29	-8523.48	-1348.96	10814.6	-6274.82	1770.5	-4386.32	4168.28	601.283	-2082.08	1174.74	1457.24	-2791.68

-3570.48	1178.27	398.299	-7877.61	-2409.7	-1791.13	2490.16	2734.56	-8899.78	1200.31	-456.582	2804.77	1691.48	5536.49	-2302.39
3005.55	340.129	1650.43	-11811.5	-2389.37	-3182.86	-4257.53	-8804.54	-8208.07	2047.19	-6127.95	-1059.7	1535.19	-436.321	-1501.36
1212.75	2263.99	2509.36	-6666.46	-5951.41	2209.28	1751.99	1175.24	2261.77	3897.37	-5408.9	4725.1	3287.83	-1342.54	1664.1
-2407.99	-655.596	5175.99	-5552.8	-4849.72	4160.59	638.756	-2669.92	-3781.55	-1141.58	-1036.89	2339.02	-3394.78	-2921.31	-6102.22
-3371.6	1695.81	-1477.54	-14080.6	-5398.78	4837.98	-5122.22	-3537.74	-2755.88	1770.04	-1910.02	850.933	2783.89	417.955	-900.366
83.1895	-114.268	859.879	-13264.3	-4557.37	-42.082	-5372.12	-2637.94	-6924.2	7200.1	-695.473	-1240.05	6498.23	3589.42	-1145.77
735.201	-2599.17	4361.68	-10562.7	-7470.47	4564.37	-3280.3	4036.35	-6033.83	3163.35	1464.35	6793.67	-387.066	-1198.44	-4147.4
1446.55	3836.85	-739.494	-8778.01	-8887.66	1039.86	-4851.6	2260.28	-7752.23	-3133.75	-2895.92	7837.56	-1105.06	-2714.14	-3195.99
3377.78	1347.3	6277.23	-11285.1	-4498.81	-105.98	-5132.3	2401.64	-5817.95	2034.85	4878.13	957.078	-400.877	1649.99	-10789
2596	5746.75	6724.46	-3883.07	-6607.19	2737.88	7077.71	446.037	-9151.15	5270.22	-7143.27	-1098.3	-2520.42	9345.52	-2104.96
-478.047	-7239.54	9870.95	-10745.6	-4846.69	3723.06	-331.637	5107.79	-8287.63	1548.47	3795.66	-1204.81	1061.91	6476.58	-2996.5
10248	3150.25	6716.67	-9756.22	-4348.29	4521.79	13942.9	313669	1072.93	871.685	11817.5	16389.8	8915.13	-652.622	8801.44
12674.1	3625.6	11781.6	-4330.38	-8476.72	4840.92	3990.04	83342.6	-2048.41	8127.52	22500.6	14944.8	115448	6398.98	17975.6
-2040.92	901.957	2840.28	-3493.47	-8153.56	-196.143	-6031.41	16214.7	-1293.47	14701.5	-4607.46	9485.04	3373.85	8348.27	5326.53
10752.1	5411.66	5696.27	-1917.33	-760.35	12951.9	4922.03	4473.95	6832.22	2108.79	-4018.07	7711.05	13037.8	7592.77	6497.97
-4546.58	1599.56	-3671.07	-3382.01	-3422.16	3769.46	-3124.49	-2680.17	-2297.41	2244.35	-2884.26	463.191	1361.43	1297.71	679.986
-2213.32	-1727.71	3564.82	-11635.1	-1152.4	2816.48	1060.41	4627.86	-7523.62	95.6719	1620.15	198.257	537.686	-6117.34	-4442
5429.83	276.828	3537.2	-2984.34	-2706.21	7755.2	3736.37	2379.8	-1863.7	767.842	6573.25	4404.63	-304.496	-5196.76	-3370.24
-646.52	6846.59	3880.07	-6496.16	2239.28	2779.46	-5068.2	419.393	-5165.5	-4219.48	1632.74	1157.4	-2576.37	3114.6	-4976.08
-3976.47	1581.12	1038.91	-8517.91	-4375.1	4315.57	-3097.28	922.1	-10526.9	-90.2627	3102.6	6806.97	2579.43	-6767.44	2066.5
1052.67	-1281.06	4916.85	-7200.62	-2551.64	11875.5	2178.18	4375.91	-678.49	-5165.58	5305.18	5218.04	2604.94	2976.32	1073.95
-5295.58	-1141.61	-2993.86	-5878.28	-9544.43	7047.69	-6727.17	111.225	-8581.81	9507.71	-1705.49	1692.38	2536.85	-277.728	-4071.67
7792.34	-3096.76	-3393.05	-13896.2	-9088.29	4479.05	-856.797	-3404.95	-8734.29	1534.47	6221.51	-8744.23	3837.43	-1758.92	544.971
-261.594	-2949.6	-2226.52	-1012.21	-1142.38	5387.61	-3766.68	-1890.47	-5557.35	2537.96	2727.81	2782.99	-1878	6782.27	-6847.07
5167.43	261.611	332.689	-7276.64	-7688.5	676.164	-345.271	2676.49	-5621.17	999.826	-4412.66	-1469.45	-5066.58	1604.23	-2831.8
1951.76	680.227	489.701	-10715.6	-6701.28	-4787.81	1206.81	-104.01	-6341.14	80.4941	-4144.65	-6798.99	1340.47	3722.15	-1679.8
5382.54	-5342.74	829.16	-7788.47	-3536.07	-65.4902	244.998	2138.76	-12762.7	3533.15	-1149.21	-958.835	85.2871	2824.09	-1105.92
-4389.65	-61.7812	1487.41	-9932.85	-4547.05	4624.82	-708.514	-907.293	78.7559	2777.81	3351	525.665	-202.967	-2003.31	6164.81

2541.34	2234.1	3225.04	-8337.85	2680.79	3518.31	-3298.92	830.289	-4356.66	-1343.28	2010.45	-3107.62	5113.19	202.697	-3872.83
2189	63.4512	667.18	-7806.14	-3706.84	-507.674	-2058.78	100.648	-4159.98	4966.41	3914.36	-4060.86	1859.66	-831.242	3220.45
3246.36	-1334.91	3448.58	-7007.7	-4316.54	3952.48	-1506.39	1212.16	-2769.74	2930.77	4015.75	3151.84	-648.959	6302.16	5312.63
1959.44	-682.324	1138.69	-6828.82	-7046.97	-5520.39	-3732.16	14074.7	-6645.65	4378.44	-963.023	-1838.1	7531.13	-1608.41	14199.7
6928.8	4547.97	-19.8848	-5994.09	1602.59	1954.74	5050.99	154885	-5367.98	5714.87	4240.07	3923.19	8897.73	-3040.43	11980.4
13922.4	8572.99	5892.38	-5249.45	3249.79	8414.35	4275.41	142269	-3171.78	604.729	6828.54	15100.4	86168.4	6545.37	53296.4
202.652	-1873.14	753.137	-5672.21	-1882.09	424.48	-260.18	8231.9	-2687.41	1759.85	2948.88	3001.28	72.4395	4607.43	1614.33
3371.84	-312.488	-4625.78	-7824.63	-3968.15	375.67	-5768.67	2145.57	-7241.77	-4053.01	668.564	-592.467	115.316	818.873	2368.99
1023.95	-1172.35	-2617.21	-6774.17	-4029.45	-4184.49	-9133.2	-4149.89	-6105.74	-3145.64	-1405.58	1063.47	5799.53	2559.39	5043.74
3387.79	-3138.01	2930.81	-11521.4	-8321.79	5197.58	-5625.83	-2772.94	-7819.12	4579.77	-6085.43	-3913.59	-579.682	-2380.89	6992.39
-508.014	-1672.43	2626.28	-3663.78	-311.605	5452.01	1331.14	2544.21	26.1133	3452.83	-6296.34	-5217.59	-153.795	-754.188	13088.1
1329.18	-4349.75	-1362.66	-4295.44	-3099.95	-1376.15	-2939.31	-3160.02	27.8945	6421.09	806.572	4214.93	5279.56	-975.886	-1026.97
-4013.9	-5235.95	2970.19	-14016.3	-2068.38	-3052.87	-890.729	-3056.57	-1257.23	3313.98	-2509.67	3521.13	309.895	-2238.75	-3485.14
-2312.18	5178.54	7822.91	-5280.5	-4797.07	4266.62	-674.303	5712.89	-5877.06	3184.77	964.066	1250.13	4346.18	4190.68	-3929.14
2889.3	646.725	3851.9	-9509.33	-6445.06	-4392.85	-4915.8	4907.42	-10613.3	2092.46	-3284.35	-2008.74	1972.3	633.029	-6206.09
3890.5	1335.03	2776.18	-12604.9	-538.6	2114.29	-5198.03	3010.24	-8476.2	-233.729	-2726.59	-7010.24	3472.71	1921.37	-5115.26
664.715	3552.07	943.133	-8155.61	-6389.1	5374.32	-172.193	-3057.39	-12907.8	-5285.23	38.1523	-4231.8	5466.77	-1501.24	331.635
-5150.11	-7571.83	-1245.74	-9163.22	-9107.56	-1166.38	4088.83	-5627.73	-4774.34	6782.47	5642.06	-8965.36	-187.342	-365.316	-5240.3
-1294.1	-5924.4	-7041.21	-2330.89	1061.11	-5640.42	-3103.46	-3824.72	-10134.6	-540.124	3128.65	2378.65	-4181.42	-2272.25	-2884.66
-8625.66	-2405.75	131.803	-11956.6	-9788.18	1977.95	2181.6	833.875	-4704.15	1468.41	-5317.97	3021.59	-4331.71	-1188.61	-1773.69
-1478.43	2094.02	627.797	-6772.78	-8123.66	-4105.78	-5726.47	-238.832	-10054.8	-2647.07	-4868.47	-5059.74	1230.57	6378.04	513.881
-2602.37	-1752.74	511.121	-12520.5	-4020.33	851.994	-2636.64	3882.01	-9984.62	-366.191	5715.17	-2485.43	-581.051	4657.05	6261.36
-3099.27	3509.79	-2288.95	-4496.4	-6549.79	2839.33	-81.8711	-3423.66	-179.838	-1831.47	-6137.81	-4646.73	-47.7871	-2362.97	-4832.93
-523.248	1266.78	2971.1	-5764.31	-1272.01	3914.46	-5744.19	-1610.35	-2758.1	4196.34	982.312	4951.01	-1064.44	-3945.99	4666.41
-10092	-1551.96	5711.77	-4378.32	-2516.87	2401.95	2360.15	950.748	-9642.9	-3764.37	-5460.36	1767.46	-1015.9	-2018.72	-518.924
-3661.16	-1765.89	-1371.72	-16122.7	-10133.7	-3871.13	-681.418	-3826.18	-1905	1072.02	2903.02	-5441.85	-2004.11	256.074	-321.512
3625.63	-3049.07	-6774.88	-13819.5	-2279.54	-4713.92	-2168.67	-167.373	-10306.3	5245.33	4885.1	-194.445	-6169.69	239.15	-6234.91
2224.93	-4233.18	6542.37	-11854.9	-2937.21	2663.95	-6863.91	-148.322	-7635.17	-1721.79	-399.336	4135.64	-908.334	4979.06	-1954.96

5878.35	-3510.09	8841.05	-7482.21	-4198.96	8203.88	-3957.69	1393.6	-13352.3	3106	1311.39	7089.36	-6398	-1650.5	-1733
3398.6	1656.88	-1659.35	-5453.14	-5962.13	-3271.05	4266.23	2221.06	-6075.13	8598.28	7686.56	1645.85	609.592	722.234	392.654
-1864.73	6757.29	2093.09	-9386.37	-6606.76	177.541	-8653.97	-3714.75	-6765.84	-211.867	4590.1	-1458.29	-1477.07	607.58	4010.64
-1452.96	1537.02	3587.39	-10495.2	-8196.21	-2170.83	-5125.18	-5608.27	-4054.86	1448.28	-3180.45	4075.11	-4192.93	150.18	2296.89
6062.8	-665.072	4704.87	-6358.3	1821.94	3916.42	-898.262	-1210.05	-6956.33	-4630.79	-4341.53	957.437	4038.89	-390.011	-8030.81
13893.8	4256.35	4075.67	-10088	-848.061	2765.84	108.572	834.777	-6829.01	-2430.82	-31.0781	-5295.5	2335.09	-6131.16	-427.004
-458.006	-2666.54	95.8789	-2966.67	-5713.56	7.97656	2724.58	5400.55	-4858.78	1278.12	551.387	239.064	859.303	2062.28	-5013.01
4602.67	282.145	-3381.64	-7844.28	-5039.92	6306.92	-950.643	-4398.18	-1270.39	-5805	-277.344	-2918.01	2482.87	-797.683	335.23
-698.559	-4975.06	-3989.11	-7766.45	-3374.19	4452.85	476.664	-7080.94	-9154.71	-969.748	4077.96	4399.85	2736.02	2358.22	-4751.07
3163.41	-89.4531	2133.82	-10835	-8616.78	-1559.42	-4968.77	3774.04	-9913.8	3740.06	-725.629	-3696.49	993.637	-1742.26	-9766.83
5714.03	-5277.65	-1409.27	-12861.9	267.131	-2887.17	-7735.15	7731.99	-9327.12	1511.62	474.883	-5558.39	-5251.35	2985.55	4630.8
2125.38	7005.08	610.748	-7620.08	-2856.76	-4910.97	4240.95	107084	-318.535	-846.075	4599.5	10919.1	30830.3	2557.2	17314.2
-337.887	-7540.31	-1643.1	-1948.74	-4942.53	2636.74	-3076.15	2015.52	-942.625	-2866.61	2179.07	-2905.36	-3334.98	6008.6	-822.83
261.715	-274.43	802.217	-7906.68	-10057.1	4664.93	-4697.77	2389.46	-7925.79	-2352.97	229.301	5295.53	-118.473	1629.94	-2812.42
6552.77	-567.904	3803.06	-7712.68	-4549.26	11492.6	-1003.34	6323.04	-3160.14	3900.29	-468.771	6551.35	33.6719	-2460.26	-2623.66
2273.94	-77.6973	4035.78	-8085.43	-4214.98	5537.12	-679.953	-1921.06	-8188.18	8758.99	2089.09	8384.31	3948.84	6510.51	-301.803
2258.93	-1562.75	6184.08	-8674.4	-7595.93	3291.14	-1288.01	552.15	-4145.63	7677.29	6224.2	4513.26	2959.77	1261.75	220.088
-331.424	-3184.27	6983.13	-6560.78	3686.25	6326.03	1297.41	-963.885	-5373.86	2034.45	4183.68	2075.75	299.822	-3643.2	7177.86
5717.24	6508.13	1069.55	-4616.13	-1538.33	2521.56	4877.85	-3575.87	-5247.69	6143.95	5894.94	899.496	-1519.87	-424.065	6854.16
2401.49	4974.64	671.988	-3608.33	-690.199	8555.68	-1820.04	7183.81	-6535.06	2995.47	-3737.52	-231.217	-160.201	1217.86	-2416.08
-509.48	359.203	-7763.09	-12328.3	1419.28	7683.35	-8380.49	5949.84	3682.62	1416.82	-2066.07	-3405.32	-1292.34	12953.1	-1629.73
1048.46	-1098.81	-15.0352	-7349.85	-4899.4	1063.19	-4410.59	2004.2	-5486.5	66.8682	4964.8	-149.421	-4833.76	5496.36	-159.029
754.617	2399.76	-334.387	-3308.07	-5018.5	4890.26	1118.62	10142.4	2328.36	274.849	1628.51	1559.79	2807.81	5803.93	-3733.27
516.781	-1935.89	4514.64	-10396.1	-5900.72	121.186	-6569.75	1083.21	1514.25	4827.08	-1321.07	3152.42	678.609	7666.43	-2119.66
-842.277	1700.37	7027.55	-9254.77	-4394.14	3409.76	-5241.9	72.1602	-5083.76	1188.23	-1374.49	969.162	2305.91	3047.54	-762.648
1258.7	3495.02	-1684.15	-12985.2	-7453.64	1451.4	1879.8	-2510.85	-8455.68	-559.142	-2894.98	-996.621	5273.11	4570.95	-1862.2
1493.97	1845.95	328.953	-6205.14	-6082.5	2743.37	-3264.36	-3741.54	-4590.22	4306.32	-5357	4627	-4156.27	3789.5	-894.095
4058.88	-1984.25	7103.66	-8246.1	-10545	2651.91	-520.686	-5254.57	-4093.62	3396.7	-4658.44	2031.4	4529.91	-253.686	-706.82

-714.697	-3105.75	1271.78	-2695.96	-6920.51	5280.31	-2472.66	4348.68	-4055.82	-360.098	886.168	660.264	-6533.08	-2574.89	-6672.37
-518.533	560.674	1011.25	-3526.14	-6697.95	3352.24	-3374.95	399.619	-6048.9	1575.24	6738.22	4688.78	2108.11	657.541	-1972.56
814.896	-1061.09	1173.15	-9110.97	-2544.9	-131.578	1285.1	2180.89	-2904.74	688.372	999.215	-4258.9	-1402.61	3845.8	-837.779
-1689.67	-129.273	3649.14	-6567.4	-8061.03	1525.31	-8686.89	205.842	-7833.11	4736.87	5585.71	-5189.11	2481.16	-4153.46	-664.158
-930.838	-6589.17	-444.783	-6830.04	4842.79	-6098.78	3513.84	-5030.04	-9121.79	-1507.87	138.504	-5256.61	-7964.8	-2059.03	-308.779
-2151.39	1293.6	359.951	-3278.47	1059.32	2825.85	-4074.16	-1488.13	-8221.96	-2382.74	1099.44	-3823.36	1786.83	-1004.88	-5361.69
4508.64	310.574	358.4	-6027.91	-4704.56	2697.34	227.213	-1893.06	-7565.54	4240.77	723.301	-3640.67	1676.82	-1466.27	-5346.04
5739.62	5471.44	-4373.87	-11773.3	-7072.68	-6773.54	-4341.68	-844.869	-6964.61	10330.3	7100.32	-2444.39	-2103.64	-600.965	-2025.5
-2966.66	-6432.93	5375.96	-10076.8	-5278.52	-1542.03	-2887.24	-2473.87	-6435.67	2398.19	1731.75	-1479.82	4076.14	3434.82	-1505.5
-2001.74	-3175.49	3281.67	-1197.28	-5118.22	176.098	1923.81	738.939	-8965.68	-2405.74	-1009.42	7407.93	-5817.79	7886.2	-1773.77
-4970.74	-5111.69	4260.22	-7689.93	-7111.16	-3174.15	1157.29	-4955.82	-6028.75	1163.2	-2831.65	4410.9	750.072	6707.38	-7955.38
-2746.12	1511.17	6347.29	-1709.34	-4385.81	4059.53	1112.86	3559.54	-14302.8	-1649.89	-2628.44	5245.77	-312.346	-238.248	-638.07
-621.703	2870.26	6591.25	-6965.04	-358.176	7292.58	-1585.41	28186.5	-6224.28	1651.06	2402.23	-542.461	8099.06	2240.12	3732.5
6446.08	1040.16	-1857.78	-6245.06	-11104.8	-3022.23	-3804.73	-3731.92	-7827.57	1515.31	2145.19	-2287.45	1153.63	-717.446	-182.496
6991.36	1412.21	-2366.53	-6940.91	-6814.2	926.932	1672.09	-4292.27	-1655.28	-58.2822	-6102.96	1717.7	3714.79	694.83	-2877.3
3347.87	513.078	1083.54	-6411.5	1716.62	-872.178	-2288.81	-259.646	-3395.94	5392.31	-3179.94	-1300.7	5267.52	5276.85	-263.414
2562.88	4400.66	-2033.35	-5821.18	-4334.93	-4425.44	-2468.3	-9570.56	-5256.79	3048.39	-2531.69	-2285.18	6249.55	3982.83	2854.6
-1343.83	-4119.55	-4029.17	-623.34	-5295.58	-1412.01	-2626.84	-6063.34	-8199.99	664.886	-1085.62	-450.316	-1595	1767.75	-1481.82
2215.07	-811.414	9521.36	-4591.38	-7071.77	784.824	-1259.34	-5833.06	928.061	2063.14	-425.844	-1037.79	-1500.8	-177.726	-2777.43
-1444.94	3883.81	-2967.95	-5230.79	-12063.9	1570.21	-6036.36	-1332.61	-7403.42	-4766.13	2414.42	840.494	556.125	-1255.99	-8323.39
1001.26	-1499.05	-4212.09	-11326.7	-2590.79	3085.66	-8464.01	-6469.33	-9774.58	-1943.7	5723.51	600.179	-3426.91	21.9648	-5168.9
-263.467	-545.975	-4115.28	-8751.25	-5605.2	3099.5	-6757.61	-8812.35	-241.154	2120.12	-2152.28	1884.43	-1918.88	58.1055	5131.98
2867.23	-3204.41	3060.64	-8362.94	-4501.63	-4067.73	-5998.49	3322.25	-516.373	-3121.75	-683.232	-2216.48	-5242.69	7201.08	-4638.87
-673.086	1060.69	1070.38	-12947	-5184.63	4327.02	-5364.8	-561.482	-6272.9	96.0039	8198.04	-232.66	-1592	115.656	-343.709
-1807.7	-4276.55	4267.89	-6114.41	-3974.1	6956.25	-3900.46	-4036.1	-5609.46	-418.925	-177.094	-6455.66	1150.82	9949.54	843.686
-1112.36	2094.6	4443.82	-6170.59	-1080.48	-2613.41	-13736.4	2561.2	-8013.07	-5393.21	3572.38	4339.97	-6528.01	-825.662	-7026.37
-184.584	-1549.84	-2033.03	375.238	-9764.74	415.273	-5666.42	882.035	-2468.99	-1272.6	-2928.1	-2777.26	6351.68	-9884.93	-3895.99
2588.82	911.859	-4364.13	102.971	-5109.93	5383.03	-3535.47	-2507.43	-7056.14	3557.19	-1994.51	-5225.03	-3488.34	6911.11	-4783.51

6693.84	4501.16	7974.72	-10021.9	-5750.97	2285.05	-1160.3	-4105.14	-3032.92	-6258.04	1486.3	-3798.54	-3958.99	-795.909	23.1621
-3653.64	-4046.13	2351.91	-8846.88	-7885.83	114.418	-2091.08	-410.936	-2402.3	7507.53	-177.475	2092.1	2394.89	4581.2	1515.84
-46.0469	-7034.67	-916.576	-3045.53	-11319.6	-475.975	3214.13	296.029	-1169.49	1480.96	1694.35	6972	1238.72	8206.31	1702.87
-4831.36	596.303	3875.66	-6640.26	-8055.38	1304.69	-8070.12	-1801.2	-6836.53	725.928	592.313	2567.63	-2855.14	-53.0039	-3359.02
-1463.85	-1043.5	-1932.49	-8078.77	-641.373	786.211	-8053.13	-5403.4	-8262.2	-919.046	2148.23	-6861.69	-7901.93	-2364.43	-4823.2
-3793.17	673.635	-4058.45	-6691.12	-10266.4	4205.96	-4071.27	-2862.25	-4278.52	-3090.43	3397.42	4447.31	1140.38	-2490.07	75.3672
2176.92	-2256.73	227.975	-748.709	-9817.83	1067.13	-1978.19	-3392.38	-4267.54	312.33	684.809	1264.71	1793.53	-6063.26	-5853.68
-2568.59	4682.2	-1099.18	-8786.39	-3829.31	4193.71	-256.797	-1844.4	380.264	-397.58	219.266	-3540.93	4198.33	6045.76	-545.797
-4029.07	1100.79	1420.29	-9416.57	-9153.99	-3223.02	2451.41	-6045.47	-3464.28	4798.76	-1301.61	1572.58	3335.15	1739.12	-6973.84
10908.4	-4119.7	478.932	-11046.2	-11048	5209.12	-1157.34	672.008	-6175.17	-1279.39	380.232	-277.526	3055.53	195.525	-6692.75
-2513.15	-2736.86	5436.21	-5353.82	-6360.44	1344.13	-2587.43	-3788.02	-3012.53	652.736	-1137.19	-1358.37	-3012.8	5226.5	-2683.46
-4309.49	3149.79	-658.863	-9859.67	-1783.44	1893.34	-3294.74	2200.3	-9263.23	-3229.91	-1193.76	-995.134	236.211	-1467.13	-945.84
-2723.68	-5745.96	3784.34	-7714.1	-6305.47	1941.62	-1001.77	-0.779297	-7778.06	1382.03	1733.2	-3488.55	-2088.68	-2112.93	-5041.86
-6385.96	-5131.46	-2837.84	-6882.36	-4325.03	1962.23	-1327.17	-6758.67	-2120.75	-1236.3	-16.9883	-2254.27	3032.57	4058.89	-5667.95
6380.43	829.668	-4273.13	-6310.46	-8468.13	4155.27	104.074	-3392.71	-8136.66	-902.797	3316.38	-1474.3	-982.906	4248.39	1317.67
-1164.4	-2337.77	1951.42	-9004.79	-11491.2	4869.02	-3680.58	-6504.95	-6903.26	345.492	3635.76	3869.44	4330.29	2381.45	1762.97
-1037.07	-2035.06	3658.44	-9519.03	-3330.56	677.523	-3796.04	1242.91	-9163.98	3643.93	4387.88	-3598.65	3617.45	-1872.22	624.402
-997.078	-2292.64	7848.98	-1915.71	-10008.2	845.699	2920.32	-6954.33	-6252.12	4709.24	3976.72	5011.61	-6106.34	-966.838	-4461.93
1633.13	1049.03	4642.17	-7836.76	-9758.5	2918.82	-1352.8	-5355	-5447.57	-6517.5	1482.75	1297.27	-2608.06	3827.94	-12001.7
-512.385	-2251.08	5363.92	-3840.64	-6523.69	949.922	-5881.31	-4915.55	-3081.6	672.641	3328.98	3516.2	-1290.71	-5236.21	445.996
-3754.84	-1612.96	1804.02	-1282.73	-9112.46	-1415.34	-10678.8	-3109.14	-9278.79	-2563.91	5474	-2848.28	1184.66	-708.555	-1361.35
-600.977	-1927.75	-1242.09	-10530.5	-726.463	3154.72	-5705.06	2069.33	-5920.07	-615.937	7010.31	1889.86	598.039	169.92	-3774.17
2436.83	6485.02	-1982.32	-9952.2	-1055.72	5312.76	-5303.98	-329.781	-10516.5	538.479	-2948.53	-299.327	-1766.73	-976.955	-5452.37
-905.365	238.467	7699.87	-3455.07	-656.619	2072.05	-3530.1	-3327.2	-9531.12	1337.79	4957.98	6204.8	-2015.81	3257.37	1389.88
5809.11	-1412	-843.932	-7852.46	-7846.21	5900.07	-3981.64	-2737.08	-2843.49	87.1045	287.967	-4003.07	-3723.28	6794.68	-7676.84
-937.705	-5684.57	3830.08	-12467.3	-6124.07	-3133.05	-4064.08	-3877.32	-2784.78	663.187	-2953.01	787.895	-5994.26	-3338.5	2940.14
2333.12	721.719	3374.34	-6696.04	-7920.1	2259.84	-1750.78	-4024.46	-4225.19	-3870.21	-3874.57	447.454	-2444.03	-4933.75	1869.62
-634.721	-2271.13	872.316	-8410.14	-4102.86	3532.31	79.9414	-7363.38	-4531.35	-5249.41	3859.14	-3584.89	-349.816	-4905.72	-4717.52

-2235.14	-5500.75	873.867	-9392.17	-4547.15	4202.38	387.807	-2873.25	-1271.41	1553.64	4521.5	-3485.26	-2308.29	-5326.56	-2122.88
-350.84	582.209	-6494.08	-4102.55	-2752.15	903.025	647.439	-2426.71	-4207.44	1198.43	-2092.22	-5610.62	349.514	-6242.87	-3853.98
-3315.82	-6213.78	-454.48	-5845.71	-5512.61	-2009.56	-3757.51	-4652.66	-4733.08	-2439.09	1396.49	-2350.42	-3440.95	3296.69	-380.936
1678.65	-5419.68	6043.16	-8987.97	185.762	1440.89	-3936.97	-5107.99	-9735.09	2782.95	-2938.54	-76.2148	-6970.41	4408.7	-322.135
-6722.72	6106.86	-1870.81	-783.391	-13272	-6433.41	4861	-2983.79	-4121.26	6429.22	-63.0586	2131.73	1338.25	-3091.07	3685.72
-9888.88	-3911.12	-1020.89	-1441.26	-6347.14	-1404.11	-332.158	578.074	-8463.33	5528.98	137.846	3351.48	2220.08	-5563.62	-6361.3
5786.57	-2777.01	-571.275	-4280.08	-8394.89	-619.33	93.3691	131.699	-2968.99	4327.33	508.535	-2451.65	-2280.22	6068.68	-8738.54
2237.98	-7798.91	617.426	-8781.41	-10514.5	-4477.5	-1969.82	-3434.79	-4464.19	-2933.3	-2326.63	-2414.12	201.221	1115.23	-2245.03
1077.67	-1510.56	5154.06	-6278.16	-1892.76	1153.32	-3109.91	-3183.26	-15193.2	-414.789	-702.264	3351.41	-7711.8	592.689	-8350.42
7589.96	2530.95	48.1504	-7467.71	-11948	-3901.02	-3090.52	-4407.86	-2121.27	-2874.48	-5672.42	1912.54	-1700.82	6949.29	-340.789
-380.809	-958.516	-215.443	-4572.69	-14653.7	1001.42	-4741.5	-5813.01	-6776	2215.03	2344.17	-10373.5	1553.41	2626.33	3687.29
-4115.64	-426.635	5988.55	-6734.67	-3108.06	6238.76	-799.221	52.6484	-3345.44	4748.24	-6608.52	-4469.83	418.605	-1678.63	-3564.54
-4095.13	1067.98	3741.74	-7423.99	-7718.24	4226.62	-3509.56	-849.818	-4235.84	5165.57	-1927.58	-1400.24	2878.54	7864.35	-6844.66
7482.39	2012.34	1411.95	-10678	-7703.33	1188.07	-6069.98	-4492.52	-5109.85	5433.22	4593.37	-646.329	415.014	-902.482	-5374.5
2546.02	-4982.14	1106.23	-14241.8	-11804.3	-4261.09	-7896.9	-1296.05	-4053.64	-1722.16	-2120.51	3045.53	-1827.97	1783.29	-5049.89
-346.533	-6670.67	141.738	-11674.6	-7936.18	3817.61	-2183.63	-356.666	-4218.86	2416.84	545.635	-5301.16	2918.71	-2846.34	-1609.42
-6087.69	-5242.78	-5277.79	-1590.5	-1510.77	6027.92	183.447	3164.45	-6153.73	5025.79	7708.18	-1141.05	5100.46	-2733.64	-2337.73
4482.05	-2459.69	-1139.08	-3828.67	-5738.14	3002.07	-1064.85	1369.82	-5724.84	2631.54	5562.11	-2469.1	-3355.27	5533.36	778.07
6786.86	-4535.94	1039.38	-6854.8	-210.701	2296.83	-3407.28	-1171.49	-11555.1	155.754	3071.97	-817.383	-1736.52	-85.0254	184.373
763.326	-1521.98	540.529	-11114.7	-5965.03	-3486.85	1146.27	-6451.48	-10967	-2740.17	4174.69	990.311	-373.182	-2582.9	-1275.75
-3110.6	1776.88	-2296.11	-9861.31	-9671.48	4298.65	-7978.29	4893.6	-6985.25	-5531.95	2912.82	566.202	544.424	959.187	-3153.9
2706.19	-2908.71	118.252	-6422.28	-2336.24	-5038.21	3577.66	1520.43	-3954.98	587.632	-1382.27	-1653.27	-3681.51	-6085.43	2215.84
359.016	-3461.92	-170.021	3170.67	-5468.58	4575.98	-4444.1	-7430.19	-3068.08	-1003.94	-6612.17	-3168.09	-2091.42	-9618.59	-2915.29
-1424.4	-1047.79	-734.686	-4183.59	-2435.56	1646.31	-2762.5	-7106.74	-1827.12	-5407.28	-6325.48	-331.095	1673.68	2267.97	-6226.23
3887.79	-4017.88	608.086	-3788.03	-6730.13	5243.17	-4864.22	-3732.62	-2315.55	-4669.42	627.309	1938.27	-2705.04	990.219	-1016.46
3866.99	-2897.17	6238.04	1676.71	-9492.66	7082.19	-7648.81	-1979.3	-10194.5	-25.7852	-1016.27	-1889.54	-1691.31	-3761.9	-5793.2
-3929.84	5346.81	-3052.83	-7922.21	-5268.58	2975.42	576.402	242.721	-7740.29	3570.26	-1503.32	617.539	-3631.79	2462.03	161.285
1330.28	-5930.15	413.361	1971.06	-6600.05	31.2188	-92.4375	1290.09	-5771.87	647.788	-54.0371	-2652.47	838.197	-5911.32	-799.795

3503.08	-809.773	-4914.13	-7480.63	-5720.13	1357.8	3010.3	-2712.08	-2595.09	921.242	2166.78	-3170.34	-7276.25	4249.28	3634.63
4459.32	2901.61	4630.52	-10172.3	-1215.23	4726.69	3522.62	-729.279	793.123	-3983.36	8611.99	-1478.9	2649.34	-111.336	-5459.4
-20.1367	-1364.95	12502.9	-3161.44	-4865.88	379.414	-2054.63	-6750.23	-7145.85	-5746.39	-953.344	-342.3	-4050.97	-2678.23	-10501.7
-5704.04	-5286.49	737.719	-6316.43	-5544.1	641.607	-4231.09	-2993.29	-6305.03	3770.12	3225.89	-3715.39	-6265.34	-6772.35	44.9531
4823.83	-1042.74	2566.27	-10621.2	-8437.17	-340.107	-1588.58	-1840.09	-9671.2	-3628.81	2939.41	1958.51	-3226.01	-1287.08	1658.4
2535.31	-1218.97	-1913.25	-5845.18	-10593.7	2003.41	-552.455	-2810.95	-7084.77	-2829.87	-4082.97	1241.96	-1531.68	-276.137	1214.8
-31.9668	2346.88	3911.39	-4627.38	-8654.1	-5267.07	-2051.39	-1852.73	1274.59	809.719	-10729.2	3443.58	-1931.87	-7731.94	-3393.6
-5159.38	-3009.25	23.2734	-6725.34	-3607.93	-6454.79	-6726.86	1627.04	-1164.01	6527	84.6543	-2118.54	-869.418	-629.519	-9745.56
3382.71	-3736.26	-4820.31	-8396.79	-8390.54	1827.03	-7019.76	-2263.89	-8602.16	1983.56	-2911.96	3325.71	-1995.04	-660.836	-9445.14
3713.41	-3982.1	1716.47	-8517.25	-4351.76	-2162.24	-3235	6307.83	-9033.56	865.222	837.943	2527.63	-4087.22	1382.4	-4124.66
5003.08	-8191.31	637.295	-9671.86	-9050.6	73.123	-1245.88	2523.68	-4692.44	-1202.91	3082.41	-465.399	-3363.19	-2434.47	-3980.67
4408.22	-3960.17	7211.87	-7481.95	-11485.8	2396.31	1040.85	-7398.12	-6193.8	3298.36	3584.74	3911.18	904.92	-3559.44	3517.51
2218.78	-9106.58	-575.328	-1387.56	-7812.7	4611.79	3539.29	-2161.3	-9889.23	-2920.42	1247.28	1199.8	-3123.18	1948.85	-5305.67
-4753.63	-225.711	2067.18	-788.83	-3190.35	1643.98	-3378.89	-10409.4	-5356.34	323.853	-1941.06	2629.02	3134.06	10652.8	-1145.33
211.955	-2602.51	-1447.06	-4912.83	2125.32	-3606.66	-384.469	-2232.07	-4199.66	657.647	1980.9	1569.4	-546.912	-1522.54	302.654
-8375.65	-1359.18	2795.01	-4493.6	-3071.86	1009.05	4132.08	-10791.2	-9602.93	-4343.67	2499.26	1582.83	2731.28	2876.45	3020.12
-7140.89	-2475.58	3399.94	-2925.43	-5482.89	-2810.34	1729.64	5643.37	-5194.65	1960.15	-3327.5	933.855	-2420.41	2436.03	520.641
6389.48	459.469	2502.45	-15034.8	-6832.01	5179.97	-532.578	-3435.24	-10047.1	1156.29	-2495.93	1072.92	-8275.65	5407.68	-5013.99
1131.43	972.061	1645.5	-4560.92	-7171.7	2956.92	-3758.45	-5136.8	-7203.31	88.9512	-3391.46	-3135.08	-3333.2	-754.084	1546.85
-3823.55	-5385.84	6937.98	-9961.99	-4522.65	-542.924	-12762.6	-3305.36	-5967.44	-930.641	-5929.61	-2620.95	2049.13	1128.39	46.4941
1878.96	-8157.08	2282.45	-4120.09	-1146.65	-4494.55	3266.3	1230.25	-5743.24	1495.51	-857.021	-2062.52	-248.104	4235.35	542.836
-2835.74	-2779.32	1354.56	-5059.1	-817.875	-410.496	-2928.91	-6524.18	-4286.11	-3215.51	6351.39	931.512	2756.2	-6338.83	-3850.03
301.203	-7111.03	-4400.5	-6645.96	-5692.2	5856.2	-3027.82	-7943.3	-8668.72	-4828.02	-800.156	-3727.56	4713.86	-421.041	358.32
-2020.59	-662.643	6578.65	-8131.78	-11795.1	-907.76	2972.71	-6210.73	-15797	2035.52	2727.43	5821.43	1327.21	-6700.48	-4715.79
-359.893	-6312.58	-387.875	-11271.7	-8812.35	-161.809	-1275.63	-5881.22	-7042.98	1548.43	1207.62	-59.1709	-1762.21	-3835.95	-6277.68
-4295.94	-6582.12	-3292.86	-4253.28	-10880.3	410.309	-1094.63	3295.83	-1491.25	5506.3	4552.52	-4758.53	-626.002	7268.72	-270.279
-3840.72	-4395.56	-1480.64	-10517.5	-8227.21	-1201.68	-3712.11	6340.9	-3380.47	294.604	4901.24	840.088	1114.23	3733.52	-688.68
2152.67	-376.912	2628.07	-7536.71	-374.863	3557.94	1674.85	-539.125	-6424.26	3993.35	-1142.03	2871.28	2870.35	-4900.45	-663.51

-3871.12	-2095.54	-2564.59	-1812.92	-5926.74	7704.98	-4220.25	1013.57	-7200.87	-4542.16	-723.145	-3023.75	1498.91	5169.78	-2115.01
-6389.14	248.885	1334.93	-5504.41	-2949.02	2665.05	719.697	-4732.23	-1126.54	-313.9	-568.818	-2254.94	8632.25	5597.98	2050.36
-4349.48	-2206.28	3379.91	-12084.1	-7481.16	2347.48	2105.02	-6189.71	-5040.85	-342.372	3542.73	-2124.07	1360.69	972.408	-1808.54
-5814.45	3264.03	6933.57	-9353.8	1547.95	340.031	4330.33	760.014	-9733.81	-4321.37	-3784.53	-4828.92	-288.611	5302.46	-3382.32
5159.03	-1037.21	3311.16	-7875.86	-4335.2	2701.36	-9125.46	-5926.02	-5243.01	-3369.5	2048.16	-3212.73	151.752	1819.64	-8819.19
-8629.65	-9261.7	4264.99	-5649.23	-3439.33	-334.645	-3784.29	-5689.27	-1202.44	3686.66	2611.01	-4037.52	4658.28	230.232	-2506.75
-2831.31	-558.215	2438.31	-2573.68	-7791.68	698.934	-5862.62	-1810.72	-8043.31	1286.57	-719.674	2394.77	-408.578	-2890.75	-1341.72
8307.84	-3919.56	-4408.56	-591.23	-9343.54	-1247.72	-1272	2413.18	-4297.79	-1111.04	4917.74	-1947.13	-3023.25	5558.45	-3947.42
-3244.58	-8318.37	-487.225	-4659.88	-8743.11	-795.771	-327.904	8896.45	-3050.2	-7364.34	4209.43	-2113.2	-7254.29	3247.8	-6748.69
4499.56	111.412	3946.24	-7800.5	-8639.08	1895.39	644.719	-4975.45	-536.725	4366.95	977.346	-898.029	7664.25	483.145	-4064.59
4360.55	-6528.03	1050.67	102.74	-9853.77	-3133.97	-2408.22	1652.48	-3095.09	2516.99	1479.2	4311.81	6431.47	3612.92	-3466.02
4985.22	-5886.59	-515.918	-11216.2	-6599.16	-3449.41	-6093.2	-3941.49	-9684.69	1591.49	-191.969	-643.082	908.475	2004.76	-6641.87
-446.051	-2053.47	-5406.47	-5447.38	-8391.13	-4967.19	2690.61	4327.29	-9565	1138.09	-2907.93	288.033	-2036.47	761.193	-755.078
3819.8	-2160.08	-6828.33	-7281.05	-11875	-3561.65	1011.62	-4739.3	-2743.32	4492.29	4799.37	-882.676	-142.947	5540.56	-2562.61
-4163.08	1585.5	2023.11	-1228.2	-9950.92	-1557.31	-4140.25	-3742.52	2905.05	1392.96	2811.66	-7294.29	6556.99	1585.82	-2733.5
3480.54	-385.174	-8285.71	-6222.48	-7848.15	4540.65	-591.305	-687.23	-311.377	8849.91	1808.18	-3522.78	3689.47	5450.83	-6030.82
-3539.82	3588.85	-2978.08	-11685.7	-3587.07	-3105.21	-4104.71	-11.957	-2840.65	-1171.3	1407.71	1884.13	5307.36	3065.4	-5943.98
-2268.73	2892.01	4828.27	-8806.63	564.01	-1807.47	-4288.88	-4411.6	-1803.8	1480.08	1112.89	4549.76	1624.91	4847.9	1837.34
-512.947	844.338	4278.43	-922.771	-3601.83	-439.893	-6983.98	2904.77	-6562.99	1843.53	2638.71	-1020.9	-5079.77	-364.247	423.475
-4439.75	48.5977	5201.9	-7910.28	-6403.25	3054.68	-1054.88	-29.3457	-3875.49	1013.75	2687.64	3829.79	-7305.87	1333.33	-8533.59
-1569.39	-6341.5	4792.94	-11996	-4759.97	3868.25	882.277	-6247.83	-2044.65	263.807	5394.66	-596.188	-3864.5	-1644.81	1694.95
-1670.44	-3268.34	653.748	-9053.21	-6149.89	385.736	5422.19	-994.436	-5473.04	734.385	2279.25	-3375.01	4061.22	8792.78	-1704.8
8301.28	-6063.94	1659.61	-9264.07	-4487.89	8182.36	-847.311	556.598	-4846.01	2125.92	2042.31	3638.03	-1280.34	-4875.89	-2610.3
-3320.81	-96.6504	2087.84	-9834.84	621.74	-252.969	1593.73	841.707	-1869.71	-3371.57	-2014.83	6678.91	-762.965	-1262.75	2841.87
1411.62	-1894.83	-1388.81	-10778.3	-4598.68	646.932	-3505.58	-9739.68	-5285.72	3932.12	-162.295	387.73	-4281.91	-3786.44	-6274
-762.738	3166.22	1077.73	-4764.48	-4887.77	-2190.11	1061.11	-603.771	-5240.07	-1281.61	-418.975	3300.03	-2499.35	-2868.85	-1605.72
3791.2	-828.363	5244.7	-8005.39	-10058.1	1132.58	-2968.57	-3321.27	-7529.32	-8195.48	678.219	3231.85	1506.72	417.771	-5733.35
1165.61	4382.58	1469.93	-12072.9	-8923.6	4913.34	-774.844	-105.752	-9162.59	-774.767	-3640.16	521.134	-3432.84	-3029.45	91.4531

-635.625	-1796.99	2283.84	-6221.32	-3105.48	-94.498	-5663.53	-7599.43	-8726.64	3227.16	1808.28	-6615.37	1190.69	2696.45	-4516.48
-1195.43	-400.635	-1297.32	-6303.94	-4685.35	8527.92	-1224.19	-1566.02	-3956.04	-3187.04	-5003.69	-1086.8	1289.67	-800.446	-4233.07
-2242.95	3215.98	-3256.5	-9289.15	-4088.22	-3278.07	-6232.14	2244.66	-5458.53	-4965.1	1066.17	1390.43	-2884.17	355.736	331.096
7063.31	-1809.42	1274.76	-13402.3	-4561.84	86.5742	-11044.4	2573.44	-3547.37	1972.19	5020.57	6334.93	-5175.85	1119.97	-2541.93
4165.55	-1915.21	3459.59	-10347.9	-3974.71	-6782.94	-107.119	6148.27	-7845.61	-969.748	2805.81	-1566.19	-2382.01	3494.38	-2669.18
4577.6	-6396.75	4726.65	-8714.32	-7875.41	3459.51	-9592.42	-2435.43	-2312.05	-1927.26	-7913.21	6842.43	3927.66	5064.44	2805.89
1491.01	-4779.06	4661.96	-14291.7	-7546.61	3155.08	2668.61	11850.4	-6753.25	-5021.82	-6876.27	5943.31	13800.2	-5396.71	-904.368
-2005.14	-2741.87	3753.38	-7559.29	-2003.58	1902.96	-29.9805	327.422	-185.906	-99.5312	957.783	4167.41	168.674	-3756.27	-6497.53
-473.551	1147.96	-1220.62	-8744.98	-5551.37	1386.45	1806.74	-276.674	-613.873	154.721	-441.631	-2191.3	-2946.9	-4052.2	-3376.21
-1572.95	-5880.3	6343.83	-9267.33	-7908.7	1517.88	-684.207	3161.99	-6110.19	3872.67	4624.64	452.437	1973.67	1766.71	-6570.82
-1401.33	-4257.14	1774.57	-5076.84	-3576.3	-2440.82	-5996.08	1200.98	-5090.56	4593.58	438.264	-1888.15	-4536.39	1767.86	2600.34
-2595.63	-2526.21	2706.55	-4180.26	-8460.12	3853.22	-8118.9	-2221.92	-7930.57	-4435.25	3557.76	-251.26	-3615.42	-1673.47	-4837.42
-3304.05	1871.44	3773.41	-10477.7	-12911.5	-1926.94	-431.199	1323.24	2507.86	6784.25	4551.21	2244.46	6356.08	-2270.3	1624.14
-969.576	-7983.34	1607.35	-631.02	-7203.57	-647.119	-988.619	1748.46	-5544.98	-3178.32	6183.43	1561.98	-2111.64	1689.29	-157.84
4022.86	1919.91	1270.19	-11128.3	-10516.4	408.09	-1709.21	-4202.3	-6673.65	-2140.64	-5371.04	-1267.67	-4923.21	-2638.6	-5105.42
4029.66	-6466.76	1269.28	-9124.98	-3071.39	-3982.84	-5367.31	-340.398	-7398.05	3013.23	-1955.08	-3762.78	-138.947	-2328.11	-8265.07
2644.23	-5378.52	1687.38	-15500.5	-7138.22	-2793.01	389.375	505.514	-6052.9	-6117.41	3679.9	-2060.44	2066.72	-1014.14	2039.11
-1643.35	-2974.02	-1065.92	-6320.65	-3462.97	-520.875	4478.96	-1014.71	-4307.19	1089.78	2511.62	-9322.01	1455.4	-3052.53	-785.385
1462.47	-1427.16	2169.87	-7608.31	-2224.52	2938.42	-2672.18	-5018	-13865.1	2044.09	417.713	1254.43	2023.99	2955.83	-2005.14
-498.4	-5092.64	-631.322	-7011.88	-8557.95	1941.82	-10994.6	-2744.54	-12365.3	4391.03	6299.36	-1154.28	-1571.67	5258.13	-8221.82
-959.494	-8129.11	-234.719	-5432.8	3266.07	3808.21	-9532.57	-8845.08	-11498.2	2560.23	-1723.69	5382.35	991.377	-4023.05	-1539.75
-1174.88	-621.666	937.809	-12120.6	-4465.92	765.826	-2326.3	1253.55	-6968.66	854.882	1477.5	-1706.92	1993.7	3008.55	963.344
-2007.73	-5956.6	4893.68	-12203.4	-5089.6	2130.27	278.756	-1261.4	-791.658	4828.56	-1794.26	-2603.68	-3232.26	-4858.71	-4221.2
1307.8	-7396.03	2911.1	1660.36	-4769.71	-4290.88	1713.11	-6406.29	-11982.8	-6898.08	-3894.92	691.692	138.83	623.199	-9293.5
-85.4102	-4117.39	-1308.09	-3052.95	-6131.61	1002.64	-449.23	-7752.4	-6451.61	3567.97	1914.18	936.854	1396.08	-4924.76	-3079.23
-2372.15	2291.42	-2777.2	-7901.28	-12005.1	-2224.39	-2602.29	-212.027	-4758.31	-3388.48	1840.84	-1492.58	4153.82	1385.19	926.387
-2550.8	-2794.51	-4169.69	628.979	1332.83	2084.45	-7010.73	-1613.23	-1361.98	-1421.83	6530.62	7684.58	-2174.73	2370.32	-3615.72
26.875	-3350.44	2112.4	-6248.88	-4625.85	672.17	-3630.96	1785.66	-2204.23	2419.09	450.402	965.826	2082.64	995.146	6142.62

-1415.41	4723.99	6833.58	-3637.93	-6592.55	429.334	-2748.55	2056.49	-4042.89	1255.92	4680.37	2240.24	8852.52	3629.06	-284.637
-3466.46	-2229.46	961.691	-11209.7	-927.541	-737.504	-6808.35	2605.77	-6499.17	-126.858	1148.09	775.72	5260.67	-395.799	-9511.51
-2700.67	3129.17	2089.83	-6638.74	-4212.4	8307.27	-2001.24	-4574.31	-2753.25	-5743.77	955.832	-1530.91	3316.27	-892.312	-3369.35
-4509.74	-6350.04	4202.6	-7103.86	-6728.1	-674.992	-2935.44	-1771.44	-15114.4	-2438.2	3455.48	1450.31	-150.463	3248.84	-5760.14
5000.17	-1956.15	334.398	-7037.7	-6426.64	263.875	-3161.56	-2442.18	-7126.36	-6287.25	-2554.14	7564.98	933.689	4006.43	-1482.68
3164.35	-9183.09	764.424	-1905.86	-5085.43	2602.49	-4390.5	-1295.31	-7926.15	-1281.2	-2377.01	-182.608	913.953	-2509.39	5386.14
3078.32	-4886.04	1816.86	-8710.17	-7455.99	5163.13	-5550.99	-5101.62	-7500.46	-3521.87	-2181.7	1788.38	-170.385	-2848.01	103.484
-6.49609	-491.514	1777.83	-7356.64	-4541.96	-1499.43	-1364.35	491.191	-151.67	-4927.88	-530.705	10532.7	-2578.22	-530.56	-2502.89
4995.96	-520.795	3826.46	-880.014	-548.031	-152.379	-4562.46	-2894.34	-8522.57	-84.9814	-272.934	9399.21	-845.275	-2797.24	-7541.77
5539.5	-1247.68	1427.76	-6966.75	-8013.37	-3788.37	-3159.64	-684.465	-1984.22	1590.38	-5832.47	-1315.15	-3334.79	2964.3	-2660.91
-540.885	-1025.55	4527.63	-1585.23	-1465.95	-3352.29	-1775.86	-196.861	-4809.04	-6463.58	-936.137	7310.55	-1454.37	943.465	-2338
2092.57	-2452.92	-1078.75	-6564.93	-7355.68	-3100.27	-7691.42	5275.19	-1338.66	1219.22	490.848	-28.125	-6057.13	-4795.92	-5993.43
6033.55	-3101.89	320.051	-10804.2	-10682.5	789.178	-3461.02	45.0957	-10413.8	-1661.97	3853.01	-8816.11	5360.83	1320.5	-1930.63
-2792.7	-2384.49	6442.42	-4594.44	-10267.2	856.986	-46.4043	3073.44	-6244.93	28.499	3753.97	-2923.58	2170.54	5034.37	-7078.25
5569.87	-5315.95	-805.186	-5168.75	-5613.66	-3132.28	2427.28	-97.3184	-10073.6	-731.93	-5383.4	-3731.91	343.662	887.713	-7145.21
5410.82	-8946.17	-1096.76	-12406.5	-9991.79	-3360.97	-5452.75	-4992.22	-7953.4	1125.68	159.893	-7354.92	7408.87	-3973.3	-1798.46
902.145	-6131.62	142.334	-15032.7	-5681.92	1208.54	-1823	724.615	-7517.48	3755.94	731.486	-1780.92	1286.52	1238.72	-8922.44
4384.53	-5.31641	3908.96	-8807.91	-7422.45	-715.066	-3775.16	-2963.16	-4356.17	-1178.84	3555.27	8063.55	1983.48	392.713	-2635.96
-3529.68	-2103.8	-1061.27	-12389.7	-4378.94	-3971.5	-3777.39	4972.27	-6932.05	-1033.04	-3494.4	412.035	-225.072	-985.508	-1445.78
5726.76	-2898.75	3437.73	-12831.8	-2718.94	2528.41	-2135.19	4721.92	-14763.3	1760.07	-1755.85	-6982.53	-1334.66	5524	-6072.88
7636	-4215.38	4962.67	-6384.54	-12186.8	-6223.19	2597.71	3334.31	-9387.77	-2988.95	-2310.41	-3336.89	-7695.65	-1201.1	3198.36
5371.74	-6312.19	2082.6	-13145.5	-6943.52	3089.07	-849.822	-1004.35	-6267.92	4069.16	-850.408	38.3848	-4123.13	-3788.47	781.91
4669.31	-2664.57	-4527.35	-9058.95	-2013.89	-2135.36	-4242.01	-6659.22	-2920.97	5717.16	4398.5	-5927.26	1277.04	-774.788	3540.95
8245.34	-762.117	-2757.21	-10747.1	-9188.38	3715.77	-540.947	-3040.08	-8277.7	-10214.9	2199.35	-3115.9	-304.977	-929.992	-8329.69
3377.87	-5875.68	-3730.76	-7263.45	-4391.97	-740.693	3313.49	3736.29	-6829.08	3432.52	8776.66	2639.39	-1274.35	-2454.68	-4403.77
-2152.76	-7501.52	-2800.33	-9433.28	-5494.01	7938.63	803.674	5586.74	-4205.56	-3059.6	-4769.01	2288.7	425.271	-1672.01	-5469.05
2138.06	1882.25	1080.59	-3969.6	-5055.39	-388.004	-3332.37	-433.633	-9106.16	1257.96	-5311.05	705.287	-5516.95	2833.69	-3841.73
-2674.98	-1692.38	-3601.21	-4680.81	-8828.73	7216.45	-4182.03	-7852.61	-4179.28	3261.58	1390.3	-3903.53	759.365	-3533.94	-9449.66

2188.28	-319.08	-11.1406	-9750.94	-6113.32	1133.38	-1161.1	3006.19	-6961.47	5797.22	904.135	2718.66	-4797.21	1399.98	-10850.2
-6975.16	4104.57	216.449	-9433.05	-8770.11	-734.119	-2369.3	2645.14	-7660.9	-11006.7	-289.762	-2716.52	3714.35	-2658.54	-2311.88
2010.23	-3242.34	-68.9629	90.4766	-8645.95	-1949.66	-5778.33	4657.41	-9769.66	4262.89	2627.81	2370.09	384.059	-4924.31	-1274.32
-3107.17	-4060.5	2580.3	-4947.84	-11675.6	1700.73	-1262.1	-1054.73	-3929.55	8136.04	-5142.51	-2554.33	5554.82	4245.18	2636.35
-2826.91	1758.48	4449.75	-5589.96	-10454.4	110.369	-1028.41	2800.27	-9329.03	-3214.51	-2989.68	-462.874	-3954.55	1320.5	547.486
-4670.35	-7044.57	-1355.42	223.693	-5935.66	-5402.36	-5619.65	-3743.18	-7755.4	-1353.8	4912.14	-1090.84	6272.44	3909.92	-3500.22
-2760.64	-677.768	2389.15	-10344.9	-7985.03	-126.586	-4610.97	2266.27	-10391.5	1283.44	5608.33	4389.42	-1972.9	-1524.24	-6484.69
474.826	-7684.76	-520.727	-3821.58	-10188	3318.26	3213.44	-7972.63	-4115.48	-1151.92	5002.76	-1241.27	-3412.14	2527.42	-591.596
4232.57	-6792.37	2874.14	-8309.43	-11881.2	2751.3	-1762.39	-4460.78	-13578.2	-3396.42	2967.71	-1273.08	-3854.47	2851.6	-2578.13
2469.79	-1615.08	-449.592	-7863.96	-3595.69	2667.96	-4681.31	-6166.29	-11491.3	1942.24	5657.72	5.73828	2563.88	4213.08	-4861.32
-329.27	7604.21	-4180.89	-10499.6	-5859.88	806.318	-6429.84	5987.92	-14226.6	4413.59	1852.1	-67.5127	-2606.06	1796.54	-2630.23
1287.07	-9029	-1423.26	-3222.19	-9721.56	33.6582	-4236.64	1865.75	-5603.49	3916.94	-3113.09	-148.227	-1139.09	1095.02	-8068.66
5549.98	-3036.74	1707.09	-6823.25	-6547.83	-3727.25	-7724.45	-867.367	-10028.4	3473.8	87.5684	-1219.71	-43.1582	-1068.38	-2400.96
4167.7	-1115.4	3347.4	-15465	-8304.78	-4680.75	-2351.37	-1030.49	-10796.4	6913.46	-4958.69	951.08	-4687.69	-8436.52	-2685.13
1165.42	1205.15	1115.01	-2531.39	-1881.84	-2448.64	274.711	6219.84	-5281.53	-1476.63	-4643.47	-7721.29	-19.5352	6520.86	-1469.19
-350.996	120.16	11557.4	-7360.37	1147.02	-1578.3	-4681.97	-7087.85	-2979.02	3517.45	805.584	-5119.67	3122.1	-3368.38	-4543.68
2942.75	-3342	-852.713	-19203.1	-9403.73	2042.9	-5585.27	-5433.1	-4972.31	4259.34	7619.08	2100.21	4342.77	-6895.48	-3855.06
-5747.56	-362.668	1140.52	-15627.7	-3704.5	-959.123	-4156.46	1166.54	-11957.9	3948.52	-1837.8	3742.85	3621.15	4655.64	4337.74
629.66	-7569.28	2944.91	-2347.34	-1358.4	2381.39	-191.549	2990.83	-7157.5	2051.18	3423.32	4686.93	3411.21	4933.14	4489.05
1134.92	1271.31	1571.78	-17483.1	-3524.61	1252.5	1312.41	-3927.67	-4182.71	185.74	2040.41	4502.78	-2471.25	2451.13	-1296.43
-256.225	-677.738	2135.45	-7112.83	-9055.81	6092.6	-453.17	-1779.23	-4799.21	-4106.11	695.705	245.783	-532.768	-3240.22	-5760.38
5584.13	-11583.2	403.703	-7054.11	-8617.02	6340.95	300.412	-5203.93	-4250.98	-8371.82	-2726.26	-359.863	-2424.04	-4681.94	-1323.77
510.664	-8904.56	-4700.77	-4008.01	-7362.83	318.455	1434.75	703.74	-2494.39	1772.26	-4871.33	-2046.19	-3866.54	2136.44	-10226.9
-4954.79	-3552.34	-3948.22	-1629.66	-5244.31	-3918.69	-3466.88	1914.31	-2972	2299.37	1985.57	-3534.82	-2390.86	-4382.19	-99.2539
217.949	-5632.45	-884.107	-10323.8	-11184.5	1164.64	-3662.17	3752.26	-15667.1	-2557.26	784.094	7681.13	-3641.12	-1600.07	526.939
-5066.04	1167.64	-1884.48	-2837.78	-3503.02	-1590.4	-2207	9691.84	-8538.93	-2173.83	-998.223	510.875	760.033	5189.16	-1753.09
-3633.16	-938.043	-266.67	-8137.35	-5299.01	960.184	6387.56	-1640.62	-12727.5	2273.37	3008.06	1524.85	-2084.76	-387.377	1077.11
-5240.39	-2765.38	89.6797	-7455.74	-7042.78	2215.41	-5447.13	-4760.3	-9779.42	-1267.39	-7492.92	527.56	204.811	442.102	344.748

-460.254	-5364.79	6282.04	-8240.7	-3723.06	-6062.36	-56.9375	-1017.75	-10569.6	-5854.45	-1555.35	2499.38	3832.13	125.799	-5622.8
606.123	-6510.78	4838.16	-6357.97	-9964.7	6217.9	2069.86	-1933.92	-9191.75	3739.98	3860.51	2982.45	7452.49	-4239.06	-4941.99
2436.76	-5266.29	-3334	-12501.4	-7100.94	-5688.85	662.471	-4760.56	-3391.75	722.937	712.936	2957.22	-4603.26	629.588	-2989.47
-70.1758	365.4	-182.857	-7738.82	-5246.28	1918.38	-174.32	-5354.68	-8231.65	1752.65	3270.67	-2486.76	-1880.44	4824.88	-6368.46
-7316.17	-4613.28	-2759.44	-7424.26	-8165.68	8652.94	293.646	3649.07	-2796.52	7152.61	11293.1	-921.071	1853.81	-506.049	-5427.04
4310.2	-5145.49	-2191.99	-13031.4	-15483	-5689.48	-755.244	-9099.56	-4737.17	-2493.71	719.602	1232.25	-635.74	-4264.64	-6217.09
-4888.33	-369.957	-3080.37	-6045.42	-9726.32	-819.123	-2918	268.523	-7132.82	2180.76	3033.65	-3635.51	-1005.34	2675.85	-1187.43
-204.406	1986.13	4101.11	-4844.29	-6507.32	-1358.04	702.992	-6051.82	-3635.87	2982.77	2190.27	4488.59	1035.55	1661.75	-4814.52
5470.51	-3526.74	1504.46	-11530.1	-6082.48	6088.38	-1587.4	-885.275	-8555.62	5557.56	648.494	1041.58	1855.81	5373.02	-3109.1
5790.13	2188.21	2918.21	-6158.32	-7163.61	4516.25	-3245.29	329.525	-5757.66	-5242.84	818.076	998.178	3990.01	-1308.17	-5991.45
2525.36	29.7051	-4946.6	-7236.19	-7824.36	-1779.96	470.77	3026.19	-11937.7	-5459.76	9944.02	-4634.73	614.85	2788.99	-1081.15
194.287	-1853.3	80.8965	-3291.09	-3879.54	2584.68	-3300.77	-4913.47	-7428.72	2164.1	-420.42	-2823.5	3310.79	598.896	-906.071
-4164.46	-5094.8	5826.65	-10835.2	-13832.4	2990.7	-2324.83	-1724.08	-1772.7	-3020.93	3640.52	1060.86	7842.2	2486.12	140.496
4247.3	2733.33	82.7246	-11745	-7889.27	2979.66	1597.67	-4036.18	-4211.5	642.839	-4975.92	234.127	-1200.63	726.486	-82.0859
6162.26	-413.574	-4724.1	-6328.92	-5456.18	5032.81	-4731.25	-5030.74	-8182.8	-837.396	2245.16	-3139.88	-1289.79	2825.42	-3522.37
-5535.5	21.9902	1135.52	-7699.13	-4245.72	-1193.5	-1393.96	-1854.61	-9941.24	-4297.33	1260.05	-6188.56	-8610.48	1347.51	-4475.25
5554.82	-9212	-3105.53	-5455.62	-6918.97	-1573.76	-4281.31	-4601.52	-1655.57	2159.15	3973.3	-351.656	1335.58	6151.86	-6026.98
2777.46	-6642.17	-2176.09	-11706.6	-2013.99	297.959	-3040.96	-1792.87	-7612.94	814.371	8045.95	2432.22	-3487.34	3322.38	726.865
2802.27	-4085.44	2943.48	-6398.19	809.074	-2948.37	-5429.7	-6636.39	-4484.11	9487.77	6688.73	-1611.15	-3741.27	1653.46	-4249.7
1819.31	4086.95	7987.83	-10000.6	-5300.15	7159.18	1124.02	-2292.79	-4173.8	4435.34	-1406.57	2669.97	-7210.9	-2220.29	-6625.32
9844.8	-5946.82	2425.99	-10453.6	-10716.7	6210.27	-3197.89	-6541.33	-3296.92	-4680.49	-1820.37	-7355.71	1082.39	6880.19	-3128.57
-2706.38	-6869.1	321.283	-7100.43	-1282.37	4351.81	-4737.74	-5076.08	-1032.84	8415.45	1502.49	-1920.41	-2488.87	-1063.66	1812.17
-248.982	-2247.29	6948.47	-10208.2	-4189.06	-529.252	-1184.92	2048.15	-1907.54	2133.86	7281.71	-3769.99	1525.16	3320.73	-1378.03
158.951	-3016.15	-396.896	-6110.99	-11115.5	2251.41	-1271.06	-1804.77	-3428.43	2918.92	2175.96	834.609	-2801.27	-1926.41	-6939.07
1653.86	-955.965	1724.94	-16128.5	-3663.58	1030.35	-1102.06	-3548.12	-4045.76	110.443	3804	132.604	1726.33	591.646	3905.31
-1750.23	-327.797	-8283.76	-13187	-11361.8	-4674.68	523.465	-5124.2	-7235.87	-2929.79	474.172	3493.72	131.053	3110.12	101.971
-1982.54	-2046.33	2103.38	-10762.6	-6120.29	120.271	-5667.43	-2228.91	-9163.48	-1357.05	685.848	929.369	975.234	-1233.18	137.277
-261.25	-1459.93	5116.1	-4426.94	-11040.2	-1922.17	-8736.37	-4304.89	-6988.58	-390.933	4077.35	-2427.96	-3615.94	-3533.99	-6772.67

-258.191	-3601.46	83.7187	-10736.2	-10982.5	2627.22	-6001.76	-1211.75	-9432.24	1196.62	760.756	-7564.75	2453.8	552.768	-699.602
-817.492	-2060.7	-2523.7	-3533.66	-8041.62	276.91	-3574.21	-2943.65	-737.135	2497.94	4478.2	3847.75	1733.55	-2164.09	2598.53
2270.07	-5403.55	-3297.12	-7765.95	-9467.31	-409.803	-4136.55	-3331.74	-1332.07	3531.89	6628.29	3888.67	5785.05	6614.39	-4270.65
2936.72	-8460.31	-1799.16	-12813.9	-5525.23	4009.69	-7276.18	526.75	-1652.94	1848.25	1189.6	-732.724	-6898.91	5407.86	-9934.86
-1803.39	-1697.91	1854.05	-9440.24	-1063.16	-2344.72	-4514.41	-2118.41	-4206.32	145.304	6628.67	4220.57	2258.55	-7633.66	-3462.16
4077.08	-4606.47	-1722.9	-1963.05	-6445.26	253.059	-3223.14	-6435.4	-6068.93	-1189.99	3698.22	-133.662	-1911.54	3847.94	-4556.66
1074.21	-284.09	5467.17	-3610.87	-4379.31	5765.54	-1851.32	3907.06	-1087.29	8863.28	8349.03	4045.59	11962.6	-1145.9	2280.48
-2115.74	-2011.86	5046.92	-5407.39	-2931.97	2224.81	-3511.58	-3147.98	-3814.28	2980.26	-3096.62	258.138	-3956.77	-5407.14	-5942.22
728.459	-1820.02	3853.13	-7979.41	3542.15	-5631.19	-4308.72	4611.88	-6430.4	1516.42	4228.13	3523.05	4974.86	706.928	2391.79
6861.28	2681.24	1495.6	-2855.08	-3626.24	-2287.81	3435.72	325.137	-10239	-4403.94	4846.48	-2794.24	6903.86	1147.04	-3236.17
-4548.39	-3744.27	-8310.79	-6179.62	-15423.6	-7169.77	2725.87	-1633.07	-515.055	-2205.33	-6033.3	-6319.96	2501.34	1055.2	-4215.61
-4186.28	-7305.45	3110.19	-16253.4	-2605.94	-1871.92	-1068.79	-4305.65	-3291.45	-5045.12	-2148.46	1466.18	-1329.63	800.176	-4295.09
-549.656	-3179.8	4496.36	-11162.4	-14056.3	2623.06	-6931.49	-5024.55	-8542.36	-2442.82	3482.59	2073.95	-3713.24	3208.04	-7610.2
-3039.87	-6414.61	-328.664	-8318	-9106.4	1654.52	-5069.63	2255.8	-5182.62	3831.09	2423.62	-2293.92	-314.678	2780.65	753.414
838.121	-4347.9	2374.76	-1795.32	-8845.2	-309.102	2879.35	1228.64	-9663.22	5374.73	-1665	-3570.89	-3539.85	1293.82	-4917.5
3918.56	-3252.7	4037.05	-7464.58	1132.34	2497.93	-5345.41	-5777.75	-9052	227.174	-210.9	3459.65	798.207	1879.85	-5226.49
7634.79	-659.969	-2368.55	-5637.26	-5207.05	3629.82	-3016.24	4440.27	-7506.66	-2618.41	589.094	-4221.4	2225.19	-1841.92	-4327.42
7297.34	-6054.28	1045.9	-4057.52	-5950.88	3757.56	-3689.44	-4494.26	-5154.87	-2197.91	-4035.56	-4072.2	-954.877	4646.41	-8245.01
7181.9	-2622.38	165.502	-12139.4	-9615.7	4166.41	2941.84	1608.79	-2444.78	3971.49	2953.04	2233.3	513.246	1381.12	1177.71
-1071	-2830.17	3369.61	-1838.14	-9301.05	4388.17	-2670.5	-6002.94	-2470.18	1811.03	276.055	5509.06	6079.79	3468.72	2160.01
-3955.4	-6862.45	4104.56	-2696.65	-4529.67	-1920.04	1564.13	-1036.32	-5281.76	4780.07	1731.07	-1189.18	-2973.7	1666.94	-2638.53
-248.732	-1345.12	-361.768	-3422.56	-12341.2	3694.67	115.758	-4686.82	5357.77	314.029	-1682.1	-52.5645	1364.24	-1687.5	-9764.16
494.68	-5757.92	-3846	-828.982	-9273.17	1894.72	3144.91	5946.47	-4244.71	5308.11	4554.52	-3274.69	-3829.96	-778.412	-5492.38
-1397.64	-1736.58	1718.06	-10736.6	-5059.01	-1112.99	-4054.25	-1770.71	-9418.15	9338.4	6830.33	766.814	1824.86	4599.08	2165.99
-3543.54	-4564.59	6379.52	-8579.52	-8883.23	4645.07	-762.465	-10555.3	-5895.73	7023.39	-3914.68	1062.57	5244.05	-10.5527	-4977.14
769.977	613.92	-1813.19	-14951.4	-5889.6	2833.45	-7046.47	-5805.38	-3686.59	6932.29	1974.32	2008.52	3177.49	3580.95	-2932.67
975.379	-6401.19	-9871.65	-8645.49	-8874.53	-2078.31	1899.96	-6883.09	-7598.99	-1489.85	2796.33	4917.28	-1306.41	-294.181	-2435.3
316.717	-5585.31	-4533.62	-6200.03	-7589.81	-1099.54	-2113.68	-2223.16	-4461.59	-1611.01	2996.8	4065.66	-2255.56	2026.06	94.7793

-2622.25	633.359	2494.74	-9306.69	-6385.38	1718.56	-4229.56	1526.44	-6822.05	754.77	2418.19	1868.06	-1187.67	7058.49	-4486.39
-3399.57	-4628.43	-1152.31	-8817.77	-10596.6	3004.12	-8264.22	-5127.48	848.701	357.235	1367.29	588.162	4097.21	6691.24	-4021.33
-9105.79	-2475.79	4515.04	-8163.92	-5486.1	-1207.51	-8438.87	-4372.02	-4978.18	3642.35	-1681.27	926.031	2079.9	598.4	-5083.06
-3429.13	-2095.51	4154.32	-10412.3	-13022	-1052.5	2064.63	-1370.66	-9008.92	-4146.55	-943.258	-463.866	1618.36	-6345.9	-3756.82
4351.47	-1484.17	-182.5	-14439.1	-8953.17	1777.83	301.564	-51.4219	-9128.09	-1717.84	6343.03	1911.12	2275.92	576.393	-5194.12
1341.79	2734.49	3455.49	-10671.7	-8542.24	-2251.32	-9890.58	-875.18	295.988	-2301.79	3145.15	-1235.56	-3134.14	-270.53	-3525.93
-1594.06	-1032.45	3916.32	-6892.42	-3482.06	298.652	244.51	-1154.82	-8102.78	475.295	-1673.39	3451.06	-8045.08	3528.27	-3050.81
2810.17	-4738.27	752.461	-2092.38	-682.74	4173.79	-6164.9	905.953	-7595.89	-1203.02	1400.01	4664.7	-3897.2	4138.24	-3491.92
4746.11	-9518.57	-3084.43	-9529.58	-11023.6	3786.96	-5607.62	641.459	-4077.42	2016.13	-816.756	5598.79	3090.78	7092.73	1657.07
56.375	-1014.46	2063.36	-10794.6	-9784.66	-3696.1	1908.78	-2264.67	-5527.26	6907.88	3143.46	-10232.4	4216.36	-7085.39	-1953.1
2960.95	-2296.89	18.9824	-12438.5	-3769.04	-768.121	-1116.74	-3160.94	-2857.31	3395.18	3126.73	-2195.74	-3405.33	-2254.91	2319.9
-2538.28	-6921.28	-5003.15	-9403.02	-7502.97	5155.42	2263.31	5104.43	-5921.03	2423.89	-999.82	1970.15	5007.37	-1200.48	-4758.34
-2962.11	-1387.46	-1456.08	-5968.64	-3720.07	2059.35	-908.934	-4020.7	-3974.58	1758.48	1924.75	-4449.27	-3368.93	-2203.88	-3940.55
1078.52	-2662.78	9418.98	-7566.8	-5128.91	897.977	4023.41	-4069.38	-2654.99	2491.36	4998.32	1454.32	-985.127	-5537.13	754.307
7805.91	-4375.24	2624.29	-4760.1	-6948.71	-203.215	-2750.75	-4574.47	-8145.73	-6944.17	-3154.85	2484.32	8947.57	-4379.06	-3761.98
-1537.93	-843.215	7143.99	-8794.33	-7442.29	-7008.3	-4445.98	-4722.31	-2287.64	-1264.18	2543.07	847.844	419.16	1662.69	-4026.25
7122.03	-6857.1	-962.236	-5182.36	-3646.57	3112.34	-8364.66	-4069.06	-5801.56	-1958.14	4666.08	167.865	3376.48	-4635.76	-8838.3
425.225	2211.9	-1919.57	-14510.4	-7603.69	-3586.61	9704.56	-2751.35	-4824.18	-895.817	3811.24	666.396	2997.62	-4401.33	-6199.33
2806.86	2854.1	5175.04	-2776.46	-3548.49	3676.55	-244.383	2202.49	-6428.58	-4355.78	-1414.48	2055.17	1419.86	4830.14	1804.44
-159.828	1609.83	-4477.47	-4192.49	-11071.4	1860.17	-2810.13	-1306.47	-12907.5	-2772.37	6509.49	-373.436	869.004	177.533	-9167.43
5233.92	-2103.25	-4111.31	-15581.4	-3500.01	406.482	993.979	2329.12	-6460.28	-4661.88	8095.84	-3768.44	-4552.76	-2090.19	-6845.28
-714.26	148.256	-2100.67	-4763.36	-5915.7	6534.12	-4880.54	-1356.21	-7234.12	-791.568	-561.723	1586.35	-2964.22	1934.51	-8347.21
-3519.63	-2815.04	-4441.83	-7582.89	-7231.42	-111.166	-4534.11	-1208.41	-134.254	-5766	-1484.14	1830.45	1754.25	-1125.64	-11.3086
-2926.96	-1636.19	4754.23	-20169	-10649.5	-8196.58	-1800.23	-9638.31	946.631	-2784.33	-1689.66	926.889	5984.92	1105.45	-3148.87
1228.23	-4560.58	703.145	-11140.6	-11856.7	-4435.81	-1252.09	-1512.19	-3671.13	-2414.9	967.234	-1537.83	559.086	-3209.76	-10072.1
1902.69	-4739.45	6946.88	-5831.27	-5064.86	-1412.1	-6484	1317.09	-6131.04	-4438.1	-556.982	-2841.83	-4162.01	-134.701	-3772.31
776.437	-7838.85	3480.96	-6342.67	-7459.94	1601.97	-3691.39	2258.28	-6025.45	-3546.31	4928.46	492.117	-4156.79	-413.192	-3934.14
-2801.28	-1431.93	1976.21	-8913.11	-3867.53	2136.45	3587.46	-5359.56	-7055.05	-2403.71	-3842.59	866.105	5304.77	1471.06	-5587.19

-5701.17	-1643.06	-6945.08	-14598.3	-3119.99	6296.03	427.318	-2509.59	-1505.96	-1919.1	4935.15	-4246.34	4310.82	-24.6074	-2427.16
-393.139	-1427.67	-892.732	-13306.7	-4262.51	-81.1582	-2792.24	1900.99	-5367.29	2347.27	7150.9	2978.66	2290.18	6238.17	-2737.58
-2408.83	-3438.62	5300.97	-7437.25	-7754.6	-1410.1	-3234.27	2476.54	-8631.65	-857.56	2053.05	-5998.82	-666.51	-3431.36	3674.8
515.158	1959.13	-3424.56	-8514.94	5546.74	4325.99	-9778.12	80.4941	-2436.93	-3284.01	4830.72	-1582.72	-1335.37	-1803.43	-10384
-526.9	-2729.2	-1116.39	-9711.22	-3339.22	1636.69	-9994.82	-5332.44	-8677	842.585	3687.4	-1329.06	-1402.2	934.781	-1228.14
4141.6	3614.85	-1725.16	-9292.71	-5637.32	-2447.92	-2175.58	-2483.09	-3269.18	3102.08	935.33	7541.87	-612.82	719.184	-6018.7
2901.32	-3229.89	-483.41	-5103.41	-11396.6	-7042.66	-6689.23	701.857	-4546.29	-2468.37	4273.26	1375.05	1609.25	3564.15	-5581.08
2619.75	-4562.79	2258.61	-10137.9	-10258.1	2629.61	-7936.79	1243.69	-7254.6	4767	5892.97	8565.13	-4827.46	-5578.8	-7254.06
1804.92	-12531.6	3097.55	-9161.97	-5598.59	2361.04	-4302.31	-1399.4	-2527.77	300.439	4694.76	-3859.93	1578.59	-3570.73	-7447.25
7657.64	-4831.61	5962.64	-7352.99	-3037.45	6085.5	-6910.05	-273.588	-5890.85	-5308.13	2275.19	80.9736	824.09	-1318.13	-3250.31
-3413.24	-1299.46	1077.97	-8890.36	-5409.67	2394.87	-4216.73	529.994	-2023.48	-3141.76	-298.885	5494.16	-1384.8	-1123.5	-3782.93
-123.213	-675.277	3412.18	-14605	-813.871	-1135.9	-3191.86	-1831.83	-4030.55	-5159.31	2617.6	-5199.03	-1920.47	2624.37	-4876.76
-7000.48	-4082.79	12509.7	-5803.91	-7225.73	5100.23	-4573.48	1500.25	-8519.87	-2741.2	2611.75	685.627	1100.53	-5629.07	-1029.08
1376.85	3760.19	5715.98	-5660.17	-9337.01	-2924.49	-5027.29	-5084.41	-3797.06	-2202.64	8503.05	-557.296	-123.914	632.039	-3719.43
-3482.85	-1556.1	2772.64	-9189.23	-6575.26	-1908.47	-4637.09	-3657.96	-3094.93	3060.17	2850.56	4201.25	-692.762	-23.1992	-7052.1
-4195.17	-4976.58	-181.904	-4718.3	-3696.64	7783.07	-2940.91	-4102.93	-8863.2	-2392.75	4154.72	2413.85	10321	2696.37	-6005.78
1309.24	1335.88	3093.98	-6972.56	-7258.03	2360.18	2039.14	3706.21	-5786.19	-468.852	5034.64	-1937	-679.395	-2570.62	-5366.31
-5016.91	-181.242	-1434.59	-12295.5	-9978.97	121.518	-7469.24	-10.416	-1366.44	-1584.16	2270.15	-4040.9	-5547.53	648.283	-6825.9
-157.863	-2749.55	-2881.08	-13319.7	-13154.3	-5103.36	-6080.33	889.346	-3427.54	4433.68	566.033	4845.81	3965.98	-3206.84	-5933.22
-5039.1	-6524.5	1824.05	-9847.6	-1016.8	632.262	-3571.98	1272.1	-8147.67	284.635	4456.15	-888.876	-527.213	-2252.54	-6523.56
-2561.07	-5308.27	-5642.49	-11555.5	-8617.73	-230.561	-2767.17	2199.26	-5644.36	4591.62	6943.71	-5430.6	-4321.64	-1179.07	1904.28
-629.352	-10502.8	4953.85	-7789.36	-8188.24	-74.6699	-10335.5	-3557.14	-5587.06	-2631.38	-755.074	348.523	1378.5	96.5937	-6916.9
-2584.45	579.629	4835.62	-6850.09	-11722.7	845.977	-6010.38	-2689.21	-6707.1	2970.29	-2608.83	6815.49	3806.92	-3345.18	-6502.21
-5820.23	-1208.92	3638.69	-9593.83	-5680.31	2649.05	-4980.77	2652.93	-10535.7	1415.64	7721.68	-2451.05	453.152	4968.89	-5462.15
-1586.63	-1145.41	-3150.91	-15740.6	-5196.16	-3233.5	-2687.97	-1424.5	-1155.54	1201.6	2509.52	-3946.55	2071.9	-4445.12	-3021.29
386.236	-2245.5	2568.58	-8226.56	-3285.64	-3668.2	-2066.63	1111.67	-8863.16	5815.36	501.82	5587.74	1209.98	-1252.55	-3376.56
-1672.44	-5645.84	2482.94	-8307.19	-4014.94	-2294.8	-3575.57	-8086.47	-6621.18	1368.89	-864.092	1276.23	5947.3	3904.6	-7416.3
4340.76	-5298.3	2123.93	-6871.02	-11879.8	845.09	-3567.31	-1506.74	-414.584	-8693.9	3259.69	-2672.54	1183.84	-6821.55	-6025.79

4810.5	-4606.47	6377.01	-4197.99	-6577.11	5292.71	1838.06	-286.449	-4964.69	640.809	1859.29	-991.03	672.426	-5316.13	-10395.4
-4770.93	-4962.94	-3895.68	-7344.32	-9722.58	-1180.05	2528.45	-6429.49	4437.62	-2512.87	-3972.16	1469.61	8245.02	2427.06	-5135.38
941.602	-9829.24	-6505.04	-5931.65	-8005.64	-934.467	-8437.86	1749.34	-7637.97	-2855.16	2910.14	189.08	1267.34	787.92	2158.28
-3031.5	-8076.99	-145.502	-15219.7	-10516	1021.59	-3594.2	3145.96	-6853.88	2746.65	1572.08	-2738.91	3320.05	-7937.06	-2969.44
2671.38	-11608.7	3394.73	-9351.59	-3081.11	-3332.23	-1203.41	1108.01	-1309.7	2336.19	2819.27	1293.82	3237.55	-195.979	-3444.29
-166.197	-9276.76	2318.97	-6135.71	-6189.3	336.039	-2079.12	-64.1035	-3816.15	1408	6595.24	-1197.77	-3063.38	1412.29	1776.22
2876.79	-5310.45	1576.03	-8372.3	-3254.54	-2782.25	-5160.51	1240.62	-2003.29	-478.121	1610.77	5368.53	3550.1	-3700.02	-11942.8
-267.119	-5814.48	-721.691	-9194.21	-107.377	-2350.3	814.449	-1625.01	-6675.34	-3428.03	3142.92	-1774.04	3732.68	-4968.41	-2272.79
-5036.29	-1199.87	1448.86	-14657.7	-1802.3	-470.484	-2874.76	-3898.96	-8281.29	3402.13	3181.54	-806.516	-2026.7	-4042.01	-6520.26
-1226.74	-240.168	2216.4	-8627.13	-8238.92	-1161.66	-244.627	-711.469	-9129.94	748.528	4073.37	-14.5752	-3912.75	-1984.53	1327.13
1011.34	1119.44	1773.34	-4597.67	-421.373	353.344	-627.119	4048.23	-4152.39	-1249.96	2618.64	-2605.08	2797.52	-3779.42	814.893
-1542.89	-3073.01	-1608.73	-6584.41	-7607.13	-3713.49	-3612.23	-3943.57	-4148.63	-864.835	5009.57	-6454.58	1661.94	-309.123	2525.34
-3696.87	2158.93	-4675.26	-5104.59	-3998.11	4543.43	801.336	-2273.7	-5590.86	3801.51	3657.73	-3439.43	867.633	-2749.45	-13096.3
-328.178	-3553.83	-3541.28	-6578.84	-4860.31	2989.79	-1912.66	-1411.78	-3973.46	281.754	1146.62	1252.4	-2617.98	-5373.26	-3861.77
2940.41	-7694.45	-853.27	-13324.2	-3359.53	3753.85	-302.656	-7686.05	-6895.27	-1109.89	-239.865	-4125.79	5871.69	2018.87	2105.21
443.518	-3685.17	-602.709	-10941.9	-9037.13	-5217.76	-1162.32	1715.75	-9089.47	-2220.81	3477.8	-1145.6	-446.977	-1705.18	-6397.23
5697.17	-10925.3	-952.66	-13033.5	-8888.98	3396.42	-3821.57	-3165.35	672.863	-2611.88	-2075.85	-3654.06	-3111.93	-3629.85	-10207.3
-897.623	-2719.21	859.6	-7830.99	-6727.02	-2624.8	317.012	-12279.2	-6653.2	1728.39	-603.205	-3177.96	124.203	-7165.23	-3774.71
-1966.37	1079.86	725.24	-9015.53	-1108.01	-3865.3	6282.07	-7655.04	-8625.55	829.291	6264.57	-4300.65	-7762.49	-7792.07	906.354
-1221.02	3208.5	6861.12	-8244.26	-9143.48	-902.24	-6384.85	4404.28	-622.977	-2414.87	3213.62	221.907	-5872.63	-3954.7	-7473.91
-7345.57	-3338.2	-1279.04	-15134.5	-2339.39	-564.084	-2045.01	-6245.32	-7623.59	724.119	2603.81	-725.284	-58.043	-1608.7	-2490.34
-1861.27	-8062.1	1118.15	-6933.1	-5626.95	-3810.33	-2197.58	-6154.77	-12001.4	-2006.62	-2699.09	-905.356	-7271.69	7150.07	-3871.91
-4554.19	2315.9	-5555.06	-3217.31	-7671.34	-8555.03	107.283	-2197.36	-9726.91	-768.009	-2258.87	-26.5469	4497.47	1372.68	-2244.05
-3347.62	-6995.39	-3331.17	-10349.9	1556.37	4098.88	-3769.47	6453.81	-7443.86	1821.44	4571.3	-4521.19	145.42	-159.473	-3784.69
4557.75	-7831.17	927.477	-12845.5	-6402.68	1143	-139.412	-83.5156	-6381.55	-3431.5	-87.8691	-5750.03	3111.77	-2470.43	-5275.72
-220.92	-5157.28	-2539.12	-6935.6	-11267.2	-1133.71	-3474.3	2756.94	-4866.93	138.177	370.832	-3916.34	3324.34	-4891.17	-5895.4
-3229.13	-6375.37	1733.76	-9305.37	-5934.16	1490.15	-7994.68	218.562	-2169.1	-1086	-2496.74	-1150.25	-1711.08	-2327.38	-371.473
1835.23	-6411.27	-359.342	-9356.31	-11921.7	2611.17	1199.14	-17236.5	-13311.8	-2510.92	-703.986	-5418.08	3032.57	951.418	3128.53

-0.158203	-6251.93	3415	-11355.7	-3531.27	864.67	-1305.9	-5576.18	-10499.6	-3062.04	6429.51	664.299	-161.793	-2991.12	-4762.91
-2530.2	-2038.83	-2496.36	-8404.9	-9790.82	780.471	-941.818	834.916	-1176.98	-6012.94	-2004.42	-616.027	2431.36	-3258.84	-5979.18
-1627.11	-4181.87	-4603.33	-9228.99	-1206.59	4446.3	-2183.84	-5324.13	-8653.42	-349.057	4624.77	-8472.26	791.432	-1101.16	-8699.1
-586.898	-4754.27	371.078	-13883.4	-3896.72	4046.94	250.684	-5840.24	-10635.9	364.658	7005.19	714.418	-6447.69	-2284.22	-2924.5
1922.38	-5442.61	849.348	-9015.9	-7684.17	-8586.21	-154.617	-4871.5	-5441.18	1388.05	-1472.84	-4792.58	-1455.74	550.941	-7983.18
-1583.85	3705.18	1068.59	-5835.62	-3095.05	3040.32	5488.94	-4039.57	-1814.36	-2184.91	2749.27	-3141.26	625.105	1940.53	-4512.1
2618	1050.15	1989.33	-5824.87	-11819	1842.53	2684.47	-932.854	-9894.67	-3155.32	3642.12	1687.09	1872.88	-340.439	-5670.95
981.154	-9139.87	-2138.18	-4274.28	-10942.4	-5794.43	-6128.67	-2683.74	-5732.32	3238.72	4983.62	-3159.86	538.5	-2023.46	-7104.74
7119.78	-4858.67	3149.49	-8462.26	-4572.09	3600.79	-9806.54	2078.46	-4131.94	4219.72	2313.33	2343.05	-304.014	-171.207	-5093.69
-6015.92	-6814.61	3102.56	-8043.46	-8090.33	-3159.12	2516.87	-3489.02	-9126.04	-1873.68	1081.85	-4383.33	-2727.29	-1879.63	-4565.01
935.234	-13178	6570.71	-4662.32	-5294.6	777.559	-2685.11	-986.102	-9321.97	4263.44	8616.61	-2000.7	-3300.36	-39.7051	-3383.73
-492.813	-4662.6	2987.2	-3383.6	-5709.28	-4445.88	-2096.1	-6740.64	-7650.9	103.611	323.545	788.098	885.666	1648.25	-109.799
-8554.79	1431.89	-4354.24	-7385.98	-9475.95	-5007.21	-4060.74	-7750.12	-5239.54	-688.908	-943.258	-6001.93	-3717.21	-8615.81	-10502.3
1677.8	-3.67578	-1829	-10008.9	-8861.95	619.727	-1212.82	-6077.81	-5835.79	-428.193	-415.732	-263.728	-4449.08	3055.75	-12190.6
4898.85	-815.574	-3218.67	-11972.5	-5978.96	-4254.32	-2340.11	-2964.68	-6783.99	-6795.97	-968.219	9364.37	-3451.57	-4881.58	-5896.69
-4204.6	-6765.62	-4075.54	-1734.86	-7252.56	2031.01	-4507.01	-2927.02	2711.01	-6400.1	3959.95	376.232	-6023.92	-4178.93	-4463.03
664.996	-7096.54	7859.63	-5921.17	-4921.29	-808.641	-5250.1	974.646	-1401	708.646	3277.51	596.64	1232.46	2942.27	-2882.92
-1639.51	-8272.59	-3772.29	-12518.3	-7883.6	-224.293	256.193	-4748.3	-6850.71	3688.28	4018.61	-8652.67	-2040.32	-1543.48	-1053.68
2212.91	3090.23	-1964.6	-10424.8	-12136.1	858.262	-4719.11	-6151.01	513.584	770.943	-1465.62	-6830.64	527.502	590.447	-8184.16
-1481.3	-8079.93	-886.096	-7735.13	-6595.77	4922.74	-3202.64	-3024.34	-12430.8	1879.05	1099.72	1827.82	-139.244	3091.16	-2973.6
1121.69	-3887.46	-888.559	-3343.61	-12914.6	-6043.34	2093.54	-6946.78	-2758.46	-3948.76	3180.76	-5928.47	-2487.61	5257.71	-1568.76
441.02	3693.79	2370.67	-9459.19	-2527.13	-1023.6	-5653.48	-2901.9	-4220.27	-2629.64	3461.79	1472.65	2650.45	7131.3	-7213.05
2302.13	-4018.64	-2897.09	-7800.6	-5953.53	-585.051	-3155.07	1529.3	-4478.24	-855.049	5406.49	-3314.57	1299.85	4702.87	-5276.02
-1225.89	-4262.15	1131.38	-11460.9	-5935.64	3188.66	-2585.27	2102.16	-2310.01	7337.14	-1149.74	260.775	8201.58	-5537.62	-819.477
-305.547	-5162.11	-2782.64	-9399.92	-903.514	-4979.45	-6273.88	-1896.98	4554.02	1217.11	1139.45	1757.27	4379.58	-2713.41	9837.79
483.287	-574.496	-1052.88	-5172.18	-6184.61	-2393.23	4246.04	-3769.08	5019.19	-5976.64	734.35	-4655.68	-4249.77	6850.41	1544.5
218.605	-2738.53	1044.55	-6178.73	-3511.07	2419.28	-1974.32	-762.113	-6421.1	-2265.23	-7427.27	-3400.15	-9893.47	24.4414	2080.58
3882.29	-166.998	-1586.23	-2747.22	-5616.24	-459.723	-8741.36	-4205.14	-10627.5	3017.04	385.605	-3320.9	-6970.26	-3834.7	-7713.09

-5586.79	-2469.5	-3267.79	-14066.7	-6975.74	4093.03	-1270.4	-7396.1	-9516.74	-292.63	3808.81	-3105.57	-5570.34	-419.867	305.006	
2428.24	-12643	2391.81	-15260.1	-7511.93	272.361	220.133	1294.45	-8111.72	-978.869	1068.63	-5132.93	-5999.44	738.271	-4655.31	
-3347.22	-1874.57	6705.34	-7091.23	-12743.1	-6594.27	-4313.33	-2848.77	-6007.91	316.651	1745.26	-282.328	-4084.33	2676.87	-2218.5	
627.318	-4269.59	2012.14	-12771.6	-7380.01	2375.63	-7126.99	-1839.05	-3763.58	-1273.82	-3068.16	-1049.2	-4438.16	1730.72	-6885.81	
-307.482	-4637.97	-7665.05	-6607.42	350.25	6346.45	-7898.15	3672.69	-7685.83	-2905.35	-1816.79	-2870.24	1701.52	-1631.93	-7741.24	
-6448.8	-5922.46	-4815.46	-6771.53	-8308.82	-1233.82	1973.26	-2065.16	-13095.8	-5014.33	139.086	1675.52	5535.38	3857.17	-4532.16	
-2683.59	-810.289	-1463.87	-10160.6	-5229.57	42.4492	-3156.71	-6217.64	-10966.9	3371.07	1643.92	-3695.43	5149.48	-2562.48	-3630.37	
2600.24	-3020.85	2023.94	-10559.4	-8785.21	1627.34	-3970.66	-4528.98	-6097	-5030.43	707.234	2021.3	4603.22	-4617.14	-796.605	
5473.6	513.564	-5293.45	-3921.97	-10518.7	-1184.57	1638.58	2438.32	-6189.02	-5513.6	2323.44	-426.282	-3659.22	1719.43	-7971.71	
-1006.04	-5043.53	-3687.72	-8415.29	-9115.85	178.346	-4267.96	-4545.24	-10207.9	-8703.06	-1197.68	-6476.38	3281.24	2817.75	-1331.91	
-755.434	-8042.63	3214.19	-7275.91	-9175.11	-1496.29	-1189.91	-5075.4	-7883.11	-1429.03	-4923.84	-3564.89	3878.79	311.824	-1084.96	
357.703	-3221.81	-610.379	-10744.9	-8819.87	5228.09	-22.9004	-5041.04	-8349.73	4663.75	-738.781	1051.14	-3035.54	6725.5	-2746.4	
-1877	1805.68	4523.9	-13512.2	-6260.26	1543.73	-1043.44	2834.41	-2035.32	-2535.77	-2183.63	-3069.54	1537.71	6108.55	2390.57	
3408.3	-7786.25	-17.0234	-11319.8	-8931.24	-1379.81	-7155.77	-8187.6	-3352.56	2690.29	3071.64	-3906.73	3905.15	1586.16	-3029.21	
-2484.34	-6287.65	-2775.77	-13139.5	-2356.75	2177.38	3936.44	-1177.68	-5677.67	1402.83	-6172.63	2396.42	3843.72	5353.15	-2029.01	
-1800.83	-2199.24	-444.426	-7270.41	-3147.76	7923.1	137.727	1124.19	2528.24	4023.52	1936.25	-753.195	5231.53	2829.96	516.936	
-6489.22	2222.08	-2887.8	-11006	-4733.37	2552.21	1115.79	-3520.96	-3712.72	2235.86	4504.35	2617.46	8591.26	5198.31	28.0273	
497.553	-6307.36	-2585.29	-5091.08	-13310.5	-318.17	-8065.65	-97.6582	-2389.6	3542.08	161.514	-4440.01	3397.29	-2842.8	-4573.18	
-5164.09	-1703.2	-2610.01	-20195	-4257.04	-2188.25	-6926.19	-7331.21	-5896.42	1994.93	2639.06	2679.64	6093.86	4383.12	-3793.91	
962.486	-587.162	2327	-5397.56	-10006	-3422.4	-4276.43	-4683.51	-3428.59	-2394.85	948.254	-7934.55	-135.023	-4597.66	-6567.6	
6394.79	-1513.21	2527.84	-5746.41	-1619.09	-2447.42	-89.2988	-4817.79	-10980	361.593	4230.84	1127.97	5095.42	-4028.94	-2996.61	
-6225.16	-3130.69	-2100.03	-6973.81	-7442.6	3280.49	-4090.48	-5786.13	-6773.6	-1890.67	1939.47	-4770.1	2357.27	2276.7	-2443.3	
-3472.61	1222.53	-216.357	-7991.18	-3334.45	1025.08	-7296.72	-9768.5	-8800.3	6823.95	-1845.58	-4189.09	-2878.88	1317.65	-5065.27	
7045.18	1690.74	7102.9	-2711.55	-5034.76	1181.69	-4828.16	2199.76	-8750.06	-2604.16	-2384	3155.31	1670.31	-934.816	-8193.86	
302.795	2992.7	3548.05	-3344.23	-8021.52	-2901.95	-2486.2	-2811.61	1009.2	-4102.05	-3734.41	1490.04	-1688.38	-729.677	-1657.01	
469.645	3646.53	5669.21	-13365	-5795.87	6234.84	-1546.67	569.801	100.559	264.472	-2359.29	-7559.21	-252.176	2461.37	-7956.38	
-1455.77	-982.936	4794.13	-12786.9	-5884.6	-2120.52	3282.49	-4314.56	-7146.68	-3412.82	4354.56	-1073.53	4771.17	-860.135	1682.92	
-659.256	-3165.5	-4071.25	-14608.9	-13486.7	-166.912	-2068.34	-2788.99	-3565.31	-1359.05	2518.54	3075.95	-401.248	-1483.06	-543.445	

-4259.07	-6661.31	-7052.38	-16358.7	-9052.98	1501.85	-9861.33	-5131.19	-6806.25	-3606.21	-41.748	-4257.12	-3942.7	-4445.71	-5479.86
-193.887	-8260.32	-1661.26	-11864	-11830.2	2901.21	-2677.79	-730.4	-12917.2	2218.2	-396.371	-5339.02	1329.14	4014.93	-6505.45
-139.914	-1073.3	2015	-6138.35	-6709.78	2702.24	314.328	-7142.96	-11368.3	4196.31	2430.13	-2937.22	5211.83	-1257.32	-9617.22
-2386.17	-5756.5	-5988.03	-931.805	-4619.39	212.846	23.168	-6774.05	-8562.65	1662.99	1986.43	-1574.29	3845.02	1639.15	1603.76
-399.383	7824.12	3709.99	-11967	-261.457	-5019.08	237.814	-2414.88	-8916.23	499.927	1420.03	1091.65	-4016.05	-3209.19	-5300.46
-4749.51	979.625	-6463.79	-6886.75	-5653.72	865.807	-1273.08	-8390.15	-2546.24	26.5049	969.16	-1419.35	-433.201	-798.309	-5276.99
-1892.92	-2199.57	5402.39	-7695.17	-7687.38	1152.02	-4923.65	-1915.53	-4214.17	-3613.71	2725.12	2199.28	-2452.44	108.016	-5222.35
-876.178	-3793.78	1226.48	-9848.36	-4821.99	6922.75	-2177.04	-1590.3	600.926	674.339	1988.61	4488.57	-677.359	-8781.71	-5696.93
2760.38	-4891.17	2889.87	-6021.75	-11485.6	-2238.75	-1463.42	4317.23	-3339.27	1571.81	-6005.25	8550.7	1936.86	-28.2324	-4550.68
-4833.83	-1608.13	4451.29	-7558.2	-6549.64	-2446.09	1969.11	4229.69	-5537.59	-1931.29	5617.58	-7048.79	-2275.04	-515.54	-1177.4
-3629.41	760.445	-1805.92	-3975.63	-12212.8	-6535.61	-6129.89	-5067.52	-9412.87	1125.2	-3001.39	-1445.15	1279.19	1656.3	-4182.27
-981.096	-1377.4	-2968.71	-8775.18	-10200.7	-3637.14	-2519.43	-4875.07	-10686.2	5291.05	-2445.3	-7859	1794.35	-1384.86	-1775.1
-3971.42	-9128.11	-840.195	-7589.85	-2962.28	-390.611	-1171.88	4868.98	-5479.17	7565.72	1106.66	-9295.93	4903.06	1569.24	-3349.96
1526.25	-5739.49	454.412	-10666.3	-7837.11	-413.99	4042.21	1170.31	-1105.53	5839.4	4353.27	-205.447	-2552.26	-3296.26	-9250.21
-1165.71	-4418.21	6587.47	-11093.4	-4185.24	1336.2	1203.32	615.676	-3580.19	-1035.92	5281.03	-1126.39	2779.45	-3283.82	-4768.07
3466.83	-3998.23	5444.24	-8036.93	-3082.51	-437.758	2622.16	-5317.94	-2548.19	1327.49	3724.12	-7290.08	4623.55	5368.8	-3393.11
779.402	-7647.86	1108.21	-6544.56	10.0117	-299.533	-4227.4	-1378.07	-6630.25	-1016.13	6882.05	944.699	-1366.88	-2100.83	851.336
870.055	-3059.64	2192.52	-6191.62	-4282.39	-1133.37	2241.23	-10583.2	-11570.5	-482.257	2052.7	2955.17	-2128.71	-2896.88	-9844.43
3963.7	-1471.5	2704.17	-13422	-7794.04	-449.518	-3999.57	-6028.69	-6897.75	1280.59	-3322.18	-363.516	129.129	-2854.79	-1370.11
1386.5	133.129	847.52	-9615.25	284.166	-1567.9	-3360.75	-9021.35	-7925.43	3409.11	-1070.62	-1522.93	-3069.23	-996.304	1083.6
2463.48	-2455.96	-2264.91	-9645.85	-2860.09	-7109.06	-8385.41	-7658.95	-3800.72	-4630.38	-722.891	-9287.2	12127.8	-244.115	-2369.12
-1301.34	-6681.05	-4278.37	-11306.8	-10474.3	-5116.98	-3840.47	-622.904	-9736.54	-3194.2	-1502.36	-6955.5	-276.096	-6352.68	8484.59
-2997.85	-4129.21	6333.22	-9163.75	-7891.24	-2862.95	-2686.16	-3448.82	-1337.08	-509.731	4799.55	-5689.06	2953	-599.4	-173.033
-958.838	3113.95	2888.92	-11500.1	-7471.12	410.947	2698.42	-1218.12	-11867.7	-3203.95	2757.15	-3249.57	5450.77	-4460.24	-4617.87
-4057.7	-5728.58	2324.49	-12590.1	-10541.1	194.764	841.824	-8004.38	-3930.41	-1970.4	5091.05	-6192.69	229.213	-1303.79	-8043.17
4377.38	-8504.41	-2074.96	-11531.1	-11246.8	236.586	-1638.63	-3705.04	-12528	-4252.86	-33.0293	6649.42	329.889	-3443.43	-2999.58
-4490.26	-1763.28	6552.07	-13962.2	-6895.63	4198.59	-1783.07	4976.76	-999.357	2383.97	1323.51	7697.59	6558.36	484.709	-4474.52
-4235.63	-4302.52	-2483.84	-10524.4	-7873.68	1871.12	236.42	-4353.85	-1060.61	931.878	9911.81	-8000.07	732.15	-2760.89	1785.22

6310.47	-1600.5	-3198.44	-10412.6	-8981.96	251.201	-6396.32	-3313.66	-3163.44	-5642.7	2140.45	-3870.03	-1767.4	-3115.79	-5054.11
3591.39	1714.01	-7094.74	-10951.7	-4767.01	2123.22	3770.65	-2407.7	-3221.45	-345.732	-1862.91	1858.61	-6703.81	380.014	-6270.05
432.779	-176.898	2599.49	-8168.4	-10477.5	-1182.79	-7493.48	-518.332	-6958.83	4280.58	7681.8	-8447.55	-318.529	6691.45	-947.76
-3867.41	-2506.38	-1537.71	-11250.6	-9894.35	4174.71	-1172.68	1375.65	-4196.99	-2407.26	4813.41	615.172	193.223	-3610.24	-4613.65
4972.86	-7774.03	-558.598	-7365.71	-4873.18	-4141.2	-5090.9	3482.42	-4488.66	2637.82	1190.85	-3271.39	2531.48	464.266	-6575.15
-602.412	-9691.52	-1561.63	-12076.2	-8008.14	3804.21	-3982.69	128.354	-1967.4	-3515	1127.85	-2012.28	-143.873	1869.08	-1753.52
-4196.11	-2834.48	-987.154	-4671.75	-10004.2	-4957.98	-1533.38	-5706.7	-8660.44	5010.98	1758.69	1643.62	-3995.28	2369.48	-912.344
-4691.33	-1141.15	3931.66	-12115.1	-4984.22	-602.689	-4669.25	-2217.19	-5052.53	-1534.9	3149.84	-3829.78	-3167.32	-1611.07	-2222.29
-5224.59	-2997.77	1806.13	-3341.99	-2417.79	-4512.86	-3098.61	2079.55	-12504.7	-2659.22	6615.54	-4721.2	-444.939	3721.08	-5218.94
-3034.59	-6419.99	2650.08	-3065.93	-1157.1	-556.486	3865.68	1388.45	-2327.16	-1108.34	2240.93	-4316.39	4090.95	1429.29	-4140.75
-6395.82	-301.313	-457.381	-4888.24	3561.18	2935.43	-4439.99	-8404.06	-3814.77	1309.73	577.768	-2007.86	3189.67	-655.777	-7487.18
5898.7	-4424.99	3084.64	-12510	-10445.1	2589.31	-7813.2	3608.08	-4755.83	2687.75	4221.97	386.829	1040.92	-6802.12	-3175.64
7201.94	1668.39	-2634.25	-13576.6	-6254.72	-2773.15	-5795.94	-4007.72	-12517.2	2231.9	725.049	2342.87	-4074.44	992.383	-7620.55
2153.51	1153.97	357.525	-6220.93	-3800.49	-496.998	-5264.33	-2958.37	-7988	1538.39	-2837.2	1940.66	-3507.26	-6512.19	-8848.79
366.414	4028.82	1778.19	-1352.85	-9102.8	-896.416	-1993.5	-1827.63	-1204.85	-6554.53	-557.871	-3065.71	2330.09	-5681.09	-3514.58
1739.05	-10346.1	-694.867	-8040.49	-8493.23	-4845.16	-9388.16	1864.69	-13864.8	-958.706	1084.54	-3089.54	2781.38	-5139.44	-4236.04
2174.14	-7365.35	4059.5	-11157.8	-9523.33	-401.705	-1903.81	-4208.41	-6710.96	-1495.2	2154.49	1809.28	-4901.29	3143.89	-6836.04
1906.96	-3387.26	-4537.84	-8605.6	-3425.04	-436.676	918.023	-1615.44	-6380.99	1339.86	5971.14	-1786.49	-77.002	-5625.76	-11540.9
-1373.11	-5918.09	-3076.64	-5630.77	-1144.04	2683.47	-5112.87	-957.977	-6672.14	-2631.16	1775.11	-6033.47	-5784.43	-3175.16	-1570.71
345.029	-1144.22	-6106.45	-11296.7	-10054.8	-6851.88	-6679.18	-4095.32	-3856.1	-4016.85	1912.76	5197.61	5434.55	-3356.62	-11902.1
-198.6	-8441.14	-881.922	-9083.41	-9797.93	1348.73	-4239.67	3694.11	-5243.93	749.229	6758.97	-3917.22	2362.04	-6928.8	-7876.82
608.869	-5397.47	-1219.08	-9871.34	-5531.86	-475.615	-2346.7	2828.92	-3769.48	-4723.15	9553.44	-3797.3	6192.2	1803.14	-4369.3
-2352.7	-9604.29	2117.25	-10226.7	-1608.46	4673.66	1998.82	-585.943	-2091.52	-2102.23	4864.22	-6038.41	-4495.52	-1400.79	-2822.58
-4239.81	-4356.98	905.223	-7327.04	-10289.5	-4626.62	-7337.39	-3091.63	-3857.32	-5647.61	3455.88	-6688.85	-3373.08	-1926.85	2140.95
7001.35	-1582.34	-2673.64	-15427.6	-9600.03	-2963.02	-3592.49	-1164.28	-3679.21	-5988.09	7816.15	-421.615	644.1	-4868.64	-1305.74
-3190.98	1511.14	2696.86	-10041.5	-7470.18	-282.727	2521.75	-1600.45	-3687.81	402.805	8623.62	-1650.97	-41.5664	4327.27	-1533.67
983.9	-7034.82	-2417.31	-6556.39	-7485.16	98.8594	769.043	1986.15	-5927.59	705.433	-2837.13	-6096.19	-2862.36	1498.15	-760.486
1884.21	-6758.78	-6279.08	-20114.4	-7523.22	1787.67	-2850.66	-1472.32	-5676.58	-842.456	2508.28	-5749.03	2670.22	641.035	-5655.97

-752.811	-6759.33	-2243.41	-10581.3	-450.664	1886.82	-9630.22	614.475	-4532.11	3346.84	4722.23	1470.58	-781.738	1318.18	-2796.79
-5627.59	-4360.44	-4552.02	-12364.4	-6048.78	-1418.67	-6233.32	-3490.89	-5069.94	-9007.21	-1692.01	2420.32	1665.6	8604.07	-11060.4
-3443.49	-4346.77	-1010.68	-7795.03	-5611.26	3755.96	-6196.36	-2411.79	-2918.66	-802.463	5267.42	-2330.04	579.045	3503.27	-6707.13
-333.672	-2699.47	784.016	-15374.2	-11692.7	-3802.46	-3130.83	-2957.21	-2613.43	-629.269	-5497.74	-5144.02	4405.5	-3032.68	1030.12
-1367.15	-5721.57	-3433.7	-10946.3	-8628.22	-5105.66	-7639.25	-1599.17	-4812.67	-2554.05	3623.72	-4152.3	3656.1	-6423.94	-1508.61
85.5918	-2601.51	582.971	-8905.23	-14504.6	-3730.3	-12468.4	-627.332	-17098.4	-2794.45	5161.85	-79.3262	5708.14	-5277.77	-3817.83
-2723.11	1530.7	188.75	-3444.06	-549.373	-202.41	-3255.96	1382.14	-7872.95	-6889.36	4440.77	1833.38	959.682	-3283.51	-8981.29
48.8828	-64.7578	8559.01	-7991.51	-6444.59	-4168.18	1288.97	-3937.14	-10552.8	-3483.02	-5371.82	-3810.15	1364.54	614.254	-6649.98
4161.27	-2370.73	-712.551	-1661.18	-7274.13	-3647.23	-9904.88	1400.73	-5077.07	-4864.25	3317.85	-2096.11	886.48	-7212.82	-3487.38
-3563.83	-6022.12	2805.9	-9599.4	-7953.77	-4323.1	1927.65	-672.125	-5506.12	3948.37	3821.25	-2893.66	561.863	-6037.89	-3226.92
-2291.02	1240.96	-5310.1	-5099.22	-6460	2956.45	7824.84	-2763.21	-5632.98	58.3369	5900.49	3129.31	168.266	7620.51	-6067.45
-1893.73	-8465.53	-2336.44	-8356.64	-7030.42	-1899.43	3147.49	3412.12	-975.643	1604.16	5742.11	3079.71	-2853.55	2096.7	-6451.11
-715.508	-2765.86	2553.28	-7631.32	-7033.8	-919.74	-2923.51	-801.699	-3546.15	4449.56	-2037.89	-427.297	3086.55	2465.29	-4831.58
-2384.92	-287.947	3970.84	-7768.59	-11890.6	-1688.1	-2265.55	3645.14	-6640.11	7376.87	-1157.37	1147.5	-3321.39	-6048.16	-4507.78
4232.94	-7644.15	-1837.51	-13638.6	-3595.08	-3460.84	-4772.75	-4442.35	-5126.54	7121.37	1865.98	3734.3	-6254.67	4702.01	-2098.09
-1337.27	-3085.95	1789.08	-8171.8	-7153.4	2090.47	-1678.14	4115.34	-7217.46	2315.55	2927.37	5074.08	-1731.85	-5183.04	-5302.7
-1327.5	-6618.27	2532.77	-6017.49	-12185.2	-3814.41	-5401.42	-242.037	1906.79	1139.89	4326.3	-6684	1025.29	-3767.35	-5194.07
-2817.04	-3419.7	952.83	-6050.56	-4935.95	-2007.32	-6341.43	-3921.88	7565.55	-2448.95	-3929.99	-9296.96	-3082.75	372.113	-1339.37
944.693	-745.533	5463.15	-2008.68	-9911.93	1224.62	-4003.93	-1274.2	-2197.66	-2772.96	-4805.12	3347.19	-612.303	-1822.42	-6952.29
-3582.15	-3606.83	-239.289	-14871.5	-4421.91	720.344	-3577.91	-7455.36	-2922.09	-4091.75	7899.5	542.597	-2715.62	4927.75	644.516
-9933.86	1802.58	4033.47	-10745.4	-2217.77	-6080.39	-3549.28	-1042.15	-5348	-3348.97	-546.973	1311.34	-1556.27	4390.58	-3206
1046.61	-6954.88	7571.71	-10832.3	-3966.05	-6162.31	-5660.84	-8384.91	-6490.2	-7559.06	4477.46	-793.553	-6597.51	-233.763	262.857
-8399.68	-3371.49	-5324.05	-8159.34	-9609.36	-464.355	-2087.9	1169.93	-843.971	35.5156	5645.23	-2864.13	2233.75	-1492.09	-3674.09
2299.6	1704.35	3908.09	-5666.8	-11534.6	-2150.2	-3493.87	-2369.58	2200.78	-2581.45	4201.67	2684.62	-2826.74	-3622.29	-3085.8
1320.79	2161.9	-3299.34	-9085.13	-5844.23	1958.68	120.918	309.771	-3105.12	-6311.32	1176.38	125.569	9463.98	2719.71	-3910.13
-102.078	-4286.45	1308.19	-12550.9	1608.87	279.461	-509.037	-1559.53	-14459.9	2249.48	5755.16	3435.82	1406.78	-1058.81	-2485.8
1501.09	-5806.64	4279.5	-12690.3	-11781.3	-606.795	-1050.31	-6458.63	-5099.66	-1865.56	-6609.58	-2148.66	-2196.95	4685.4	-5550.24
3722.87	-2279.79	-361.051	-8104.91	-4655.57	4248.92	-6512.7	-2326.31	1996.51	5144.63	5778.47	-3593.42	-1779.51	847.922	-2415.7

6807.12	-779.066	1836.49	-6910.28	-3797.42	-2765.58	-375.193	-9025.5	-1346.78	531.833	1109.78	-259.557	5877.69	-1319.72	-1928.71
-649.08	-6811.72	811.912	-13074	-2873.77	-636.803	-4732.61	-2486.77	-8307.05	3756.75	1722.22	-2160.41	7365.07	-4618.13	-2521.03
288.779	-3563.12	-2573.57	-10139.9	-8956.85	1069.84	1863.24	-4491.43	-1148.74	46.4463	2902.56	-99.5498	1780.35	2497.91	-3402.54
509.289	-7749.67	-247.555	-3605.99	-10745.6	3551.53	1252.5	3143.12	-5714.74	-5337.71	6969.84	4096.93	3722.2	3377.5	1302.45
-5917.75	-7703.08	-563.367	-10728.7	-6622.84	-674.326	-6881.8	-2510.97	-5948.14	-1486.82	4019.35	4473.87	-5075.66	-4453.77	-2502.16
-965.861	-4246.18	-2363.15	-5864.43	-6421.17	6759.79	-6568.08	-4611.73	-342.746	-1327.14	-1788.71	-383.739	2650.6	2885.61	-3499.63
238.334	-11344.7	2332.76	-7547.02	-4924.52	-1929.49	-2022.24	-7371.16	-4877.74	-1355.17	4856.92	-3216.68	-1117.94	3010.56	-2123.29
-1534.09	2429.47	-2951.02	-4385.37	-7976.76	3746.03	-8832.94	993.418	-5099.92	-2427.35	4274.96	4210.4	8576.74	-1285.7	-10183.9
337.477	-2541.49	-1720.79	-11842.8	-11152.5	-13416	-12849.9	-6258.76	-5400.14	1387.94	-1853.92	-2509.08	3725.12	-2365.48	-4653.2
-2337.16	-8368.94	-410.17	-10163.1	-557.422	-7037.03	-5776.9	-5883.91	-6641.5	-2739.91	-806.213	-5368.87	1549.45	-2057.96	-6797.92
1992.68	-270.178	2349.41	-5964.58	-9669.59	-938.85	-2280.34	-8530.81	-6968	4355.99	-227.27	-140.944	2917.49	-2004.95	-5674.65
-1268.69	-3062.68	4799.46	-11824.7	-8092.74	-5831.09	541.25	-42.9883	-3539.42	-4144.44	-1751.36	-157.132	2279.07	2311.49	-9703.36
1251.98	-537.166	-1828.57	-3441.91	-14299.1	2868.9	-1289.65	-4210.87	-11439.2	9608.01	-8819.79	-2671.72	-1789.36	5658.81	-9398.59
-1650.28	-941.811	-995.301	-13946.1	-5497.69	5716.17	-5795.17	-7395.62	-2423.7	-2338.61	-4904.94	-1669.9	-3394.07	2699.11	-3558.43
-2917.59	-3301.72	5509.33	-10760.7	-9756.22	226.129	593.596	-1459.46	-3145.89	-6680.02	2884.06	-2072.71	-3259.52	796.865	-1666.63
5285.3	-1972.49	-3608.92	-13402.2	-1673.12	-5320.4	-3104.05	-4686.01	-8428.33	3119.85	-3614.11	-71.1426	-2095.35	1063.67	-4639.66
1022.64	-761.205	1154.55	-14980.8	-1029.79	-2084.58	-5037.09	-977.729	-5233.28	572.011	-5569.03	829.649	785.174	-3299.67	-2857.7
-244.986	-4667.89	3485.06	-15348.3	-12676.2	4707.72	-8922.43	-1684.27	2719.81	1695.48	-2574.29	-2617	5948.93	-5489.46	-342.924
-4307.02	-8210.08	-4570.34	-5012.42	-9964.72	-2560.71	-5055.23	-520.135	-5271.04	2492.03	-1485.36	-3608.25	-5397.27	1818.03	-4574.61
565.072	-10915.2	-4372.56	-19028.4	-5692.93	649.123	-5039.53	-3216.59	-614.203	-962.584	5727.97	105.346	-3287.54	-5103.14	-2483.77
1537.46	-8310.59	757.389	-14958.1	-4332	-75.1406	-2240.72	-7180.6	-7157.99	565.77	7274.97	-4423.82	-1591.74	-6319.04	-6334.07
-4656.67	-1188.81	-5260.23	-16786	-6750.13	783.133	-3929.26	-5722.54	-3889.81	1579.19	8514.35	-3842.16	-963.652	-4800.11	-2413.48
798.32	-12362.8	-1846.97	-13214.3	-4917.18	-966.195	4295.35	-4922.3	-7466.89	4348.79	3291.22	-3786.88	3279.87	-262.16	-992.341
-9755.71	-9003.15	-579.979	-9399.82	-9671.48	-2311.66	-94.9141	-1623.77	-9368.64	3360.95	-5168.03	-1428.96	-6767.42	-4460.47	-5181.26
38.2695	-1239.96	3172.27	-8746.99	-4212.87	7101.88	-8727.31	3291.72	-7942.18	-1195.38	2535.5	-5043.35	-8607.3	-8006.42	-4403.42
-7495.19	-5307.54	-975.986	-11753.1	-4500.66	1130.44	2328.94	-4294.17	-11173.1	-4300.24	1669.41	-5761.84	-789.92	-3113.91	-2136.88
-396.666	-10252	1029.33	-14925.2	-5210.18	-8006.41	-6289.23	5366.2	-6597.53	4768.14	3356.8	-1648.58	4538.24	-1426.01	-6900.27
-65.9629	420.65	-635.496	-1086.55	-6992.9	-1330	-3066.67	-5302.73	-5213.82	-6494.53	3104.86	-1000.07	273.682	-621.2	-9574.23

-1315.79	-5275.07	-2327.26	-12477.1	-5390.48	-6858.59	-8180.04	-3514.69	-7120.59	3050.42	-442.797	1798.62	1680.38	-5343.59	-3105.53
-8224.96	-5921.46	-1460.42	-10356.5	-9009.91	-7159.67	-5926.26	1306.35	-4527.16	-2333.07	-3905.43	711.329	-4737.75	1130.35	-7581.89
4079.48	-1164.94	5886.38	-9787.54	-9662.33	-1368.17	-6555.59	1550.76	-2275.38	4147.12	-7017.7	-7763.99	-1988.01	-2830.93	-11530.1
482.662	-1527.48	2610.54	-8524.54	-8225.75	1979.78	-1936.76	1623.23	-11264.1	-5139.25	-666.762	-4745.37	314.264	-3629.87	-9789.19
-302.736	-4576.68	-9354.75	-7786.69	-4479.72	361.525	-7024.78	1691.95	-2580.48	-4671.3	-2933.07	-8073.29	-253.287	-5508.91	-872.33
3675.33	-7517.65	262.35	-5140.39	-9306.32	-6533.36	-1648.32	585.967	-9512.45	-188.049	3873.76	-3830.51	-3607.79	515.609	-2555.82
-6099.14	-7170.99	1064.98	-8809.99	-6557.39	7710.41	-3682.46	-3082.09	-2696.65	-3438.04	2139.26	1802.77	342.293	-8498.71	-7065.68
-471.771	1638.1	-803.754	-8536.4	-13194.1	4775.97	-3990.57	3442.15	-6276.49	-2422.95	1777.79	-5100.35	-36.3457	-6883.04	102.889
-2264.46	-2222.29	-4930.35	-14579.5	-9807.1	-4173.26	749.025	-5952.22	-5332.39	-6087.72	1874.02	-9399.21	3986.39	2369.33	-3692.42
-1941.93	-3310.11	1046.22	-18184.3	-15202.7	-274.186	-774.146	-4077.19	-5956.68	-3476.74	-3002.93	-3678.41	-2093.31	5221.83	421.285
-1156.65	-3368.67	5364.08	-8674.37	-10157.3	1692.32	-3425.79	-6693.52	-8227.4	3497.7	2601.96	2064.93	-2625.46	-2623.21	-6801.3
34.1797	300.582	-855.693	-10939.6	-9466.17	-3449.38	-2050.31	-8006.12	-3780.7	9614.21	2902.33	-2650.6	-4017.05	-2141.85	-7158.52
-9689.57	-2521.9	-7242.34	-10906.6	-5147.82	1658.9	-2995.59	1797.54	-3288.87	-3231.39	6379.9	-1810.57	2155.88	1860.77	-3871.72
-5403.9	-5217.02	3166.07	-6073.8	-3497.98	-2944.66	-1444.84	-5432.88	-9329.82	-3245.9	3155.9	384.597	1523.61	279.23	-5989.88
-2184.2	-1142.01	1672.84	-9771.05	-9365.74	-6261.66	-3604.38	6186.47	-2444.58	5076.01	1710.82	1438.92	-3445.21	1793.49	-4023.68
2050.81	-10535	-4914.61	-12318.3	-10488.3	-7274.77	-3046.82	-1857.84	-2295.86	-835.772	-1242.11	-2402.23	-4501.33	-272.355	-8496.39
-1063.22	-1513.66	-3588.77	-10594.5	-10224.3	-5544.16	-3292.82	-1781.11	-7447.89	-2700.69	5600.68	-2952.15	-1859.89	-2912.52	-9283.66
-2194.16	-7091.04	-279.863	-6598.65	-6426.22	488.047	-3308.83	-1081.8	-16333.9	1706.3	9842.13	5179.55	-2363.13	8664.7	2046.76
-1974.71	-3527.98	1472.27	-8769.8	-5518.36	1252.22	-8579.48	-3021.69	-3823.38	664.664	5486.65	-4810.21	2733.69	-1344.68	-779.869
2345.64	-7380.66	-5376.27	-7032.98	-11796.1	-2460.01	-5113.12	-9096.4	-5663.39	-5130.8	-6867.78	2402.51	-1266.79	-6287.44	-9708.44
-362.172	-2254.15	3229.93	-10661.7	-10491.6	-2883.31	3172.6	-5246.32	-13300.9	-3120.12	-896.984	3598.96	973.012	-1822.68	-10174.2
1343.2	-301.707	-3484.65	-10622.3	-8363.69	-3168.05	1679.91	-3825.38	-7539.15	-1763.97	2053.63	-3748.6	3165.24	-1225.72	182.859
4960.31	-1070.26	-2356.19	-12801.5	-2544.41	-4002.81	-5270.47	-2038.29	-7663.77	-7126.55	2499.08	-5527.36	-6099.49	-8375.37	-3759.17
-1072	-1627.9	684.029	-9797.03	-10081	-3790.7	-5665.23	-9077.43	-4862.97	-5602.48	6804.46	-5214.2	-2740.1	-4671.01	-6141.74
-3086.59	-4567.81	-2418.55	-13342.2	-3843.6	2820.89	-12687.7	-3727.68	-3172.41	-2497.4	-1068.82	282.014	-3942.26	-3893.87	-6036.17
294.896	-4058.28	-1603.48	-16414.8	-4033.58	1908.26	-776.762	-4633.89	-6568.27	7289.43	-4233.22	1713.8	-414.836	-639.453	-6814.33
1618.74	-4085.92	-7334.34	-9531.19	-6646.42	-1433.42	-6691.74	4256.15	-4350.82	-3710.42	2220.78	-2230.75	-5449.89	-5035.66	-6899.33
5854.25	-7087.25	-669.949	-10793.7	-7514.78	-233.527	-216.414	3725.22	-1590.04	-4921.71	-4420.49	-4195.61	-2239.01	-8088.27	-3810.75

-1143.92	-2196.2	1438.05	-10531.7	-10041.6	-1166.87	-1153.68	3544.04	-395.059	-8347.74	1325.89	2468.63	-4858.38	-1425.15	-13071.1
-3366.73	-1140.37	1665.61	-5822.7	-8024.83	-1821.92	-9623.73	-6170.16	-8779.94	-1465.4	727.813	-5740.92	-6649.68	2425.68	-6054.5
-3742.23	-1136.27	-4857.7	-9133.39	-4662.34	6435.91	-1558.14	-422.715	-11305.8	-2107.95	2343.08	2743.96	-1286.45	-607.797	-3629.67
41.5156	-4274.88	-3087.85	-8668.1	-9137.46	-1855.95	-10377.6	-5404.82	-7954.06	4667.37	3113.86	2868.3	2533.85	-4675.31	-5579.68
-782.623	-1109.29	-6067.67	-11027.1	-11730.9	4597.17	-8845.91	-1910	-3140.88	-4101.46	8178.96	-3850.32	2347.86	-1342.18	725.92
-4439.32	-5356.56	57.5684	-11543.5	-10115.2	-6297.93	-1709.84	-2244.17	-5520.63	2957.14	-401.77	-88.2998	397.057	-3708.16	-7037.07
2481.93	-2399.71	1002.46	-15841.1	-6794.87	-6768.32	-7996.5	-3475.92	-7285.57	-8813.85	-406.381	-472.704	3842.61	2277.3	-2905.2
-7436.44	-7009.73	-2948.72	-7819.23	-10345.4	-2120.88	380.865	35.7812	-6402.63	-3751.86	5157.85	-1126.94	441.674	-5989.1	-5012.15
-2212.36	-3723.62	1356.47	-12031.5	-9896.66	-5126.6	-2990.96	144.459	-7039.94	-1107.93	1307.79	-4345.67	103.912	-6345.38	-3654.84
-3787.93	3269.68	-3719.79	-10604.8	-2674.1	-3712.38	-7049.02	-5221.56	-7726.77	-1313.14	548.953	-7657.3	632.252	2353.34	-1957.18
169.439	977.74	1580.72	-13976.1	-6620.36	-3853.71	-5063.39	1071.65	-1202.47	-77.3008	2520.57	6434.13	692.754	-3168.9	-3686.04
-4313.42	-2040.08	-3961.29	-14474.5	-3688.79	-4485.01	-3264.26	-1061.63	13.1191	-1683.5	6001.35	-2713.66	1478.99	570.551	-489.482
-3792.14	-1334.09	-2602.42	-14225	-3596.63	-8285.19	-7481.42	-4596.69	-7294.45	-3025.66	-2832.87	-4214.28	-4098.7	-2343.44	-7407.35
-1381.19	-6130.13	-8720.15	-9713.36	-11501	551.168	-4878.94	-2118.09	-114.199	-1909.24	1851.77	-5725.63	4758.36	-348.367	-8528.13
-1847.94	-2373.92	-3847.2	-10345.4	-6127.35	-4186.21	-2840.93	933.139	-6271.55	743.506	2071.85	1427.72	-3282.8	2915.1	-9534.49
-1013.34	-4135.19	-698.404	-12347.5	-14457.1	-3624.52	-3920.44	-3961.56	-3831.49	4913.68	5829.06	-2289.17	-2038.55	899.004	-6880.62
-6502.52	-2511.66	4206.42	-14251.2	-2204.82	-4642.21	-6494.11	-10141.3	-2032.65	-116.187	3910.3	585.614	2061.5	-310.062	-6979.19
115.811	-5410.44	454.135	-9418.74	-1669.34	-3092.45	102.121	-3002.02	5689.43	983.134	7097	3229.44	-1221.36	1223.96	-5882.55
-6059.37	-4682.89	-2203.95	-12701.1	-12632.5	3694.94	-2998.45	-3816.64	-5965.88	-4757.45	6628.7	-6196.34	-1910.03	-4185.19	-2159.4
-1984.41	186.982	-3463.63	-10750.4	-8171.61	-7448.02	-6284.38	-3909.4	-3556.87	-8227.46	1914.31	-5770.56	-7625.04	5070.67	-10863.1
-2161.32	-9173.61	-2651.1	-8002.35	-9057.07	-7656.27	-1072.49	-4107.54	-10506.1	-1060.55	-7116.51	-7509.36	3190.19	-1237.4	-383.693
-2788.61	-4247.15	-1586.83	-15616.4	-12117.3	1769.29	-2286.09	-1347.8	-5285.95	-5293.84	-2098.43	-6628.34	4288.49	-7111.28	-3885.96
-165.635	459.195	2137.79	-8436.09	-14592.7	773.064	-1348.17	-6182.08	-3678.31	-4540.02	284.189	-1819.18	784.729	-1228.77	-6288.73
-2217.6	-8328.54	2687.52	-10208.5	-3537.98	-5187.75	-4809.89	2164.35	-5112.39	-1041.06	3412.78	3182.36	-2898.43	665.859	-6236.8
-5059.93	-8649.6	-3659.47	-10224.5	-9071.62	-6167.64	-5607.03	317.846	-7130.22	3370.18	5852.47	842.297	-2904.98	-1933.76	-6010.65
-30.5	-6067.8	6959.12	-17031.8	-570.777	2120.17	-2615.37	-5110.33	-10846.1	3205.74	10268.5	484.79	1532.45	169.998	-5346.23
-1278.12	-8250.73	6316.96	-9517.94	-9228.87	-4106.36	-9341.36	-8025.25	-7749.36	-1106.35	4289.23	1462.21	-5817.9	4067.24	-6157.86
-2328.73	-1808.47	-2468.74	-9770.26	-5280.51	-2890.71	-9516.22	-1339.86	-6134.4	-4972.38	-2456.83	-3860.42	2407.62	-6126.65	-5502.46

-5203.08	2989.54	2528.04	-7295.13	-9553.27	-1477.38	-3002.5	-2994.95	-16.5352	-6970.13	3669.13	-7158.3	-3617.75	-4574.82	-5457.04
-8164.16	-1822.35	-5036.73	-14387.7	-6734.95	495.812	1229.37	-1375.52	-2899	2207.68	4268.85	237.464	-1624.58	-2999.15	-14864.1
1264.75	-3251.79	-5524.02	-16232.9	-9403.54	-2484.86	-6025.48	422.979	-4218.2	-9794.48	3271.71	-2822.4	1232.2	-7187.82	-10392.7
-1663.7	-2001.47	1357.42	-10948.8	-637.186	-4444.69	-8005.56	-4285.07	-452.549	2124.48	2852.92	-4842.33	-4515.58	998.537	-7388.99
612.553	3151.1	-4764.04	-2879.15	-7256.4	-2640.17	-4739.52	-2708.5	-2581.89	410.19	1323.81	777.546	3451.09	-3622.37	-2946.51
-4342.27	-7580.52	2349.41	-15431.9	-12135.3	-1553.76	-7704.08	-8274.76	-5643.99	-235.354	2137.87	-7731.16	216.289	-6192.05	-2002.63
-1269.25	-4327.88	938.047	-10206.9	-7263.35	-6819.27	-4341.33	-8319.6	-6995.18	-2955.79	-2059.81	-4952.9	-4815.02	-4919.78	-8420.42
-1973.52	1231.67	5725.12	-10805.7	-5436.24	989.553	415.32	-6434.43	-9145.01	-6328.64	5484.06	1120.15	-4866.82	-3893.45	-3798.37
371.283	-3742.18	5571.76	-10278.7	-11771.2	93.0078	-1398.24	-3378.72	-4270.18	3454.79	521.965	1597.8	720.857	-3708.39	-7434.2
-2882.85	-302.436	4101.62	-12029.9	-4031.87	-429.521	1419.64	-3238.53	-4722.09	-909.702	3519.16	-6356.14	-710.275	-1980.05	-8639.08
-7129.06	-5703.77	-4071.89	-11742.3	-9223.55	-2226.52	-1898.09	1628.72	-5915.35	375.958	-2243.66	-1200.89	-3150.54	-1959.34	-4317.26
-3487.91	-7960.04	-1608.77	-12503.4	-9663.31	-3879.61	-1781.37	-2136.68	4387.58	-598.175	7214.33	-1885.42	3845.35	2391.31	-8645.76
-3194.63	-3694.92	1016.61	-8320.71	-7176.29	7350.85	4674.92	-127.588	-2349.75	-495.588	5283.99	-6266.77	4218.03	-4429.83	-6907.25
2876.1	-3328.12	-7707.14	-2063.43	-9234.24	-1650.47	12.8105	-2488.86	-4409.83	1332.88	1877.01	-3653.91	1219.39	-5200.2	-6226.66
-697.027	2118.77	513.387	-6729.83	-2605.13	-4431.26	787.666	-1892.27	-2820.73	-3676.74	5997.52	-153.885	-674.656	-1247.47	-6527.62
-3627.26	1576.9	-236.268	-9950.65	-8282.29	1436.9	-5182.3	-5926.44	-1217.88	-3330.32	995.693	1081.48	1748.21	-2096.08	-6668.82
311.816	-6548.2	717.053	-7880.11	-6820.85	-3143.26	499.576	-4022.48	-9516.61	3018.66	2401.09	-10946	92.2871	-7297.44	-6217.6
-4115.64	-2990.09	-4757.68	-9298.55	-5731.03	-2410.51	-2699.62	-4198.33	-4622.05	-2656.49	-4388.31	-14632.4	-6761.39	-3111.9	-5696.82
-116.064	-8224.45	4323.65	-12806.1	-10642.7	426.062	-11592.9	-5487.66	-2107.98	-393.223	9314.32	4489.54	2869.54	-2362.61	-7258.14
-1456.58	-7127.76	-3785.76	-11743.8	-11789.6	-3845.36	-5812.4	-2102.2	-2611.25	2169.16	2329.27	425.021	-2423.59	977.963	-8316.74
-2723.11	-7377.99	1375.82	-9655.7	-3571.01	-8261.7	-4074.86	-2192.33	-3492.58	349.037	2663.49	-2769.39	-774.146	-2594.61	-8808.81
942.258	3635.47	1363.26	-8160.99	-15708.2	824.512	-8234.58	169.02	-6962.13	-3361.86	1236.81	-4348.83	-2062.65	-813.172	-5664.11
-4659.95	-816.365	3332.38	-7112.5	-10205.8	-4004.97	-2546.88	-2089.4	-7002.8	-3821.06	-699.172	-2317.6	-1613.48	-5099.54	556.623
1945.79	-185.283	-171.055	-13767.1	-10115.6	3449.25	-4655.37	-4623.75	-9699.14	2057.45	5263.52	-973.873	1066.69	2844.12	-9247.76
-1560.81	-2653.75	6605.12	-16286.5	-14897.6	964.537	-3026.14	287.215	1883.8	-9329.22	5389.36	-8509.37	-666.324	-1223.58	-9698.46
-4152.19	-3126.98	4962.03	-14142.2	-11286.9	3457.35	-5000.4	-3920.75	-7447.49	2264.11	237.588	5421.02	341.701	3781.47	-6638.33
183.299	145.826	-931.518	-6661.52	-8214.52	3820.35	-2194.41	-4765.43	-5221.24	-2516.2	-1945.02	-5080.78	-4789.1	-5910.95	-4991.98
-281.01	-8313.33	-1661.78	-4569.98	-3742.05	88.1543	-1487.7	-606.496	-5272.43	-5164.26	1424.54	-905.063	1329.32	-8502.67	-7666.54

-994.424	-2632.52	-485.158	-9532.48	-5544.95	2448.43	-5640.79	-1182.06	-7994.63	-5716.41	4509.59	919.9	3718.2	-4808.67	-3038.54
1478.93	-5326.4	5210.13	-7647.04	-9623.87	1619.38	-6962.32	-2752.55	-7846.79	-9334.13	-28.9512	-4328.97	-2360.28	-7811.37	-6173.72
-3351.96	335.754	-917.809	-5066.95	-7688.25	-710.656	-1355.8	-324.293	-4096.88	-1119.97	-1823.13	-10123.8	7172.53	-4385.43	-371.799
386.391	-3202.43	-6184.94	-3784.76	-6483.4	-2156.69	-2262.27	-2536.84	1113.99	-7194.47	1619.89	-6292.5	-661.178	-261.221	-5369.94
945.037	-3109.52	3463.52	-12285.1	-2810.49	-2092.24	-6140.87	-5135.29	-5116.51	-2641.01	3835.72	-4943.59	4730.11	424.682	-4159.45
3428.22	-9713.45	-1758.27	-12674.4	-10055.2	-6151.86	-7655.57	-6079.25	-10120.4	-3798.9	2697.17	-1116.59	2416.36	-3286.32	-9200.79
-3366.17	-9370.68	2753.09	-8513.99	-5614.33	-2782.08	1491.48	-6071.62	-3612.61	-5608.91	151.021	-6077.91	-5701.01	-3808.65	-1092.16
-5818.26	-5911.56	1486.38	-13742.9	-12623.2	-2903.14	1057.83	-4102.21	-4308.11	-3697.9	4801.62	-2755.93	-2677.41	-4234.97	-6545.43
-69.0215	-6683.73	-2583.31	-6299.32	-9590.35	-540.096	-1918.63	-3727.24	-3616.93	1088.86	-1685.35	6394.79	-285.502	-3624.76	-7321.75
-886.729	-2074.1	-382.113	-6951.52	-8416.33	-10681.2	-3630.26	-2439.24	-5128.09	-4122.17	-5069.81	535.698	-3542.07	965.523	-47.9141
1612.19	-1995.73	-4693.62	-12225.3	-11029.5	-9924.94	-6615.16	-6034.17	-2018.83	-610.952	4346.6	-1387.23	3210.96	-5969.36	-6808.6
5542.24	1439.34	-4417.46	-3471.78	-6225.79	-4900.88	-5202.46	785.355	-5691.46	-2780.46	-195.619	-1168.04	-176.715	2081.66	-1206.38
-2542.34	-7410.21	-352.586	-11634.4	-5193.78	-3339.61	-1311.79	-333.527	-893.941	-2196.66	272.279	-3122.05	-1240.58	-2246.2	-4487.29
-6034.34	-2376.26	48.8652	-13105.2	-10811.4	3372.12	-3104.99	2129.93	3297.69	-7026.74	-1317.29	-1741.28	-6443.84	-3693.19	-8306.76
-1695.32	-3292.95	-677.779	-6192.18	-6568.41	-526.229	611.834	-6112.48	-4181.19	-8030.19	-1143.86	1680.5	-3796.56	-138.586	-7344.28
-3996.42	-8598.69	-567.539	-18516.9	-3461.37	-1878.02	-283.65	-5161.8	-6175.96	-4143.56	2878.11	3251.13	6795.74	1766.21	-5374.05
-814.807	-2476.79	5370.52	-11157.8	-3870.92	-6187.63	864.039	-8282.36	-8046.41	-1550.82	2822.69	5052.19	-476.746	2070.39	-3178.85
-1723.38	35.3242	-5687.43	-14835.6	-5368.39	853.658	-4710.19	-10412.1	-7876.74	2175.4	-3381.05	-1768.83	-3868.28	-5836.35	-2577.72
4674.71	-9598.58	-3957.4	-10224.4	-1954.09	-6390.28	1583.41	-13292.5	-11204.8	-2224.43	-2972.93	-3166.62	377.283	-2541.41	-2652.04
-3207.75	-8302.94	-583.236	-9172.68	-10453.1	-2780.97	-8589.07	-3882.45	-11471.1	-2380.74	4003.13	212.46	564.9	-1136.23	-5006.96
-4417.93	-8303.36	-1979.1	-10153.3	-10685.9	-3029.69	-3988.83	-507.934	-5177.54	-2404.75	1371.53	897.174	-339.338	4706.67	-6641.82
-2512.03	-8010.28	-320.318	-4874.75	-13180.3	-1388.52	1162.21	-2856.36	-3020.88	-5972.95	1796.22	-3143.88	-3192.31	-4419.67	-2243.84
-3042.64	354.678	-1160.66	-10786.8	-3921.82	368.82	-7229.42	-2816.48	-1770.75	-5183.72	-343.586	1118.82	-2319.69	-1747.37	-4935.83
-2909.32	-5387.69	-5009.43	-17629.8	-3824.96	500.832	-1142.13	200.832	-6645.75	185.112	1546.51	-4548.16	-2802.86	1719.28	-1245.01
2003.02	-2341.57	-1967.54	-8671.93	-7271.24	-4270.8	-2055.61	-3418.43	1728.65	2470.68	2852.41	-3256.56	-904.297	-6244.49	-2287.98
-257.473	-1691.87	4631.67	-8729.16	-12240.4	-3793.94	-7105.69	-6186.1	-5853.54	919.654	-1736.23	-1186.46	2357.6	457.773	-2882.9
-3206.68	-4764.75	-6930.82	-6713.01	-5944.66	-1604.18	-8337.28	-7655.6	940.396	-3097.97	-7203.38	-4469.63	-2906.72	-2613.8	-8232.23
2306	-6189.3	-4235.38	-10986	-10076.3	1960.04	-2239.67	-201.551	-8384.66	388.588	1798.96	1252.63	-3242.45	-6677.53	-2332.97

-1727.04	-1745.72	-800.02	-14313.4	-8995.01	2288.21	-2875.17	-2611.08	-727.701	-1092.57	1612.09	-3733.33	165.34	-884.933	-12979.7
-1847.78	-5805.73	-2266.98	-9890.03	-4226.14	-2309.92	-3324.14	-1920.8	-11572.3	-2147.36	1409.39	-3596.84	-5761.51	-1450.6	-6203.57
-4330.56	-7168.47	5535.96	-1823.41	-6155.52	-7428.94	-6428.34	646.947	-1106.19	9.40723	3299	-2483.13	1507.46	-1841.22	-9458.79
-2196.65	-4093.88	2669.52	-8528.26	-4403.07	-3295.6	3578.7	-2634.98	-6904.54	6194.83	-374.172	2536.61	5197.32	-1179.7	-9716.68
1111.2	-917.723	1540.43	-4830.25	-4774.87	-1030.09	-999.5	-9242.36	-8293.04	-8718.98	-4376.04	-8485.61	-1515.17	-580.835	-4192.36
2638.39	-3737.71	-4606.15	-8586.88	-10928.7	-5021.21	-9093.31	-4146.65	-6334.91	2854.29	7346.74	-3403.69	5346.35	-204.662	-2086.52
-3607.06	-7320.03	1589.38	-18717.9	-4142.68	-2107.63	-1388.9	-7202.38	-9123.37	-2387.35	5677.97	-4923.45	2898.86	-1296.49	-6749.36
1144.98	-7261.59	8916.47	-10496.9	-1275.6	-5865.87	-3300.77	-1559.47	-7377.5	-4077.79	9162.28	-6540.93	1774.8	-2680.92	-8887.1
-9560.14	-703.707	97.0703	-13083.9	-8361.92	3939.86	1024.77	-334.027	-3773.14	951.745	-474.902	-1131.78	3470.53	-3116.36	-7182.39
-5160.69	-5225.04	-5512.34	-6896.77	-9908.11	1511.83	-4653.62	-1166.08	-5851.13	-3979.63	-1816.54	-810.371	-6271.22	-3190.31	-9931.8
-2907.54	-3412.74	-3762.83	-8142.23	-6337.12	809.756	-3289.47	-5419.22	-13925.6	1979.02	-713.338	-7865.26	1968.15	-386.334	-8635.03
-365.48	-7380.33	3658.8	-13271.2	-17343	-1379.7	-1417.7	-8755.26	-3891.92	-154.85	6752.33	368.859	1721.29	-5461.09	-2568.83
-803.102	-1566.27	-7984.6	-7245.65	-7222.54	-5939.81	-5634.27	-6579.41	-10330.5	-256.735	174.387	-903.937	-6220.42	-4941.32	-7445.55
-2272.98	-2230.31	-693.078	-10530.5	-6264.7	-2078.79	-8184.64	-9663.03	-3284.78	1007.91	-2332.46	-1591.2	-595.418	-8920.12	-3055.25
343.187	-368.803	-43.9258	-12531.7	-8625.56	-4087.59	-8713.53	-5151.68	-5656.07	-4216.01	9328.84	-7736.98	-2046.88	4155.27	-7398.05
-4231.7	339.611	-2612.52	-13302.1	-13431.9	-3129.86	-2046.33	-5832.54	425.252	-4716.79	-1317.57	-9583.36	2323.24	-2619.09	-3683.36
-4609.79	-2765.68	-1216.53	-18001.9	-7835.83	1151.68	-4027.61	-4576.47	-5588.94	-163.417	-1020.55	-7981.92	-4005.09	1220.34	-3853.82
-6293.62	-9215.8	-4473.5	-16903.1	-4620.69	1342.6	-3102.69	571.283	-5305.68	-4967.69	5729.21	-4041.22	-6597.17	-3211.49	-5476.02
-827.387	-1495.32	-2493.18	-12895.4	-7858.63	-5517.67	-2446.82	1086.85	-4923.26	1931.9	-2855.12	6244.12	2570.58	-2951.69	-6316.17
-3329.96	-4260.97	-1829.68	-10405.1	-8526.36	-2329.66	3518.37	-588.207	-3943.37	-3269.39	6113.43	-136.255	-1434.3	378.918	-3058.65
-2687.31	-5144.4	1543.53	-7350.05	-9104.73	-874.451	-8489.02	-10173.5	-6845.37	-3394.61	-2949.56	669.191	877.26	4003.3	-5826.81
5568.46	-10674.9	251.063	-13069.1	-12463.9	-2717.32	-5768.46	-6439.44	-3005.64	4599.49	-4421.4	-7016.37	-9541.19	-2842.82	-8998.62
-3468.65	-7552.24	1607.35	-11629	-8305	953.723	-3086.44	990.012	-1762.8	-2450.09	5049.99	-980.321	2735.24	-3893.76	-6431.37
3464.03	-9539.38	3352.61	-13575.1	-12818.2	-601.969	-3562.67	609.625	-3762.42	-1803.85	3125.56	-752.654	-215.557	-2695.52	-6337.1
-2776.46	-139.631	1652.93	-9189.04	-10282.1	557.242	-669.246	-6.16797	-4072.41	408.972	-1915.77	-3219.43	725.967	-2887.93	-6372.14
-3869.25	-1544.46	496.219	-9821.19	-10804.4	1196.86	-2299.2	-518.371	-1205.84	1281.44	9663.5	2574.85	-2388.34	-4544.31	-230.564
1013.4	-8309.32	-794.494	-10481.5	-7415.68	1372.44	-2179.69	3735.25	-4636.6	3115.38	2120	-7113.77	-2968.67	6109.98	-4293.68
523.15	-6560.16	-3732.87	-7863.2	-2860.62	4115.91	-2931.74	-5154.33	-10816.5	3357.26	3423.6	-4379.67	-3925	70.3613	-5101.37

686.098	-6449.66	1598.92	-6772.78	-2462.59	-4195.39	-4202.85	-8799.75	-1471.09	-3541.55	-446.75	-4977.34	-1341.14	-21.5039	-5972.53
2157.97	-640.924	4717.39	-10061	-9681.07	518.748	-3864.26	-6285.29	-2507.38	-2985.19	-1730.71	1860.15	-2690.48	-7170.29	-7664.51
-4324.13	-6653.63	2357.64	-7147.18	-8651.6	-5420.22	-2147.68	-1731.75	-7227.62	-4514.87	5818.29	-568.591	-800.473	-2049.38	-3954.07
-494.686	-1745.57	4153.52	-17982.4	-1383.44	1635.44	541.598	4362.85	89.1133	-4880.83	8774.51	486.977	5724.88	3049.41	-8119.79
-7611.72	-11896.8	-3585.31	-8498.2	-11825.1	-3239.44	-1807.14	1001.73	-6905.96	-2782.75	4008.75	2327.23	4289.93	2986	-9173.33
-2470.61	-5700.97	-2659.25	-14290.2	-7610.76	1063.05	-6431.06	-3300.57	307.336	-4667.72	-395.914	-7711.14	4006.46	3258.49	-10835.2
-4413.84	-6683.06	2196.61	-6882.23	-6930.31	-3183.53	-9899.2	-6340.62	-6891.08	456.684	4454.28	653.161	609.443	-3058.71	-6602.05
-4307.3	-6733.57	-389.982	-10659.9	-11801.2	-2600.9	-1427.01	-2944.53	-4459.87	-1476.15	7466.55	-1157.8	-1129.35	-3475.48	-9734.99
-2082.37	-9194.75	1988.61	-10171.8	-9589.88	-2410.51	-4270.12	194.521	-75.6094	-1344.46	-2355.57	-1862.76	-3387.26	-2515.57	-2632.6
-2156.6	-4796.92	-1174.57	-11061.1	-12317.8	-7057.83	521.965	8518.11	-4046.42	978.592	-8461.97	-1435.34	-7121.18	-2069.67	-5820.24
-3286.69	-9665.31	-2240.23	-10564.5	-7231.36	-3304.47	-5742.65	-3810.07	-2170.29	-4827.06	3231.64	-4055.76	-1389.83	-5399.55	-2555.61
-7571.2	-4378.21	-397.016	-8880.11	-11836.1	-5055.41	4548.47	-3614.91	-880.252	-3609.79	-6356.27	-8826.75	694.754	1393.14	-3711.56
-3408.75	123.865	-5981.75	-8009.8	-2887.47	1273.79	-4541.96	-1263.94	-6647.7	-1028.91	4362.87	-2068.6	2602.57	261.211	-819.584
-2544.46	-2471.48	7280.26	-8456.96	-491.299	-6004.76	-1168.92	329.123	-5326.62	-844.782	4344.19	-6704.59	5791.05	-212.354	-2092.55
-4903.03	-4451.5	-2776.72	-13093.2	-1414.91	-1889.06	-2653.41	-5057.77	-869.301	1219.37	2639.62	-5303.58	-5378.24	1908.38	-15983.5
-3311.41	-7027.53	1764.48	-3039.33	-6760.54	-853.873	-6617.6	7247.16	-8343.07	-2179.45	-3726.52	2317.35	2993.65	-5534.86	-2639.47
-5406.36	361.937	1969.06	-15519.1	-3920.99	-5469.22	-7026.91	-4885.76	-8343.73	-3008.05	1600.69	-7709.72	-6798.34	-5635.88	-6578.17
-2645.6	-10174.1	-1045.89	-10651.2	-6893.74	-3617.67	-5302.17	-2540.72	-4707.41	-6533.63	-709.105	-151.315	-2807.97	1571.04	-11628.2
-5012.38	-646.877	2161.4	-8476.67	-1086.44	-2275.89	-7044.9	-1347.78	-5049.06	-4636.66	-1124.78	-597.788	1482.91	-1385.98	-10606.1
-3482.63	-1152.42	3331.9	-17315.6	-5493.99	-1844.44	-6146.63	-4869.78	-2664.12	-4781.2	2986.26	5014.99	469.629	2039.78	-7203.1
-1393.87	-4387.48	-900.322	-9723.22	-8955.41	3701.85	-4057.07	-7173.89	-3527.25	-2180.15	6749.34	-5700.88	-5466.11	-2689.73	-7017.39
-4558.81	-6400.64	76.207	-6216.08	-5218.9	-6661.63	-8667.57	-3894.45	-7456.86	-6679.46	833.18	-5036.68	4228.17	-6093.48	-2192.14
3157.61	-4499.5	542.635	-8696.59	-5989.16	144.121	2774.59	-10.8359	-5877.82	-6199.39	431.092	979.354	-2275.22	-2478.85	-11037.1
-3792.55	-3277.15	-3803.12	-11701.1	-6292.57	-874.729	-7636.15	-9244.54	-1145.58	3085.36	177.453	-2379.53	-2264.23	-4849.89	-3833.6
-623.545	-6811.75	59.3184	-10377.4	-2988.48	-2416.14	1550.25	-5472.71	-2328.05	-3150.4	8458.91	-378.17	2346.23	-3739.84	-3321.38
1381.53	-7097.63	1076.82	-9830.99	-7375.52	-6283.68	881.406	-5339.37	-6705.68	-6322.7	1066.24	7162.76	-5879.7	-1004.31	-759.918
-2330.04	-7959.74	-2146.53	-10152.8	-6954.11	-7523.29	-694.076	-4816.71	-3542.09	-435.948	4352.76	-86.8789	8240.5	4696.61	-7733.83
1740.96	-3931.04	958.99	-9712.04	-7238.96	-1028.71	-4051.88	4566.5	1273.87	-5031.17	4704.9	2852.63	-1401.28	1853.75	-2359.95

-4309.18	226.895	11521.5	-7086.62	-8112.28	-2911.32	-8905.83	-2184.01	4268.51	-1346.01	6969.2	-3881.55	-6183.36	2157.9	-2920.69
-5807.8	-5279.59	-1742.25	-8550.68	-3610.1	-2294.64	-3734.56	1528.3	-2267.76	-2156.96	-1439.56	-7764.51	12629.7	6847.44	-889.472
-6787.33	-5494.58	2644.32	-9117.93	-3181.65	1482.74	-2482.74	-1823.24	-9824.84	-115.743	3857.04	-1447.11	-1115.43	-2288.81	-5221.32
3638.59	-2650.5	-789.527	-7998.92	-7213.02	783.742	-6173.93	-356.305	893.164	-2791.31	-4535.03	1200.37	-2529.97	-8143.58	-6750.96
10021.3	-2589.24	5696.74	-5516.01	-4960.81	-1613.08	-2512	5529.07	-2139.38	1796.48	10769.8	-2383.32	4356.77	-2239.87	-11979.4
2045	-1984.52	6860.41	-9369.43	-13080.7	-792.666	3286.78	-92.5508	-6420.3	-3210.89	4975.79	-10724.9	-5091.1	1322.32	-867.924
4671.25	-5824.87	6420.73	-10381.4	-3749.51	-3254.36	1501.01	-424.057	-6348.6	-1558.53	-5074.06	1280.99	-560.428	-3459.81	3545.98
4997.68	0.880859	-1768.28	-7342.11	-6480.63	407.785	-2822.31	1111.99	4585.16	-3491.7	4212.39	-2675.42	-255.064	-558.982	-3629.24
2401.08	680.621	1202.36	-4343.7	-10987.5	5089.64	-3746.25	-5450.09	-3735.41	5230.78	6310.08	3546.43	1478.02	-1621.58	-3180.5
-740.98	30.5254	-1278.17	-12031.1	-3057.55	4584.75	-1211.43	-2582.61	970.412	5624.77	11852.9	-1319.32	5578.66	-178.69	1863.27
4101.93	1390.98	3513.63	-5367.96	-4865.8	-273.742	-873.92	-2318.04	-1357.04	-529.562	8704.36	8198.72	6361.38	4100.77	-302.316
9621.32	-291.957	7594.6	-6203.69	-5461.26	-3.39453	535.809	1382.92	8445.17	13077.9	4762.85	14153.3	12643.8	11379.1	1131.53
3524.96	-1180.79	4592.77	-9124.36	-4438.09	477.867	5228.22	5960.34	-493.021	13278.9	806.039	-355.579	15717.4	5255.76	-3170.93
1634.95	-1452.15	3893.82	-5618.21	-6098.87	-2880.59	5415.63	-4241.76	451.145	925.009	17933.9	3249.01	6565.8	2017.69	2357.1
2416.04	-2738.16	2574.5	-183.934	-9039.2	-7356.27	1459.44	-476.242	-965.449	850.599	6452.93	5070.86	370.508	3734.3	-9212.29
-1371.14	3574.88	2817.59	-2216.26	-5628.03	-1515.82	-3678.94	-1044.68	-2666.47	1605.34	4853.65	-2002.3	9069.83	-2612.65	-3204.48
7559.06	768.828	-1055.07	-5432.41	-8055.87	-1467.23	4975.39	6140.77	-1836.95	-3179.95	3408.4	-2984.09	-434.795	-1504.13	-5495.3
14336.7	-6921.43	4550.92	1065.85	-7573.5	-3461.28	15482.8	467.371	-2102.64	-6373.51	24358.1	2079.81	-10640.1	-8191.64	-5176.2
-6040.39	6687.28	9162.07	-5487.89	-8745.17	992.66	2030.42	-2239.23	-9202.83	-1119.09	19573.5	4638.43	-1007.27	-3115.63	-4741.9
-610.777	15390.8	17188.6	-7276.17	-3476.21	924.297	6674.25	-209.223	-3292.37	5775.88	5760.3	15444.4	8313.81	5188.06	-1751.28
-3072.11	2932.8	7376.71	-10737.7	-4740.87	-1045.07	1909.48	-106.033	-4864.12	3758.6	2904.54	4066.38	2857.91	2870.87	-6713.57
3280.79	-711.666	4151.97	-2790.3	11731.2	-1445.38	5689.25	-6101.43	-5349.18	-2453.2	4072.48	999.26	-991.236	2538.59	-3183.75
6744.6	-5692.32	5551.34	-5828.1	29823.4	-1085.03	-5051.67	-997	-4859.07	-2456.19	2636.91	-3468.38	3791.44	-4849.95	-5527.28
54696	-5490.51	-1002.53	-931.045	2719.61	-4747.54	2429.27	-2432.24	2265.53	4319.72	2092.41	227.972	828.236	-378.876	-7561.72
23038.5	-1964.08	4389.26	17604.8	-3539.65	1323.05	12883	-1411.98	-3471.08	-5822.65	8925.62	6997.12	-2294.37	-10465.4	-6889.49
975.877	-1495.86	7789.97	4541.47	-5977.33	916.281	12337.3	2772.25	233.352	-7202.89	13438.8	5658.65	-6247.27	-3669.09	-5217.13
-1118.26	2994.97	30668	-8880.11	-14698.6	-1079.29	1822.4	4207.11	-953.807	-6223.8	-62.1719	15448.4	3550.84	159.619	-1527.64
-2902.04	15517	15656.5	-4430.5	-10604.5	3934.48	-6120.44	-5234.88	8012.46	3326.61	-172.254	2754.65	1669.94	-459.269	-2990.66

-4756.66	2451.22	892.982	-11816.5	-5450.33	-5397.31	2433.25	1912.59	2046.51	2942.74	2502.5	5966.67	730.484	2720.91	9932.03
690.75	2672.74	-619.043	-7634.42	-7743.3	706.338	-4345.51	5153.83	588.49	2545.5	-3663.55	-7104.8	2507.01	4662.76	7932.58
-764.486	-2318.27	-8090.67	-3358.38	-7247.54	1420.01	-3659.62	3645.59	-2281.28	13294.5	7883.23	-341.488	14001.7	8970.02	4785.61
8728.54	418.889	-977.258	-5228.28	-4421.79	330.658	-2725.11	13612	-3024.97	15266.7	1745.36	-2909.51	9951.6	1914.82	-3691.88
4890.39	-1319.42	6335.01	-7076.76	-5423.76	8201.88	1076.8	4401.23	-1228.83	4395.94	2520.92	-1670.6	10326.6	7695.66	-2724.25
-2636.33	1940.6	3644.02	-3164.77	-11187.2	-1309.15	-1092.75	4531.46	-3040.34	-968.936	5174.39	2977.94	2687.74	309.766	-1961.42
229.686	2693.03	-2736.51	-10937.7	-4655.33	6177.94	-2094.25	2760.33	1454.52	539.81	2923.09	1862.24	612.998	-2534.94	-1347.64
4537.52	9246.08	5983.43	261.803	5125.2	6151.78	4304.11	-979.752	8287.31	2854.26	16478.8	2347.77	150.975	950.66	-2172.6
-7133.99	-656.719	-813.371	-10027.6	-5968.06	15863.7	920.709	5175.74	7151.84	5456.3	3307.23	11226.3	13535.7	3901.94	1531.38
-1367.49	-1127.94	2970.19	-10330.6	-9938.56	6329.83	-3489.61	1106	-5519.71	4776.6	1320.39	-3455.93	8384.54	6623.13	-2389.85
4921.91	-3007.22	2478.21	-10213.4	2173.6	2971.57	1093.89	-4216.24	-4605.86	-3758.32	3752.73	-2542.08	6787.52	2579.37	-2750.07
26525.1	10510.5	11738.4	14063.3	3997.89	-54.1191	19062.4	-4119.38	3422.28	636.045	28080.6	-4832.71	3516.14	2235.45	-3126.11
9424.32	18735	18679.1	4321.72	-422.592	5313.84	2657.9	-530.371	21871.3	1525.83	14803.4	10809.9	13766	5962.91	-2034.69
4577.63	5815.3	2697.33	4424.97	-3101.25	18201.7	6437.32	2552.62	-1756.04	6021.86	42917	245.67	20963.1	14153.4	16006.3
-1861.14	-451.391	10007.7	-10696.2	-8975.07	11792.4	3457.79	8436.43	-6197.53	20926.8	12545.9	1108.9	16813.3	3184.9	-5750.92
-1267.19	11593.6	10801.6	-6207.74	-7176.25	-4553.18	-1481.84	2093.31	-7238.67	-1000.36	-1531.75	5961.48	-1476.14	-1681.95	-3216.97
240.486	2806.2	-3045.96	-13341.9	-5057.16	-2255.97	-11254.4	-1789.69	-136.135	-3148.19	1040.39	1427.45	2931.89	-2045.52	-683.137
1479.61	4209.36	3057.46	-11391	-6664.31	-3670.03	871.223	-1951.49	-1794.77	-1018.97	1189.6	-2571.06	3851.27	-685.243	-2844.62
-2922.84	-9828.45	2393.08	-13178.6	790.557	-949.693	-3072.31	6881.73	-5774.35	271.192	4217.39	102.076	2997.5	-1604.76	-1308.57
-2780.43	-3878.35	-1630.66	-8631.12	-6576.68	2168.87	-459.865	1231.29	5627.91	-3966.78	-448.6	-7294.81	1233.57	7362.33	-7448.74
-5835.15	2374.01	-320.277	-13100.5	-871.416	-5757.29	-3477.3	-2260.74	-5352.32	-1620.13	2308.36	3873.55	-2903.87	8995.26	-8672.58
479.916	-5116.91	3198.26	-10057	-2749.86	823.096	-1105.72	-3491.41	-5940.92	-4889.99	4075.22	2256.21	180.633	3352.47	-3055.52
-1524.26	-5680.26	-571.434	-9726.15	-6143.63	-327.988	-765.951	-2188.08	-6178.2	-4071.73	3244.77	-5175.4	-236.699	-1828.99	-9289.5
1401.26	773.445	-1524.36	-2120.37	-12335.1	-352.172	3710.56	-5943.91	-5302.15	2344.35	-1296.26	3044.2	-3636.04	5658.19	390.816
-2873.29	-7492.47	-442.916	-12268.9	-7572.31	-2234.87	1279.59	-2181.85	-5780.22	-9511.98	1611.43	-7124.62	-5216.43	-2085.73	3148.37
-1330	-2625.29	-4599.79	-10115.3	-6058.35	-5937.84	-8647.62	-7587.21	-5521.16	-1302.62	3583.07	-6453.99	4511.02	-7298.92	-3401.73
-2511.12	-8673.53	976.158	-5734.21	-1267.51	-2396.97	-3335.19	-4993.6	-1998.18	-2876.84	-2929.39	1884.74	-2747.17	-7390.58	-8896.21
-3075.64	-12640.4	150.998	-10644.7	-7275.83	673.557	3375.53	2496.51	-577.262	-1358.53	-5234.45	-5837.32	-351.631	5270.23	-2701.08

-4688.05	-6334.12	-462.109	-10525.6	-4387.13	-6475.4	-484.871	-87.1211	-1915.62	-5460.38	781.23	-6171.72	-209.855	1206.91	-7621.2
-3082.54	-5310.51	2346.47	-2205.12	-6412.92	-1241.4	2452.18	-4122.39	-4854.98	-6415.79	8704.67	-7331.25	-3527.07	-2000.28	-13359.4
2229.83	-10309.6	-5108.94	-4309.05	141.203	-5906.67	-1922.71	1258.39	-3147.4	-3675.38	8435.49	-4832.46	-4370.07	-640.21	-1251.83
2098.69	-10363.5	-2615.97	-2985	-1591.08	-8104.06	-3047	-2157.65	-3993.18	-6443.53	7831.89	-5428.57	-1694.57	-5475.74	-8549.49
-2948.74	-3878.83	-3434.54	-11646.6	-6958.89	-4859.08	-194.27	-7067.74	-5022.38	-2890.39	3404.57	347.802	-363.924	2223.51	-6220.66
-3954.03	974.734	-602.193	-6951.82	-8105.2	-4486.01	-704.189	-8308.12	4081.1	3005.04	-6528.8	-2525.56	-4317.82	-1895.4	-4138.04
484.535	-12183.7	-1668.46	-6670.85	-6808.14	5534.49	-8054.7	-5534.75	15464.2	-2170.84	-732.775	-13384.4	-519.326	9140.3	669.631
-2761.54	-18508.1	4538.05	-4443.99	-9031.19	5458.77	-4748.37	-2107.91	-6218.8	-9791.12	-1659.32	-4908.98	2541.26	-5121.89	-1046.11
-1037.25	-11255.5	-1905.94	-10269.4	-5171.58	14954.8	-132.158	1089.6	-9165.13	5281.41	431.549	-3478.79	19276.2	-1084.99	-10404
-4279.96	-2770.66	-2567.93	-11167.1	-5669.45	2864.65	-6533.31	7865.31	3281.4	13215.1	-331.32	-2315.07	9131.3	3732.37	-4431.89
718.5	-4738.57	2882.52	-10910.8	-6931.96	129.283	-7448.42	-7065.57	-9469.44	-5152.07	3328.17	-419.992	-63.8574	-3136.07	-5966.93
2517.93	-7139.76	-1127.24	-10537.4	-11421.8	-2682.38	-6535.57	-7196.11	-10009	-6788.36	1668.83	1143.06	-2718.11	-2658.65	-7391.83
-1696.29	1861.29	-2491.07	-11499.1	-4578.41	1357.41	-8376.62	2356.66	-1381.28	-2164.64	-1666.21	4403.12	-491.445	-248.809	-9306.15
-1757.85	-3400.74	-6119.8	-13551.1	-6336.55	-7092.08	-3301.96	-1270.35	-5652.7	4545.13	-3328.29	-5841.31	5836.37	-422.866	-6047.39
-4721.89	-7620.71	1586.36	-14651.6	-3179.37	-3903.27	-5103.77	-289.715	-2634.67	-8219.23	-1434.9	-3755.65	-3298.17	-1394.67	-8915.79
-6761.99	-2085.48	4291.5	-5101.07	-11222.7	-3171.41	164.963	4414.03	-2259.61	1941.76	2199.8	-3671.02	416.273	-3648.05	-7525.82
-2015.72	-4961.94	-3254.79	-13275.8	-11894.8	-9850.19	-3584.78	-1464.11	-4384.7	-7656.33	5634.11	-9166.24	-4608.26	-1467.55	-8725.92
-5739.53	-10014.6	-7152.21	-11635.4	-9775.13	-5360.17	-2700.07	-4770.49	-672.947	1628.53	5695.53	-3055.9	-6587.32	5952.82	-14378.1
-2735.66	-2745.57	-3186.16	-9662.26	-3852.77	-3299.31	-5975.12	-4334.3	-1901.67	-6643.49	6208.18	1580.17	-5334.99	200.48	-160.354
5342.77	-3457.85	-2750.49	-3393.52	236.682	4769.46	-2244.28	-7668.07	-5702.31	-3932.4	-2160.77	-9689.17	6718.39	-2907.62	-1156.18
-2605.65	-8360.01	-2946.89	-14760.4	-6533.42	6160.82	1388.12	-6926.95	-5692.02	218.828	-2525.66	-46.7031	3361.67	-10971.2	-3683.9
-3351.78	-5536.47	-632.236	-3037.38	-9638.26	-3500.77	-3796.19	-1211.55	-4509.61	-2987.88	-1882.38	-10128.5	110.355	-3295.32	-5220.11
-2931.01	-2906.38	-1226.95	-9469.64	-11973.1	-9838.05	-5680.68	4742.27	-4769.46	-3971.06	4955.45	-2881.08	-5046	-3710.16	-4428.38
741.693	-9008.25	-2142.27	-13419.5	-9928.15	-8008.65	-2828.27	4769.98	-5434.05	548.819	2723.3	-8200.2	-1897.18	4617.91	-6965.54
-5546.59	-6258.82	-524.461	-11973.4	-9814.68	-5493.96	-1186.7	30.4922	-9449.45	-2873.18	2174.74	-3321.24	-4122.47	-5764.46	-14085
-8507.07	-12001.5	-6843.47	-13597.3	-7420.48	-4896.55	-7220.25	-3581.18	-2872.48	-7037.11	3409.13	-3813.17	1618.17	-1895.33	-6534.46
1588.84	-2021.18	-2427.37	-8650.2	-9913.62	7309.05	-2925.6	-5452.16	-254.053	-6465.76	1180.43	-519.283	-7487.01	-112.691	2086.07
-3857.48	-3712.72	-7576.75	-11962.7	-5036.38	-4741.77	-1141.23	-6749.19	-3780.83	-3465.18	-1102.22	68.709	836.049	-1045.48	-10322.7

-4295.22	-4889.01	83.4004	-11516.6	-6447.8	-1006.71	-5540.91	-6315.36	-2320.73	-1015.87	949.318	-11564.8	-2354.76	-9355.9	-6235.99
-2699.02	-3616.76	-2630.32	-10248.8	-4635.71	-1859.52	-7753.99	-4996.51	1865.27	-1295.6	4110.7	2337.17	-3598.72	-4266.57	-12300
2026.71	-4286.21	-3326.21	-11511.3	-7662.27	-7014.12	-2901.47	-5661.22	-6416.58	-5219.46	1810.44	-6901.37	4079.47	-2293.61	-9739.18
-581.061	-134.74	-249.303	-14675.7	-12677.8	-4671.44	-3838.03	-3788.04	-5140.43	-2655.53	1776.48	-2414.79	-7638.67	-4422.14	-5760.76
-3228.63	-4461.83	4452.8	-12979.2	-15129.9	-8185.95	-2240.3	2656.02	-4227.46	-2006.59	1168.04	-4530.08	-4692.61	-2944.65	-6927.55
-977.1	-950.648	-345.553	-4292.64	-5772.33	375.004	-3426.98	-3505.49	-1370.26	-6729.17	-2527.1	-6961.95	-1105.1	-4016.97	-10501.2
-3979.41	-4592.93	-5984.21	-8936.61	-15452.3	-208.4	-5060.91	-4796.34	1604.76	-1036.55	-1734.56	-7584.57	-2745.58	-2595.21	-9757.37
-5786.17	-6681.57	-3953.1	-14378.7	-9178.52	-85.4023	-8541.54	-4524.21	104.484	2880.7	1537.41	-4229.2	8094.69	-3896.45	-4120.25
-1829.27	3335.74	-1933.2	-12076.2	-5388.02	-5106.02	-1633.96	-2604.61	-6899.26	-2618.64	4738.8	-1980.07	-2864.14	-6420.53	-8117.27
-6165.6	-6269.51	-6021.05	-8454.55	-10215.5	-92.834	-1938.82	-4728.24	-6583.25	-1322.45	-1433.46	-6652.19	-2137.6	-3781.77	-13276.4
397.1	-2770.36	243.074	-5576.8	-11055.9	-4207.2	653.682	-3455.03	-7711.76	94.4531	-2099.07	-163.017	-1182.78	-7447.53	-6577.23
-1259.48	-16200.2	-5612.29	-6163.4	-536.973	-1658.81	-6637.54	1315.15	-6043.33	-4308.22	1142.87	-4087.59	-2321.8	-7998.1	-4183.54
-9156.8	-5152.29	-5924.84	-10747.7	-12802.5	-6383.32	-3115.07	-4790.17	-3606.41	-4777.02	-610.682	1692.97	-3882.94	-6578.42	-7668.86
-5571	-826.631	806.031	-11747.5	-8963.75	-4242.09	-10697.9	-2063.42	-6513.48	-277.23	-6287.52	-6025.87	-5174.33	-7200.23	-6029.76
-5185.29	-5889.32	-553.631	-10070.9	-8394.36	-1443.55	369.184	-4447.06	-3603.34	2105.24	1036.87	-5013.64	-5197.85	-5556.63	-6756.09
-11391.4	-4641.13	-4465.59	-9149.28	-10670.6	-8001.39	844.195	-3857.43	-1667.41	-1456.5	4881.15	-6972.61	-1380.69	-371.21	-8487.82
-908.393	-4031.25	-5991.13	-14334	-8716.22	-5750.58	-6755.31	368.969	-7346.63	3744.82	-2353.21	-1996.03	2923.19	-4924.83	-3895.75
-5931.01	-3209.93	-1951.56	-9763.01	-9565.19	-5120.89	3512.62	-2763.75	-6963.91	670.499	-3674.6	-4211.51	-1.0957	3682.9	-2450.85
-3598.95	-4970.81	-6135.9	-10271.2	-9888.19	-264.34	-9473.32	2794	-214.604	1931.49	2026.82	-673.136	-6693.29	-6901.53	-4486.66
-4139.14	-1246.86	-3148.17	-20119.5	-6740.29	-4280.06	-414.949	-4902.93	1815.59	-10413.9	-3685.5	-6859.95	-6436.55	-9703.18	-5335.84
-893.098	-1906.34	-1143.05	-5075.48	-8304.25	-3525.54	-2549.38	-261.328	2927.15	-2877.91	2454.71	-2424.22	598.002	-6239.64	-10872.9
-5669.7	-9309.81	-1668.57	-6659.67	-9661.99	-8355.68	-341.889	-13102.8	-4292.67	-5435.35	4429.62	-2823.43	-2415.96	-3542.03	-8916.54
-4821.9	-92.9766	-5681.12	-8878.69	-11343.4	-8734.91	-4540.11	-2514.78	-2803.45	-3901.08	2152.46	-6471.98	-3649.96	801.012	-9171.92
230.186	-3549.15	545.615	-7554.74	-3271.33	-6206.3	-4270.5	2087.9	-2773.5	-187.716	6340.06	-4593.14	-1241.06	-3878.85	-10624.4
-5748.18	-5527.05	-2282.48	-10702.7	-7095.96	-4808.44	-5426.42	-9940.07	-9025.64	-413.977	6613.67	-8720.33	1592.11	-3300.43	-2361.74
-6141.94	-9593.08	-3511	-14527.1	-11795.2	-4599.83	-33.084	-4659.47	-2181.77	-4273.51	492.469	-5788.29	-5911.87	-4148.61	-6263.86
-4791.9	-15598.6	2409.74	-7656.97	-4830.96	2970.57	-8207.52	-5536.72	-5258.58	-7073.86	-1296.29	858.801	-7187.35	-8446.71	-4820.93
-1672.63	-4256.87	-78.2227	-7608.11	-5531.76	-3851.58	-7653.62	-7215.04	-9306.96	-1495.83	-2470.64	-1346.35	1026.74	-7994.03	-11939.1

-2985.24	-2595.74	2655.29	-12243.7	-12353.8	-687.832	-2701.96	-7957.06	-3493.57	-2764.21	-3089.09	5145.28	6419.51	73.1777	-7054.38
2640.32	-12303.8	-7847.46	-8811.74	-10512.2	-4414.21	-3966.51	-6712.81	-381.766	-6429.13	-625.15	-501.855	-7087.82	-5049.63	-3341.44
-1205.35	-7566.94	-9224.61	-14530.4	-7417.51	4037.2	-5978.47	-8896.83	-12088	-2056	1843.56	1357.94	504.693	-6438.81	-7763.13
-437.84	-7569.22	-4288.79	-11150.1	-1457.61	-6078.23	-755	-9083.56	-2599.11	-4413.95	2421.97	-303.927	-2284	-4052.51	-5986.1
-4658.08	528.508	-3127.15	-6511.42	-4576.24	-1659.67	-13213.2	-1000.25	-5488.77	-7559.32	3266.76	-5280.83	-5737.77	-5199.21	-6569.25
-5137.97	-6083.59	-535.311	-5611.91	-12093.4	-6838.74	-1957.44	-5058.29	-6385.37	-1545.9	2049.93	-4836.38	-4957.43	-2800.71	-5489.46
-2253.15	-4324.21	-3159.97	-9290.11	1004.99	1114.61	-3002.5	-5529.76	-6323.96	-2865.72	-4488.1	-4646.25	-9998.55	-2784.8	-5874.9
-5874.64	2333.85	-122.572	-15175.2	-10591.1	-2752.6	-5630.26	-1574.33	-8952.12	-10871.5	2997.46	-6726.5	-7766.04	-3685.1	-10226
-190.576	-6758.48	-4440.79	-11466.3	-4835.63	1796.85	-1772.79	-2102.84	2213.41	-4484.26	395.641	-5922.97	-8427.46	-8077.48	-2646.74
-4411	-6565.6	5561.23	-6992.37	-7922.91	-3009.11	-5251.98	-3437.04	-2574.84	-5375.78	1443.6	-7039.8	-5772.43	-1636.16	-1463.3
-6459.22	-3342.09	5030.03	-7294.7	-8491.69	2550.43	-5590.88	-6067.09	-7121.87	2136.15	3463.56	-3439.97	-473.635	-6381.18	-4399.85
-4158.31	-221.549	6644.46	-8696.32	-5014.84	-4828.77	-174.182	-13931	-5402.25	-4991.06	-5146.59	-1518.94	-1810.16	-10791.8	1416.16
-1589.94	-4587.7	2765.33	-13561.1	-5620.47	-9565.95	-1486.51	136.047	-7891.88	965.076	-3161.34	-2292.68	-1868.41	-4810.88	-4587.88
-9242.3	-5921	-7782.72	-13315.7	-5478.62	-1128.24	334.379	-6705.5	-3491.1	-131.918	-2877.55	2632.59	2816.63	-3371.38	-3376.92
-6906.77	949.463	-3792.79	-9836.62	-5782.98	-2211.63	-3005.53	-2751.39	-1189.54	-2940.25	-2419.43	-9449.53	-4655.18	-5863.15	-6995.44
-7350.79	-8782.67	-7061.4	-13658.9	-3037.81	-3535.63	-3072.52	1674.86	-2350.97	-2719.52	-239.51	-4051.86	-1926.43	-3060.77	-7825.5
-8254.37	-4844.54	3403.39	-8555.23	-10476.9	-7324.08	-3212.51	-6151.25	-13851.3	-828.756	1398.19	-1046.25	-872.676	-1134.3	-9272.52
-1473.41	-7642.94	-4030.79	-8512.37	-6453.15	3551.62	-4136.24	-2355.28	-4004.13	-3185.04	3625.62	-6443.53	-2657.79	-936.12	-10806
-5640.89	-10722.7	-7443.62	-9747.32	-3867.71	1677.04	1837.95	-6884.31	-1584.1	-2194.74	6307.27	-7431.87	1749.58	2624.61	-13928.6
299.361	-4380.46	-2152.81	-12217.6	-6328.48	315.377	11.1367	-8047.25	-4978.48	-2556.26	-3634.31	-3439.97	2499.16	-3880.41	-4212.79
-7329.97	-328.557	-11863.1	-14352	-9845.64	-999.143	-5038.24	438.244	-6632.69	-1813.16	2530.58	-6966.62	3711.31	-2203.31	-4338
-3435.75	-3985.14	272.562	-17802.6	-6294.5	-1181.88	-94.8438	-3268.3	-3881.04	1330.89	967.412	-5350.27	-3813.77	-3403.59	-11561.5
-1559.25	-5548.59	-1107.33	-8985.44	-6414	-4960.34	-4979.76	-3198.37	-4887.71	-1346.01	-1719.63	275.25	-4723.97	-2290.64	-6191.19
-8452.41	-136.836	-3295.37	-15274.4	-10588	-2525.05	-800.965	-3652.07	-3779.71	1007.99	5584.67	-718.633	486.254	-2960.84	-3006.58
-8907.41	-4099.5	1273.29	-2300.2	-8895.3	-7831.02	-3794.93	-2248.44	-11586	-6590.28	2886.09	1624.32	52.4082	-2515.52	-2877.17
-3549.28	-8390.08	-3776.94	-9926.88	-7472.48	-2848.7	-1286.27	2942.87	-2393.33	-7164.52	2955.45	1536.23	2915.27	-1920.75	-5659
-2817.64	-8295.86	-10958.4	-16247.8	-7358.34	-1451.2	-5998.59	-11063.7	-319.525	-1365.25	1567.44	-8278.08	-3029.58	-5396.03	-4076.54
-2549.74	-4594.63	-833.6	-13156.1	-5414.98	2876.63	2011.9	-5298.58	-2714.42	-3059.71	1057.83	-5616.51	338.961	-9007.74	-13576.8

-1832.14	-3998.35	-3381.13	-10140.3	-5063.38	-2426.93	-6007.38	-9105.01	-874.777	5441.35	2122.89	-2481.01	2870.98	-1085.27	-10271.6
-1299.5	-3932.53	-3432.11	-7811.05	-3859.56	-8041.93	-4829.04	-10288.1	-6571.04	-701.759	7150.7	-6807.98	3694.83	1311.34	-5141.49
3085.19	-8472.82	2440.81	-11507.1	-6321.43	-3359.03	746.201	-4857.5	3096.49	2913.9	-1107.19	-1286	-3542.59	-5432.72	-4844.58
-2909.6	-5416.82	-1424.57	-7644.14	-9518.58	-2237.53	-4970.97	-2154.29	3261.18	-6912.07	2672.29	-5671.61	7455.67	-5845.37	-8916.27
-5552.05	-5414.79	-1826.38	-4925.85	-13300	-4830.77	-467.922	3063.71	-3212.58	-2998.67	5913.62	-4324.78	-2148.48	-3434.2	-6770.42
-5304.66	-3500.71	3009.21	-7975.09	-7412.11	-2429.62	153.664	1331.17	-858.582	7780.87	3693.1	-248.059	2851.88	-3763.07	-5520.55
-1333.4	-6614.14	972.461	-5096.75	-9316.46	-4080.65	-5747.57	-5403.48	-1882.5	-7412.27	3026.66	-7938.54	6988.65	-4935.16	-899.826
-454.729	-3314.36	2267.23	-8838.18	-8824.4	558.24	429.48	-6365.9	-4666.15	-9116.73	1057.37	-8490.46	-5581.08	-9890.8	-5707.23
-4876.97	-10614.2	-4276.71	-11224.8	-14186.4	-9834.72	-1034.97	-3939.37	-10320.1	2071.49	3257.82	-7105.52	-127.359	-5349.48	-7529.14
1283.23	-6261.92	-5460.32	-11284.7	-8231.7	-3075.23	-3790.29	-7328.37	-6512.53	-4734.19	2170.89	-2139.53	-1958.94	-1456.23	-9845.99
-5191.72	-3616.64	-593.648	-15216	-12186.4	-8323.65	6885.69	-6228.67	-3541.53	-7852.01	1172.29	-789.72	-2952.45	-3434.77	-10282.4
-2779.87	-3221.9	2090.19	-13825	-5166.21	-8766	-3836.92	-4847.28	-3293.85	-4092.67	-1055.9	-6426.15	-5669.76	-4386.34	-9795.87
-5847.6	-9542.99	-3856.62	-10228.9	-10839.7	-5289.67	-1078.07	-7438.81	-6970.02	-1972.35	-2171.39	-1562.45	-725.789	-868.714	-10236.2
-3673.18	-2276.99	-5411	-5653.32	-7968.36	-6775.62	-2876.26	-4437.14	-7569.04	-3516.07	1591.26	-1740.68	-1012.82	-3105.17	-7114.31
-1515.33	-4533.82	-5995.78	-9784.9	-9936.75	-4393.88	1382.92	981.598	-3985.96	1758.22	1501.93	-1707.53	-5068.29	-4544.1	-6232.63
6588.52	-3033.25	-2610.69	-12555.8	-10980.1	-5284.04	-1530.1	457.035	-3677.06	-8089.46	-6475.2	-1494.57	-1812.72	2718.14	-12051.9
-3271.96	-1450.39	-6686.65	-11196.2	-8990.6	443.533	-4316.19	-4984.85	-1417.53	-6388.69	4521.61	-4193.09	-5811.53	-7809.81	-7882.52
1239.97	-1652.56	-2918.43	-18559.6	-6512.36	-2628.58	5504.46	-11234.1	1534.93	-3727.82	1309.11	-6192.1	-3747.64	-5537.67	-5821.72
-53.8184	-6669.12	-3115.23	-13394.5	-1838.26	-9857.99	1386.65	-9376.7	-5466.04	-8230.16	112.148	-881.098	-4030.71	1841.34	-5674.63
642.895	-3999.33	2377.31	-9602.17	-5421.08	-1111.96	-3988.86	-1803.59	207.428	-5115.84	-228.41	2082.63	-5572.52	-3905.24	1455.23
5410.13	-5026.46	218.477	-9962.88	-16142.8	-8766.89	-3832.91	-4509.08	-4711.6	1390.2	2613.29	-2326.82	-7071.42	-5233.05	-6914.63
-3298.9	-390.064	-9039.93	-13303.5	-10552.7	-9139.85	-8856.72	-3461.8	-13135.9	-968.751	5384.85	-80.8594	289.27	-2092.17	-9297.93
1664.29	2980.79	-1979.1	-8487.52	-6030.55	-2258.78	-3642.22	-4346.32	-195.273	-9941.57	3978.88	-6037.35	-1534.13	510.342	-6667.42
507.916	-8509.85	615.598	-12736.8	-7559.19	-3697.93	2080.81	936.324	-1975.55	-11579.5	-2942.85	-5808.37	6772.97	-3508.57	-5476.32
-5029.86	-2546.35	-4298.52	-18499.8	-6458.46	-7826.92	-3231.62	-2780.66	-2979.52	-6700.51	-1572.61	-5802.44	-3604.98	-4198.88	-9144.62
-2301.82	750.027	-6290.8	-5653.35	-10812.3	-2276.3	-3939.03	-8197.7	-3749.86	-3264.18	-1409.91	-5974.72	-608.043	-3353.86	-8777.02
-5542.59	-1602.54	-6236.72	-6082.14	-13134.9	-1861.16	-6155.21	-8003.58	-3218.49	-3885.17	-5269.37	-7145.74	-1237.43	-7338.61	-4199.71
470.301	-7801.95	-1897.04	-7421.79	-6886.91	-316.479	529.566	1750.06	-4027.88	-7895.62	-2278.18	-10031.9	-7129.58	-7455.3	-6130.96

65.582	-2799.37	1359.57	-9214.62	-10459	-5362.61	-2453.83	-3311.23	-2996.3	-3616.22	3634.84	-8257.88	-6125.74	-3119.99	-3594.14
-3427.29	-2344.12	-1520.94	-15879	-12707.5	-399.348	-261.191	-3955.05	-7158.25	-3390.29	2377.9	-6709.46	-7459.72	-3603.17	-8284.4
-7448.4	-1745.63	-8442.17	-10921.5	-4864.6	-5546.04	-5134.35	-9110.16	-4740.76	-1661.27	-1607.35	-4421.81	-745.822	-4551.77	-630.58
-1367.37	-7098.76	-732.381	-11323.9	-8446.76	-6135.11	-6341.96	340.604	62.4629	1462.32	-2526.32	-3118.13	-5713.45	-122.393	-955.573
-3699.28	-598.219	8205.8	-12398.3	-10225.5	-1929.83	-3504.47	-2236.96	-4989.29	-4355.19	6525.48	-8135.95	-254.77	-2045.71	-556.773
-3802.69	-8361.1	-3835.79	-12111.7	-11369.4	-7718.89	-1813.83	-7081.48	-2776.3	-2407.29	-3722.98	-2355.47	-4320.71	-2375.07	-6432.62
-4819.59	-7396.61	-2978.8	-9962.22	-10312.1	-6005.06	-6064.19	-8031.7	-6284.58	-4942.17	3479.15	2814.78	-650.18	-2951.51	-10020.4
1181.34	-6914.3	-1824.43	-12236.6	-8144.38	-5558.44	-7906.56	-649.268	1264.96	-4384.48	4185.08	-564.127	-3623.9	3133.59	-7791.04
-4907.65	-6427.28	582.375	-8285.99	-12465.6	2954.76	-671.164	-2423.43	-6485.58	-4785.3	476.25	-1978.26	-2568	-8264.78	-9159.7
4167.2	-2156.89	-2774.85	-9657.68	-14498.7	614.705	-8968.39	-6845.79	356.119	-6250.24	-2831.98	-6026.26	-4111.43	-4130.33	-4278.82
-1648.19	-4888.56	-22.7852	-14368.1	-11693	-6233.73	-2752.84	-2604.09	-1842.16	-2888.32	2860.9	800.836	914.473	-7144.58	-10830.6
-1074.43	-3933.84	3481.92	-9584.37	-4366.4	-1850.37	-1678.14	-7041.25	-1713.16	-116.74	1193.13	-3301.33	-6553.15	-2457.97	-12265.2
-2487.24	-6967.63	3293.63	-11097.9	-7873.8	-4641.96	-1466.46	-2126.12	-1667.64	-2812.88	-3602.15	-5123.53	-6144.11	-5422.52	-4533.6
-3782.81	2982.89	-830.459	-13420.4	-7819.93	-3895.31	883.395	-5310.58	-5510.18	-5363.19	135.234	-11532.8	-818.654	-8888.54	-740.939
-2164.44	-7346.37	-2687.31	-6142.8	-7463.49	-3195.26	-4243.05	-3067.81	-6765.38	-4185.03	-2861.12	-8313.45	-1214.44	-6105.79	-6639.87
-4543.55	-4625.73	-3429.01	-9299.67	-10054.3	-10047.5	-6742.34	-9860.96	-5186.67	-7080.69	-3890.23	-6590.67	-9908.58	-1147.75	-9107.12
-2583.8	236.279	5513.94	-8591.06	-9907.78	-7660.93	-733.205	-2924.61	-2831.65	-5263	-2105.78	-133.437	-3096.23	-5973.46	-12452
-6690	-1595.88	-4193.97	-10069.6	-9950.98	-983.168	-2106.84	-12567.4	-3789.01	-4068.04	6352.4	5135.05	-379.291	-4302.4	-17272.3
-2865.24	-3024.11	-8130.21	-11068.2	-2826.73	-950.83	-4916.53	-4846.56	-1067.07	-69.0283	6196	-6447.66	-8765.11	-2430.61	-4553.2
2572.49	-7356.06	6444.65	-11940.1	-9739.92	-6027.25	-2011.56	-4747.16	2076.83	2342.84	-1141.02	-6796.39	-8185.23	2157.2	-4945.43
-4181.66	-9264	-2671.57	-11100.5	-8701.4	-6946.7	-2955.45	-5007.46	-1657.26	2205.72	-3426.23	-4279.26	718.6	-6031.06	-9621.65
-3492.34	453.363	-4275.55	-12179.6	-11411.9	-7600.22	693.646	-5001.51	-4360.59	-1631.95	4216.3	-9430.93	390.465	-7664.95	-4201.74
1369.23	1046.75	-1130.06	-10444.5	-8197.37	-7284.28	-10125.7	-3969.03	-5467	2144.27	-1412.55	-7629.96	-1630.25	-8347.18	-5875.12
-4283.7	-932.061	-5919.63	-8985.21	-7561.82	-4023.08	-5318.56	2190.67	-5379.1	-3254.91	-2452.4	3.88965	1335.69	-1155.52	-6058.96
-1298.75	-5888.35	-395.268	-5210.61	-14016.1	-9088.66	-4690.69	-5256.53	-3104.59	-7700.94	-1972.62	-6471.31	-5825.68	-1122.07	-11064.7
2559.76	-3632.22	-6806.39	-8651.85	-12087.4	-623.406	-4792.14	-6355.72	-2708.98	-11339.1	-2159.53	-3069.79	-826.578	-7737.26	2189.48
-6582.4	-7489	-2004.69	-10219.3	-10490.1	3957.47	-2004.49	-8472.47	1317.93	-739.5	-5115.42	-117.925	639.176	4135.74	-3580.63
-4579.88	-83.8027	-4072.92	-6884.87	-12142.9	-3220	-2595.87	-4873.64	-4897.3	4019.79	-764.578	-7740.29	-1494.47	-559.947	-3255.85

-7346.88	-1585.22	-12027.5	-12070.2	-6840.77	-5633.74	-1959.01	-6094.21	-7048.52	-1467.14	36.5059	-7539.48	-1149.23	-7103.07	-7399.18
-1163.4	-6949.23	1434.04	-13066.6	-4912.96	-3488.76	-3147.12	-3357.07	-9507.14	-5989.79	1447.02	-2822.08	-2811.53	-4565.69	-4050.37
-1913.58	-3702.75	-4521.27	-9791.72	-8725.04	-6703.62	-2977.84	-3921.46	-6536.08	-1396.05	8.19922	-5570.07	-4550.8	-12036.9	-10910.1
705.078	-8823.46	-3111.05	-2447.32	-3194.15	-3215.81	-7429.7	-2895.35	-5432.3	-3416.29	3798.47	-2664.53	-7283.99	-3581.74	-5576.97
-4112.55	-3705.88	-3633.59	-10700.8	-9802.91	-1383.31	-4831.9	-765.799	1158.45	-3445.46	3286.76	-4250.55	-8861.45	-7139.6	-480.615
-3816.05	-5974.16	1209.83	-13518.7	-7353.93	-4434.62	-8552.38	-2353.97	-6302.88	-1379.36	-236.621	-1957.57	-4325.04	-8757.62	-5800.96
-8887.84	-7718.66	-2263.04	-6903.59	-8543.83	-3981.81	-6111.83	-165.531	-7081.4	-7110.01	-4466.71	-5342.8	-6756.83	-6704.52	-9698.92
-5714	-7676.66	-4230.61	-14404.1	-5910.76	435.906	-2117.72	-8043.54	-3966.92	-7632.55	3529.3	-8637.86	-3107.74	-5302.83	-8057.06
-9376.78	-2155.95	-5426.22	-14268.6	-10275.3	-1343.93	-6805.36	-5697.56	-2886.54	-3042.32	-1332.78	-9325.26	-5658.31	1907	-13194.2
3552.62	-10757	-3840.16	-12653.4	-3195.31	1771.59	-8416.44	-660.486	563.984	-4442.45	1027.9	-13725.8	1444.44	-8874.36	-3950.5
-3836.34	-4043.22	-2340.85	-15014.6	-1931.1	1619.52	-3181.09	-1977.21	-2254.79	-2179.85	-2073.32	-2131.73	334.221	-7420.93	-7941.35
-3622.3	313.521	4525.65	-10095.8	-4770.48	-2429.62	-529.857	-5856.02	-7630.68	603.326	2421.66	-2115.34	813.906	-373.218	-6829.47
-5862.21	-4940.44	3190.11	-5460.6	-490.119	-5165.68	-5213.83	-1898.44	-9152.23	275.624	-4635.28	-5107.56	-5411.79	5655.55	-11414.4
-7016.59	2159.66	-3293.3	-8315.93	-6468.25	-3909.15	-5995.77	-3408.91	-4618.23	-3556.73	-6800.22	-4102.83	-3842.62	-4597.58	-6465.33
-3110.16	-3438.83	594.812	-10419.8	-9916.3	-389.752	-10462.5	-6342.68	-1872.74	2640.92	-972.07	876.387	-1947.16	1715.6	-3604.61
-4630.61	-6178.39	-1720.63	-17036.2	-9702.15	-4075.91	-6236.85	-8447.53	2118.78	3248.58	2076.21	-3387.62	-7407.69	-3088.35	4832.57
1349.47	-61.8125	-2226.84	-11226.3	-11669.5	-8442.49	-3143.35	-826.119	954.777	-1985.76	3381.69	-8020.31	-3108.26	749.641	-10836.5
-7072.99	-8985.69	-1623.71	-12101.8	-9039.67	-5962.99	-6938.29	-5873.85	-6137.7	-2380.3	6227.44	-3704.16	5298.81	-3576.08	-8013.59
-1024.42	-1085.02	-5748.79	-15426.4	-13095.9	-5007.51	-6764.69	-1424.9	-8111.29	-2417.08	-4499.91	-7418.54	426.789	-6835.16	-3843.06
-10044.1	1039.4	5553.01	-16570.4	-9347.54	-3887.3	-8610.38	-930.65	-6010.45	-4083.33	-1761.6	-4153.22	-5639.73	-175.666	-11948.9
-5289.71	-2926.21	-1872.8	-4172.57	-3326.78	-5799.89	-7153.78	-1545.18	-5170.41	-6811.92	725.607	-9000.26	-5361.76	68.1973	-3144.49
-1912.71	-9433.37	-4225.76	-10624.2	-8235.83	3234.48	892.391	1600.2	-9147.39	-10140.8	-2631.31	-7894.03	-24.0527	605.676	-4734.74
-5251.28	-4764.39	-4321.33	-11323.2	-4697.65	-2034.05	657.309	-700.352	-8809	-2320	3536.32	-8701.33	-5637.13	-5541.3	-9115.34
-6736.89	-5799.6	-3007.17	-9184.95	-8860.99	-4452.95	1265.05	-4627.02	-5421.91	1928.35	6099.85	-7578.66	-3976.77	-1003.81	-7296.58
-5180.48	-4655.01	-2834.66	-10373.8	-4134.93	-3287.75	-3055.02	-4074.17	-1705.18	-9411.35	6307.85	-5653.67	424.566	-3568.18	-12444.9
3586.21	-2886.94	-4674.18	-3548.53	-6697.42	-6172.35	716.141	-3464.82	-2311.76	-1743.32	5263.06	-1483.34	-7422.91	-124.766	-14528.8
-6531.24	-3324.72	-3010.12	-3790.63	-7806.03	-4644.31	-3410.38	-4460.8	-3791.75	2401.96	4128.79	-1048.61	-4935.47	-4728.67	-8390.46
-4171.48	-4913.8	-3960.5	-5122.99	-13947.9	-4746.9	-1225.1	2929.61	-6428.68	-4793.16	-582.807	-5238.71	-9501.8	-9617.13	-11785.6

-6449.64	-3956.68	-2413.62	-6442.99	-7731	-379.463	-7541.05	-12914.1	-6233.52	-2377.75	-2256.84	-10109	-6746.5	-2890.54	-6918.44
-3907.74	-9101.69	-3357.96	-13013.4	-9615.29	-1195.83	851.973	-4917.52	-5482.24	-5490.67	-768.986	-5184.83	-5719.78	-4460.71	-7695.84
-597.791	-8341.06	-6772.85	-7380.78	-9280.2	-1074.86	240.639	-1950.03	-8278.36	1195.44	3299.68	939.537	-8276.46	-5604.64	-5349.47
-6526.21	-7040.26	-1003.45	-9262.12	-9178.5	1166.22	-2671.76	-4428.75	-2855.13	-432.662	2547.41	-21.7227	-3203.42	-4360.03	-3404.28
-7403.32	-6708.75	-5427.85	-8612.46	-4179.94	108.594	-1769.44	-1224.55	-8397.69	-3813.6	1381.28	4921.79	-6240.42	-941.361	-9110.63
504.951	-6192.55	831.861	-7861.69	-7443.11	-933.58	-4601.66	-1197.63	-5418.75	-836.03	-784.293	-977.684	-4056.82	-4391.63	829.52
2783.61	-4111.16	4126.54	-7287.68	-8758.38	-3897.47	-1119.99	-4489.77	-8617	-8237.77	93.8027	-8774.11	-13193.7	-9941.08	-10141.9
-6402.47	-3102.5	-600.684	-11993.4	-2853.2	-324.715	108.957	-5532.45	-4229.18	-3681.14	5151.97	-4820.38	-9159	-3513.89	164.34
-1389.81	-6650.62	-6038.26	-15454.7	-6321.49	1008.22	-5876.53	-3818.17	-3083.05	-7170.65	6039.64	-5021.35	-5866.48	-6503.16	-5730.86
370.16	-3735.1	-6687.81	-12316.1	-11720.1	-3715.1	-5126.26	-3182.52	-1836.92	-6213.06	12123.4	-3477.1	-6741.32	-5100.98	-6751.31
637.275	-2390.2	-932.988	-10604	-5794.77	-10829.1	-6907.61	-2611.66	-10356.3	-3734.13	513.273	-6836.3	-4396.21	-4672.84	-6689.56
-8646.16	-7592.97	-1399.26	-12841.1	-9657.76	-133.963	-5161.31	-8460.31	-2583.97	563.443	-139.514	-6818.56	796.912	-7056.34	-3645.67
-625.48	-7831.56	-5085.57	-8990.22	-13511.4	-4557.73	-7314.54	-1215.52	-5641.82	-3044.68	2990.9	-7852.05	-1762.84	-9873.1	-10035.4
-2561.23	-3279.79	-1952	-14593.8	-14658.7	-1010.01	-679.918	-1026.55	-7017.58	-4759.96	-3031.32	-4810.73	-2984.29	-2768.17	-2000.52
-5585.14	-8310.87	-8843.62	-12641.1	-8330.53	-7221.66	2606.81	-433.252	-4759.63	-6076.9	330.742	-2536.36	-9213.84	-1786.69	-12691.6
-2625.75	-4354.85	-2284.38	-12045.2	-4832.7	-1375.24	1799	-1566.24	-3290.42	-4818.75	-1268.99	-7622.27	-2301.33	-6102.17	-5269.99
-1768.12	-1466.01	1532.56	-13160.1	-7086.81	-5453.39	-7213.34	-271.586	-9280.87	-6861.45	3135.19	-9851.63	-2441.96	-8004.12	-6833.87
-1149.07	-5367.43	-9594.11	-14178.1	-13739.2	-5379.48	-8465.62	-5248.22	-3428.46	-2287.43	812.248	4799.32	-8425.42	-6100	-4323.45
-3716.91	-4748.08	2875.61	-14744.7	-6138.91	2177.77	-10826	-1642.7	468.066	-110.684	-1635.12	-1077	1169.44	-3351.17	-2972.71
-1295.66	-2994.46	-327.631	-11359.2	-6017.6	-5849.28	-6672.59	-5319.82	-3588.04	-6168.63	2978.56	-4928.5	-843.646	-4461.31	-9889.98
-9854.07	-6729.01	-6726.95	-14948.9	-8816.76	-4637.05	700.029	-1821.12	-5020.96	-4287.06	1856.73	-3764.79	1076.5	-74.5176	-6237.2
-3107.48	-3321.5	-4111.47	-7640.42	-9126.61	-7870.59	564.545	-3563.39	-8907.76	-3109.56	-1521.26	-7799.23	-6680.93	-4651.77	-6790.43
-3749.03	-6263.56	6022.57	-14149.7	-4192.15	974.826	-7996.88	2730.68	-6040.76	457.163	2390.54	-5458.58	-2194.1	-965.638	-8150.52
-4398.55	-8500.73	-2540.63	-10026.2	-7472.99	-12685.2	-6639.88	83.0391	-6710.43	2514.48	2608.58	-4291.93	-1345.96	-977.45	-8871.58
-6911.67	-4579.53	-4292.05	-5601.06	-4642.71	-6508.65	-7928.49	-2952.5	-2153.07	-4123.76	-1409.54	-834.157	436.305	2775.46	-8044.84
-1028.48	-4542.11	-4119.06	-6826.45	-9935.14	-8342.48	-231.199	-4389.95	-3808.04	-7605.52	1709.5	-9271.62	-1436.75	-5955.2	-8004.37
-4005.69	-1906.49	-3362.21	-8449.01	-8215.26	-4043.82	-3446.4	-10400.9	4828.71	-10269.8	-1.25195	-6268.22	-4450.64	-5669.2	-8385.22
-650.484	-8395	-2721.09	-13671.5	-6225.51	-7852.65	-762.15	-2930.52	-4265.76	-2299.43	-3478.05	-2292.46	-1689.49	-3940.75	-11254.2

-5027.8	-8038.35	-255.463	-11528	-9789.74	-8609.11	-1248.71	585.727	3421.35	-1691.44	-2009.84	-4697.12	887.998	-10116.4	-7862.44
-6614.46	-12027.3	-3048.38	-11321.8	-3369.53	-6314.32	-2163.44	-668.238	-5189.34	-2371.88	-3059.9	-6942.31	1871.36	-343.543	-7099.69
1931.47	-5030.83	-1027.33	-17735.7	-8467.8	-6201.03	-4482.57	-3499.24	-245.707	-820.226	-7791.09	-5708.32	-6339.17	-842.221	-9231.83
89.2754	-6673.95	-10202.2	-4408.55	-6703.6	1914.11	-4383.63	-6688.43	-2993.47	-4586.66	33.5664	-6966.55	-3293.84	1879.1	-1102.13
2078.43	-8503.25	-552.201	-12202.9	-11200.5	-3296.65	723.359	6.05273	-7016.42	-1284.16	3579.4	-6987.27	-6172.84	3670.49	-7265.49
-5059.52	-1871.86	-6970.2	-11544.1	-9704.14	-4266.61	576.682	-3582.8	-4034.34	1104.81	1818.72	-7609.17	-7262.92	78.0273	-7548.53
1490.45	686.697	-4199.13	-12646.4	-5365.28	1107.73	-5329.96	-8306.04	1761.27	-5531.32	251.906	-7363.94	-4156.94	-8404.96	-12020.9
-4802.55	-8533.69	-2778.75	-10458.1	-8861.13	-497.053	-161.174	-6052.4	1079.79	-1014.65	-769.393	-1941.83	-7336.75	-7057.85	-10695.6
-1194.64	-9349.23	-2625.04	-9438.82	-6871.87	-3920.27	-12398.5	-8399.03	732.861	2288.29	-2425.81	-11240.9	-6302.92	560.434	-2657.99
-3360.05	-6469.71	-6769.04	-12414.7	-9909.41	-2887.61	839.697	-2204.49	7308.31	-11897.5	3569.79	-9823.16	-5332.33	-4401.86	-9786.33
-583.027	-2821.69	-7511.66	-12796.1	-14741.1	-11457.1	-1185.65	3345.43	1535.82	-10655	875.449	-8652.85	-2643.94	-2483.78	-5565.94
-955.373	-12025.9	-3153.93	-15305.8	-10839.9	-3851.63	-5963.26	-3508.12	-1746.38	-6903.21	1729.95	81.2666	-8225.7	-7542.24	492.145
-1080.21	-8926.58	-3782.74	-15586.1	-10168.9	2915.77	-7289.82	-1777.71	-3222.87	-3107.24	-1499.34	-8225.23	-9233.2	-6122.51	-6235.91
-8890.77	-6325.07	-2539.32	-12553.7	-5625.83	-5914.13	-2454.56	-6635.18	-5570.24	-2867.16	-2663.41	-1809.89	-6276.18	-4676.15	-11579.3
-4520.42	-7522.9	-4225.96	-11047.3	-4531.27	2071.39	-1649.16	-4169.36	-2262.94	1819.89	-279.346	-11582.3	-5949.87	-5821.43	2796.1
-10182.1	1742.71	-11708.1	-6728.28	-7802.45	-3852.1	-4293.24	-3090.01	-57.6328	-1847.57	2763.82	-4359.81	-2716.85	-1855.51	-3389.43
-1149.88	-977.895	-1991.7	-5979.29	-13095.9	-6533.86	154.117	-652.453	-5244.2	-5577.93	-247.67	-11797.2	-3382	-1490.91	-7748.86
-4681.68	-9534.52	310.752	-4227.7	-6451.18	-2199.04	-1733.66	-7607.87	-4085.34	-757.558	-450.043	-3750	-3522.96	-4572.71	-12364.2
-4447.78	-2954.64	-6110.9	-9710	-3540.18	-6670.53	-11049.6	-8579.55	-8646.66	-2931.86	3581.55	-6878.03	-2281.33	1639.64	-5202.56
-4327.53	-9946.75	-8477.77	-7991.77	-12691.3	-12118.7	749.025	-7810.6	-10047.7	-7308.32	-360.895	-11390.1	-6235.71	-5115.45	-6036.93
-3965.39	-7844.59	-4000.08	-9562.81	-13737.9	-4155.59	-8676.81	-5168.43	-5844.11	-4048.69	-2006.24	478.409	150.604	-5473.27	-8715.81
-10964.7	-9674.18	-4461.14	-11168.2	-1379.35	-12671.4	-4134.11	-1452.11	245.391	-4485.36	-5106.88	-1801.64	1847.59	-1954.41	-9032.42
1121.72	-2413.25	-2889.82	-11419.5	-9805.47	-8238.9	-8098.43	-6743.62	-4907.59	-2303.6	723.934	-3023.35	-1957.98	-4222.82	-1598.99
-4581.76	-2866.8	1772.47	-12933.3	-3694.68	-12238	-9792.45	-1457.82	-7780.1	-6456.86	-2851.32	-5087.38	2884.31	351.721	-5989.78
-6748.56	-6079.95	280.471	-9160.32	-5059.82	-7485.43	-1800.13	-7784.62	-7638.77	-3437.38	938.828	-7043.72	-1839.27	-5088.38	-12539.2
-7163.71	-8464.01	-4723.94	-9061.79	-4953.2	-1903.92	-2736.38	-5843.6	-5595.87	-148.831	-1475.4	-10811.9	-4906.4	-2903.11	-11445.9
-5483.75	-365.279	-7791.58	-12422.6	-12700	-1307.26	2492.43	1390.09	-1489.14	-4655.05	6571.68	-1985.43	-808.582	-5335.69	-10610.7
300.547	-4338.88	-4431.14	-12552.9	-11876.3	-5819.8	-4967.27	-3484.92	1640.84	-6104.38	-292.93	-6070.51	-4125.32	-1126.97	-1707.59

-2708.22	1016.59	1792.42	-12164.7	-16300.1	-7729.51	-7543.52	-3096.46	829.67	-14062.8	-1274.9	-9561.63	-5144.97	-4835.32	-9848.43
2281.28	-470.951	-3542.59	-14415.2	-9392.06	-2658.34	673.873	-413.58	-7868.17	-1405.58	-230.691	-6206.13	-5275.56	-4580.24	-11388.2
-761.334	-7307.82	-5301.16	-10320.7	-4570.79	-10908.9	-380.389	-4567.74	-3313.35	-3108.71	-7669.98	-6617.11	1254.12	-217.309	-10384.7
-5062.52	-5515.27	-4239.43	-8707.96	-8206.01	-3264.73	-1325.71	-6338.42	-2089.35	-9475.2	6856.92	-6643.58	826.125	3450.85	-9607.92
-4944.96	-11102.3	-730.037	-10926.5	-11293.2	-4797.26	-8116.29	-4591.02	-3174.58	-2043.44	4907.65	-6482.33	728.227	-7598.51	-7891.8
-927.84	-4165.47	-9748.1	-11981.5	-9336.99	-2863.76	-1451.15	-7442.29	-4801.02	-4092.04	-4862.54	-8161.13	-4394.21	-430.871	-12456.2
-3076.29	-5327.34	-2267.69	-6453.5	-18613.7	-4106.53	-1035.98	-5441.28	-1625.95	-2626.13	565.832	-2079.94	-5403.16	-3907.82	-10219.7
-2894.62	-10621.1	-4603.88	-14985.3	-6717.28	-3706.69	-6655.43	-3943.35	-7595.62	-1461.86	-4654.67	-6682.56	-7425.43	-9593.82	-5321.46
-2588.92	-4888.1	828.881	-10766.4	-8821.03	-8817.12	-4161.63	4452.21	-5309.11	-3753.22	7376.11	-7603.71	-8758.11	-5347.45	-4286.06
-5420.29	-6912.2	-6743.2	-6555.4	-1436.49	1434.54	2222.05	-5127.98	-6437.26	-2657.78	535.447	-2656.82	-2498.46	-6304.83	-6925.28
560.516	-11583.9	-2192.7	-13075.6	-10655.5	-6718.43	-10124	-5540.18	-1327.28	-1372.89	-4359.72	-12854.3	315.375	-10191	-7038.94
-9987.65	-5982.33	7562.89	-4801.96	-13398.1	-6514.42	-7430.15	-1245.45	-11079.7	-264.047	-295.234	-4108.83	-6575.84	-10441.3	-9156.13
-3121.84	-7481.41	2648.38	-9314.37	-6986.19	2297.86	-1897.67	-5806.32	-12179.5	-2845.04	-4259.83	-9374.81	-5729.37	-2524.96	-9255.54
-8662.02	-1215.45	-5571.19	-10605.6	-4989.57	-2040.04	-7514.09	-5225.72	-2950.59	-1783.35	-5936.27	-7699.44	-8891	1523.34	-6576.71
-4659.33	-4619.26	2282.65	-8151.03	-10985.4	-1752.67	1280.39	-8474.95	33.7324	2483.35	288.422	-8039.07	-5617.36	-12230.9	-9436.3
-6732.21	-9832.82	-4790.74	-10025.6	-14026.5	-7291.91	3585.92	-10819.4	-2649.15	3408.62	-5002.91	-5946.53	-5603.99	-7142.52	-6951.51
-4452.52	-6614.72	-4949.34	-15135.5	-10571.3	1193.42	-5858.47	-1872.68	-4455.58	-1601.59	5681.42	-9747.99	-2049.03	-2663.45	-8872.29
-1416.31	-7698.77	-6759.86	-15265	-8880.02	-5841.82	-3869.8	-4528.84	-3405.97	-4770.75	-2021.83	-5253	-3099.15	-7279.55	-13031.7
-6788.68	-212.406	-6625.18	-10132.5	-3084.14	-8476.94	-3056.9	3637.87	-5679.88	2106.27	3539.76	1685.71	-4921.96	-6046.23	-9691.87
-6849.17	-9715.21	-5210.32	-19846.9	-7079.33	-778.16	-2055.75	678.018	-7417.01	6548.5	-664.811	-6306.93	745.443	-2084.04	-642.07
-582.09	-4934	-2467.27	-11096.9	-6632.31	497.393	2852.15	-7215.42	-4841	688.631	-755.277	-1683.14	-199.449	-5594.68	-5088.23
1614	-3272.99	-3851.49	-15881.4	-2441.98	-7342.82	-4007.52	-10100.7	-10356.2	-5806.22	-2723.42	-2952.28	-10738.8	-9463.55	-5134.78
1270.12	-4698.45	-7443.9	-15148.2	-8066.64	-6802.49	-7068.44	4938.71	-7920.32	-7715.67	3328.04	-5841.54	-1832.12	-4268.68	-6056.47
-7264.51	-1908.13	-5357.91	-10276.7	-7470.14	-4886.79	-712.455	-2024.39	-2562.8	-7704.78	1770.19	-3355.17	-5594.29	999.346	-9774.65
-5701.61	-6445.81	274.191	-13223.4	-11307	-5447.37	1855.04	432.875	-7315.23	-3395.57	743.092	-7075.94	-7826.73	-2376.64	-7715.42
-6057.13	-2326.5	-1659.79	-13815	-10522.4	-6542.38	-4299.55	-107.455	-2281.97	179.536	-1959.11	-1490.37	-1153.86	-5760.73	-13686.9
-2124.51	-4649.3	-2179.55	-12578.5	-14077.5	-6466.77	-1683.51	-10031.3	-4246.6	-959.482	-2590.05	-4368.09	-4309.01	-7781.59	-5826.83
-1744.52	-2960.41	661.695	-7676.91	-10695.3	-1492.05	-4559.32	-7021.72	-5874.22	-4937.85	793.926	-6959.96	-521.066	-1441.6	-4800.41

-7606.42	-288.311	-179.719	-10985.5	-6951.96	-4458.5	-595.35	-5077.86	-6585.82	-2164.86	1607.4	-2702.77	-2933.94	2928.34	2093.31
619.232	-3340.15	-8603.51	-18068.7	-5321.63	-10571	-188.76	-8614.32	-2822.58	-3960.69	447.691	-9625.72	-2486.72	-3032.76	-6667.2
-5407.55	-6726.92	-6251.58	-13120.6	-5942.88	-14111.6	-5552.49	-4719.29	-2177.48	-4691.09	-809.762	-5633.67	-3452.06	-1449.53	-5012.69
-4869.91	-7259.38	-1784.85	-4221.53	-7950.27	-6747.94	-3775.19	1760.12	-3567.98	-11273.5	12.9141	-7127.73	-7825.17	-11535.7	-9762.02
-8078.65	-6753.4	-4045.74	-10721.7	-3148.15	-1159.75	2585.99	-4088.11	-2727.62	-5528.92	-5165.9	-5429.58	-9834.71	-3659.84	-12211
525.709	-3346.13	-2931.75	-22120.2	-7194.73	-94.832	-4224.85	-4756.65	-6038.25	-2453.6	486.666	-9896.68	-1587.11	2683.36	-9423.65
300.484	-2233.92	-3456.16	-9345.06	-10853.2	-4418.2	-8176.65	-6525.26	-6530.27	8302.41	-2075.25	-14651.3	-600.082	-8175.39	-1812.13
-9048.26	-1790.37	-406.355	-1476.73	-10898.5	-1976.37	-9533.34	-5332.1	-387.834	-8806.98	-444.875	-4301.08	441.34	-3095.53	-3178.37
300.484	-1850.69	-9317.52	-10356.9	-7717.2	-6793.25	-10352.2	-2157.49	-305.869	-8589.14	-3161.34	-7525.95	500.879	-5006.9	-5623.12
-5939.6	-4378.39	-8714.62	-9267.86	-9469.02	-5214.57	-1802.36	-2088.2	-234.426	-8333.15	-1487.23	-5164.26	-2026.55	-6767.73	-6951.29
-7302.5	-11651.8	-5491.75	-13168	-9762.67	-5498.51	-9453.51	-1728.83	4488.15	-9455.11	-4260.13	329.585	-1777.62	-4235.57	-1984.43
-3329.96	-6802.82	-986.002	-4507.78	-10709.1	-6967.95	-1125.74	1555.22	-4425.04	-8953.14	-1786.96	-6862.59	-6159.36	-6828.36	-4186.81
-6823.45	-6348.31	-4162.29	-11180	-15716.9	-8637.12	-3800.27	-4719.45	-8900.43	-2672.88	5295.6	-6302.78	-6522.01	-1953.79	-11660
-1547.33	-1537.05	-1166.38	-13285.1	-6905.74	-4138.56	-5231.96	-4892.55	-4601.5	-3908.99	-6741.46	-4060.95	653.729	-4750.07	-7008.39
1422.86	-3402.63	-6850.54	-14505.2	-5890.96	-3677.02	528.416	-2098.61	-3310.15	-11008	-5778.67	-6276.87	-4688.76	-2580.84	-9192.06
-3619.52	-8466.9	-3512.35	-9094.75	-9248.87	-4314.95	-1150.47	-3828.54	-1778.24	376.217	-2479.34	1418.84	-1674.13	-3117.46	-10422.4
-6174.66	-7478.07	-1284.96	-7889.97	-6876.61	-5468.5	-8702.76	-731.822	-5745.29	-4664.58	82.8047	566.743	-1147.34	-6902.47	317.793
-2839.08	-5292.11	-8724.92	-9503.9	-16568.2	206.994	-11804	-3749.99	405.826	-5302.66	551.539	-2506.17	-2734.58	-5784.93	-4776.59
-4075.55	-9915.04	-4758	-14994.2	-6341.19	-3580.92	-4007.66	-2985.82	-5130.7	-1219.24	-1746.34	-11088.2	-6603.54	-6763.51	-5991.13
-3198.16	-4066.73	-6223.09	-12457.5	-6398.61	-8502.26	6522.8	-7586.09	-2555.01	-5664.49	-9224.76	-5771.76	-7745.31	-5319.39	-6614.27
-6254.29	-3780.97	-9210.02	-8911.53	-10790.8	-19975.7	-3803.75	-100.844	-5256.5	2845.73	-231.375	833.392	-10199.2	-2988.14	-7465.26
-7444.25	-9209.94	-8361.29	-10092.7	-9767.36	-7479.08	-6752.17	-775.275	-7243.49	-8055.6	-529.209	-6388.86	-3705.36	-4606.53	-4184.76
-1278.71	-9557.9	-1734.42	-11693.4	-9011.8	-1694.04	-5638.42	-7238.96	1616.21	-8026.94	-737.133	352.875	-1135.75	-9508.58	-12416.3
-5753.74	-4387.29	-3006.9	-9394.74	-6878.54	2035.94	-5768.81	-9441.11	-3916.92	-4591.61	1099.64	-5963.94	-1484.66	-3306.64	-6031.71
-4209.5	-3987.72	-7843.52	-11557.3	-9698.57	-1431.54	-4174.84	-8457.53	1559.74	-7594.62	-1783.97	-3259.72	4073.73	-8641.08	-1080.45
-3858.07	-9323.93	1075.55	-15206.9	-13085.5	450.939	1704.56	-9039.68	-9418.05	946.834	4082.82	-8813.27	-3426.25	-3441.68	-12499.7
-3675.92	-6054.68	-1970.24	-9517.61	-10492.7	-4180.88	-5827.36	-4341.29	-3465.83	-11930	-2141.29	-8557.04	-1180.78	-5511.05	-1421.91
-8087.36	-12335.6	-3700.91	-11753.1	-5392.76	-5835.14	-3988.55	-3678.4	257.926	1188.94	-527.842	-10843.5	-5988.3	-5783.26	-7500.86

-4810.35	-7333.25	-1121.44	-9124.32	-7390.42	-5691.42	1804.06	-5384.99	-5710.56	-4361.25	1671.64	-5761.07	-8343.29	-9523.05	-10640.7
-2356.39	-1307.51	-4890.09	-12978.4	-10890.9	-11190.1	-1896.9	-8206.31	-1576.48	1903.06	-3872.46	-3486.34	-6300.58	-1762.73	-10527
-413.086	-64.8496	-2336.05	-15815.7	-12236.5	-5593.41	-112.107	-1976.77	-3776.31	2849.75	-5646.98	-5505.34	-3680.81	-5398.35	-11637.2
-6126.49	-1078.62	-1108.48	-14497	-9662.96	-6197.76	-5098.89	-3024.16	-3807.15	-6434.85	-771.445	-9491.26	-5324	-8023.32	-11764.2
-7286.86	-5724.85	-2547.7	-11624.2	-8888.43	1370.12	-6670.57	-5790.83	-165.721	-5597.06	2225.68	-1716.98	-8713.79	-4368.74	-13524.9
-2970.85	-11991.5	-2374.91	-13032.8	1200.07	-4817.12	331.24	-7042.33	4733.22	3429.93	3099.71	-5888.14	-7659.96	-10917.3	-5965.04
-6799.82	-11379.4	899.102	-15943.1	-4771.46	-1967.82	-3592.49	-7670.67	-10123	1736.03	-7082.04	-559.641	-6692.37	-7011.1	-9679.83
-6160.64	-7296.28	-902.984	-9537.39	-11377.2	-1698.11	-9327.45	-6341.48	-3466.53	-409.028	-2327.24	-7595.6	-941.695	-4878.5	-11026
543.658	-2585.17	4391.8	-14079.9	-9239.32	-3157.37	-1026.39	-5109.07	-1873.79	-9729.34	5031.98	-9859.23	4863.74	-3162.18	-11114.4
-11282.2	-7435.27	-1272.45	-5487.33	-10015.4	-4258.57	-6133.17	-719.342	-4318.63	72.7021	6040.55	-3048.15	-4955.21	-4057.55	-10229.2
-8133.97	-6789.61	-3629.9	-8749.63	-18281.1	-4085.87	-4878.59	-2578.83	-1155.87	-4529.38	-2.72266	-9632.22	-4132.5	2941.67	-2485.34
-2950.62	-204.691	-2913.07	-14664.7	-10593.1	1994.09	-9013.76	-7647.77	-2819.48	-3282.2	7476.81	-10323.2	-3901.49	-3232.76	-6805.43
-2870.83	-4301.97	-6046.52	-12204.2	-7632.88	-6957.27	1733.61	-1662.04	-2672.6	-8338.84	1022.91	-2135.43	-2012.81	-4092.41	-7690.36
-6790.67	-12935.6	-5286.5	-13559	-10796.8	-6248.01	-9111.89	-7567.94	-5599.04	-4701.65	-3950.95	-6792.25	-7922.19	-9269.51	-9620.82
-5619.66	-6267.18	-8979.65	-11787.8	-5233.78	-5919.59	-5245.84	862.041	-2538.82	4802.12	180.57	-760.522	4219.58	-1307.83	-6664.07
-5703.35	-7363.95	-6623.67	-8796.74	-14913.1	470.879	-14012	-5197.04	-2567.98	2052.1	-4137.73	-11077.5	-4077.07	-6419.62	-7104.85
1755.16	-2626.54	-264.563	-13279.2	-9527.76	-5444.37	-4964.38	-1398.72	1075.79	-5569.73	4556.5	-1960.16	477.664	-2456.14	-2404.05
-1439.6	-8305.64	-4640.96	-6242.88	-10300.5	-10336.6	-7137.7	-4315.36	-8279.45	1984.89	2752.59	542.304	-7589.35	-6029.6	-7142.21
-1486.08	-7874.03	-5941.85	-11666.3	-9248.71	-4094.19	6655.53	-5447.93	-1238.39	-1583.13	1557.46	-527.716	-3544.51	-3767.9	-7828.18
2988.23	-4080.03	-2440.96	-10919.2	-7866.28	-3412.91	-4449.54	-8355.78	-2240.74	-1298.48	-2787.13	-6831.81	-5745.96	-7451.47	-7417.6
-7686.74	-4015.18	1470.04	-9221.97	-13479.9	-10608.5	-3985.93	736.535	-5344.47	-4684.08	1325.94	-713.313	-2550.04	-5554.96	-12383.6
-6059.37	-7989.42	681.088	-8738.55	-11669.5	-4572.15	-7201.83	-8754.71	-2096.07	-2141.15	4389.35	-1903.91	-1904.4	-4635.81	-6703.94
-2110.5	-7984.98	-6818.71	-9288.49	-6979.99	-1946.5	307.387	-4581.26	-1357.37	-1217.39	1091.76	-10416.9	-4567.9	-4008.47	-7102.09
-8592.5	-702.645	549.033	-12835.7	-9934.53	-3079.58	-10363.5	-2828.38	-1669.36	-9553.89	-7303.98	-9553.44	-2600.62	-3245.05	-5087.06
-7260.01	-12428.8	334.119	-5931.35	-11683.8	-4521.81	-5186.07	-397.473	-801.256	-7307.17	-1725.08	-900.036	-3686.29	-4332.44	-14246.2
-2371.68	-9106.48	-1045.25	-9216.79	-9903.88	-7811	-4327.97	-4037.59	-3287.42	-1832.99	-2458.23	-8402.64	-6846.36	-7536.66	-9911.55
-5102.72	-6992.24	-5923.85	-16702.1	-7283.52	-6291.5	-5263.73	-5605.65	-1109.52	-6553.02	-1192.74	-14613.6	490.771	-4994.51	-9340.81
-8445.04	-6590.36	-5594.92	-11245.3	-687.822	-4329.4	-3175.79	4413.77	-5592.8	-2426.2	4089.54	-5472.1	-2439.96	-2428.29	-9381.77

-7261.26	-10462.4	-3187.16	-9795.98	-6886.16	-8707.37	-419.865	1908.8	-5498.5	-225.162	2458.51	-6798.49	-7367.04	-3531.15	-8932.23
982.309	-7831.62	53.9531	-11085.3	-12522.3	-671.83	-2931.63	-2769.6	-3071.38	-7261.86	-1236.63	-503.659	-4366.33	-5659.84	-7108.91
-3518.38	-5054.55	-727.016	-14358.6	-10031.8	-8623.98	2034.61	-2819.7	-10666.2	-6358.67	-1268.81	-7743.52	-7322.68	-5744.56	-5162.36
-6172.72	2007.79	-993.711	-12756.6	-6227.5	-9591.99	-1434.79	270.027	3887.35	-2075.46	4759.86	-6825.12	-11334.6	-1953.55	-11874.7
-3518.22	1037.85	1528.34	-8009.93	-1641.11	4117.96	1883.81	-4536.75	4565.83	-4531.41	-2170.58	-4637.71	1513.83	-1773.63	-7469.42
2961.2	-15996.8	-3765.25	-10234.5	-7488.23	-6426.7	-3863.42	322.533	-5420.66	-2567.12	-4865.25	-10951.2	-60.3027	-7573.64	-7654.86
4971.46	-5594.18	5161.37	-9702.35	-10736.2	-7360.71	-7612.5	-4103.98	-2269.9	-2725.25	1908.81	-871.471	-3385.48	-3499.57	-5318.68
-8860.28	-5227.59	-2945.1	-9063.83	-17607.2	-4250.63	-4208.04	-7469.22	-3146.09	-3386.93	-3536.97	-1222.26	-5480.92	-3845.34	-5611.85
-3839.87	-7257.43	-1889.29	-11607.2	-12452.6	-4259.84	-3162.25	-3387.4	92.3125	-2717.6	1519.65	-3120.38	-374.365	-4534.69	-9049.18
-5537.66	-8024.44	-6498.17	-6472.79	-13065.2	-1005.27	-5395.31	-3081.29	-7834.69	-1459.97	7707.75	1741.17	-6193.73	-8452.61	-9284.25
-3398.01	-4291.43	971.35	-11912.4	-11479.7	-3347.1	-4295.54	-5726.97	427.396	-188.898	-3570.98	-1706.95	-4045.27	-86.252	-6746.5
-7407.26	-8715.42	-5400.35	-9397.15	-10732	-9194.54	-7622.06	-1254.48	-3934.3	-223.057	3851.74	-12142.5	-6888.32	-1761.89	-3650.62
-3140.97	-13052.9	-7968.47	-13951.2	-12421.5	-9034.52	-6090.55	-3008.11	-3734.26	-2722.59	-2922.83	-3810.49	-8162.68	-1621.81	-9007.71
-11002.5	-4571.39	-2744.06	-10644.2	-6360.8	-1331.22	-7026.07	-8323.93	-1251.12	1589.24	-2530.68	-5928.23	-6775.79	1623.13	-10602.5
-12482.6	-2417.84	-1003.09	-10643.3	-10164.1	-3193.37	-4161	-8157.21	1446.3	7564.47	-349.719	-10356	-2360.35	-2908.69	-11804.8
-1715.99	-10838.4	-5633.59	-11339.5	-9244.95	-5952.04	3484.47	-442.707	1244.21	-545.072	811.26	-3229.46	-6437.66	1077	-4950.27
-5101.47	-1953.63	1902.77	-8957.61	-13348	-8803.78	-4578.54	-6982.7	-5745.42	-2856.93	-384.967	-4914.48	-4099.44	-7737.58	-5609.58
-5709.6	-4278.07	-21.7129	-11472.2	-7952.93	-5859.68	-10610.5	-2574.98	19.9121	-11927.6	-32.7773	-4036.62	-8060.63	-6081.96	-6638.14
-3373.13	-3674.38	-7847.54	-8944.56	-9690.34	-6543.18	-4478.49	-7934.04	-923.988	-4195.37	-3125.26	-5590.85	-5369.13	-4323.73	-10229.4
-6393.08	-8111.95	-9585.8	-14440.6	-7451.16	-3100.44	-1805.78	-8870.89	-466.766	114.357	121.373	-1944.65	-2470.43	-3920.52	-8438.42
-2987.2	-4552.83	-644.795	-8300.96	-8920.77	-8387.69	-8675.17	-4202.56	1191.93	-6766.47	-687.312	-616.749	-1419.34	-8486.06	-6789.51
-3877.05	-5099.44	-3891.35	-6774.99	-10718.5	-7846.19	-2310.82	259.529	-9384.34	-9218.84	683.541	-4615.71	-7234.96	-4385.84	-6871.67
-3419.2	-498.227	-5454.8	-15567.6	-5664.9	-6942.77	-3889.75	-4588.59	-6971.99	-3098.74	548.879	-3737.71	-6399.08	-6213.67	-11400.8
-2343.03	-5618.26	820.219	-9004.36	-5530.84	-3806.23	-2771.63	-1435.38	-3147.67	-1069.46	-2230.79	-1149.48	-7664.11	-1527.81	-8458.67
-7147.98	-8598.66	13.8555	-12222.8	-7463.82	-1593.75	-578.088	-2263.87	-6378.35	511.152	2477.59	-13770.7	-3834.47	-6219.54	-7673.38
-5306.38	-7239.39	-5170.58	-12325	-6660.06	-14571.3	-4805.74	117.154	-5053.88	-4419.71	-2213.23	-2803.89	-1812.05	-8357.69	-7444.23
-8398.62	-8292.43	-3566.16	-16051.2	-13988.8	-6164.56	-2476.29	-6297.67	-4009.34	808.463	1737.3	-9877.15	-5988.6	-3368.54	-7582.54
-1936.75	-14613.7	-3579.83	-14283.1	-4625.25	-6420.37	-1580.7	-2840.48	1369.22	-785.919	-2746.51	-1869.52	-6500.09	-5917.91	-9202.61

-10344.6	-5410.41	-6051.73	-7831.59	-7745.33	-673.883	-7088.49	-5472.97	-967.1	-1977.01	-5418.3	-4375.3	-7258.73	-9712.31	-13930.7
-4009.34	-5231	-2253.11	-11454.8	-11676.2	-8448.76	-8660.21	-7181.04	-2613.56	-1123	-5117.35	-4422.15	-4781.96	-10775.8	-3531.26
-4503.97	815.937	-7117.91	-8124.33	-6831.13	-1933.65	-3903.25	-4029.59	-2216.37	-3375.74	-5881.63	-7312.71	-3681.7	-2713.23	-7708.52
-5697.08	-6602.96	129.697	-4206.6	-8791.13	-13385.8	-5120.12	-3346.21	-3521.34	4705.59	438.695	-6919.04	-386.029	-3090.75	-8101.59
-8209.83	-8830.69	-4244.2	-7257.48	-9304.69	-4061.85	-5519.67	-7484.46	106.562	1458.25	-5271.65	-2700.65	2498.64	-5267.42	-1179.1
-2818.04	-2446.57	-5604.97	-6495.6	-12649.7	-1786.2	4301.42	-9363.34	-7725.51	-4956.98	-2397	-7579.05	-3610.79	-7978.65	-8424.31
-504.924	-13573.4	-1807.31	-13709.2	-10408	-7199.88	-5999.53	-5505.84	-7509.6	-5079.95	-1433.86	-8701.08	-1529.2	-8907.24	-11252.8
-2562.76	-3163.34	-6651.64	-13597.6	-4826.26	-5921.56	-4856.87	-1587.13	-2724.35	-5942.22	-80.4688	2369.09	-2653.53	-4021.48	-4954.86
-6240.18	-7753.56	-5987.63	-13364.5	-19544.6	-6034.38	-3070.5	-4668.49	-1174.07	-7490.3	-1877.99	-6650.55	-21.7207	-1293.36	-9776.24
-1971.46	-4128.72	-153.807	-8080.75	-10083.6	-8463.65	-4642.67	-10352.6	-1343.71	-2903.83	-4386.61	-5542.29	3731.57	-5441.01	-11807.3
-3750.16	-6503.49	-4267.8	-15434.1	-5698.28	-4579.75	-2738.99	-56.3516	-6908.24	-1826.19	-782.494	-4536.41	4149.64	-4536.93	-8058.47
-6320.18	-969.785	-1115.71	-13631.5	-6964.62	-3301.92	-7516.81	2671.56	-6528.79	369.532	-3877.08	-2452.33	1154.96	-8062.22	-3685.01
-3239.18	-5784.23	-5179.16	-10139.7	-14692.7	-5123.97	-4968.98	-2152.38	-4793.67	-2136.06	5572.37	-4548.86	-3506.86	-4655.24	-9436.95
-4034.69	-5640.77	-6024.59	-13588.2	-12002.2	-1889.59	-2796.15	-3261.93	-5805.81	-8228.57	-849.496	-4782.27	-2616.69	-639.401	-10693.5
-5785.58	-7444.08	-5541.47	-13934.3	-6107.46	-3488.54	-1093.59	-8028.2	-6194.37	-107.619	4141.01	-174.356	-3881.61	-3764.95	-7654.97
-5253.56	-1475.36	-1426.64	-17998.5	-8252.88	-5867.5	-6482.08	-6623.22	5283.89	537.15	-3037.5	-3978.43	-5951.05	-8774.23	-6277.92
-6247.54	1277.2	-2781.17	-11541.1	-14727.3	-6349.65	-2107.15	-4842.67	3255.44	-4254.56	-7164.88	-2569.07	-7606.94	-2743.42	-6911.98
-3602.97	-7181.34	-6868.86	-13226	-11722.6	-3878.84	-6580.32	-8241.95	-8711.67	-4642.72	-1114.34	-3194.29	-2296.4	-9042.78	-5431.58
-7490.48	-6433.11	-3851.17	-3954.17	-10964	-2994.44	-2826.46	-7575.19	270.393	-9846.4	-5867.24	-4675.79	-2126.19	-5457.65	-6267.46
-7953.13	-8956.22	-11638.9	-14100.7	-12596.9	-2782.11	-6866.98	-9979.93	-2177.81	-5030.32	-6141.58	-3688.81	-1907.44	-3154.48	-11392.2
-4776.48	-587.314	-6710.86	-20806.4	-8327.28	-3321.92	-10869	-2838.11	975.062	-4445.67	-1383.08	-8293.45	-3753.38	-8681.27	-8982.75
-3660.38	-7602.24	-7358.74	-12222.3	-7204.77	-750.428	-2336.41	-9122.48	1904.48	-10686.7	-330.865	-10574	4911.06	-3450.34	-11317.3
-467.746	-5051.64	-1555.63	-13109.4	-13363.2	-6625.22	-1895.3	-7959.26	-4002.32	-6196.74	-3778.32	-3558.67	-2069.76	-8288.72	-12537.9
-2224.59	-4338.12	-3470.3	-7680.37	-7726.45	-9685.93	-2322.22	-1725.56	-2198.69	-5878.23	-4990.13	-4560.94	-5737.37	-6315.73	-6476.3
-5377.3	-3240.06	-503.717	-11656.9	-7664.23	-8833.04	-6643.3	-422.975	-3449.37	-5520.17	-4655.58	-4338.28	-8431.05	-494.784	-6648.95
-5200.02	-2396.82	-5160.4	-19823.2	-14191.5	-7572.26	-6994.27	-979.051	-5968.69	-1818.03	-5222.71	-6854.77	-7000.17	-2028.57	-6087.64
-5458.43	-13644	-3231.59	-11109.1	-8160.94	-5165.65	-1558.42	-3447.29	-10397.4	3887.07	-7067.19	-6161.44	-5936.39	-10971.8	-9632.12
-11461.3	-10132.4	-4382.29	-12188.4	-14374.7	-4835.23	3495.84	-1499.41	-4504.1	-1491.99	-5883.43	-14316.3	-4984.57	-5093.34	-14994.5

-7871.75	-12410.5	-4533.19	-6419.12	-11253.9	-2446.67	-2429.04	-3273.81	-5355.02	-8157.63	-4195.79	-7298.13	-11575.6	-8658.4	-2115.53
2375.77	-4209.33	-1504.61	-11290.2	-12518.4	-4457.67	-5812.3	-1171.43	-7667.82	-4598.81	-457.316	-2938.44	-3338.49	-4932.27	-4274.52
-3340.35	-11492.1	-1468.76	-8768.72	-6857.58	-7269.52	-3981.29	3753.24	-1429.27	-1537.34	-2334.66	-3765.78	-5736.14	-3662.18	-5700.61
-2089.08	-6258.7	2438.95	-3744.77	-9443.33	-3365.96	-7638.03	1021.44	-3159.08	181.014	4346.75	-4899.44	-4581.57	-2801.39	-10822.7
-6581.25	-7588.69	166.219	-17675.2	-12287.1	-5600.01	-6217.04	-779.521	-5319.1	-1701.34	5677.97	-7590.48	-627.445	-1542	-10722.4
-6326.96	-12011.9	-1923.94	-11375.7	-10428.5	-10825.6	-5431.79	-3772.63	2831.37	5267.23	3616.52	-2773.95	-814.471	-2711.56	-10666.8
-3152.49	-9797.98	-694.906	-11994.7	-8615.36	-5234.4	-9401.55	-3178.17	-7153.44	-1129.87	-467.199	-10056.2	-8610.33	-3492.46	-6166.64
1663.35	-7271.56	-3282.25	-10733.6	-2014.7	-5800.94	-102.936	-730.662	-710.979	150.732	1341.62	942.062	-5973.34	-9477.47	-6445.92
-1864.67	-6809.54	-8662.64	-9263.8	375.008	-6789.12	2387.25	-8535.13	-2765.05	-5890.23	2890.37	-13891.4	-3649.3	719.523	-4644.6
-10513.3	-3293.37	-7040.1	-10409.3	-8636.78	-5909.94	-5827.05	-7811.38	-4186.79	-2703.46	-1914.51	-2706.51	2115.48	-1504.68	-6000.94
-6336.95	-2673.25	-402.738	-5434.95	-7852.88	-6965.9	-554.967	-5728.63	-6260.86	-4050.75	-1949.93	-578.038	-2243.53	-4008.42	-9271.84
-7194.14	-1337.13	-6896.04	-15624.8	-6483.68	444.338	-3681.28	-3341.48	-4107.27	-2474.73	-314.418	-2157.37	-1535.2	-3444.89	-11178.1
-3583.46	-7663.29	-3389	-7914	-7685.69	-6718.62	-3056.8	-3689.23	-1756.64	-3854.89	4413.75	-7701.18	-4616.63	6883.03	-8015.29
5664.67	-5170.09	1200.25	-8793.57	-3583.82	-6457.21	-4225.79	-4087.29	-3936.68	-6842.54	-656.625	-7898.7	461.594	-4720.27	-468.422
278.258	-8088.19	-3660.38	-4074.93	-6770.95	-3021.54	-6786.49	-808.289	-2541.55	-870.3	2409.2	-6686.64	-3690.66	474.305	-5128.37
-4394.08	-6230.76	4177.33	-6955.74	1165.76	-4660.79	-7517.79	25.8242	-1892.2	1656.82	-2599.81	-6238.41	-5790.5	-2313.01	-6240.42
-3686.48	1231.33	322.715	-9604.51	-10600.8	-1568.54	-5561.24	-4355.03	-2914.37	-9207.62	-22.666	-8073.79	-2284.04	-2769.84	-11378.7
-8955.95	-6252.99	-1508.42	-13638	-8238.33	212.207	-365.045	-10023.3	437.26	-10884.1	1627.55	-3311.44	47.4473	-2056.53	-1985.03
-5119.8	-2936.2	-1116.67	-12523.1	-4766.46	-4976.98	2624.42	-115.568	-8270.31	-4502.94	-1228.09	-1801.89	304.525	-5705.42	-5779.09
3683.04	-5640.41	-4817.84	-9047.12	-6782.98	269.865	-2508.72	-6855.67	-1931.65	325.219	-698.133	-8967.93	-2422.52	-5673.95	-4694.81
-2007.17	-8892.83	-96.0664	-9361.51	-15065.2	-7079.47	-3180.21	-2795.7	-4475.04	-7529.63	-7797.2	-7029.22	1540.9	-3880.86	-4899.28
-3606.03	-5387.36	-742.555	-11499.1	-10766.2	-9470.08	-3707.88	-4481	-6855.96	-3421.13	-5393.39	764.379	2013.32	-5429.75	-4812.98
-1328.25	-4698.87	41.1172	-8137.91	-8232.05	-2307.26	-2159.29	-912.361	-4459.94	1448.21	1892.08	-2459.95	698.938	-6478.24	-5768.57
-926.092	3638.66	-2628.33	-10622	-5364.85	-2104.38	-1432.73	-7032.24	-1485.84	389.142	4807.07	-5473.91	-6169.47	-1648.07	-6436.59
2420.94	-6423.42	5213.15	-6634.82	-8074.75	-7340.69	9364.23	-233.643	949.863	-5312.78	3033.37	-4624.79	-7144.17	-5885.14	-4614
-3453.63	-63.7871	997.816	-6747.96	-9204.97	-6042.12	-1821.92	-740.559	3579.77	-3047.19	6656.24	-1084.21	-6131.59	-1732.82	-4512.13
4020.95	-2705.3	-1711.18	-11069.4	-5246.85	-1949.24	-1638.21	-789.459	2841.53	1565.72	-4743.79	-3747.56	1340.87	5266.89	-3948.55
4973.11	958.908	1782.44	-7623.37	-8997.79	2235.4	-5999.64	4157.61	2282.25	-4148.36	-241.918	-53.8047	-1378.28	-5723.15	-4506.67

-1031.6	-2114.43	2712.39	-6252.87	-2321.46	2369.36	-2775.79	4952.3	-2246.25	-2042	2966.88	1482.1	-2778.57	791.857	-7605.38
1382.94	-799.932	-2023.65	-7432.73	-2389.23	277.049	1937.9	-492.85	-2213.63	-1829.51	-264.928	-2944.37	-691.799	2547.55	-5819.16
-12349.7	-2865.86	-2442.47	-6838.38	-4339.71	-1146.27	-3678.38	3602.65	-289.18	4087.55	3261.39	-440.441	964.607	-71.9883	-3769.63
1257.1	-1003.32	1768.93	-7575.87	-229.85	-1807.88	-382.936	2949.22	-586.729	-3129.98	-2549.91	-1816.23	1930.35	-872.729	-3647.89
2547.89	1800.42	3558.97	-1605.07	-1852.69	-1943.56	2818.11	2994.69	-3522.99	4957.92	-780.518	-3005.74	514.617	528.439	2166.36
4328.53	-3951.91	-4275.47	-8096.37	-6473.25	-2143.99	-4441.87	-594.176	-1648.52	-2257.88	6341.22	-1053.44	865.412	-4245.29	-1652.41
-1533.43	2390.5	1681.9	-6955.71	-4998.25	-1636.9	127.51	3042.45	-950.971	1473.06	3145.36	-43.1631	-1045.26	700.645	739.328
5886.43	3663.96	2520.37	-7643.05	-674.039	2790.11	1719.52	1521.15	-2014.93	-992.718	1653.07	-459.041	995.266	1391.61	68.7168
3669.55	-2414.74	-534.715	-3758.88	-282.518	-253.301	2456.44	6421.34	44.8828	-440.343	8402.55	-4185.08	-379.254	3939.7	-100.012
1811.57	4717.15	-2834.23	-1135.87	1561.16	2026.76	2992.76	4129.26	501.148	1493.04	6664.58	1103.6	1864.88	6937.81	-76.248
5361.37	-1543.16	7319.6	-2900.61	-2805.82	1999.22	4006.29	4221.99	-880.846	550.187	6120.81	1044.01	2867.87	6449.57	1180.17
4614.96	4908.94	3288.66	-149.912	-1633.06	111.146	5612.7	6942.29	2403.7	-389.013	4807.61	2645.59	6302.8	1849.08	187.43
5091.32	2398.34	3679.11	-4630.64	-6637.33	4121.6	2027.28	4563.14	3840.61	1841.9	5075.03	5938.31	-993.496	4316.16	-674.729
7370.42	4734.96	5599.9	-4549.74	-4008.17	5670.55	4013.82	6887.87	-444.6	3867.35	8641.26	2261.78	-930.809	5129.03	-3543.29
3942.45	6119.1	5644.61	1024.57	-2367.97	5206.48	6464.66	3187.65	1413.91	5109.62	8102.51	7151.9	1716.74	5205.14	587.443
6005.18	4621.51	4800.61	-666.064	-1806.43	2527.58	2854.1	5814.86	2786.57	5896.67	9557.17	6253.74	3271.21	5058.13	326.822
8570.9	4220.23	10085.9	-1309.73	7022.54	2660.39	2096.33	120.32	4765.15	9793.43	9483.5	6021.41	11013.4	5841.14	3470.82
4772.86	8933.26	7114.94	3326.7	6317.2	-848.799	6262.96	6648.55	5921.01	5416.16	5295.42	6034.64	2777.3	2974.5	-1393.14

Table 1. (continued).

S: number of wine sample.
